# Surface-Enhanced Raman Spectroscopy for Viral Diagnostics: Principles, Strategies, Clinical Challenges, and Future Directions

**DOI:** 10.1021/acs.chemrev.6c00201

**Published:** 2026-07-30

**Authors:** Yanjun Yang, Yiping Zhao

**Affiliations:** Department of Physics and Astronomy, 1355 The University of Georgia, Athens, Georgia 30602, United States

## Abstract

Viral outbreaks such as SARS-CoV, MERS-CoV, and COVID-19 underscore the urgent need for rapid, sensitive, and scalable diagnostic technologies. Current standard methods, including nucleic acid amplification tests and immunoassays, offer complementary strengths but face limitations in cost, turnaround time, and early-stage detection. Surface-enhanced Raman spectroscopy (SERS) has emerged as a promising alternative, leveraging plasmonic nanostructures to amplify weak Raman signals and provide molecular “fingerprints” of viral components with single-molecule sensitivity. This review provides a comprehensive overview of SERS-based virus detection, highlighting the fundamental principles of Raman enhancement, the role of substrates and hotspots, and analyte-specific challenges. We categorize sensing strategies into direct detection of intact viruses and components, affinity-based approaches using antibodies, aptamers, or viral receptors, and labeled methods that amplify specificity and multiplexing. Advances in nanofabrication, receptor engineering, and machine learning have significantly improved sensitivity, reproducibility, and classification accuracy, with detection limits reaching down to a few viral particles per milliliter. Despite these advances, challenges remain in handling biological complexity, ensuring reproducibility, and translating assays into clinical practice. We conclude by outlining opportunities for integrating SERS with portable devices, standardized spectral libraries, and artificial intelligence, paving the way toward rapid, robust, and deployable viral diagnostics for future pandemic preparedness.

## Graphical abstract

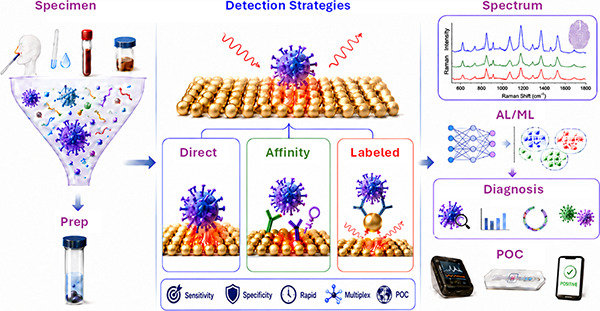

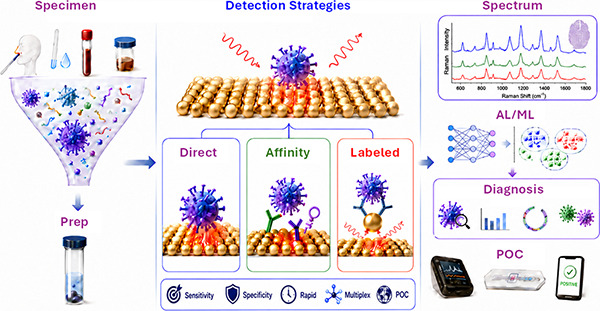

## 1 Introduction

Infectious diseases have repeatedly reshaped human history, from plague and cholera to influenza pandemics. In the modern era, emerging viral threats including severe acute respiratory syndrome coronavirus (SARS-CoV),^1^ Middle East respiratory syndrome coronavirus (MERS-CoV),^2^ and coronavirus disease 2019 (COVID-19)^3^ have posed unprecedented global challenges. Effective pandemic response requires not only vaccines but also rapid and accurate diagnostics, particularly for early-stage detection when large-scale screening is essential.^4,5^ Current viral diagnostics fall broadly into nucleic acid tests^6–8^ and serological (immunological) assays.^9^ Nucleic acid tests, most notably polymerase chain reaction (PCR), directly detect viral genomes with high sensitivity and specificity,^10,11^ and remain the clinical gold standard. PCR enables detection across diverse specimen types, including blood, saliva, swabs, and stool. Related approaches include nucleic acid hybridization,^12,13^ which relies on sequence-specific probe binding, and next-generation sequencing (NGS), which supports viral discovery and variant tracking.^14,15^ Despite their analytical power, these methods are resource-intensive, time-consuming (typically 1.5–4 h), and costly. During the COVID-19 pandemic, PCR testing incurred a median cost of $96 per test in the United States,^16^ and an estimated ¥352 billion (∼$52 billion USD) in China in 2022.^17^

Immunoassays include tests that detect viral antigens and serological tests that detect host antibodies. Techniques like enzyme-linked immunosorbent assay (ELISA)^18,19^ and lateral flow assays (LFA)^20,21^ are widely used due to their simplicity, speed, and cost-effectiveness. ELISAs are common in human immunodeficiency virus (HIV) and hepatitis diagnostics,^22–25^ while LFAs serve as essential tools for point-of-care (POC) testing.^26–28^ However, antigen tests can support early diagnosis but generally have lower sensitivity than nucleic acid tests, whereas antibody-based tests suffer from delayed detectability because antibodies take days to weeks to develop and can yield false positives due to cross-reactivity. A Cochrane review of COVID-19 rapid tests reported only 69.3% sensitivity.^29,30^

Taken together, nucleic acid and immunoassays provide complementary but incomplete solutions. Preparing for future pandemics requires diagnostic platforms that integrate speed, sensitivity, specificity, multiplexing capability, and scalability. In this context, surface-enhanced Raman spectroscopy (SERS) has emerged as a promising alternative. Discovered in the 1970s,^31–33^ SERS exploits localized electromagnetic field enhancement at plasmonic nanostructures to amplify otherwise weak Raman signals, enabling molecular detection down to the single-molecule level.^34,35^ Its narrow, well-resolved spectral features produce molecular “fingerprints”, allowing discrimination between closely related viral strains and biomarkers.^36^ Furthermore, SERS can support multiplexed analysis and, when integrated with portable Raman instruments, holds great potential for rapid, on-site, POC diagnostics.^37,38^

SERS-based virus detection gained momentum following Vo-Dinh’s 1998 demonstration of SERS-active primers for PCR-based HIV DNA detection.^39^ Subsequent studies, including the demonstration of distinct SERS signatures for respiratory viruses,^36^ catalyzed rapid growth of the field. Today, SERS-based viral diagnostics generally follow three complementary strategies: direct, affinity-based, and labeled detection. Direct detection measures intrinsic SERS spectra from intact virions^40,41^ or purified components such as proteins^42,43^ and nucleic acids.^44^ While simple and rapid, this approach faces challenges from biological background interference and limited hotspot accessibility for large analytes. To address these limitations, chemometric methods, such as principal component analysis (PCA),^45^ partial least-squares discriminant analysis (PLS-DA),^46,47^ and machine learning (ML),^41,48^ have been increasingly applied to enhance spectral classification and quantification accuracy. Affinity-based detection introduces specific capture molecules such as antibodies,^49^ aptamers,^50^ or viral receptors^51^ onto SERS substrates to selectively bind viral or host targets. This approach improves specificity and reduces matrix effects by concentrating analytes within plasmonic hotspots.^46,52–54^ Depending on the target analyte, affinity sensors can be tailored to detect viral nucleic acids, capsid or envelope proteins, or host-derived antibodies, cytokines, and metabolites. Labeled detection employs SERS tags, nanoparticles (NPs) carrying Raman reporters and biorecognition ligands, to amplify weak signals and enable quantitative and multiplexed analysis.^55–57^ These tags form sandwich-type assemblies (“substrate–analyte–SERS tag”) or signal-on/off configurations that provide exceptional sensitivity for both viral and host biomarkers.

Although fewer in number than PCR- or immunoassay-based studies, SERS investigations have demonstrated remarkable versatility across viral- and host-derived targets. Advances in nanofabrication, plasmonic substrate design, and data-driven analysis have substantially improved the signal strength, reproducibility, and analytical robustness. Combined with molecular fingerprinting, multiplexing capability, and compatibility with portable instrumentation, SERS is increasingly recognized as a strong candidate for rapid diagnostics and real-time public health surveillance.

In this Review, we present a comprehensive overview of recent developments in SERS-based virus detection, with a focus on detection strategies, target analytes, and translational applications. Section 2 introduces the core mechanisms of SERS, hotspot localization effects, and different SERS substrate geometries used for virus detection. Section 3 outlines the potential viral and host-derived analytes and introduces the three detection strategies: direct, affinity, and labeled detection. Section 4 details direct detection with different configurations, describing how intrinsic viral components yield spectral fingerprints under ideal conditions, while also highlighting challenges such as weak signal strength, background interference, and the need for machine learning for reliable interpretation. Section 5 focuses on affinity-based SERS assays, dissecting the role of molecular recognition elements such as antibodies, aptamers, and host receptors in enhancing specificity and capture efficiency, along with practical considerations in sensor fabrication, surface chemistry, and detection of a range of viral targets. Section 6 discusses labeled SERS strategies using engineered nanotags to boost sensitivity and enable multiplexed detection, elaborating on tag design, signal generation mechanisms, and their successful deployment for nucleic acids, viral proteins, and antibody assays in both research and clinical contexts. To bridge laboratory innovation and clinical relevance, Section 7 explores how successful virus detection using SERS requires careful attention to the entire diagnostic pipeline, including specimen selection, matrix-specific sample preprocessing, biosafety handling, and integration with microfluidics and LFA platforms for automation and POC readiness. Section 8 systematically identifies and analyzes the key technical and translational barriers across all detection strategies, ranging from intrinsic signal weakness and geometric mismatch to substrate variability, batch-to-batch inconsistency, and lack of standardization in clinical workflows, while providing mitigation strategies that incorporate nanofabrication advances, optimized chemistries, computational tools, and standardized validation frameworks. Section 9 concludes by evaluating the overall promise of SERS for virus diagnostics, demonstrating its unmatched capability for molecular fingerprinting and multiplexed detection, while also emphasizing the need for scalable manufacturing, robust signal processing, and clinical validation to realize its full potential for pandemic surveillance and POC diagnostics. Through this Review, we aim to clarify the current state of SERS-based viral detection and catalyze future innovation toward standardized, high-performance diagnostic platforms.

It is important to distinguish between a SERS-based biosensor and a SERS-based diagnostic. A biosensor is an analytical platform that converts a biological recognition or molecular interaction event into a measurable SERS signal, such as a spectral fingerprint, reporter intensity, or machine-learning classification output. A diagnostic, however, is a validated decision-making tool in which the biosensor is embedded within a defined clinical or public health workflow. Therefore, diagnostic performance cannot be judged only by analytical metrics such as the limit of detection, enhancement factor, or classification accuracy. It must also be evaluated in relation to the intended use case: the disease being tested, the infection stage, the target population, the specimen type, the end-user, the testing environment, the turnaround time requirement, the decision threshold, and the clinical or public health action triggered by the result. This distinction is particularly important for viral diseases because the optimal diagnostic target and workflow differ substantially among acute respiratory infections, chronic bloodborne infections, gastrointestinal outbreaks, neurotropic infections, and population-level surveillance.

## 2 The Principle of SERS

Because the theory of SERS enhancement and the design of SERS substrates have been extensively reviewed elsewhere, this section provides only the background needed to understand SERS-based viral detection. Readers seeking more comprehensive treatments of Raman/SERS theory, electromagnetic and chemical enhancement mechanisms, plasmonic hotspots, and substrate design are referred to several excellent reviews and monographs on SERS fundamentals, plasmonic nanostructures, and analytical SERS substrates.^58–61^ Here, we focus on those aspects most directly relevant to viruses, including hotspot accessibility, analyte size effects, substrate reproducibility, and compatibility with clinical specimens.

### 2.1 From Raman to SERS

Raman spectroscopy probes molecular vibrations through inelastic light scattering, producing sharp, chemically specific spectral fingerprints,^62,63^ as shown in Figure 1A,B. Despite this specificity, spontaneous Raman scattering is intrinsically weak—only ∼ 1 in 10^7^ incident photons undergo Raman scattering,^64^ severely limiting sensitivity for trace analytes such as viral proteins or nucleic acids. This limitation was overcome in the 1970s, when molecules adsorbed on roughened silver surfaces were found to exhibit Raman signals enhanced by several orders of magnitude (Figure 1C).^31,32^ This phenomenon, termed surface-enhanced Raman scattering (SERS), enables detection at ultralow concentrations, approaching the single-molecule level.^65,66^ SERS enhancement arises from a combination of electromagnetic and chemical mechanisms.^58^

**Figure 1:**
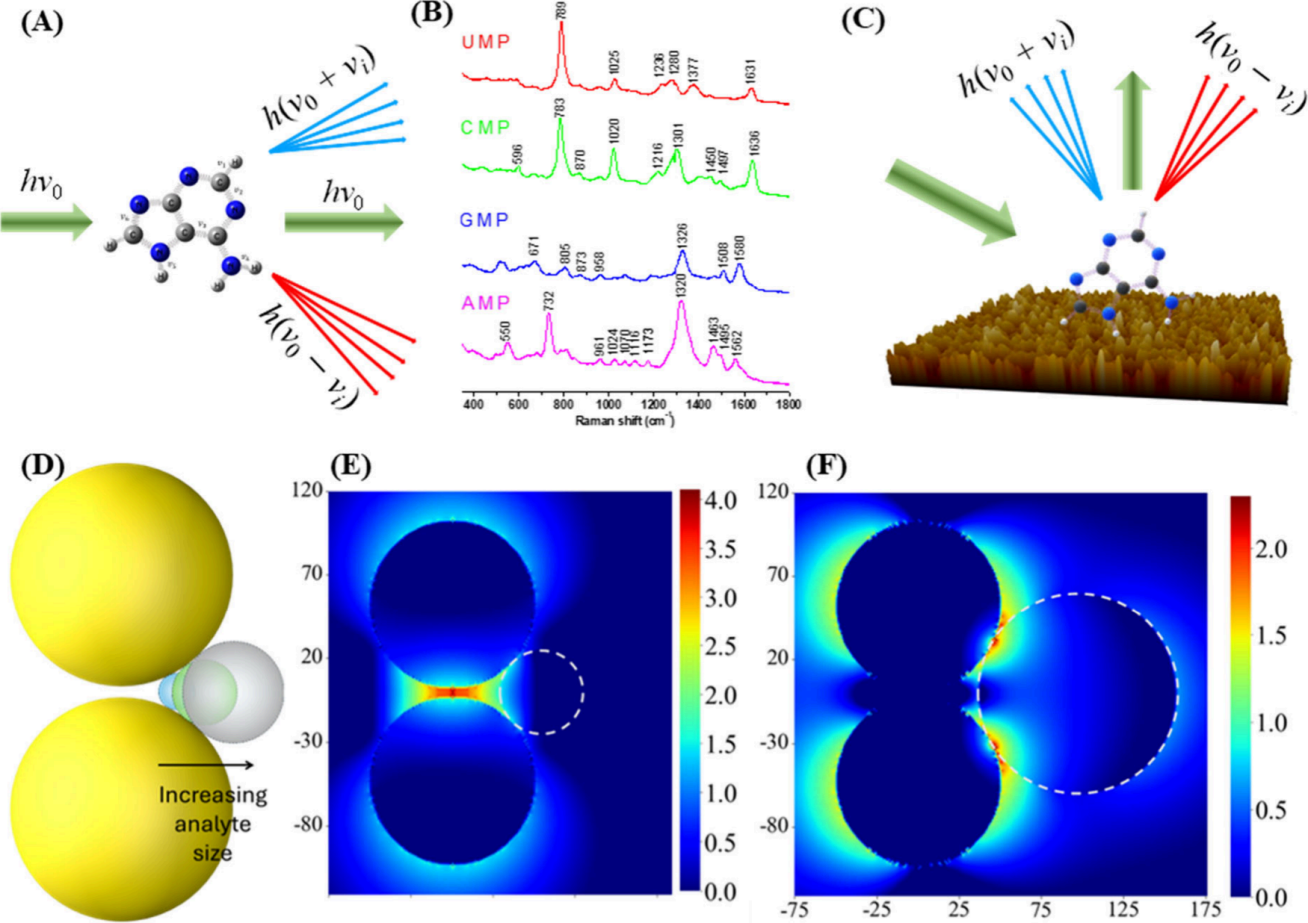
(A) Schematic illustration of the interaction between an incident electromagnetic wave and a molecule. (B) SERS (Raman) spectra of AMP, GMP, CMP, and UMP. (C) General schematic of the SERS effect. (D) Geometry of a spherical analyte positioned between an Au NP dimer. (E) Finite-difference time-domain (FDTD)-calculated local electric field distribution *log*_10_ |*G*_*l*_|^2^ for a 10 nm analyte under vertically polarized excitation (white dashed circle indicates the analyte) and (F) corresponding field distribution for a 120 nm analyte under horizontally polarized excitation. Panels D–F are reproduced with permission from ref 78. Copyright 2024, Royal Society of Chemistry under CC BY-NC 3.0 https://creativecommons.org/licenses/by-nc/3.0/.

While conventional Raman spectroscopy has been widely applied to fundamental studies of viral structure and composition,^67–72^ its diagnostic utility is limited by low sensitivity and strong interference from host biomolecules in clinical matrices such as saliva, blood, and transport media. Consequently, most Raman-based virus studies rely on purified samples, high viral loads, or long acquisition times, conditions that are incompatible with rapid, sensitive, and reproducible clinical diagnostics.

Ultraviolet resonance Raman (UVRR) spectroscopy offers intrinsic signal enhancement by exploiting electronic resonance with nucleic acids or aromatic amino acids and has been used for viral characterization with improved sensitivity.^73–77^ However, its clinical applicability is restricted by UV-induced photodamage, strong autofluorescence, shallow penetration depth, and the need for specialized, nonportable optical systems. In contrast, SERS combines large enhancement with visible or near-infrared excitation, flexible assay architectures, compatibility with complex biofluids, and integration with affinity capture, microfluidics, and lateral flow formats. These features have established SERS as the most practical Raman-based platform for viral diagnostics rather than viral characterization alone.

### 2.2 Electromagnetic vs Chemical Enhancement

The dominant contribution to SERS originates from electromagnetic (EM) enhancement, which can increase Raman intensity by factors of 10^6^–10^8^.^79,80^ This effect arises from localized surface plasmon resonances (LSPRs), collective oscillations of free electrons in metallic nanostructures excited by incident light.^81^ At resonance, the local electromagnetic field near the metal surface is dramatically amplified, and molecules positioned within this region scatter Raman photons much more efficiently.

In contrast, chemical enhancement (CM) provides a smaller yet crucial contribution to the overall SERS signal, typically yielding an intensity increase of about 10–100-fold.^82–85^ It arises from short-range interactions between the analyte and the metal substrate, such as charge transfer or the formation of surface complexes. These processes alter molecular polarizability and increase Raman scattering efficiency. While weaker than EM effects, CM enhancement can dominate for analytes that directly bind or interact strongly with the metal surface.

### 2.3 Hotspots: Open vs Gapped

A defining feature of SERS is the presence of nanoscale electromagnetic “hotspots,” where local field enhancement is maximal. Hotspots occur at sharp tips, edges, or junctions of plasmonic nanostructures and are commonly classified as open or gapped.^78^ Open hotspots form on individual NPs, whereas gapped hotspots arise in nanogaps (<10 nm) between closely spaced particles, where plasmonic coupling produces substantially stronger fields (Figure 1D).

For biological analytes, hotspot accessibility is strongly analyte size-dependent.^78,86^ Small biomolecules such as nucleotides or short oligonucleotides readily access nanogaps, maximizing enhancement, whereas larger targets such as viral proteins (∼10–20 nm) and intact virions (30–300 nm)^87,88^ are geometrically excluded.^78,89^ As a result, viruses primarily interact with more exposed, open hotspots that provide weaker but more accessible enhancement. This size mismatch affects sensitivity, reproducibility, and spectral completeness and represents a fundamental constraint in SERS-based virus detection. Numerical simulations further reveal that the effective hotspot location shifts with analyte size, as shown in Figure 1D–F.^78^ For small analytes, the strongest enhancement occurs near the center of nanogaps (Figure 1E), whereas for larger analytes the highest local fields relocate to contact regions between the analyte and NPs (Figure 1F). As a result, both enhancement magnitude and spectral response depend on analyte size and orientation, potentially influencing observed Raman spectral shapes.

In addition, electromagnetic enhancement exhibits a strong distance dependence and decays rapidly as the target molecule moves away from the metal surface.^90^ Experimental studies confirm that even nanometer-scale separations can reduce enhancement by several orders of magnitude.^91^ Thus, analyte position relative to plasmonic hotspots is a decisive factor governing SERS signal intensity and reproducibility.

### 2.4 SERS Substrates

The design of the SERS substrate is a primary determinant of detection performance, governing the signal intensity, specificity, reproducibility, and practical usability. For viral diagnostics, an ideal substrate must deliver strong enhancement and low detection limits while selectively amplifying target signals over complex biological backgrounds. It should be compatible with the intended detection format, maintain stable and uniform performance across batches and environmental conditions, and exhibit biocompatibility to preserve viral integrity and minimize nonspecific adsorption. For clinical and POC deployment, scalability, cost-effectiveness, and manufacturing reliability are equally critical. Importantly, substrate fabrication is not merely a materials-processing step; it directly determines the hotspot density, analyte accessibility, surface chemistry, signal reproducibility, and compatibility with downstream diagnostic workflows. Together, these considerations define the key benchmarks for substrate design.^92,93^

To meet these requirements, SERS substrates for virus detection are commonly classified into colloidal, thin-film, and hybrid systems (Figure 2). Colloidal substrates are typically prepared by wet-chemical reduction of metal salts,^94^ seed-mediated growth,^95^ galvanic replacement,^96^ ligand-assisted shape control, or core–shell growth.^97,98^ These solution-based methods enable the synthesis of Ag or Au nanospheres, nanorods, nanostars, nanocubes, and bimetallic/core–shell particles with tunable plasmonic properties. They are attractive because they are low-cost, scalable in solution, and readily functionalized with antibodies, aptamers, nucleic acids, or Raman reporters. However, their performance is highly sensitive to reducing agents, capping ligands, ionic strength, temperature, purification steps, and storage conditions, all of which can alter particle size, surface chemistry, aggregation behavior, and SERS reproducibility.^99^

**Figure 2:**
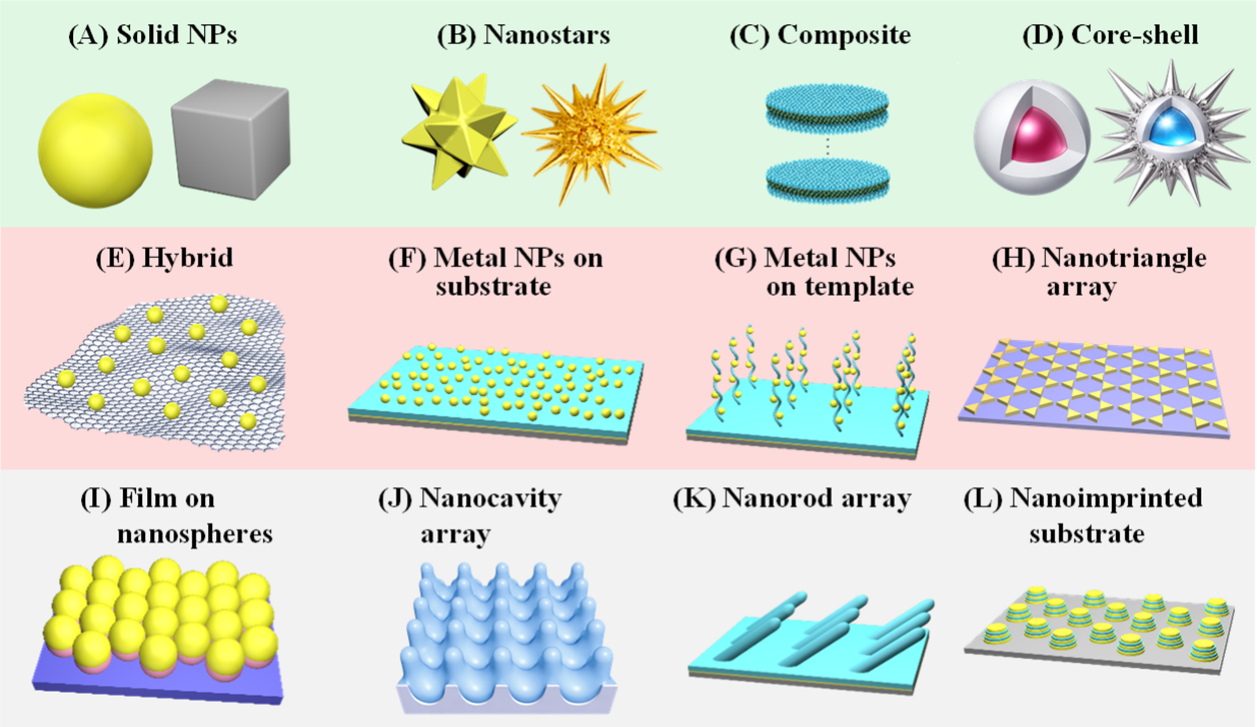
Overview of common SERS substrate configurations employed in viral detection assays.

Plasmonic colloidal substrates, typically based on Ag or Au NPs with sizes of 30–120 nm (Figure 2A,B), offer high surface area and versatility for detecting intact viruses, proteins, nucleic acids, and antibodies.^100–108^ Their high enhancement often arises from interparticle nanogaps formed during controlled aggregation or target-induced assembly. Nevertheless, uncontrolled aggregation, ligand desorption, and colloidal instability can lead to poor quantitative reproducibility. Composite (Figure 2C) and core–shell colloids (Figure 2D) (e.g., Ag–Au, Au–Cu, MXenes, MoO_3_ quantum dots, ∼ 100–1000 nm) are commonly fabricated through sequential deposition, shell growth, heterostructure assembly, or surface coating strategies. These designs improve chemical stability, magnetic enrichment, bioconjugation capacity, or multifunctionality, but they also introduce greater synthetic complexity and cost.^43,107,109,110^ Hybrid colloidal substrates (Figure 2E), such as NPs supported on carbon, polymer, oxide, or other matrix materials, are usually prepared by adsorption, in situ growth, or matrix-assisted assembly. These platforms combine plasmonic enhancement with improved handling or enrichment capability, but require careful control of nanoparticle loading, spatial distribution, and surface accessibility.^111,112^

Thin-film substrates are generally fabricated by substrate-supported assembly, template-assisted growth, physical vapor deposition, or lithographic patterning.^113^ These include NP films (Figure 2F,G),^42,44,114–119^ nanopatterns (Figure 2H,I),^41,120–123^ nanocavity arrays (Figure 2J),^124–127^ nanorod arrays (Figure 2K),^128–136^ and imprinted structures (Figure 2L).^137^ Compared with colloidal suspensions, thin-film substrates offer superior mechanical stability, batch-to-batch reproducibility, and device compatibility. NP films can be produced by drop-casting, spin-coating, Langmuir–Blodgett assembly, electrophoretic deposition, or layer-by-layer assembly. In these approaches, drying dynamics, surface functionalization, and particle packing determine interparticle spacing and hotspot uniformity. Lithographic nanopatterns and nanocavity arrays can be fabricated using electron-beam lithography, focused-ion-beam milling, nanosphere lithography, anodic aluminum oxide templates, or other template-assisted methods. These approaches provide precise control over geometry and periodicity, improving reproducibility, but often require specialized equipment and higher fabrication costs. Nanorod arrays can be produced by oblique-angle deposition, which generates dense tilted metallic nanostructures with abundant nanoscale junctions and strong electromagnetic enhancement. Nanoimprinted substrates, fabricated using reusable molds and polymer/metal replication processes, are particularly attractive for scalable manufacturing because predefined nanostructures can be transferred over large areas with improved batch consistency. Although the enhancement factors of thin-film substrates are often lower than those of optimized nanogap colloids, their reproducibility, stability, and device compatibility make them especially attractive for portable and integrated diagnostic platforms.

Overall, substrate selection requires balancing the sensitivity, reproducibility, cost, scalability, and system integration. Colloidal and composite systems remain valuable for high-sensitivity laboratory studies and flexible bioconjugation, whereas thin-film, template-assisted, and nanoimprinted substrates are better suited for standardized manufacturing and scalable POC diagnostics. Thus, future substrate development for SERS-based viral diagnostics should consider not only enhancement performance but also fabrication reproducibility, surface functionalization, clinical-sample compatibility, and integration into automated diagnostic workflows.

## 3 SERS-Based Detection Strategies

SERS-based virus detection integrates three core elements: the plasmonic substrate, the viral or host-derived analyte within its biological environment, and the Raman readout system. The central challenge is to convert analyte–substrate interactions into robust and interpretable Raman signals. Depending on whether intrinsic molecular vibrations or extrinsic reporters are measured, SERS strategies are broadly classified as label-free (direct or affinity-based) or labeled (tag-based) detection. Each approach involves distinct trade-offs in sensitivity, specificity, multiplexing capability, assay complexity, and suitability for POC deployment. A critical factor in strategy selection is the nature of the target analyte, as viruses present diagnostically relevant features spanning nucleic acids, proteins, intact virions, and host-response biomarkers. These targets differ markedly in molecular composition, size, abundance, and accessibility in clinical specimens. Table S1 in the Supporting Information (SI) summarizes viruses reported in the SERS literature together with their biological classifications and physical properties, providing context for analyte selection.

### 3.1 Biological Characteristics of Viruses Relevant to SERS Detection

Viruses are structurally simple but biologically diverse entities, and their physicochemical characteristics strongly influence how they can be detected by SERS. In general, a virus consists of a nucleic acid genome, either RNA or DNA, enclosed within a protein capsid; some viruses also possess a lipid envelope derived from the host cell membrane.^138^ Enveloped viruses, such as influenza viruses, HIV, and SARS-CoV-2, contain membrane-associated proteins or glycoprotein spikes that mediate host cell attachment and entry. These surface proteins, such as influenza hemagglutinin and neuraminidase or the SARS-CoV-2 spike protein, are often attractive targets for affinity-based detection because they are externally accessible. However, the lipid envelope can also make these viruses more sensitive to environmental conditions and sample handling procedures. In contrast, nonenveloped viruses, such as adenoviruses, rhinoviruses, noroviruses, and rotaviruses, lack a lipid envelope and are often more environmentally stable, but their outer capsid proteins provide different constraints for selective recognition and SERS hotspot access.^139–144^

Several viral properties are particularly relevant to SERS assay design (see Table S1). First, virus size, morphology, and surface composition determine how efficiently intact virions interact with plasmonic substrates, enter electromagnetic hotspots, or bind to capture ligands. Typical viral dimensions range from tens to hundreds of nanometers, much larger than many plasmonic nanogaps; therefore, intact-virus SERS often probes only the outermost viral components in contact with accessible hotspots. Second, genome type and abundance influence whether viral RNA or DNA can be detected directly after extraction or through amplification-assisted SERS strategies. Third, viral surface chemistry controls nonspecific adsorption, affinity capture, and matrix interference in clinical specimens. Therefore, the same virus may be approached through multiple SERS-detectable targets, including intact virions, viral RNA/DNA, capsid proteins, envelope proteins, glycoprotein spikes, or virus-induced host biomarkers.

Viral infection is also assessed through host-derived targets. Antibodies such as immunoglobulin M (IgM), immunoglobulin A (IgA), and immunoglobulin G (IgG) reflect immune response rather than direct viral presence,^145^ while cytokines,^146^ microRNAs (miRNAs), exosomes, metabolites, and volatile organic compounds can provide complementary information about disease state or severity.^147^ These targets differ in their diagnostic timing, specificity, specimen availability, and clinical interpretation. Thus, SERS target selection should be matched to the biological question being asked: active infection, viral load, strain or variant identification, immune exposure, disease severity, treatment response, or public health surveillance.^148^ Understanding these biological characteristics provides the foundation for why direct, affinity-based, and labeled SERS strategies have been developed for different viral targets and diagnostic applications.

### 3.2 Possible Analytes for Virus Detection

SERS-based virus diagnostics can target whole virions, viral components, or host-derived biomarkers, with the choice guided by the infection stage, diagnostic objective, and specimen type. For acute infections, viral nucleic acids or antigens are often preferred, whereas antibodies and host biomarkers are more relevant for assessing the exposure history or disease severity. Key selection criteria include sensitivity, specificity, reproducibility, and practical considerations, such as specimen accessibility and reagent availability.

The major analyte classes include: (1) Nucleic acids, typically viral RNA or DNA, often combined with amplification strategies such as PCR.^149^ (2) Viral antigens, including surface or internal proteins (e.g., SARS-CoV-2 spike, influenza hemagglutinin, nucleoproteins), commonly detected using antibody- or aptamer-functionalized substrates.^115,150^ (3) Host antibodies (IgM, IgG, IgA), which indicate immune response but do not directly distinguish active from past infection.^151^ (4) Host or virus-associated biomarkers, encompassing viral proteins (e.g., HIV p24),^152,153^ cytokines and immune mediators (e.g., IL-6, TNF-α),^154^ cellular or metabolic changes,^154–157^ and volatile organic compounds (VOCs) associated with infection-induced metabolic alterations.^108,158–160^ Table 1 summarizes these analytes, their diagnostic relevance, and specimen requirements.

**Table 1:** Target Analytes for SERS-Based Virus Detection

	Analyte Class	Diagnostic Relevance	Typical specimen
Virus	Viral nucleic acids (RNA/DNA)	High sensitivity and specificity; enable variant-specific or pan-viral detection; often combined with amplification	Swab, saliva, blood
	Viral structural proteins	Enable antigen-based detection of intact viruses; surface proteins support direct capture	Swab, saliva, aerosols
	Viral nonstructural proteins	Intracellular targets; limited diagnostic use but relevant for mechanistic or drug studies	Cell lysate (enriched)
	Intact viruses	Preserve full structural and molecular information; enable direct detection with minimal preprocessing	Saliva, swab, breath, wastewater
Host	Host antibodies (IgM, IgG, IgA)	Indicate immune response and exposure history; not specific to active infection	Serum, plasma, saliva
	Host nucleic acids (miRNAs)	Reflect infection-induced gene regulation; enable indirect but sensitive detection	Blood-derived fluids
	Exosomes/extracellular vesicles	Carry viral RNA/proteins and immune signals; emerging diagnostic targets	Blood, saliva, nasal fluid
	Host biomarkers	Indicate disease severity and host response; complementary to viral detection	Blood, saliva
	Breath-borne VOCs	Noninvasive signatures of infection-related metabolic changes; suitable for AI-assisted screening	Exhaled breath

The diagnostic value of each analyte depends strongly on disease progression.^161–163^ In general, intact virions, viral RNA/DNA, and viral proteins are most useful for detecting active infection during the early and acute stages, whereas host antibodies and immune-response biomarkers become more informative later or for assessing exposure history, disease severity, or treatment response. Viral nucleic acids provide high specificity and can support variant-level detection but often require extraction, amplification, or enrichment. Viral proteins and intact virions are attractive for rapid antigen-style or direct SERS detection, but their detectability depends on abundance, epitope accessibility, matrix interference, and hotspot access. Host antibodies such as IgM, IgA, and IgG expand the diagnostic window but appear after a delay and may not distinguish between active and past infection. Host-response biomarkers, including cytokines, miRNAs, exosomes, metabolites, and VOCs, can provide complementary information about the disease state but are usually less virus-specific. Therefore, SERS target selection should be matched to the intended diagnostic window, specimen type, and clinical decision being supported. Figure 3 summarizes the approximate time-dependent availability, advantages, and limitations of the major SERS-detectable viral and host targets.

**Figure 3:**
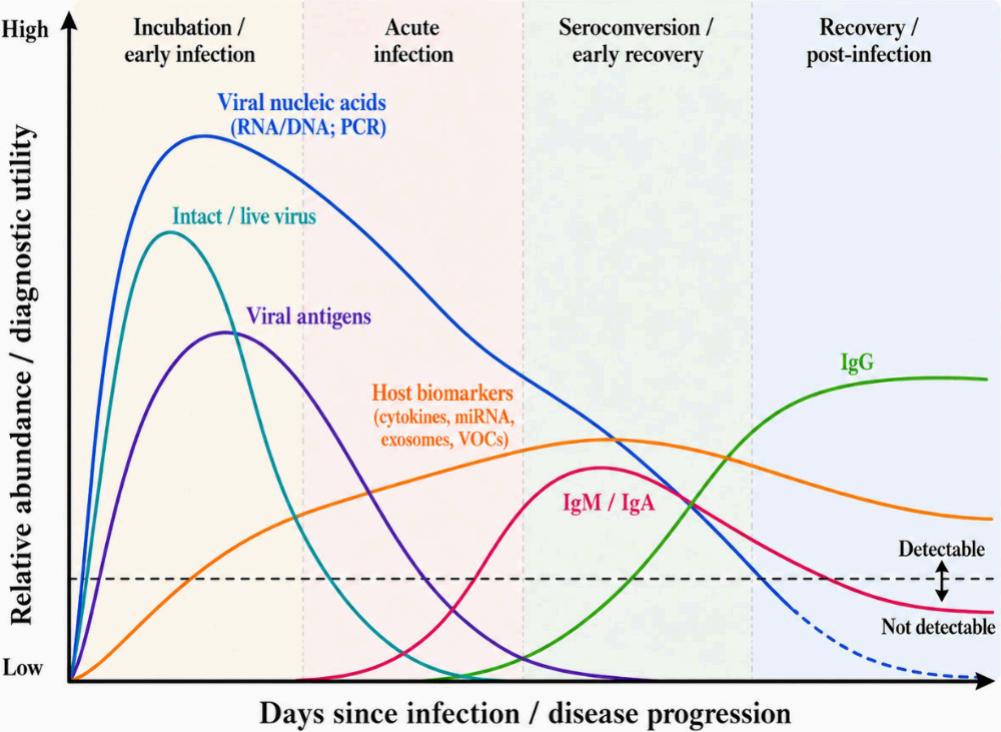
Approximate time-dependent availability and diagnostic utility of viral and host-derived targets during viral infection. Exact timing depends on the virus, specimen, and assay.

### 3.3 SERS-Based Virus Detection Strategies

SERS-based virus-detection strategies are generally divided into label-free and labeled approaches (Figure 4). Label-free methods rely on intrinsic Raman signatures or binding-induced spectral changes, whereas labeled approaches use engineered SERS tags to amplify and standardize the signals.

**Figure 4:**
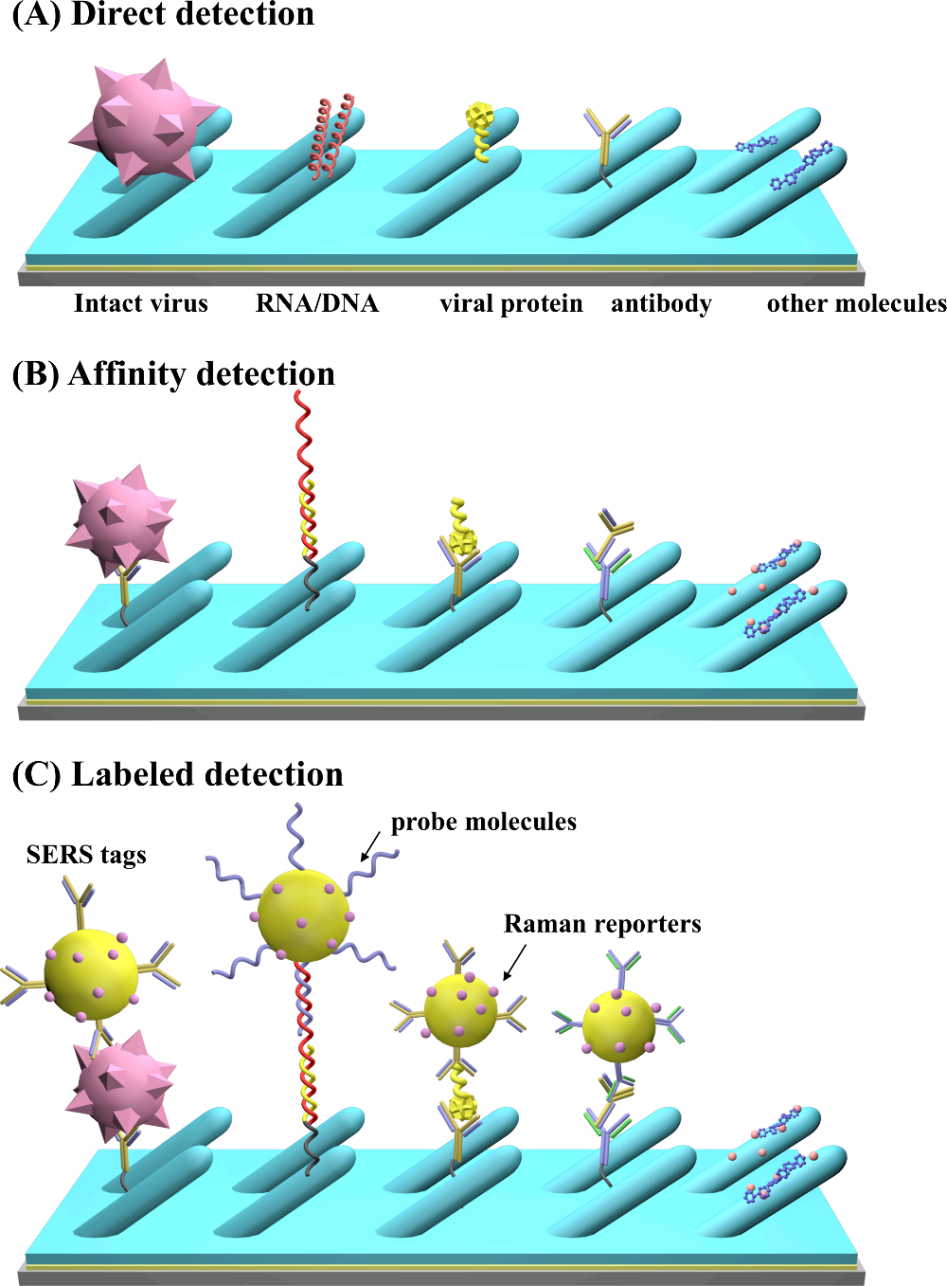
General SERS detection strategies for viral and host-derived analytes. Label-free detection: (A) direct detection and (B) affinity-based detection. (C) Labeled detection.

Direct label-free detection involves the adsorption of viral analytes such as whole virions, nucleic acids, or proteins directly onto SERS substrates (Figure 4A). The resulting vibrational fingerprints enable classification or quantification with minimal sample preparation and no chemical labeling. While this approach preserves analyte integrity and enables rapid measurements, its performance is often limited by weak scattering, restricted hotspot access for large analytes, and spectral interference from complex biological matrices.

Affinity-based label-free detection improves specificity by functionalizing substrates with capture molecules, such as antibodies, aptamers, or viral receptors (Figure 4B). Selective binding enriches the target at plasmonic hotspots and induces measurable spectral changes. This strategy enhances selectivity but introduces additional surface chemistry steps and may alter analyte conformation or binding behavior.

Labeled detection employs SERS tags, i.e., NPs that integrate a plasmonic core, a Raman reporter, a recognition ligand, and a stabilizing layer, to achieve strong signal amplification and multiplexing (Figure 4C). Upon target binding, tags form sandwich-type assemblies with capture substrates, producing intense and reproducible signals. This approach offers the highest sensitivity and supports the simultaneous detection of multiple targets but requires careful nanoprobe synthesis, reporter selection, and quality control. Increased assay complexity, cost, and potential spectral crosstalk must be addressed for reliable quantitative performance.

Importantly, these strategies should be viewed as complementary, rather than hierarchical. Direct detection prioritizes simplicity and speed, affinity-based approaches balance specificity and robustness, and labeled strategies maximize sensitivity and multiplexing capabilities. Optimal strategy selection therefore depends on aligning the physicochemical properties of the analyte (e.g., size, abundance, and molecular composition) with practical constraints, such as specimen type, required detection limits, reproducibility, and deployment environment. Effective SERS-based viral diagnostics must balance analytical performance against considerations of cost, robustness, and scalability, particularly for clinical and POC applications.

## 4 Direct Detection

Direct SERS detection aims to exploit the intrinsic vibrational fingerprints of viruses and their components or biomarkers without requiring labels or amplification agents. In principle, these spectra enable virus identification, strain discrimination, and concentration estimation. In practice, performance is governed by substrate design, analyte size and surface accessibility, and the ability to distinguish viral signals from complex biological backgrounds.

### 4.1 Direct Detection Configurations

Three common configurations have been employed for direct virus detection using SERS (Figure 5). Thin-film substrates are the most widely used platforms, offering superior reproducibility, scalability, and compatibility with portable Raman systems.^36,41,46,118,164,165^ Ag nanorod (AgNR) arrays fabricated by oblique angle deposition generate dense hotspots at nanorod tips and inter-rod gaps and have enabled detection of respiratory syncytial virus (RSV), influenza, HIV, and coronaviruses, with reported limits of detection down to ∼ 10 PFU/mL in buffered media.^93^ Nanocavity and nanocone arrays further improve signal consistency by geometrically matching cavity dimensions to virion size, enhancing particle trapping and hotspot access.^125,166^ Lithographically patterned or templated thin films provide uniform hotspot distributions and are readily integrated into device-based and point-of-care platforms.^167^ These substrates represent the current benchmark for direct, label-free SERS virus detection.

**Figure 5:**
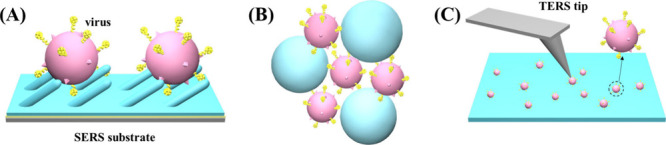
Representative configurations for direct SERS-based virus detection using (A) thin-film substrates, (B) colloidal NPs, and (C) TERS.

Colloidal NP systems, typically based on Au or Ag NPs, offer low-cost fabrication and experimental simplicity.^168–171^ Mixing viral particles with NP suspensions can induce aggregation around the viral surface, generating transient plasmonic hotspots. This approach has been applied to a wide range of viruses, including influenza,^168,169,172^ adenovirus,^172^ SARS-CoV-2,^168,172,173^ human papillomavirus (HPV),^174^ and HIV. However, uncontrolled aggregation, sensitivity to matrix effects, and poor batch-to-batch reproducibility limit the reliability and hinder translational use.

Tip-enhanced Raman spectroscopy (TERS) combines scanning probe microscopy with localized plasmonic enhancement to achieve nanometer-scale spatial resolution.^175^ TERS has enabled detailed mapping of individual viral particles, including tobacco mosaic virus,^176^ avipoxvirus,^177^ and adeno-associated virus,^177^ revealing spatial distributions of proteins and nucleic acids within single virions. While uniquely powerful for mechanistic studies, its technical complexity, low throughput, and specialized instrumentation restrict TERS to fundamental research applications, rather than clinical diagnostics.

### 4.2 Direct Intact Virus Detection

Direct SERS detection of intact viruses was first demonstrated by Shanmukh et al. for multiple respiratory viruses including adenovirus (AdV), rhinovirus (RV), HIV, RSV, and influenza A (IAV).^36^ As shown in Figure 6A, the spectra are dominated by broad features at higher wavenumbers (Δν > 1100 cm^–1^), with substantial overlap among viruses, including common peaks at 659, 732, 855, 1004, 1032, 1449, and 1593 cm^–1^ (AdV, RV, and HIV). Despite this overlap, virus-specific features enable differentiation: AdV exhibits bands at 907 and 1657 cm^–1^, RV at 958 and 1244 cm^–1^, HIV at 1128 and 1375 cm^–1^, and RSV at 767 and 836 cm^–1^. These results established that intrinsic SERS fingerprints can distinguish viral species. Subsequent studies further demonstrated strain-level discrimination, with subtle but reproducible spectral differences observed among RSV and IAV variants (Figure 6B),^178^ supported by chemometric analyses that achieved clear strain separation.^41,46,166,169,173,179,180^ SERS has also been shown to probe viral structural integrity. Sitjar et al. reported that SARS-CoV-2 pseudoviruses retained characteristic spectral features after mild heat treatment (65 °C) or UV exposure, whereas autoclaving resulted in complete spectral loss.^124^ Similar behavior was observed for adenovirus and SARS-CoV-2 by Zhang et al.,^168^ indicating that SERS signals persist unless viral biomolecular structure is severely denatured.

**Figure 6:**
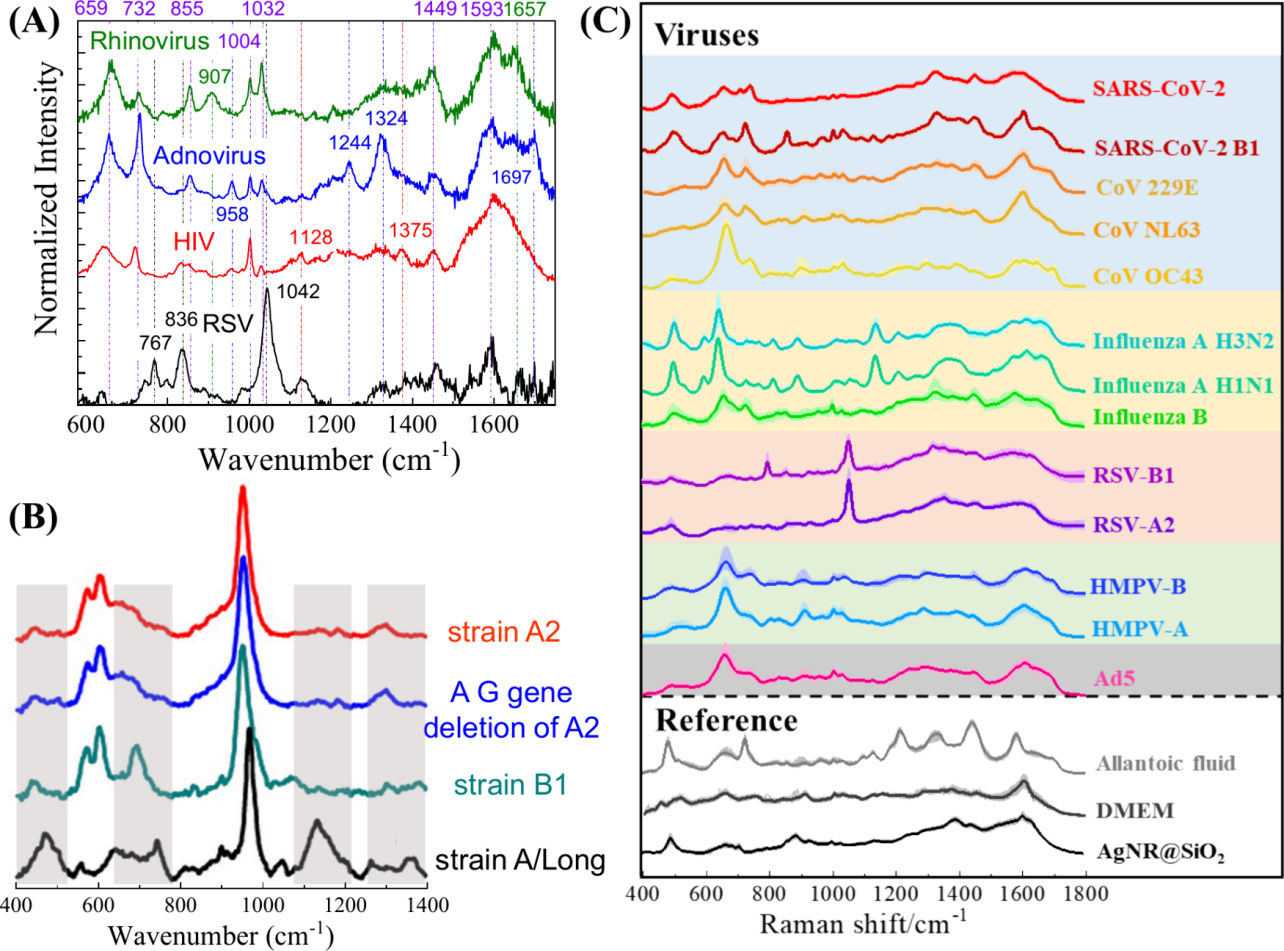
(A) Representative SERS spectra of RV, AdV, HIV, and RSV; Adapted with permission from ref 36. Copyright 2006, American Chemical Society. (B) SERS spectra of four RSV strains; Adapted with permission from ref 178. Copyright 2008, Springer Nature. (C) Normalized SERS spectra of 13 viruses along with 3 reference controls; Reproduced with permission from ref 40. Copyright 2022, Elsevier.

A broader study encompassing 13 respiratory viruses confirmed that intact-virus SERS spectra are predominantly governed by proteins and nucleic acids.^40^ Average spectra (Figure 6C) revealed both shared and virus-specific features. For example, SARS-CoV-2 exhibited characteristic bands at 656 cm^–1^ (guanine), 726 and 1330 cm^–1^ (adenine), and 1003/1031 cm^–1^ (phenylalanine), along with an amide I shift at 1660 cm^–1^ distinguishing it from the B.1 variant. Common coronaviruses showed prominent peaks at 658, 725, 1004, 1034, 1329, 1450, and 1603 cm^–1^, while SARS-CoV-2 and its variant exhibited an additional tyrosine band at 858 cm^–1^. Across all viruses examined, the most consistent peaks included 1003 cm^–1^ (phenylalanine) and 1576 cm^–1^ (tryptophan ν_a_COO^–^), confirming that SERS spectra of intact viruses primarily reflect vibrational modes of structural proteins and nucleic acid components.

#### 4.2.1 Understanding Viral Spectra

Systematic analyses of intact-virus SERS spectra consistently reveal both shared vibrational features and virus-specific signatures. Across multiple studies, the number of reported characteristic peaks ranges from 15 (seven viruses)^45^ to 28 (13 viruses),^40^ with more extensive assignments listing up to 79–88 peaks.^118,125^ Despite differences in reporting depth, these studies converge on three core conclusions. First, substantial spectral overlaps are unavoidable, reflecting the common biochemical composition of viruses. Second, viral proteins and nucleic acids dominate the SERS response, with recurring contributions from aromatic amino acids (phenylalanine, tryptophan, tyrosine), nucleobases (guanine, adenine, uracil), and backbone modes (amide I/III, phosphate vibrations). Third, subtle but reproducible spectral differences encode structural and compositional variations among viruses.

These trends can be rationalized by modeling the virus–substrate interaction as a layered structure in contact with a plasmonic hotspot (Figure 7A). Upon excitation at the substrate’s LSPR, electromagnetic enhancement is highly confined within a few nanometers of the metal surface. Consequently, the SERS spectrum is well approximated as a weighted linear combination of viral components, where weights depend on molecular abundance and proximity to the hotspot.^78^ For enveloped viruses, surface glycoproteins dominate the response, followed by the lipid envelope, matrix proteins, and the internal nucleocapsid. Larger viruses therefore exhibit spectra dominated by outer structural proteins, whereas smaller or more compact viruses may show more balanced contributions from internal components.

**Figure 7:**
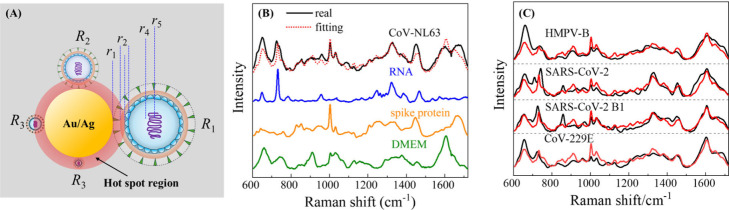
(A) Illustration of a viral particle adsorbing onto a plasmonic NP and the corresponding virus size effect. (B) Experimental SERS spectrum of CoV-NL63 along with spectra of DMEM buffer, spike protein, and RNA. The red dotted curve represents the best-fit linear combination of the spike protein, RNA, and DMEM spectra. (C) Comparison of measured virus spectra (black) and fitted spectra (red) obtained from linear-combination modeling for four additional viruses measured in different background media. Panels A–C are adapted with permission from ref 78. Copyright 2024, Royal Society of Chemistry under CC BY-NC 3.0 https://creativecommons.org/licenses/by-nc/3.0/.

In practical measurements, intact viruses are suspended in complex matrices containing abundant background molecules that readily compete for hotspot access. As a result, experimentally acquired spectra reflect superimposed viral and background signals. Decomposition studies demonstrated that intact-virus spectra can be accurately reconstructed from linear combinations of growth media, viral proteins, and viral RNA (e.g., Pearson correlation coefficient *C*_*P*_ ≈ 0.85 for human coronavirus NL63 (CoV-NL63), Figure 7B).^40^ Quantitative analysis across multiple coronaviruses including human coronavirus 229E (CoV-229E), SARS-CoV-2 (original and B.1 variant), and human metapneumovirus subtype B (HMPV-B) (Figure 7C) revealed that background contributions were consistently largest, followed by surface proteins and RNA. Notably, viral RNA often contributed comparably to surface proteins despite its internal localization, suggesting that distance-based enhancement models may underestimate interior contributions, particularly when viruses deform, partially rupture, or allow flexible RNA strands to extend into the enhancement region.

#### 4.2.2 Interference from Background Medium

In practical settings, viruses are rarely measured in an isolated setting. Laboratory samples often contain residual culture media or cell lysates, while clinical specimens originate from complex biological fluids, such as saliva, nasal secretions, serum, or urine. These matrices introduce strong background Raman signals that can mask or distort virus-specific features in direct SERS detection (Figure 7B,C). Early studies demonstrated that viral signals can be recovered in mixed environments under favorable conditions. Shanmukh et al. showed that RSV-infected lysates retained characteristic viral peaks absent in uninfected controls,^36^ and Fan et al. reported that virus-specific features become distinguishable once analyte concentration exceeds a threshold.^45^ However, clinical specimens present greater challenges. For example, direct SERS spectra of SARS-CoV-2 in saliva often closely resemble saliva alone, hindering reliable peak assignment.^137,165,173^ Zhang et al. similarly observed only minor spectral differences between SARS-CoV-2 measured in saliva and serum, underscoring the dominance of background contributions.^168^

The detectability of viral signals is highly concentration-dependent. Yang et al. observed that at high concentrations (25,000 PFU/mL), spectra acquired in buffer and saliva were nearly indistinguishable, whereas at lower concentrations (1562 PFU/mL), medium-specific effects became pronounced and spectral differences increased.^40^ Biological variability further complicates interpretation: saliva and nasal swab samples from different individuals exhibit distinct protein-related bands (e.g., 724, 1325, 1452, 1585 cm^–1^), reflecting interpatient heterogeneity.^165^

#### 4.2.3 Performance of Direct Viral Detection

The performance of direct SERS detection of intact viruses can be evaluated along four key dimensions: specificity; sensitivity and limit of detection (LOD); quantification capability; and response time. Representative performance metrics reported in the literature are summarized in Table S2 of the **SI**.

##### 4.2.3.1 Specificity

Direct SERS detection achieves specificity by exploiting virus-dependent vibrational fingerprints. As discussed in Section 4.2.1, while certain bands such as 1003 cm^–1^ (phenylalanine) and 1576 cm^–1^ (tryptophan ν_a_COO^–^) are common across many viruses, distinct peak combinations enable discrimination at the species and strain levels.^40^ Reliable specificity depends critically on the availability of well-curated reference spectral libraries that include viral variants. Once established, unknown samples can be classified using three complementary approaches: (1) Pattern recognition, in which diagnostic peak combinations are identified visually or algorithmically;^36,41,178^ (2) chemometric methods, including PCA, hierarchical cluster analysis (HCA), PLS, PLS-DA, and principal component analysis–linear discriminant analysis (PCA-LDA), which resolve subtle spectral differences in complex data sets;^45–47,117,118,120,128,137,170^ and (3) ML models such as support vector machines (SVM), random forests (RF), and neural networks (NN), which are increasingly used for high-dimensional spectral classification^41,137^ (see Section 4.2.4 for more).

##### 4.2.3.2 Sensitivity, LOD, and Quantification

For many SERS-based viral diagnostic assays, particularly point-of-care screening tests, the primary clinical decision is binary: whether the sample is positive or negative. In such cases, LOD, diagnostic sensitivity, specificity, and the clinically meaningful decision threshold are more important than exact quantification of viral concentration. Quantitative analysis is nevertheless valuable when viral load informs clinical or public-health decisions, such as monitoring chronic infections, assessing infection stage or infectivity, evaluating treatment response, estimating coinfection burden, or comparing assay performance across platforms. In SERS measurements, both detection and quantification depend on the ability to isolate virus-specific spectral features from background contributions, which is strongly influenced by viral concentration and sample matrix (Section 4.2.2). Calibration studies remain limited but demonstrate feasibility: Shanmukh et al. reported near-linear responses for RSV between 100 and 2000 PFU/mL,^36^ and Yadav et al. quantified HIV-1 from 200 to 5 × 10^7^ copies/mL.^180^ Spectral preprocessing, particularly baseline removal, plays a critical role in establishing reliable calibration curves. Reported LODs (3σ criterion) vary with substrate efficiency, reaching ∼ 10 PFU/mL for SARS-CoV-2 on optimized substrates,^164^ typically 10^2^–10^3^ PFU/mL in many studies, and up to 10^3^–10^4^ PFU/mL for lower-performance substrates (Table S2). Multivariate regression approaches, such as PLS, generally outperform single-peak analysis for concentration prediction; for example, rotavirus concentrations up to 10^6^ PFU/mL were successfully predicted using PLS regression.^46^ More advanced ML-based regression models offer even greater potential (Section 4.2.4).

##### 4.2.3.3 Response Time

Total assay time comprises sample collection, preparation, virus adsorption, spectral acquisition, and data analysis. While spectral acquisition and automated classification typically require response times of 1–2 min, overall response time is dominated by sample preparation and virus adsorption (Section 7). Adsorption kinetics depend on viral size, surface charge, and morphology, as well as substrate properties and environmental conditions such as pH and ionic strength.^181–189^ Electrostatic interactions, van der Waals forces, hydrophobic effects, and specific binding all contribute.^190–193^ Quartz crystal microbalance with dissipation monitoring (QCM-D) and related studies indicate adsorption times ranging from 10 to 60 min, consistent with reported SERS incubation periods of 30–120 min, although faster or multiphase adsorption behaviors have been observed for certain substrates.^182,188,189,193–195^

#### 4.2.4 Integration of Direct SERS Detection with Machine Learning

Discriminating viruses using SERS spectra is challenging due to high dimensionality, overlapping features, and nonlinear relationships between spectral features and biological variables. Traditional chemometric methods, while useful, are often limited in their ability to resolve complex patterns and generalize across virus types, strains, and experimental conditions. For instance, PCA applied to SERS spectra from 13 viruses at 10^5^ PFU/mL showed substantial cluster overlap, limiting the classification accuracy (Figure 8A). ML approaches address these limitations by modeling nonlinear relationships, automatically extracting informative features, and improving robustness to matrix effects and experimental variability.^196–198^

**Figure 8:**
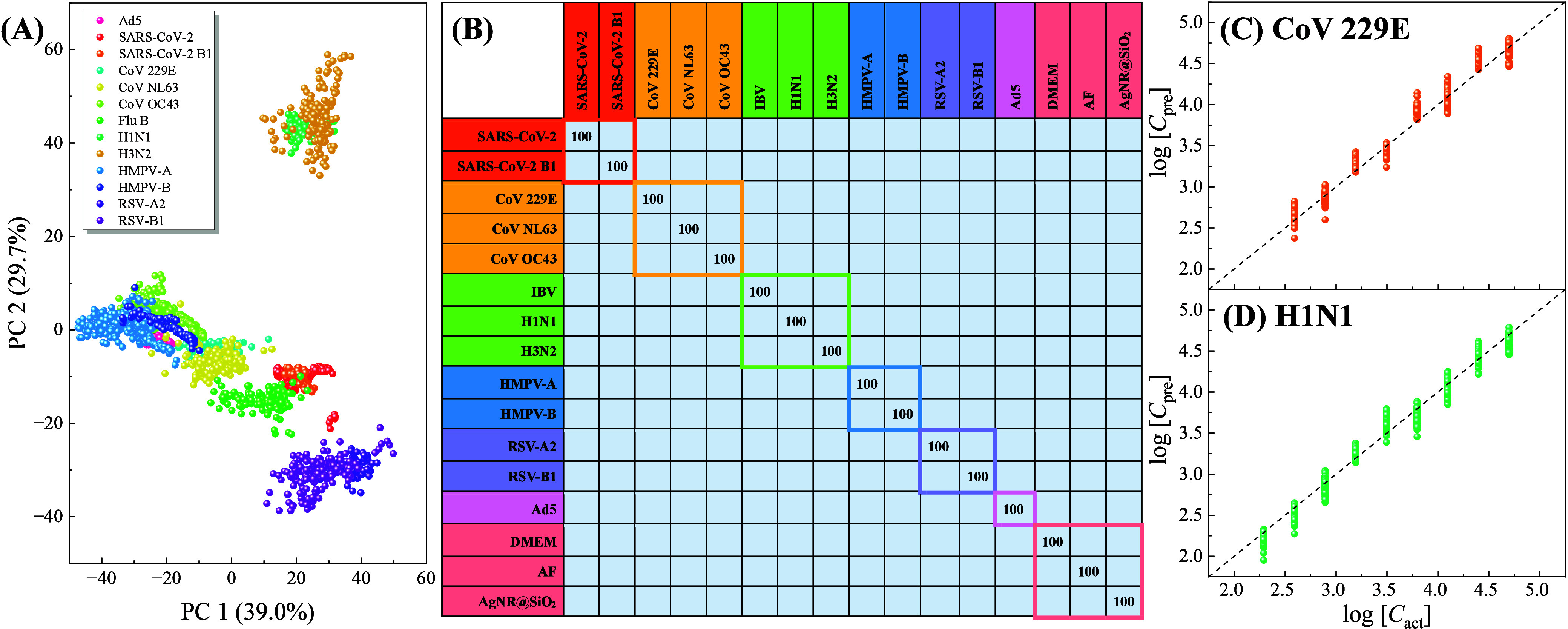
(A) PCA plot of 13 viruses based on their SERS spectra. (B) Classification results for 13 viruses using an SVM model. (C) and (D) Examples of virus concentration predictions using SVR. Panels B-D are reproduced with permission from ref 40. Copyright 2022, Elsevier.

Classical ML algorithms have been widely applied to SERS-based virus detection. SVMs and support vector regression (SVR) are particularly effective for nonlinear spectral data, achieving 100% accuracy in classifying 13 different viruses (Figure 8B) and enabling reliable concentration predictions (Figure 8C,D).^40^ SVMs have also been extensively applied for SARS-CoV-2 discrimination.^173^ RF models offer improved generalizability for noisy spectra,^199^ while simpler approaches such as k-nearest neighbors (KNN) and linear discriminant analysis (LDA) have been applied for classification and dimensionality reduction.^200,201^ Comparative studies consistently show that SVM-based models outperform RF, KNN, and LDA in multivirus classification tasks.^40^

With the growth of large, high-quality SERS data sets, deep learning algorithms (DLAs) have gained increasing importance due to their ability to learn hierarchical features directly from raw spectra.^202^ Convolutional neural networks (CNNs) are particularly effective for extracting localized spectral features and performing both classification and regression.^48^ For example, Ye et al. used a CNN-based platform to classify 14 human and avian viruses with >95% accuracy while identifying key protein-, nucleic acid-, and lipid-related Raman features (Figure 9A).^41^ As illustrated in Figure 9A, each SERS spectrum is treated as a one-dimensional input and passed through convolutional layers that progressively extract local peaks, peak combinations, and virus-specific spectral patterns. The final layer assigns the spectrum to a virus class. Importantly, the authors used a full-gradient-based interpretation method to identify the wavenumber regions that were most responsible for classification. These highlighted regions could be related to chemically meaningful Raman bands from proteins, lipids, nucleic acids, amide groups, phenylalanine, and tyrosine. Thus, Figure 9A shows both the CNN classification workflow and how interpretable deep learning can connect data-driven spectral features to viral biomolecular signatures.

**Figure 9:**
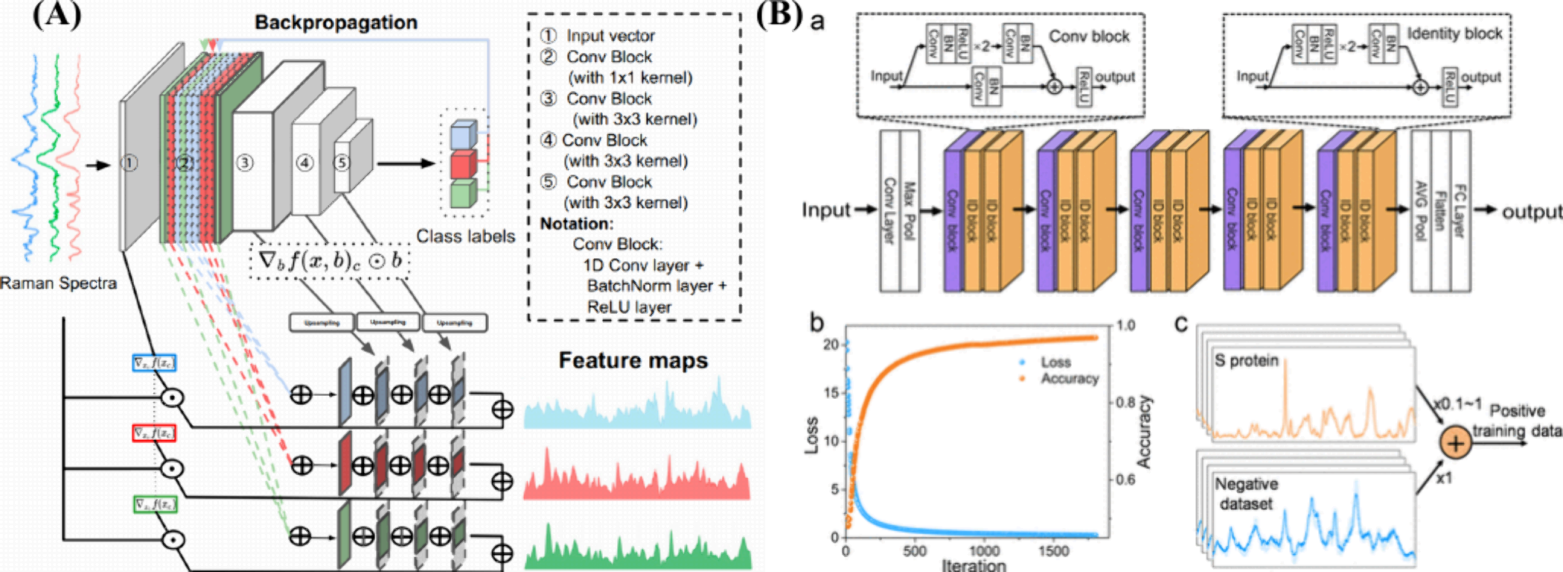
(A) CNN architecture used for SARS-CoV-2 classification; Reproduced with permission from ref 41. Copyright 2022, National Academy of Sciences under CC BY-NC-ND 4.0 https://creativecommons.org/licenses/by-nc-nd/4.0/. (B) RNN-based SERS analysis for SARS-CoV-2 diagnosis: (a) architecture of the RNN model; (b) training loss and accuracy with the dropout layer deactivated during evaluation; (c) generation of positive training data by combining negative SERS spectra with normal Raman spectra of the S protein; Reproduced with permission from ref 42. Copyright 2021, American Chemical Society.

Recurrent neural networks (RNNs) can capture contextual relationships in spectral data, in which neighboring Raman shifts collectively define molecular fingerprints.^203^ Huang et al. trained an RNN-based model using SARS-CoV-2 spike-protein spectra and negative clinical spectra.^42^ Figure 9B summarizes the full diagnostic workflow rather than only the neural-network architecture. First, spike protein Raman spectra and negative clinical SERS spectra were used to construct training data. Because positive clinical spectra were difficult to label at the single-spectrum level, synthetic positive spectra were generated by adding spike protein Raman features to negative clinical spectra. During testing, throat swab and sputum specimens were measured by Raman mapping, and individual spectral points were classified as positive or negative. The number of positive points was then used as a patient-level diagnostic score. The receiver operating characteristic (ROC) curve and confusion matrix summarize the clinical performance with 87.7% overall accuracy, 83.3% sensitivity, and 92.5% specificity for distinguishing COVID-19 patients from healthy controls. Therefore, Figure 9B illustrates an ML-assisted SERS diagnostic pipeline from spectral acquisition and training data generation to patient-level classification. Residual network (ResNet) architectures further improve performance by enabling deeper models;^204^ Yang et al. reported 98.5% test accuracy and 99.04% blind-test accuracy for COVID-19 detection using SFNet, along with prediction of reverse-transcription polymerase chain reaction (RT-PCR) cycle threshold (Ct) values with an RMSE of 1.627.^205^

Beyond single-virus detection, ML-enabled SERS platforms have demonstrated strong potential for multiplexed and coinfection analysis. The MultiplexCR framework analyzed over 1.2 million spectra from single-, two-, and three-virus mixtures in saliva (Figure 10).^206^ MultiplexCR is a multitask deep learning model that uses each SERS spectrum to predict both which viruses are present and their concentrations. As shown in Figure 10A, the input spectrum is processed by a series of one-dimensional convolutional blocks that extract spectral features from complex virus mixtures. Instead of producing only one classification label, the model generates two parallel outputs: a virus-presence vector and a virus-concentration vector. The virus-presence vector indicates whether each target virus is absent or present, whereas the concentration vector reports the predicted log_10_ (concentration) of each virus. For example, a single positive element in the presence vector corresponds to a single-virus sample, while multiple positive elements indicate a coinfection; the corresponding concentration vector provides the predicted viral load for each detected virus. Figure 10B,C summarize the model performance. The confusion matrices in Figure 10B show that MultiplexCR can distinguish single-virus samples, two-virus mixtures, three-virus mixtures, and saliva controls, while the regression plots in Figure 10C compare predicted and actual virus concentrations. The model achieved 98.6% classification accuracy for coinfections, a mean absolute error of 0.028 for concentration regression, and a rapid turnaround (∼15 min) in blind tests. Thus, Figure 10 illustrates how deep learning can convert complex SERS spectra from mixed viral samples into clinically useful outputs: pathogen identity and concentration. More broadly, this multitask strategy could be extended to larger respiratory-virus panels, viral variants, or other multiplexed infectious-disease applications.

**Figure 10:**
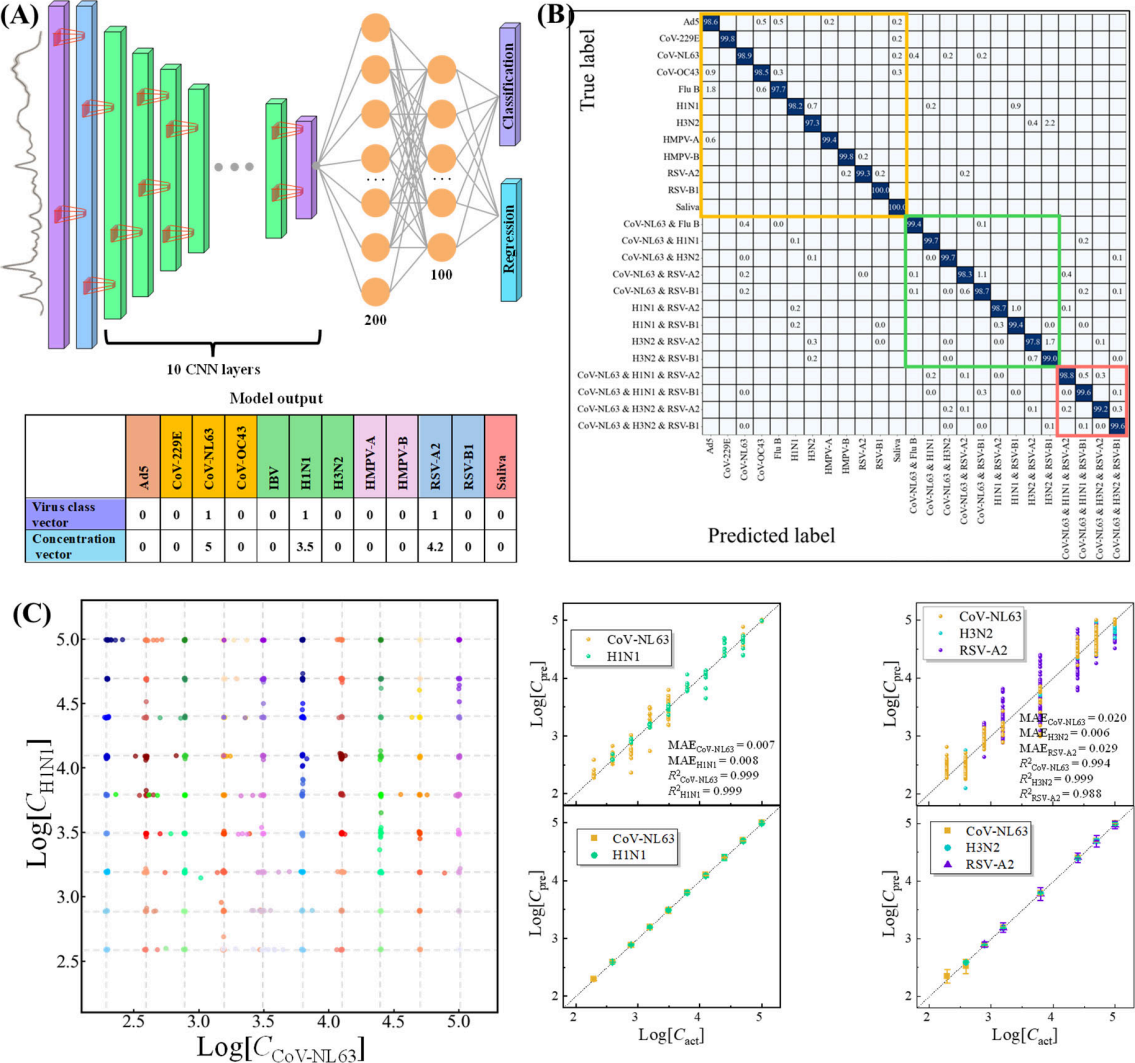
SERS-based deep learning for virus coinfection diagnosis in saliva. (A) Architecture of the MultiplexCR deep learning model and its dual 12-element vector outputs for multiclass classification and regression of virus mixtures. (B) Classification performance of MultiplexCR for coinfection detection, shown as confusion matrices for 11 single viruses (SVs), nine two-virus mixtures (2VMs), four three-virus mixtures (3VMs), and reference saliva. (C) Representative regression results generated by MultiplexCR for quantifying viral coinfections. Panels A-C are reproduced with permission from ref 206. Copyright 2025, American Chemical Society under CC-BY 4.0 https://creativecommons.org/licenses/by/4.0/.

Although ML has substantially improved SERS-based virus classification and quantification, rigorous methodological design is essential because viral SERS data sets are often small, heterogeneous, and strongly affected by substrate-, matrix-, and batch-dependent variability. Data augmentation can partially address limited data set size by introducing realistic spectral variations,^207–209^ such as low-level noise, intensity scaling, minor peak shifts, baseline changes, or synthetic spectral mixtures, but it should be applied only after training/test splitting to avoid data leakage. Feature extraction may rely on manually selected Raman peaks, peak ratios, peak areas, PCA/PLS scores, or automatically learned features from CNNs and other deep-learning models.^198,210^ Traditional feature-based models are often more interpretable, whereas deep-learning models can capture distributed spectral patterns but require larger data sets and stronger validation.^211^ Hyperparameters, including model complexity, regularization strength, learning rate, dropout rate, and the number of latent variables or convolutional filters, should be optimized by cross-validation within the training set. To reduce overfitting, spectra should be separated at the biological sample, patient, substrate, or batch level rather than by randomly splitting individual spectra. External validation, early stopping, regularization, class balancing, and interpretation methods such as feature-importance analysis or saliency mapping can further help ensure that ML models learn virus-related spectral information rather than artifacts from substrate heterogeneity, background media, or measurement batch effects.^198,210,212^

### 4.3 Direct Detection of Viral Components

In addition to intact virions, direct SERS analysis of individual viral components, particularly proteins and nucleic acids, provides a complementary and often more robust detection strategy. Isolated components exhibit well-defined vibrational signatures that enable selective identification of diagnostic biomarkers, particularly when viruses are degraded, chemically inactivated, or present at low concentrations. This component-resolved approach reduces geometric constraints associated with intact virions and facilitates the mechanistic interpretation of spectral features. Representative studies targeting viral proteins and RNA, along with corresponding substrates and performance metrics, are summarized in Table S3.

#### 4.3.1 Direct Detection of Virus Proteins

Viral proteins, especially surface-exposed spike (S) proteins and nucleocapsid (N) proteins, are attractive SERS targets due to their accessibility, diagnostic relevance, and structural diversity. Early work by Deng et al. examined both Raman and SERS spectra of the tobacco mosaic virus (TMV) coat protein using silver NP aggregates.^213^ They showed that adsorption time strongly influenced spectral features, with prolonged adsorption causing the disappearance of β-sheet (1244 cm^–1^) and α-helix (1290 cm^–1^) bands and the emergence of random coil signatures (1262 cm^–1^), consistent with surface-induced conformational rearrangement. More detailed structural insights were provided by Huang et al., who combined SERS and density functional theory (DFT) analysis to study the SARS-CoV-2 spike protein and its subunits using self-assembled AuNP substrates.^42^ The S1 subunit was dominated by β-sheet and random coil modes, whereas S2 exhibited pronounced α-helix and twisted β-sheet features; the receptor-binding domain (RBD) showed characteristic turn and β-sheet vibrations (Figure 11A). These assignments were consistent with known secondary structures. Comparative studies further demonstrated protein-specific spectral fingerprints. Sanchez et al. analyzed whole SARS-CoV-2 virions, isolated S-proteins, and N-proteins using Ag nanostructures, revealing distinct SERS signatures for each component, with the intact-virus spectrum reflecting a composite contribution from both proteins (Figure 11B).^43^ Extending this approach, Huang et al. compared S-protein spectra from five human coronaviruses (CoV-HKU1, CoV-OC43, SARS-CoV-2, SARS-CoV, and MERS-CoV) (Figure 11C).^42^ Species-dependent differences were observed primarily in the amide I and amide III regions, reflecting variations in secondary structure and amino-acid sequence. Notably, CoV-HKU1 and CoV-OC43 showed stronger Raman activity in the 400–800 cm^–1^ range, attributed to side-chain vibrations. Importantly, chemical or thermal inactivation of spike proteins produced minimal changes in their Raman fingerprints, indicating that commonly used inactivation protocols do not significantly perturb protein vibrational signatures.^42^ This robustness supports the feasibility of SERS-based protein detection in biosafety-compatible and clinically relevant workflows.

**Figure 11:**
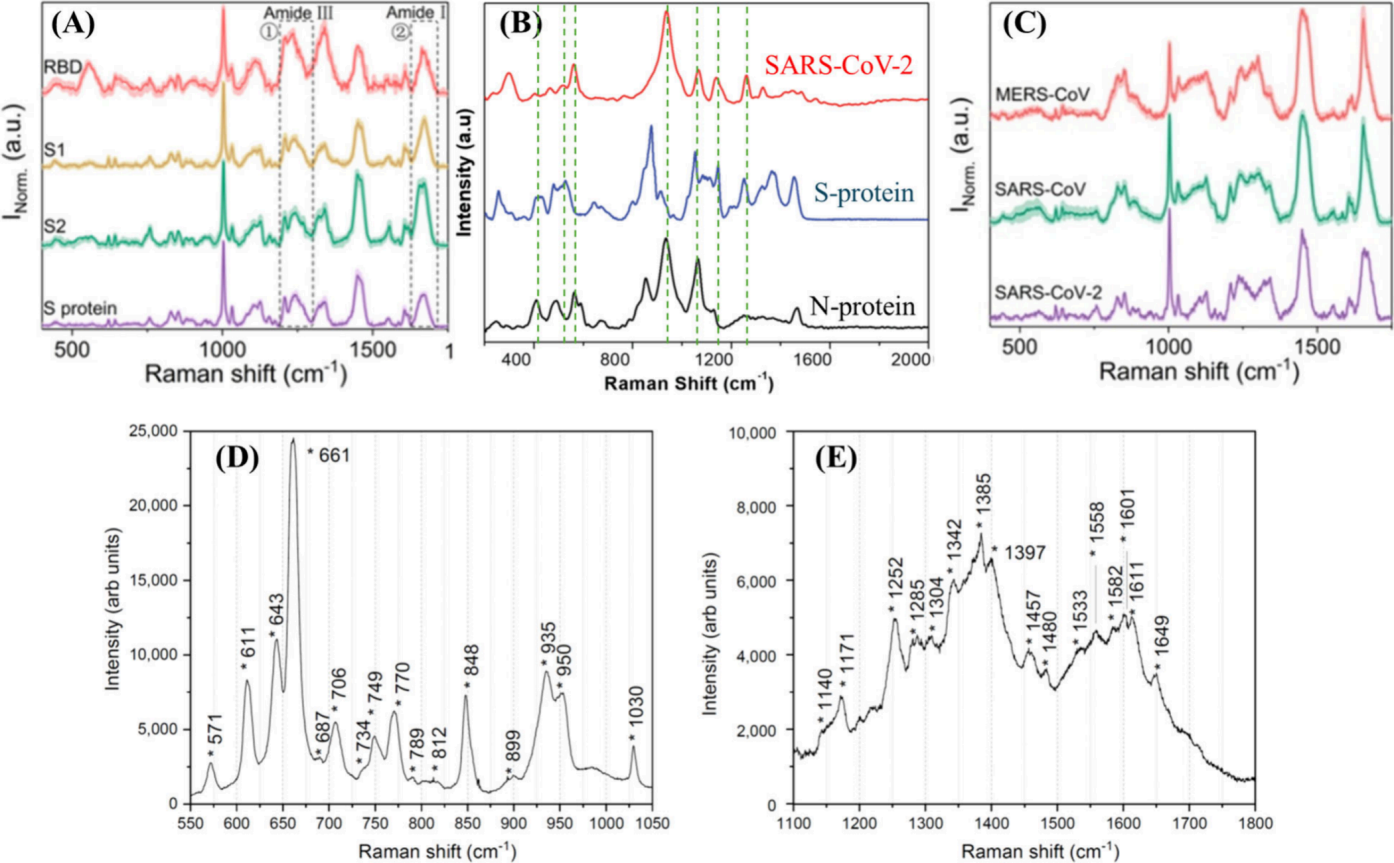
(A) Raman spectra of purified S protein and its S1 and RBD subunits; Reproduced with permission from ref 42. Copyright 2021, American Chemical Society. (B) SERS spectra of the N protein, S protein, and inactivated SARS-CoV-2 viral particles; Reproduced with permission from ref 43. Copyright 2021, Royal Society of Chemistry under CC BY-NC 3.0 https://creativecommons.org/licenses/by-nc/3.0/. (C) Comparison of Raman spectra of S proteins from different coronaviruses; Reproduced with permission from ref 42. Copyright 2021, American Chemical Society. Detailed regions of the genome SERS spectrum: (D) 550–1050 cm^–1^ and (E) 1100–1800 cm^–1^; Reproduced with permission from ref 44. Copyright 2022, MDPI (Basel, Switzerland) under CC-BY 4.0 https://creativecommons.org/licenses/by/4.0/.

#### 4.3.2 Direct Detection of Viral RNAs

Viral nucleic acids exhibit distinct Raman features arising from nucleobases and the sugar–phosphate backbone, making them viable targets for direct SERS analysis. Leonardi et al. recorded SERS spectra of the SARS-CoV-2 Omicron genome using Ag dendrite substrates, revealing well-resolved RNA vibrational signatures (Figure 11D).^44^ In the 550–1050 cm^–1^ range, prominent bands were assigned to nucleobase ring modes and backbone vibrations. The strongest peak corresponded to the guanine ring breathing mode at 661 cm^–1^, exceeding adenine (734 cm^–1^) and cytosine/uracil (789 cm^–1^), likely reflecting preferential guanine adsorption near plasmonic hotspots. Additional features included guanine-related modes (571, 610, 643, 687, 704 cm^–1^), O–P–O stretching of the phosphate backbone (812 cm^–1^), sugar–phosphate vibrations (848, 900 cm^–1^), and ribose bending (1030 cm^–1^). In the higher-frequency region (1100–1800 cm^–1^), overlapping in-plane ring vibrations and carbonyl stretches produced characteristic bands from guanine (∼1582 cm^–1^), adenine (∼1601 cm^–1^), and cytosine (∼1611 cm^–1^), confirming contributions from both nucleobases and the backbone (Figure 11E).

#### 4.3.3 The Performance of Viral Component Detection

Compared with intact-virus detection, direct SERS analysis of isolated viral proteins and RNA remains less explored and is often oriented toward spectral assignment rather than sensor benchmarking (see Table S3). Among the few quantitative studies, Huang et al. reported an LOD below 1 ppm for SARS-CoV-2 spike proteins,^42^ while Peng et al. achieved substantially higher sensitivity using semiconductor-based substrates, with reported LODs of 5 × 10^–9^ M^107^ and 10^–14^ M^214^ for S proteins. For RNA, an LOD of 10^4^ copies/mL has been achieved.^214^

## 5 Affinity-Based SERS Sensors

### 5.1 Basics of Affinity Sensing

Although direct SERS detection is rapid and label-free, its performance degrades in clinical specimens, where abundant proteins, salts, and metabolites competitively occupy plasmonic hotspots and obscure virus-specific signals. While ML can mitigate spectral complexity, its effectiveness depends on signal reproducibility, which is difficult to maintain under strong matrix interference. Affinity-based SERS sensors address these limitations by integrating selective capture elements with plasmonic substrates to enrich target analytes at hotspots, thereby improving sensitivity, specificity, and robustness in complex media (Figure 4B). The performance of affinity-based sensors is governed by six interrelated factors: receptor selection, substrate architecture, surface functionalization, binding kinetics, specimen environment, and spectral analysis.

#### 5.1.1 Selection of Receptors

Affinity receptors define the molecular specificity of SERS sensors and directly influence the sensitivity, reproducibility, and resistance to matrix interference. Commonly used receptors include antibodies,^215,216^ aptamers,^217–219^ host receptors,^220–222^ engineered molecules, peptides,^223,224^ or molecularly imprinted polymers (MIPs).^225^ Each class offers distinct advantages, depending on the viral target and application context. Antibodies remain the most widely used receptors due to their strong and well-characterized binding to viral epitopes; monoclonal antibodies provide high specificity, whereas polyclonal antibodies offer broader epitope coverage. Aptamers, composed of short DNA or RNA sequences, offer high affinity, chemical stability, and scalable synthesis, which makes them attractive for portable and cost-sensitive platforms. Host receptors, such as angiotensin-converting enzyme 2 (ACE2), enable biologically relevant recognition, particularly for coronaviruses, while synthetic peptides and MIPs provide customizable and often more-stable alternatives.

Receptor selection must be matched to the viral target, the specimen matrix, and intended deployment. For SARS-CoV-2, receptors have been designed to target the spike protein,^221,222,226–230^ nucleocapsid protein,^131^ or viral RNA.^231–235^ While many receptors perform well in simple buffers such as phosphate-buffered saline (PBS), complex biological fluids like saliva, blood, or urine require highly specific receptors such as monoclonal antibodies^216^ or aptamers^236,237^ to suppress nonspecific adsorption. Effective receptors typically exhibit dissociation constants below 10 nM, ensuring efficient, stable capture under flow and wash conditions.^237,238^ Binding kinetics further influence the response time and capture efficiency.

Practical considerations including manufacturability, thermal and chemical stability, batch reproducibility, and cost also shape receptor choice. Aptamers and peptides offer advantages in stability and scalability, whereas antibodies remain indispensable when maximal selectivity or complex epitope recognition is required. A curated summary of reported receptor–viral target pairings is provided in Table S4, serving as a practical reference for assay design.

#### 5.1.2 SERS Substrate Design

SERS substrates used in affinity-based virus sensors broadly fall into three categories: thin-film, NP-based, and hybrid architectures (Figure 2). Thin-film-based substrates include Ag nanorods^128–130,132,231^ and their modifications,^221^ Au inverted pyramidal nanohole array,^127^ Au-coated Silmeco,^222^ Au metasurface,^239^ Ag nanoforest,^229^ Au-coated triangular nanocavities,^227^ gold nanoislands on graphene,^228^ Au nanoneedle array,^131^ and Ag nanoislands.^240^ NP-based substrates are Au NPs,^133,232,241^ Ag NPs,^230,233^ Au nanostars,^242,243^ Au nanocrown,^226^ Au nanowires coated with Au@Ag bimetallic NPs,^234^ and Ag nanocubes.^108^ Hybrid systems, such as Fe_3_O_4_–Au core–shell NPs for virus capture, followed by dispersion onto Au nanoneedle arrays for SERS detection,^220^ provide enhanced performance through multifunctional properties. Additionally, some substrates incorporate nonmetallic components or hybrid materials for specialized applications. For instance, plasmonic-magnetic hybrid 3D graphene oxide architecture^244^ or Cu_2_O nanoarray^235^ has been explored for its unique advantages in virus affinity sensors.

#### 5.1.3 Receptor Immobilization and Capture Kinetics

The performance of affinity-based SERS virus sensors is governed jointly by how receptors are immobilized on plasmonic substrates and how receptor–analyte interactions proceed under surface-confined and matrix-rich conditions. These two factors are inseparable in practice, as immobilization chemistry directly influences receptor orientation, accessibility, binding kinetics, and susceptibility to nonspecific adsorption.

Receptors are most commonly immobilized via thiol–metal bonding, electrostatic adsorption, 1-ethyl-3-(3-dimethylaminopropyl)­carbodiimide/N-hydroxysuccinimide (EDC/NHS) covalent coupling, or click-chemistry-based conjugation. Thiol–metal attachment to Au or Ag substrates provides robust and oriented immobilization for antibodies, peptides, and aptamers, enabling stable sensing but limiting reversibility and reuse.^129,130,132,133,229–232,234,240,243^ Electrostatic adsorption offers rapid, modification-free attachment but suffers from weaker binding and sensitivity to pH and ionic strength.^127,131,220,221,227,242^ EDC/NHS coupling provides chemically stable conjugation across diverse substrates but requires careful control to preserve receptor activity.^226,228,241^ Click chemistry, particularly azide–alkyne cycloaddition and copper-free click reactions, offers a highly selective route for covalent receptor attachment and can improve conjugation specificity, linker stability, and site-controlled biomolecule presentation when appropriate functional groups are introduced on the receptor and substrate.^245–247^ For a broader overview of surface modification strategies for plasmonic biosensing, detailed reviews on chemical functionalization of plasmonic surfaces and nanomaterial surface-modification toolkits are available in the literature.^248,249^

Receptor–analyte binding on SERS substrates differs fundamentally from bulk solution behavior due to spatial confinement and field localization. Binding efficiency is dictated by the association and dissociation rates (*k*_*on*_, *k*_*off*_), with high-affinity interactions improving sensitivity but potentially limiting regeneration.^250,251^ Viral size, surface charge, and morphology further influence access to nanoscale hotspots, particularly for intact virions.^78^ Because viruses are typically tens to hundreds of nanometers in diameter, their Brownian diffusion is slow compared with small molecules. For example, virus diffusion coefficients are commonly on the order of 1–30 μm^2^/s, depending on particle size and medium viscosity;^252–255^ a 100 nm particle in water has an estimated diffusion coefficient of approximately 4 μm^2^/s based on the Stokes–Einstein relation. At this diffusion rate, the characteristic time to diffuse across a 100-μm liquid layer is on the order of tens of minutes, while diffusion across millimeter-scale distances may require many hours. Therefore, for surface-based SERS capture assays, mass-transport limitations can become important not only at extremely low viral concentrations but also at practically relevant dilute viral loads, such as 10^3^–10^5^ particles or copies/mL, where only tens to thousands of targets may be present in a typical microliter-scale assay volume. In addition, surface properties such as receptor density, orientation, nanoscale roughness, and local hydrophobicity further modulate binding efficiency.^256^ Extended incubation, reduced diffusion distance, convective mixing, microfluidic flow, or electrophoretic/magnetic enrichment may therefore be required to achieve reliable capture at the SERS substrate surface.^257^ Receptor orientation is another critical parameter because randomly immobilized antibodies or aptamers may partially block their antigen-binding domains or orient them away from the solution phase. For antibody-based SERS sensors, practical orientation strategies include the use of protein A or protein G intermediate layers, which bind the fragment crystallizable (Fc) region of IgG and thereby present the fragment antigen-binding (Fab) regions toward the sample solution. Such oriented immobilization can improve effective receptor accessibility, reduce steric hindrance, and enhance capture efficiency compared with random adsorption or nonspecific covalent coupling.^258^ Other strategies, including biotin–streptavidin coupling, histidine-tag/nickel–nitrilotriacetic acid (His-tag/Ni–NTA) interactions, and site-specific Fc or glycan modification, can also be used to control receptor presentation on plasmonic substrates.

Nonspecific binding is one of the central challenges in translating affinity-based SERS sensors from buffer solutions to complex clinical specimens. Saliva, serum, urine, respiratory secretions, and other biofluids contain abundant proteins, lipids, salts, extracellular vesicles, cellular debris, mucus components, and macromolecules that can adsorb onto plasmonic substrates or recognition layers, occupy hotspots, generate background SERS signals, block receptor access, and produce false-positive responses.^238,259–262^ These effects are especially problematic for SERS because enhancement is highly surface-localized; even small amounts of nonspecifically adsorbed material near hotspots can dominate the measured spectrum.^90^ Common mitigation strategies include antifouling coatings such as PEG, zwitterionic polymers, BSA, casein, or inert self-assembled monolayers; optimized receptor density and orientation; controlled spacer layers; blocking and washing steps; matrix dilution or buffer exchange; and specimen pretreatment such as filtration, centrifugation, target enrichment, or cleanup before SERS readout.^262–264^ However, antifouling or blocking layers can also increase analyte–metal separation and attenuate SERS enhancement, underscoring the need to balance selectivity, hotspot accessibility, and signal strength.^90^ Therefore, evaluation of affinity-based SERS sensors in clinically relevant matrices should include appropriate negative controls, nontarget virus controls, blank-matrix controls, and recovery tests to distinguish true target capture from nonspecific adsorption.^262^

#### 5.1.4 SERS Spectral Analysis and Interpretation

Unlike label-free detection, the analytical performance of affinity-based SERS virus sensors ultimately depends on the ability to accurately determine and interpret subtle spectral changes that occur upon target binding. Receptors such as antibodies, aptamers, or DNA probes often produce distinct SERS signals prior to target binding. Upon analyte capture, the resulting spectral changes are frequently modest because the receptor layer spatially separates the analyte from plasmonic hotspots, attenuating electromagnetic enhancement. This challenge is exacerbated when receptors and analytes share similar molecular features (e.g., protein–protein or DNA–RNA interactions), leading to overlapping spectral bands. In complex biological matrices, weak virus-induced changes may be further obscured by nonspecific adsorption and background interference, complicating reliable interpretation.

To address these challenges, multivariate analysis methods, including PCA, PLS-DA, and hierarchical clustering, have been widely applied to extract discriminative features and enable classification. While effective for well-controlled data sets, these linear approaches may struggle with weak, distributed signals in high-dimensional or noisy spectra. Supervised ML and DL models have emerged as powerful alternatives to analyze affinity-based SERS data. Techniques such as SVMs and RFs excel at identifying decision boundaries in moderate-sized data sets and offer interpretable feature rankings.^265^ For larger and more complex data sets, CNNs and ResNets can learn hierarchical representations directly from raw or minimally processed spectra, enabling both classification (e.g., virus identity, infection status) and regression (e.g., viral load quantification) tasks.^265^ Nevertheless, their reliability depends critically on reproducible high-quality data. Building standardized spectral libraries and pairing them with advanced algorithms will be essential to achieving accurate, robust, and clinically relevant SERS-based virus diagnostics.

### 5.2 Types of Affinity Sensors

Affinity-based SERS virus sensors are commonly classified by the type of receptor used to recognize and bind the viral targets. As summarized in Table S5, receptor choice influences immobilization strategies, specificity, signal characteristics, and overall detection performance.

#### 5.2.1 Antibody-Based Sensors

Antibodies (typically 10–15 nm; IgG most common) provide high specificity through epitope recognition and remain widely used for virus and viral-protein capture in complex specimens.^216,266,267^ They are commonly immobilized by physical adsorption^127,128,227,244^ or NHS/EDC coupling,^226,228,241^ with blocking steps (e.g., BSA) to reduce nonspecific adsorption.^268^ Antibody platforms have been reported for the detection of RSV,^128^ rotavirus (ROV),^244^ dengue virus (DENV) and West Nile virus (WNV),^241^ hepatitis A virus (HAV),^127^ and spike proteins of SARS-CoV-2 ^226–228^ .

#### 5.2.2 Viral Binding Protein-Based Sensors

Viral binding proteins (VBPs) mimic host–virus interactions and offer a biomimetic alternative to antibodies. ACE2 is widely used for SARS-CoV-2 sensing,^220–222^ while recombinant SCARB2 has been applied for enterovirus 71 (EV71).^242^ These receptors (typically 5–10 nm in size; e.g., ACE2 ∼ 90–100 kDa, ∼ 6–7 nm;^269^ scavenger receptor class B member 2 (SCARB2) ∼ 75–85 kDa, ∼ 6–8 nm^270^) can be immobilized via electrostatic,^271^ thiol,^272^ or hydrophobic attachment,^273^ but maintaining stable orientation and activity remains challenging, especially for weaker, reversible coupling methods.^242^

#### 5.2.3 Aptamer-Based Sensors

Aptamers (15–60 nucleotides, folded size ∼ 1–3 nm) enable closer placement of the target to hotspots than bulky proteins, often improving sensitivity and reproducibility.^274–276^ They are chemically synthesized with low batch variability and selected by systematic evolution of ligands by exponential enrichment (SELEX) for specific viral proteins or nucleic-acid targets.^217–219^ Reported systems include aptamers for SARS-CoV-2 spike^229,230^ and nucleocapsid,^50,129,130,277^ IAV hemagglutinin,^240^ envelope protein,^278^ surface domains,^279,280^ as well as RNA fragments^234^ (see Tables S4 and S5). Immobilization typically uses terminal thiols to Au/Ag^229,230,234,240^ or disulfide anchoring;^129,130^ spacers (e.g., polyethylene glycol (PEG) or alkanethiols) help preserve folding and reduce steric/nonspecific effects.^281–286^

#### 5.2.4 Nucleic Acid Probe-Based Sensors

Hybridization probes (ssDNA/RNA; commonly 15–40 bases, 0.34 nm/base) provide sequence-level specificity for viral genomes.^287^ Examples include probes targeting SARS-CoV-2 RNA,^231,235^ influenza RNA,^132,133^ and tobacco mosaic virus RNA.^243^ Probes are typically anchored by thiol–metal coupling,^132,133,231^ or attached using cationic layers such as polyethyleneimine (PEI) to capture negatively charged nucleic acids.^233^ Because probe and target chemistries are similar, many assays rely on multivariate analysis for robust readout.

#### 5.2.5 Other Recognition Elements

Synthetic receptors can improve robustness and manufacturability. Molecularly imprinted polymers (MIPs) create target-shaped binding cavities and offer high stability, but careful control is required to achieve target specificity.^288–290^ Peptides provide compact, designable binders; ACE2-derived peptide sensors have been demonstrated for SARS-CoV-2 spike detection, including nanomolar-level quantification using multivariate analysis.^222,291,292^

### 5.3 Performance of Affinity Sensors

As summarized in Table S5, affinity-based SERS virus sensors primarily target three analyte classes: (i) intact viruses, (ii) viral proteins, and (iii) viral nucleic acids (RNA/DNA). Antibodies, aptamers, and viral binding proteins are most commonly used for capturing intact virions and surface proteins (e.g., SARS-CoV-2 spike, influenza hemagglutinin),^130,221^ whereas nucleic acid probes rely on sequence-specific hybridization for RNA/DNA detection.^132,133,231,232,293,294^ Below, we briefly summarize representative performance trends, focusing on sensitivity, specificity, and quantification.

#### 5.3.1 Intact Virus Capture and Detection

Early demonstrations established the feasibility of affinity-assisted intact virus detection. Tripp et al. employed IgG2a-functionalized AgNRs to detect RSV, where virus binding generated new spectral features beyond the antibody background (Figure 12A).^128^ Fan et al. used antibody-functionalized plasmonic–magnetic graphene oxide for ROV detection, observing distinct virus-induced peaks at 445, 580, 960, and 1330 cm^–1^ together with shifts in graphene oxide bands (Figure 12B).^244^ Paul et al. reported antibody-immobilized Au NPs for DENV and WNV, where multiple virus-related peaks emerged with concentration-dependent intensity increases (Figure 12C).^241^ In contrast, Rippa et al. observed only modest intensity changes at pre-existing antibody bands after SARS-CoV-2 binding, with minimal new features (Figure 12D).^227^ Viral binding proteins provide a biomimetic alternative to antibodies. Li et al. immobilized His-tagged ACE2 on Au nanoneedles, where SARS-CoV-2 binding introduced additional bands between 835 and 1495 cm^–1^ relative to weak prebinding protein signals (Figure 12E).^220^ Zhao’s group further demonstrated ACE2-functionalized SiO_2_-coated AgNR arrays, where virus-specific changes in the 600–777, 817–877, and 1521–1649 cm^–1^ regions enabled differentiation of SARS-CoV-2 variants and CoV-NL63 (Figure 12F).^221^ By comparison, SCARB2-coated Au nanostars showed only subtle spectral differences before and after EV71 binding (Figure 12G).^242^ Aptamer-based platforms have also shown strong clinical promise. Park et al. developed a silver nanoforest aptasensor capable of distinguishing SARS-CoV-2 variants (wild-type, Delta, Omicron).^229^ Although detailed spectra were not reported, the assay achieved high diagnostic performance across 80 clinical specimens, with near-perfect classification (AUC ≈ 1.0).

**Figure 12:**
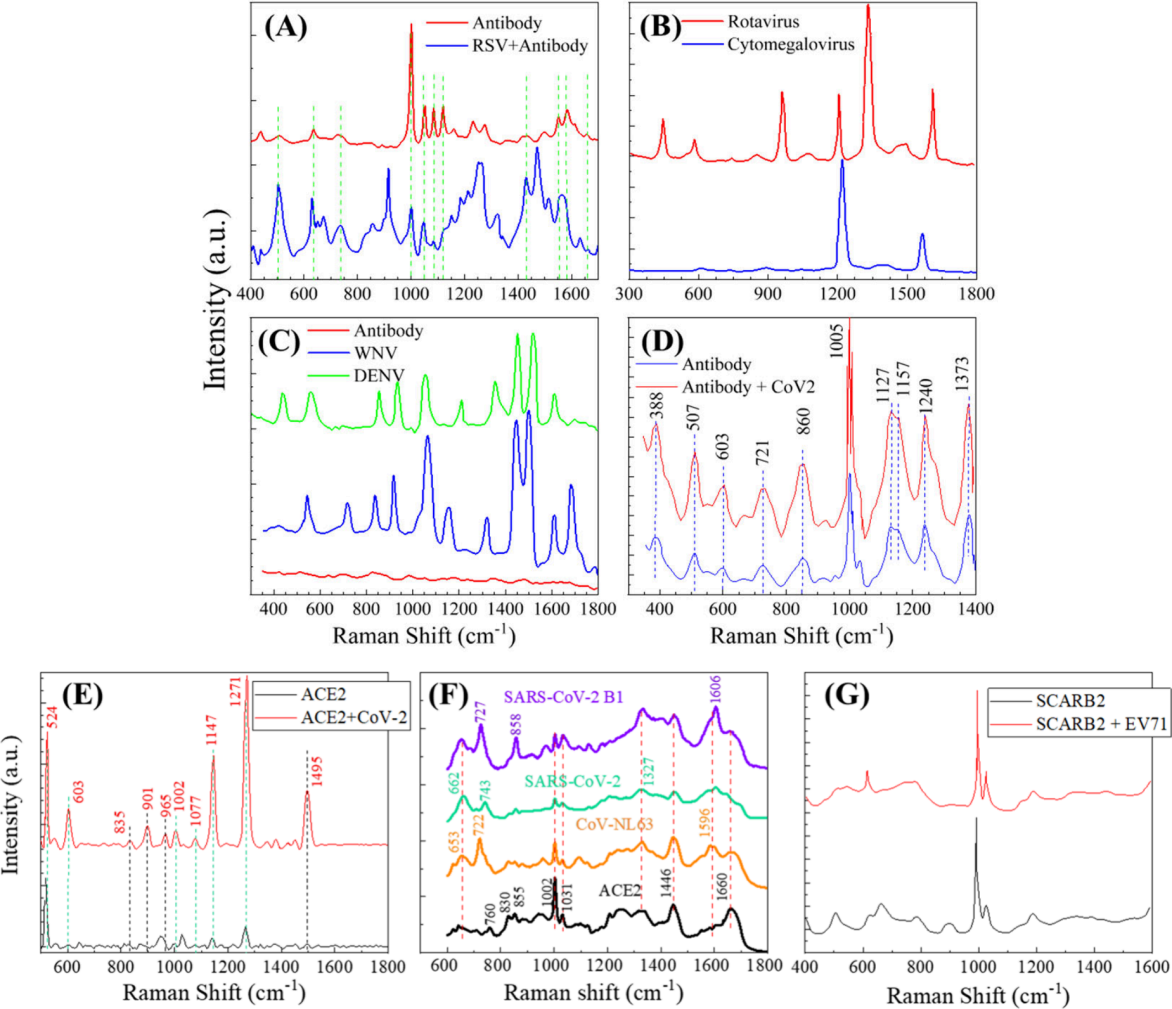
SERS spectra of antibody-functionalized substrates before and after virus binding for: (A) RSV; Adapted with permission from ref 128. Copyright 2008, Elsevier. (B) ROV; Adapted with permission from ref 244. Copyright 2014, American Chemical Society. (C) WNV and DENV; adapted with permission from ref 241. Copyright 2015, American Chemical Society. (D) SARS-CoV-2; Adapted with permission from ref 227. Copyright 2024, American Chemical Society. SERS spectra illustrating viral-binding protein receptors and their interactions: (E) ACE2 on Au nanoneedles with SARS-CoV-2 binding; Adapted with permission from ref 220. Copyright 2022, Elsevier. (F) ACE2 on SiO_2_-coated AgNR substrate showing binding of multiple CoV strains; Adapted with permission from ref 221. Copyright 2024, American Chemical Society under CC-BY 4.0 https://creativecommons.org/licenses/by/4.0/. (G) SCARB2 on Au nanostars with EV71 binding; Adapted with permission from ref 242. Copyright 2017, American Chemical Society. All spectra are replotted from the original publications.

Collectively, these studies reveal substantial variability in spectral response, ranging from new virus-specific peaks to small intensity shifts driven by differences in virus type, receptor choice, immobilization chemistry, substrate architecture, and measurement conditions. This variability underscores the need for standardized protocols and advanced spectral analysis methods.

Despite these challenges, sensitivity and speed are competitive. Antibody- or ACE2-based platforms have reported limits of detection down to ∼ 10 PFU/mL for DENV, WNV, and SARS-CoV-2,^221,241^ and ∼ 100 copies/mL for pseudoviruses in nasal or throat swabs,^220^ with typical response times of 15–20 min. Specificity is generally high: antibody sensors discriminate DENV from WNV and chikungunya virus (CHIKV),^241^ SCARB2 sensors distinguish enterovirus 71 (EV71) from DENV,^242^ ACE2-based platforms differentiate multiple coronaviruses and SARS-CoV-2 variants,^222^ and aptamer sensors selectively identify Delta and Omicron variants in complex media.^229^

For quantification, two strategies dominate: (1) intensity-based calibration using virus-induced peaks or intensity changes;^127,242^ and (2) ML-assisted analysis when spectral differences are subtle. CNNs, SVMs, and multivariate curve resolution have proven especially effective; for example, CNN analysis of ACE2–AgNR@SiO_2_ substrates enabled accurate classification and quantification of SARS-CoV-2 variants within ∼ 20 min.^221^

#### 5.3.2 Viral Protein Detection

Affinity-based SERS detection of viral proteins exploits the same receptor classes used for intact viruses but benefits from better-defined targets and reduced steric constraints. Antibody-based sensors have been widely demonstrated. Wang et al. immobilized anti-SARS-CoV-2 spike antibodies on gold nanoislands on graphene, where protein binding produced pronounced intensity changes in antibody bands (1077, 1330, 1581 cm^–1^) and introduced new spike-related features at 525, 728, 1000, 1023, 1178, 1423, and 1467 cm^–1^ (Figure 13A).^228^ Biomimetic receptors provide complementary performance. ACE2-functionalized gold nanoneedles generated clear new spike-protein bands at 762, 950, 1302, and 1395 cm^–1^,^131^ whereas ACE2-coated AgNR@SiO_2_ substrates exhibited only subtle spectral differences among SARS-CoV-2 variants (Figure 13B), highlighting the difficulty of variant discrimination by direct inspection alone.^221^

**Figure 13:**
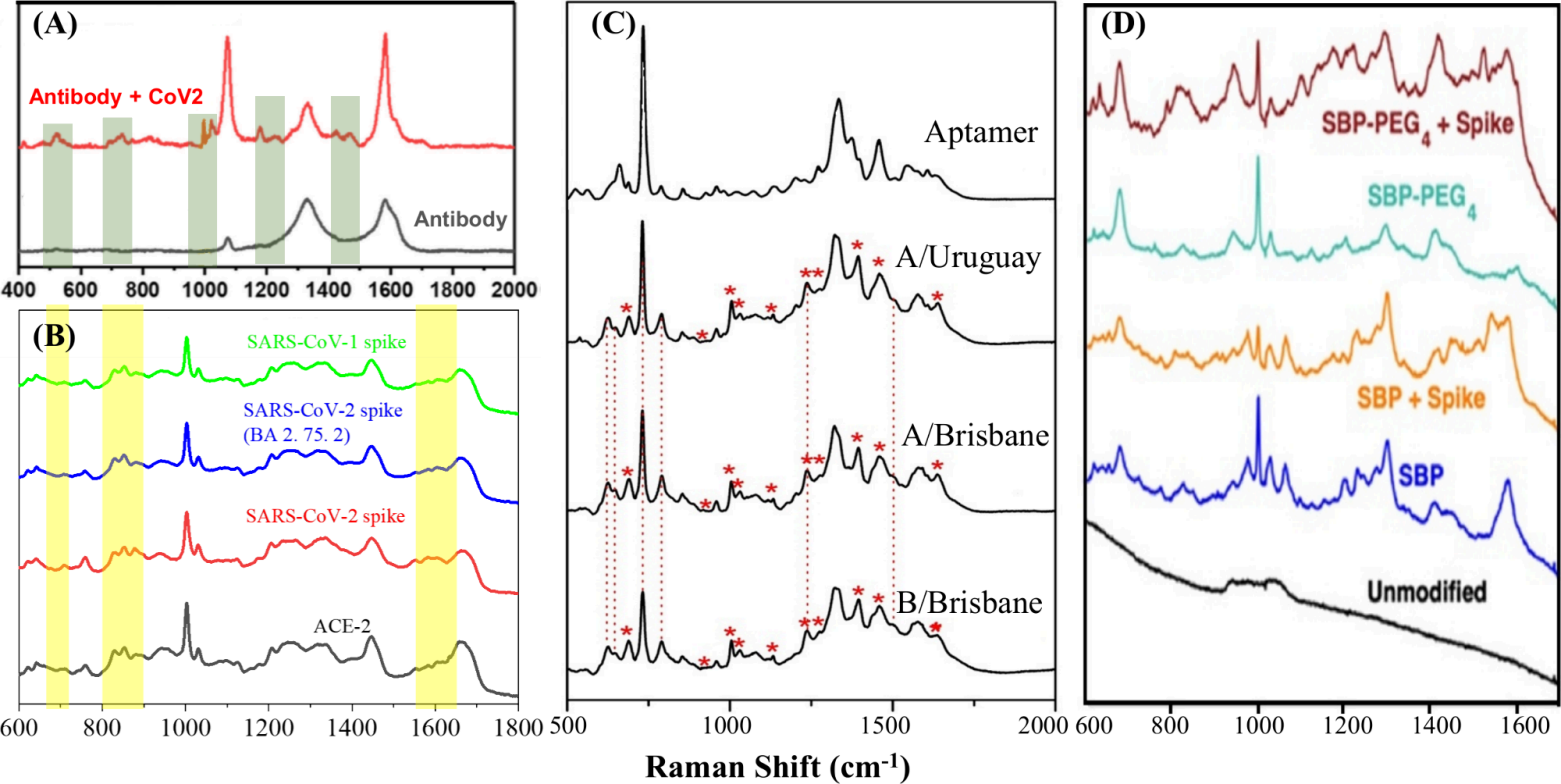
SERS spectra illustrating receptor–virus binding interactions: (A) Antibody alone and antibody bound to the spike protein; Adapted with permission from ref 228. Copyright 2024, Elsevier. (B) ACE2 alone and ACE2 bound to spike proteins from three SARS-CoV-2 variants; Adapted with permission from ref 221. Copyright 2024, American Chemical Society under CC-BY 4.0 https://creativecommons.org/licenses/by/4.0/. (C) Aptamer alone and aptamer bound to IAV nucleoproteins from three influenza A strains; Adapted with permission from ref 130. Copyright 2012, American Chemical Society. (D) Peptide alone and peptide bound to the SARS-CoV-2 spike protein; Reproduced with permission from ref 222. Copyright 2021, American Chemical Society. All spectra are replotted based on the original published data.

Aptamer-based sensors often yield stronger responses due to their compact size. Negri et al. detected influenza nucleoproteins using aptamer-modified AgNRs, observing new protein-associated peaks at 1004, 1238, 1440–1460, 1592–1602, and 1610 cm^–1^ (Figure 13C).^129,130^ Comparable results were reported using silver nanoislands^240^ and AgNPs.^230^ For SARS-CoV-2, Park et al. identified high-affinity aptamers (SpS1-C1, SpS1-C4) targeting the S1 subunit, where binding caused reproducible intensity reductions near 1587 cm^–1^, consistent with conformational changes.^229^ Peptide-based recognition has also been demonstrated: an ACE2-derived peptide immobilized on gold via Au–S bonding (with PEG spacers) captured spike protein and produced multiple new backbone- and side-chain–related bands between 793 and 1601 cm^–1^, confirming specific peptide–protein interactions (Figure 13D).^222^

Across platforms, protein-induced spectral changes are often subtle or masked by receptor signals, particularly at low concentrations, limiting single-peak analysis. Multivariate and ML-based methods are therefore essential. PCA and HCA have enabled influenza strain classification,^129,130,230^ multivariate curve resolution improved peptide-based sensing,^222^ and CNN analysis of ACE2–AgNR@SiO_2_ substrates successfully differentiated SARS-CoV-2 variants.^221^ ROC curve analysis further validated near-perfect classification for aptamer-based nanoforest sensors in clinical swabs.^229^

In terms of sensitivity, aptamer-based sensors consistently outperform other receptors, achieving LODs as low as 240 aM for SARS-CoV-2 spike protein in nasopharyngeal swabs (NPS),^229^ and 1 fM on AgNP substrates, and 190 particles/mL on nanoisland substrates.^230,240^ ACE2-based platforms reached 17.7 pM in PBS and 80 copies/mL in urine, benefiting from up to 10^6^-fold enrichment.^131^ Antibody-based sensors typically achieved fg/mL–ng/mL LODs, depending on substrate and immobilization strategy.^226,228^

High specificity has been demonstrated across the receptor classes. ACE2 mimetic peptides selectively recognized SARS-CoV-2 spike over SARS-CoV-1 and MERS-CoV,^222^ ACE2- AgNR@SiO_2_ substrates differentiated closely related coronaviruses via CNN analysis,^221^ and aptamer-based sensors showed negligible cross-reactivity in complex biological media.^230,240^ Collectively, these studies establish affinity-based SERS protein sensors as highly sensitive and selective while underscoring the need for advanced data analysis to extract diagnostic information from weak or overlapping spectral changes.

#### 5.3.3 Viral Nucleic Acid Capture

Affinity-based SERS nucleic acid sensors employ immobilized ssDNA or antisense oligonucleotide probes that hybridize with complementary viral RNA or DNA, enabling sequence-specific detection. Early demonstrations targeted virulence-associated influenza mutations, such as the PB1-F2 gene^132^ and neuraminidase stalk motifs in N1 influenza A strains.^133^ For SARS-CoV-2, antisense probes and ssDNA sensors targeting conserved regions of the N gene enabled robust detection across variants, including Omicron.^231,232^ Beyond clinical viruses, Kissell et al. developed a SERS-active hydrogel sensor for TMV RNA that recognized both conserved and partially complementary sequences, demonstrating applicability in environmental samples.^243^

Despite high molecular specificity, hybridization-induced SERS changes are typically subtle because RNA and DNA share similar backbones. Most studies report only small peak shifts or intensity variations.^132,133,231,232,243^ In Yang et al.’s SARS-CoV-2 RNA sensor (Figure 14A), unbound probes exhibited characteristic nucleotide bands, including guanine (700 cm^–1^), adenine (738, 1329, 1458 cm^–1^), cytosine/thymine (794 cm^–1^), and phosphate backbone (1096 cm^–1^).^231^ After RNA binding, these features persisted with only minor changes near 700 and 900–1100 cm^–1^, rendering visual discrimination difficult. Spectra collected from 10^3^ to 10^9^ copies/mL showed nearly identical shapes (Figure 14B), requiring ML-based regression. An RNN model achieved excellent quantitative performance (*R* ^2^ = 0.9985; RMSEP = 0.0136; Figure 14C).

**Figure 14:**
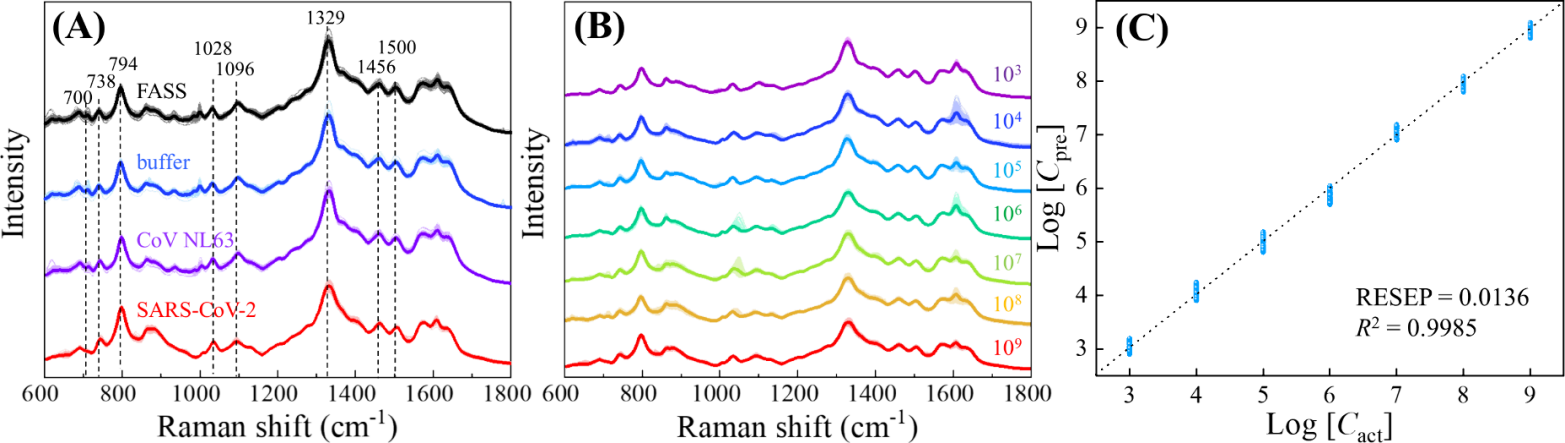
(A) SERS spectra of the ssDNA-modified AgNR substrate (black), buffer (blue), detection of SARS-CoV-2 RNA (red), and CoV-NL63 RNA (purple). (B) Concentration-dependent SERS spectra of SARS-CoV-2 RNA from 10^3^ to 10^9^ copies/mL. (C) RNN-based regression results for predicting SARS-CoV-2 RNA concentration. Panels A-C are adapted with permission from ref 231. Copyright 2023, American Chemical Society.

Sensitivity of nucleic acid SERS sensors spans from nanomolar levels to below 100 copies/mL. For example, AuNPs functionalized with antisense oligonucleotides detected SARS-CoV-2 RNA at 63 copies/mL in nasal swabs,^232^ while AgNR-based sensors achieved ∼ 10 nM LODs for influenza RNA,^132^ and comparable performance for TMV RNA in agricultural fluids.^243^ Specificity is dictated primarily by probe design and hybridization fidelity. Perfect discrimination between complementary and noncomplementary sequences has been achieved using HCA,^132^ while PLS-DA and SVM-DA enabled >95% classification of high- and low-virulence influenza motifs.^133^ For SARS-CoV-2, targeting conserved N-gene regions yielded ∼ 100% sensitivity and ∼ 90% specificity in clinical testing,^232^ with PCA and RNN classifiers further distinguishing SARS-CoV-2 from CoV-NL63.^231^ Cross-reactivity tests against unrelated viruses confirmed high selectivity.^243^

#### 5.3.4 Other Viral Biomolecules

Beyond viral proteins and nucleic acids, infection-induced metabolic changes provide alternative, noninvasive biomarkers for SERS-based diagnostics. Viral infections can perturb host metabolism and oxidative stress pathways, leading to characteristic breath volatile organic compounds (BVOCs) that serve as indirect indicators of infection.^158^ A representative example is a handheld SERS breathalyzer developed for COVID-19 screening.^108^ This device used Ag nanocube substrates functionalized with 4-mercaptobenzoate (MBA), 4-mercaptopyridine (MPY), and 4-aminothiophenol (ATP) to promote hydrogen bonding, ion–dipole, and π–π interactions with BVOCs. These interactions concentrate target molecules near SERS hotspots, enhancing sensitivity. Notably, the MBA C–S stretching mode at 521 cm^–1^ decreased markedly upon exposure to BVOCs such as ethanal, heptanal, octanal, and acetone, with stronger signal attenuation observed in COVID-19-positive breath samples than in controls.^295^ Multivariate analysis (PLS-DA) enabled clear discrimination, achieving 96.2% sensitivity and 99.9% specificity for rapid screening. These results demonstrate that SERS can capture indirect metabolic signatures of viral infection, expanding diagnostic capabilities beyond direct molecular targets and highlighting the versatility of SERS for noninvasive, high-throughput virus detection.

## 6 Labeled Detection

Labeled SERS detection addresses key limitations of label-free affinity sensing, including weak intrinsic Raman cross sections of viral components, spectral interference from complex matrices, and variability in virus–substrate interactions. By introducing external Raman reporters with strong, stable, and spectrally distinct signatures, labeled strategies decouple detection from intrinsic viral signals, enabling ultrahigh sensitivity, quantitative analysis, and multiplexed detection in clinical specimens (Figure 4C).

### 6.1 Basics of Labeled Detection

In labeled SERS virus detection, analytical performance is governed not only by the choice of recognition elements and substrates but, critically, by the design and implementation of SERS tags^296^ and their associated signal generation and signal processing strategies. Unlike label-free approaches that rely on weak and variable intrinsic viral Raman signals, labeled SERS decouples detection from viral molecular scattering by introducing engineered nanoparticles that carry strong, stable Raman reporters. As a result, assay sensitivity, reproducibility, multiplexing capability, and quantitative reliability are determined by a combination of SERS tag architecture, target amplification or enrichment mechanisms, and robust quantification strategies.

#### 6.1.1 SERS Tag Design and Functionalization

In labeled SERS detection, the SERS tag serves as the primary signal transducer and is typically composed of four elements: (i) a plasmonic core (commonly Au, Ag, or Au/Ag hybrid nanoparticles) that provides electromagnetic enhancement; (ii) a Raman reporter that generates a strong and distinct spectral fingerprint; (iii) a recognition element (e.g., antibodies, aptamers, nucleic acid probes, or receptor proteins) for selective viral binding; and (iv) a protective or stabilizing layer (e.g., PEG, silica, polymers, or proteins) that improves colloidal stability and suppresses nonspecific adsorption. While recognition chemistry and substrate engineering have been widely explored in affinity-based platforms, labeled SERS places additional emphasis on how reporters are incorporated, stabilized, and spatially coupled to plasmonic nanostructures to ensure sensitivity, reproducibility, and multiplexing capability. SERS tag fabrication generally involves three steps:^296^ (i) synthesis of metallic NPs, (ii) functionalization with Raman reporters, and (iii) conjugation with recognition elements, followed by stabilization. Gold and silver NPs are commonly used due to their strong plasmonic properties, with their size and shape tailored to optimize field enhancement.^296,297^ Reporter molecules, often organic dyes like rhodamine 6G, crystal violet, malachite green, or Nile blue, provide distinct spectral signatures.^298^ Aromatic thiols such as 4-mercaptobenzoic acid (4-MBA), 4-mercaptopyridine (4-MPY), and 4-nitrobenzenethiol (4-NBT) are also widely used because they bind strongly to metal surfaces through thiol groups.^299^ After reporter loading, NPs are conjugated with recognition elements (e.g., antibodies, aptamers, or nucleic acids) via covalent methods like EDC/NHS or PEG linkers, or noncovalently through electrostatic or hydrophobic interactions.^300^ Stabilizers, such as PEG or BSA, are added to minimize nonspecific binding and aggregation. Protective shells such as silica or polymers (e.g., polydopamine, poly­(vinyl alcohol)) further enhance durability, reduce reporter loss, and improve reproducibility.^300^

#### 6.1.2 Nucleic Acid Amplification Strategies

Because viral nucleic acids are often present at very low abundance during early infection, the performance of labeled SERS assays frequently relies on either molecular amplification of target sequences or physical enrichment of analytes near plasmonic hotspots.^58,301–303^ Molecular amplification strategies encompass three major classes. Enzyme-assisted isothermal methods, including recombinase polymerase amplification (RPA), loop-mediated isothermal amplification (LAMP), rolling circle amplification (RCA), and strand displacement amplification (SDA), rapidly increase target copy number under mild conditions and are well suited for point-of-care formats.^304–308^ Enzyme-free strand-assembly schemes, such as hybridization chain reaction (HCR), catalytic hairpin assembly (CHA), and DNAzyme-based amplification, offer improved stability and simplified workflows, albeit with slower kinetics.^135,309–311^ CRISPR–Cas-assisted strategies exploit sequence-specific recognition and collateral nuclease activity: activation of Cas12a or Cas13a by viral nucleic acids triggers cleavage of reporter-tagged ssDNA or RNA, generating strong SERS signals.^312–317^ Hybrid approaches (e.g., RPA–Cas12a or CHA–Cas13a) further enhance sensitivity, in some cases enabling single-copy detection.^318,319^ Each class presents trade-offs between speed, robustness, complexity, and cost; thus, no single amplification strategy is universally optimal, and method selection depends on the desired sensitivity, assay format, and operational constraints. Collectively, these approaches substantially lower detection limits while maintaining the sequence specificity required to discriminate closely related viral strains and variants.

#### 6.1.3 Physical Enrichment Strategies

In parallel, physical enrichment strategies enhance labeled SERS detection by locally concentrating viral analytes at plasmonic hotspots, thereby improving signal strength and reproducibility without altering molecular composition. These methods use external fields to manipulate particles in solution and accelerate target–substrate interactions. Electrohydrodynamics (EHD) generates microscale fluid vortices under applied electric fields, increasing mass transport and driving viral particles toward SERS-active regions.^320,321^ Dielectrophoresis (DEP) employs nonuniform electric fields to selectively trap and position viruses based on their polarizability, enabling precise localization on nanostructured substrates.^322^ By preconcentrating viral analytes, both EHD and DEP lower detection limits, reduce assay time, and enhance reproducibility, features that are particularly valuable in early-stage diagnostics and real-time monitoring applications.^323^

#### 6.1.4 Signal Quantification Strategies

In labeled SERS virus detection, quantification is achieved by tracking the Raman intensity of reporter molecules within SERS tags. Because signal intensity is sensitive to substrate heterogeneity and sample matrix effects, robust quantification relies on strategies that improve reproducibility. Three approaches are commonly used: intensity-based calibration, ratiometric normalization, and digital SERS. Intensity-based calibration correlates a characteristic reporter peak (e.g., 4-MBA at ∼ 1080 cm^–1^) with analyte concentrations but is vulnerable to variations in hotspot distribution, nanoparticle aggregation, and matrix effects, limiting accuracy. Ratiometric methods mitigate these issues by normalizing the reporter signal to an internal Raman standard with a nonoverlapping peak.^324^ For example, a Cyanine 3 (Cy3)/4-MBA ratiometric aptasensor for SARS-CoV-2 reduced signal variability and improved the detection limit.^325^ By normalizing the Cy3 peak at 1470 cm^–1^ to the 4-MBA peak at 1075 cm^–1^, the relative standard deviation was reduced from 9.1% to 2.3%, and the limit of detection improved from 3.75 to 0.53 PFU/mL in buffer and to 3.67 PFU/mL in spiked clinical samples. An emerging alternative, digital SERS, converts continuous intensity measurements into binary detection events.^326^ In this approach, individual pixels or sampling points in a SERS map are classified as positive or negative based on a predefined intensity threshold, and the total number of positive events (“digital counts”) is correlated with analyte concentration. By replacing analog intensity averaging with statistical counting, digital SERS substantially reduces the impact of substrate heterogeneity, background interference, and hotspot variability.^327,328^ This strategy has enabled ultralow detection limits at the femtogram-per-milliliter (fg/mL) or attomolar (aM) level, surpassing conventional analog SERS and colorimetric assays.^326^ For instance, digital SERS-based LFA achieved detection limits as low as 0.9 fg/mL (1.9 aM) for the SARS-CoV-2 nucleocapsid protein, 2 orders of magnitude lower than analog SERS and up to 7 orders of magnitude more sensitive than traditional colorimetric lateral flow immunoassay (LFIA) tests.^326^

### 6.2 Types of Labeled Detection Mechanisms

Labeled SERS detection comprises a set of assay formats that convert molecular recognition events into quantifiable Raman signals. Among these, sandwich assays are the most widely used owing to their generality, high specificity, and compatibility with intact viruses, viral proteins, and nucleic acids. In addition, signal-on and signal-off mechanisms exploit target-induced changes in plasmonic coupling or reporter–substrate proximity to amplify or attenuate Raman signals.

#### 6.2.1 Sandwich Assays

Sandwich assays rely on dual recognition, where a target analyte is captured by a substrate-immobilized probe and subsequently recognized by a reporter-labeled SERS tag (Figure 15). This two-site binding strategy suppresses nonspecific signals and underpins the broad adoption of sandwich assays for viruses such as SARS-CoV-2,^329,330^ influenza,^331–334^ RSV,^335^ and norovirus (NoV).^109,336^ A typical sandwich assay consists of three components: (1) a capture substrate functionalized with antibodies, aptamers, or nucleic acid probes; (2) the target analyte; and (3) a SERS tag carrying a Raman reporter and a secondary recognition element. Assay performance is strongly influenced by target properties, including size, surface accessibility, and epitope distribution.^337^ Features such as the molecular size, surface accessibility, and epitope availability influence binding efficiency and signal generation. For example, large virions may require spatially open capture architectures, while highly glycosylated proteins demand high-affinity probes to overcome steric effects.^338^ Nucleic acid targets similarly require precise probe design to ensure selective hybridization.^339^

**Figure 15:**
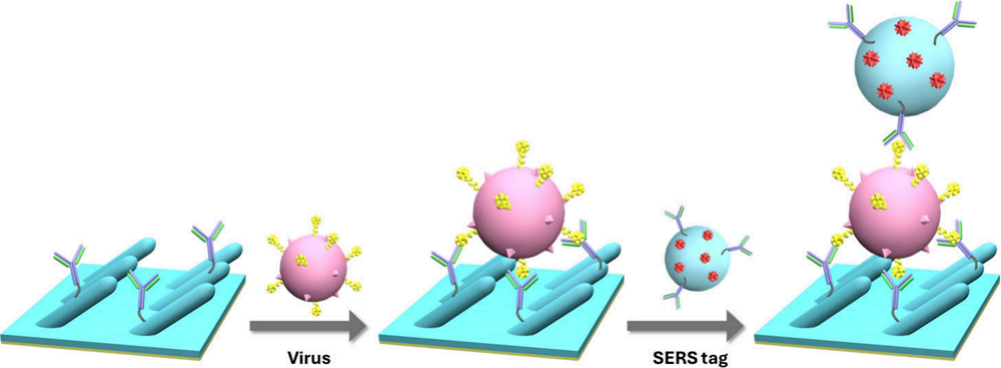
Schematic illustration of a sandwich assay for viral detection: surface-immobilized capture ligands first bind the target virion or antigen, followed by recognition with a labeled detection ligand that targets a nonoverlapping epitope to form a capture–target–detector complex.

Sandwich assays can be implemented in either solid-phase^116^ or liquid-phase^330^ formats. Solid-phase assays, using nanostructured substrates (typically thin-film-based) such as nanorods or nanohole arrays, offer high enhancement and low background but may be diffusion-limited. Liquid-phase assays, which rely on freely dispersed NPs, enable faster kinetics and simpler workflows but can suffer from aggregation and an elevated background. In practice, solid-phase sandwich assays are preferred when precision and reproducibility are critical, whereas liquid-phase formats offer advantages in speed and portability.^340^

#### 6.2.2 Signal-On Mechanisms

Signal-on mechanisms generate increased Raman intensity upon target recognition by enhancing plasmonic coupling or relocating reporters into electromagnetic hotspots. While sandwich assays represent the most common realization of signal-on detection, several alternative strategies exploit target-induced structural or assembly changes (Figure 16A–C).

**Figure 16:**
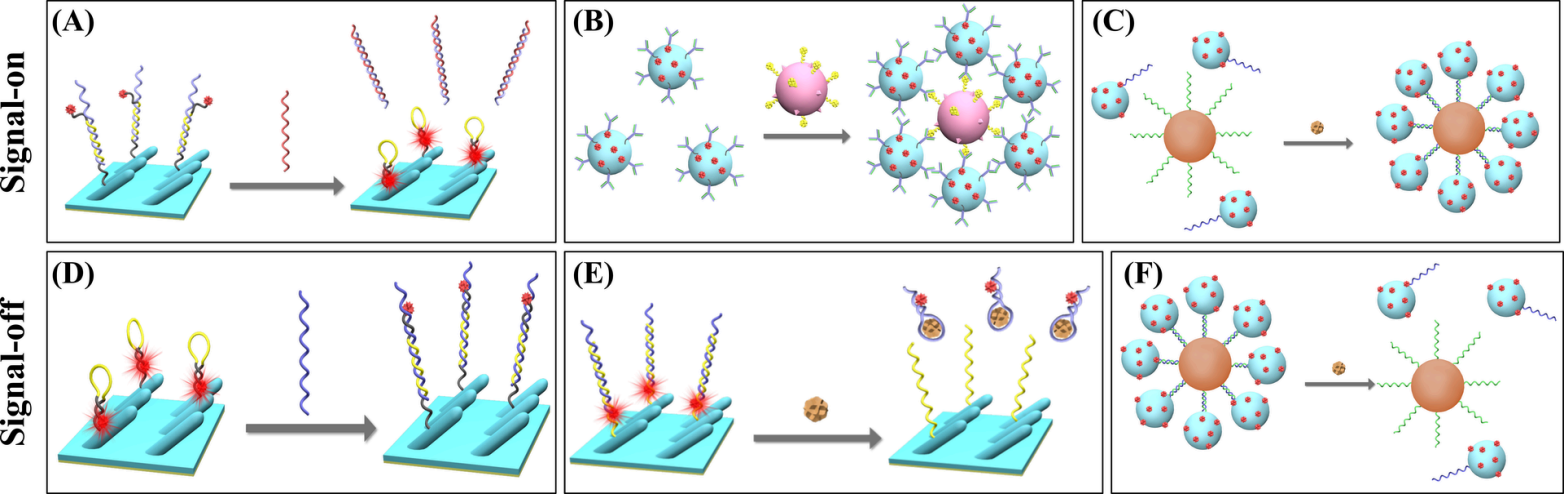
Representative signal-on and signal-off mechanisms in labeled SERS assays. Top: Signal-on strategies, including (A) target-induced probe reconfiguration, (B) analyte-mediated nanoparticle aggregation, and (C) target-triggered nanoparticle assembly. Bottom: Signal-off strategies, including (D) conformationally induced signal attenuation, (E) target-induced release, and (F) target-triggered disassembly.

Conformational change-based designs, most commonly molecular beacons (MBs), use target-induced folding or unfolding of nucleic acid probes to reposition Raman reporters relative to the plasmonic surface.^243,341^ Upon target binding via nucleic acid hybridization^243,342^ or aptamer–protein binding,^325^ the probe undergoes a conformational transition (e.g., hairpin formation) that relocates the reporter into a plasmonic hotspot, producing a strong signal increase (Figure 16A). These designs offer high sensitivity and sequence-level specificity and are well suited for rapid and point-of-care detection,^343^ although performance can be affected by ionic strength, pH, nuclease activity, and nonspecific adsorption in complex matrices.^344,345^

Target-induced aggregation (TIA) amplifies SERS signals by driving the clustering of functionalized nanoparticles into plasmonic nanogaps.^346–349^ Aggregation can be mediated by aptamer–protein interactions,^346,348^ antibody–antigen binding,^350^ nucleic acid hybridization,^351^ or electrostatic effects,^352^ with signal intensity scaling with hotspot density (Figure 16B). While TIA provides strong amplification, reproducibility depends critically on surface chemistry and aggregation control.

Target-triggered assembly (TTA) offers a more programmable signal-on strategy by inducing ordered nanoparticle architectures only in the presence of the target.^135,136,353,354^ Using molecular recognition processes such as nucleic acid hybridization, catalytic hairpin assembly, or toehold-mediated strand displacement, TTA systems organize NPs into defined geometries with reproducible nanogaps, improving both the sensitivity and signal consistency (Figure 16C). Their modularity and controlled hotspot formation make TTA particularly attractive for sensitive viral diagnostics and point-of-care applications.

From an analytical perspective, signal-on designs are generally advantageous for trace detection, because target recognition produces a positive intensity change above a low initial background. This format avoids much of the penalty associated with measuring a small decrease from a strong or variable baseline, as in signal-off assays. As a result, signal-on sensors often provide improved apparent signal-to-noise ratios, clearer concentration-dependent responses, and lower ambiguity at low analyte concentrations. These advantages are consistent with broader SERS biosensing principles, where background suppression and positive reporter generation are critical for sensitive detection.^355^ In contrast, signal-off responses can be more susceptible to false-positive or false-negative interpretation when signal loss arises from nonspecific adsorption, incomplete washing, reporter leakage, photobleaching, nanoparticle instability, or matrix-induced attenuation rather than from true target recognition. Therefore, when the primary goal is to maximize sensitivity and quantitative reliability in trace viral detection, signal-on mechanisms are often preferred.

#### 6.2.3 Signal-Off Mechanisms

Signal-off mechanisms invert the signal-on paradigm by producing a measurable decrease in SERS intensity upon target recognition and expand the toolkit for labeled SERS assay engineering. Rather than creating new hotspots, target binding disrupts plasmonic coupling or increases reporter–substrate separation, yielding signal attenuation (Figure 16D–F).

Signal-off MBs initially place Raman reporters near the plasmonic surface to generate a strong baseline signal. Hybridization with complementary DNA or RNA unfolds the probe, increasing reporter–surface distance and causing a quantifiable decrease in intensity (Figure 16D).^52,122,123,356,357^ Reliable performance requires stable probe architectures and uniform substrates.

Target-induced release (TIR) suppresses SERS signals by displacing reporter-labeled probes from plasmonic hotspots upon analyte recognition. Release can occur through binding–mediated release,^121^ nucleic acid hybridization-driven strand exchange,^358^ and aptamer conformational switching for viral protein detection,^359^ resulting in reduced local field enhancement (Figure 16E). CRISPR–Cas-enabled TIR is particularly powerful: activation of Cas12a leads to nonspecific cleavage of ssDNA linkers, releasing SERS tags and producing rapid signal attenuation with attomolar sensitivity.^121,316,317^

Target-triggered disassembly (TTD) represents the conceptual reverse of TTA.^360,361^ In TTD systems, plasmonic NPs are preorganized into highly enhancing architectures (e.g., aggregates or core–satellite structures) using molecular linkers. Target recognition disrupts these linkers via strand displacement, conformational change, or enzymatic cleavage, increasing interparticle spacing and reducing plasmonic coupling (Figure 16F). The resulting signal decrease correlates with target concentration and can be driven by aptamer–antigen or antibody–antigen interactions, nucleic acid hybridization,^360,361^ or enzymatic cleavage. Controlled architectures generally offer improved reproducibility and signal predictability compared to simple aggregates.^362^

### 6.3 Performance of Labeled Sensors

Building on the detection mechanisms discussed above, this section summarizes the reported performance of labeled SERS strategies for viral detection, focusing on sensitivity, specificity, reproducibility, assay speed, and applicability in complex matrices.

#### 6.3.1 Intact Virus Detection

Labeled SERS platforms demonstrate strong performance for intact virus detection across multiple assay formats. As summarized in Table S6, sandwich immunoassays dominate due to their dual-recognition specificity, modularity, and quantitative robustness.^54,57,109,329–336,349,363–370^ Alternative formats include aggregation-based approaches (TIA/TTA), where virus binding induces NP clustering and hotspot formation,^348^ and TIR strategies that suppress reporter signals upon recognition.^371^ Sensitivity is a major strength: many sensors achieve LODs of just 1–5 PFU/mL or a few fg/mL, comparable to or surpassing PCR-based methods.^54,109,329,331,334–336,368^ Reported dynamic ranges typically span 10^1^–10^5^ PFU/mL for SARS-CoV-2 and down to ng levels for norovirus.^57,109,330,336,364,365,367,369^ High specificity enables discrimination among viral strains, variants, and drug-resistant mutants, particularly for influenza and SARS-CoV-2.^54,329,332–334,363,364,368^ Reproducibility is generally good, with relative standard deviations (RSDs) below 8% and stability maintained over weeks to months under appropriate storage conditions.^109,336,365,366,369,370^ Importantly, these sensors perform reliably in complex matrices including serum, saliva, nasal swabs, fecal samples, and environmental surfaces.^54,57,109,329,330,336,348,349,364,368^ Assay times range from <10 min for simplified formats (e.g., aptamer strips, LFAs) to 30 min for most platforms, while more elaborate sandwich or ELISA-integrated SERS assays require 1–5 h but offer enhanced precision and multiplexing.^109,330,333,335,365,367,369^

#### 6.3.2 Viral Protein and Antibody Detection

Labeled SERS assays are extensively applied to viral protein biomarkers, which are often more stable and abundant than intact virions. As summarized in Table S7, sandwich immunoassays dominate, with over 20 reported studies across diverse viral proteins,^101,363,365,367^ including SARS-CoV-2 spike, nucleocapsid, and RBD,^116,326,372^ influenza A/B proteins,^115,263,373,374^ Hepatitis B surface antigen (HBsAg),^375,376^ West Nile virus,^377^ Zika virus,^378^ Rift Valley fever virus,^103^ and rotavirus.^379^ Reported platforms include magnetic bead-assisted assays,^101,103^ microfluidic devices,^376^ LFAs,^326,372^ and digital single-NP counting.^380^ Signal-off aptasensors^325^ and assembly-based strategies^353^ are less common but demonstrate mechanistic versatility. These assays achieve exceptional sensitivity, often reaching fg/mL–pg/mL levels. Representative examples include SARS-CoV-2 spike protein at 0.77 fg/mL in PBS and 6.07 fg/mL in saliva,^116^ HBsAg at 2.88 pg/mL,^375^ and influenza nucleoprotein at 6 TCID_50_/mL.^381^ Multiplexed platforms such as the electrohydrodynamic nanobox-on-mirror system enable simultaneous detection of SARS-CoV-2, influenza A/B, and RSV at sub-pg/mL levels with >96% clinical sensitivity and specificity.^320,321^ Typical assay times are 8–30 min, extending to 1–2 h for amplification-assisted formats. High specificity is consistently reported, enabling discrimination among closely related antigens (e.g., SARS-CoV-2 over influenza or rotavirus,^371^ influenza H3N2 and H1N1 ^115^), even in complex matrices such as fecal or serum samples (norovirus and hepatitis).^109^ This performance is driven by high-affinity capture elements, including monoclonal antibodies, aptamers, scFv fragments, and ACE2-derived peptides.

Labeled SERS is also highly effective for viral antibody detection, critical for serology and immune monitoring. As summarized in Table S8, SERS-based LFIAs are the dominant format,^107,110,382^ using substrates such as SiO_2_@Ag core–shell particles,^110^ gap-enhanced nanotags,^107^ and dye-labeled gold nanostars.^382^ Most target SARS-CoV-2–specific IgM and IgG. Reported LODs reach 100 fg/mL for IgM^382^ and 1 pg/mL for combined IgM/IgG,^110^ representing up to 800-fold improvements over conventional LFIA. Detection ranges span fg/mL to ng/mL. These assays also support multiplexing (simultaneous IgM/IgG),^382^ deliver results in 15–30 min, and remain stable for up to two months,^107^ confirming strong clinical potential.

#### 6.3.3 Viral Nucleic Acid Capture

Labeled SERS assays targeting viral DNA and RNA exhibit outstanding sensitivity and flexibility (Table S9). Sandwich hybridization platforms remain the most widely used (>20 studies) due to their reliability and simplicity.^112,383–386^ Conformational probes such as MBs and molecular sentinel probes are widely adopted for single-base mismatch discrimination, enabling genotyping and mutation analysis.^52,122,123,356,357,387^ More advanced mechanisms, including TTA/TTD^135,136,354,360,361^ and TIR,^121^ further expand assay design, though less frequently reported. Detection limits routinely reach the aM–nM range, rivaling or exceeding qPCR. Examples include hepatitis B virus DNA detection at 50 aM using an Ag nanorice–Au nanoarray substrates;^384^ amplification-free CRISPR–Cas12a assay achieving single aM detection for HBV, HPV-16, and HPV-18.^121^ High specificity is maintained, including single-nucleotide polymorphism discrimination (e.g., H1N1 mutations^361^). Multiplexed assays enable simultaneous detection of multiple pathogens, such as Rift Valley fever virus and West Nile virus.^388^ Most nucleic acid SERS assays deliver results within 15–60 min, suitable for point-of-care testing. Examples include HIV-1 LFIA readout in ∼ 15 min^53^ and RSV RNA detection within 1 h using molecular sentinel probes.^356^ Cascade amplification strategies (e.g., HCR, CHA) extend assay time to several hours but enable fM–aM sensitivity.^135,136^ Probe stability is generally good, with storage lifetimes up to one month reported.^357^

Amplification remains a key enabler of clinical translation. PCR–SERS hybrids reduce thermal cycling (e.g., 8 cycles for 100-copy LOD),^389^ while isothermal approaches (LAMP-SERS,^390^ exonuclease-assisted cycling,^136^ and CHA/HCR cascades^134,354^ ) eliminate thermocycling and enhance sensitivity. CRISPR–Cas12a/Cas13a platforms further simplify workflows,^391–394^ with CRISPR–CHA combinations providing up to 500-fold sensitivity enhancement over CRISPR alone.^386^ This broad toolbox enables tuning of assay speed, complexity, and sensitivity for diverse diagnostic settings.

Overall, SERS-based nucleic acid assays now match or exceed PCR in sensitivity (aM–fM), mismatch discrimination, and multiplexing capability, with RSDs often below 10%. Many deliver results within one hour, some within minutes, and function effectively in complex matrices. While PCR remains the regulatory gold standard, SERS assays are well positioned as complementary tools for rapid prescreening, multiplexed detection, and field-deployable diagnostics.

## 7 From SERS Biosensors to Viral Diagnostics in Clinical Specimens

A SERS biosensor becomes a diagnostic only when its analytical output is connected to a specific clinical or public health decision. Many SERS studies demonstrate sensitive detection of viral particles, proteins, nucleic acids, antibodies, or host-response biomarkers under controlled conditions. These studies establish biosensing feasibility, but they do not by themselves define a clinically useful diagnostic. For diagnostic translation, the assay must be designed around an intended use: who will use the test, which specimen will be collected, when during infection the test will be applied, what result will be reported, and what decision will follow.

Building on direct, affinity-based, and labeled SERS strategies established under controlled conditions (Sections 4–6), clinical translation introduces additional variability from complex specimen matrices. Common specimens including NPS, saliva, blood, feces, and cerebrospinal fluid (CSF) contain proteins, enzymes, lipids, salts, and cellular debris that can attenuate Raman signals and/or generate strong fluorescence backgrounds. Viral load also varies across patients and disease stage, and biosafety inactivation can modify viral structures, together degrading sensitivity, reproducibility, and clinical reliability. Despite these challenges, > 110 studies have demonstrated SERS-based virus detection in clinical matrices. Approximately 20 used direct (label-free) detection on unprocessed or minimally processed specimens (e.g., saliva, nasal swabs, cell lysates) (see refs 36, 41, 46, 47, 124, 137, 164–168, 173,^174^,^179^,^395^,^396^ ) (Table S2). About 12 applied affinity-based sensors (antibodies, aptamers, ACE2) to specimens such as NPS, serum, urine, or lysates (see refs 129–131, 220, 227, 229, 231, 232, 234,^240^ ) (Table S5). Another 81 employed labeled assays validated in complex matrices including saliva, serum, feces, blood, nasal aspirates, and cell lysates (see refs 54, 57, 109, 329–332, 334–336, 363–366,^368–370^ ) (Tables S6–S9). Collectively, these results support SERS as a viable route toward real-world and potentially multiplexed viral diagnostics.

This distinction between biosensing feasibility and diagnostic utility is especially important, because different viral diseases require different end-user workflows. For acute respiratory infections such as influenza, RSV, and COVID-19, the diagnostic goal is often rapid triage, isolation guidance, antiviral-treatment decisions, or multiplexed differentiation among pathogens with overlapping symptoms. In this setting, saliva or nasal swabs, short turnaround time, simple sample handling, and robust point-of-care operation are critical. For chronic bloodborne infections such as HIV, HBV, or hepatitis C virus, the diagnostic need may involve screening, confirmation, viral load monitoring, or treatment-response assessment; therefore, quantitative accuracy, broad dynamic range, blood matrix compatibility, and integration with laboratory workflows become more important. For gastrointestinal viruses such as norovirus or rotavirus, stool complexity makes sample preparation and matrix rejection central to diagnostic reliability. For neurotropic infections detected in CSF, a limited sample volume and low viral titer impose stringent sensitivity and workflow constraints.

Therefore, successful clinical deployment requires treating the specimen preprocessing method, spectral analysis pipeline, and decision threshold as integral parts of the diagnostic strategy. Matrix-aware collection and handling are needed to preserve targets, remove interferents, and enrich analytes, while standardized spectral preprocessing, including baseline correction, normalization, and deconvolution, is needed to manage drift, overlap, and fluorescence. Robust chemometric/ML pipelines can then extract subtle virus-associated signatures and, when interpretable, connect discriminative features to plausible molecular origins. In this intended-use framework, the target analyte, receptor chemistry, substrate architecture, sample preparation method, and performance metrics should be selected according to the disease-specific diagnostic decision rather than analytical sensitivity alone.

### 7.1 Types of Clinical Specimens and Matrix Characteristics

The successful clinical translation of SERS-based viral diagnostics depends critically on the choice and handling of biological specimens. The specimen type directly determines collection procedures, preprocessing requirements, spectral background, and ultimately diagnostic reproducibility and reliability. Selection is guided by viral tissue tropism, infection stage, and clinical application. Respiratory viruses are most commonly sampled via NPS/oropharyngeal swab (OPS)^397^ and saliva,^398^ whereas blood,^399^ stool,^400^ and CSF^401^ are preferred for systemic, enteric, or neurotropic infections.

Each specimen matrix presents distinct chemical and physical challenges for SERS analysis. Swab eluates and saliva are rich in salts, mucins, and proteins that can mask viral Raman features and compete for adsorption on plasmonic surfaces. Blood-derived specimens contain high concentrations of hemoglobin, albumin, and lipids, generating strong fluorescence backgrounds and broad scattering. Stool samples represent the most complex matrices, with intense light scattering, enzymatic activity, and microbial interference that obscure viral signatures. CSF is comparatively clean but typically contains very low viral titers, placing stringent demands on detection sensitivity.

These matrix-dependent constraints require specimen-specific preprocessing. Most clinical samples require clarification, filtration, and buffer exchange into Raman-quiet media prior to SERS analysis. Protein-rich matrices such as serum and stool often require additional fractionation or inhibitor removal to suppress background signals. Viral inactivation procedures, whether thermal or chemical, must balance biosafety with the preservation of diagnostically relevant molecular features. Even relatively clean specimens such as CSF benefit from gentle clarification and microconcentration to improve the signal quality.

Specimen properties also dictate the most appropriate SERS detection strategies. Direct, label-free SERS is best suited for relatively clean specimens, such as saliva or clarified nasal fluids, where intrinsic viral biomolecular signatures can be accessed. Affinity-based SERS methods extend compatibility to moderately complex matrices, including serum or urine, by leveraging selective capture to suppress nonspecific background. Labeled SERS formats are the most robust for highly complex or turbid samples, such as blood, feces, or sputum, as signal generation is decoupled from the native spectral contributions of the specimen matrix. Aligning specimen type with the appropriate SERS strategy, together with matrix-aware preprocessing and robust spectral analysis, is therefore essential for achieving reliable, sensitive, and clinically reproducible viral diagnostics. The major matrix characteristics, dominant challenges, key preprocessing steps, and SERS strategy compatibility for common clinical specimens are summarized in Table 2.

**Table 2:** Clinical Specimens for Viral Diagnostics: Collection, Composition, Preparation, and SERS Implications

Specimen	NPS/OPS	Saliva	Blood/Serum/Plasma	Stool	CSF
Typical viruses	Influenza, RSV, SARS-CoV-2	Respiratory and enteric viruses	HIV, HBV, HCV, dengue, Zika, WNV	Norovirus, rotavirus, adenovirus	Herpes simplex virus (HSV), enterovirus, varicella-zoster virus (VZV)
Dominant matrix composition	Mucus, salts, ribonucleases (RNases); viral transport medium (VTM)/guanidinium background	Proteins, enzymes, microbial background	High protein and fluorescence; anticoagulants	Extreme complexity, enzymes, fluorescence	Low viral titer, dilute matrix
Key preparation steps	Mucus liquefaction, low-speed spin, filtration, buffer exchange	Centrifugation, filtration, Raman-quiet buffer	Spin, protein removal, virion enrichment	Homogenization, clarification, inhibitor removal	Clarification, ultrafiltration
SERS strategy suitability	Direct (after cleanup); affinity; labeled	Direct (favored), affinity, labeled	Affinity, labeled (preferred)	Labeled (most robust)	Affinity, labeled
Reference	^397^,^402^	^398^,^403^	^404^,^405^	^406^,^407^	^408^

### 7.2 Standardized Preparation and Detection Workflow

Clinical SERS-based viral diagnostics require a standardized matrix-aware workflow to ensure sensitivity, reproducibility, and clinical relevance (Figure 17). The process begins with selecting an appropriate specimen type (e.g., NPS, saliva, blood, and CSF) based on viral tropism and diagnostic goals. Specimens are collected using sterile, optimized protocols to preserve viral integrity and minimize contamination. Matrix-specific preprocessing then reduces background interference and enriches viral targets through clarification (centrifugation or filtration), viscosity reduction (enzymatic treatment), and concentration (e.g., ultrafiltration or PEG precipitation). Selective molecular recognition using antibodies, aptamers, or oligonucleotide probes further isolates and concentrates viral analytes and forms the basis for direct, affinity-based, or labeled SERS detection; direct intrinsic SERS detection does not necessarily require a selective capture step. These steps can be integrated into microfluidic chips or LFA formats to enable automation, reduce sample volume, and support POC operation. When required, molecular or physical amplification strategies are incorporated (Section 6.1). Raman or SERS spectra are collected using benchtop or portable instruments, followed by spectral preprocessing (baseline correction, smoothing, and normalization) and chemometric or ML analysis to determine viral presence, load, or multiplexed signatures. Diagnostic outputs are benchmarked against gold standards, such as RT-PCR or ELISA, using sensitivity, specificity, and LOD metrics. Continuous optimization of specimen handling and assay design remains essential because of matrix variability.

**Figure 17:**
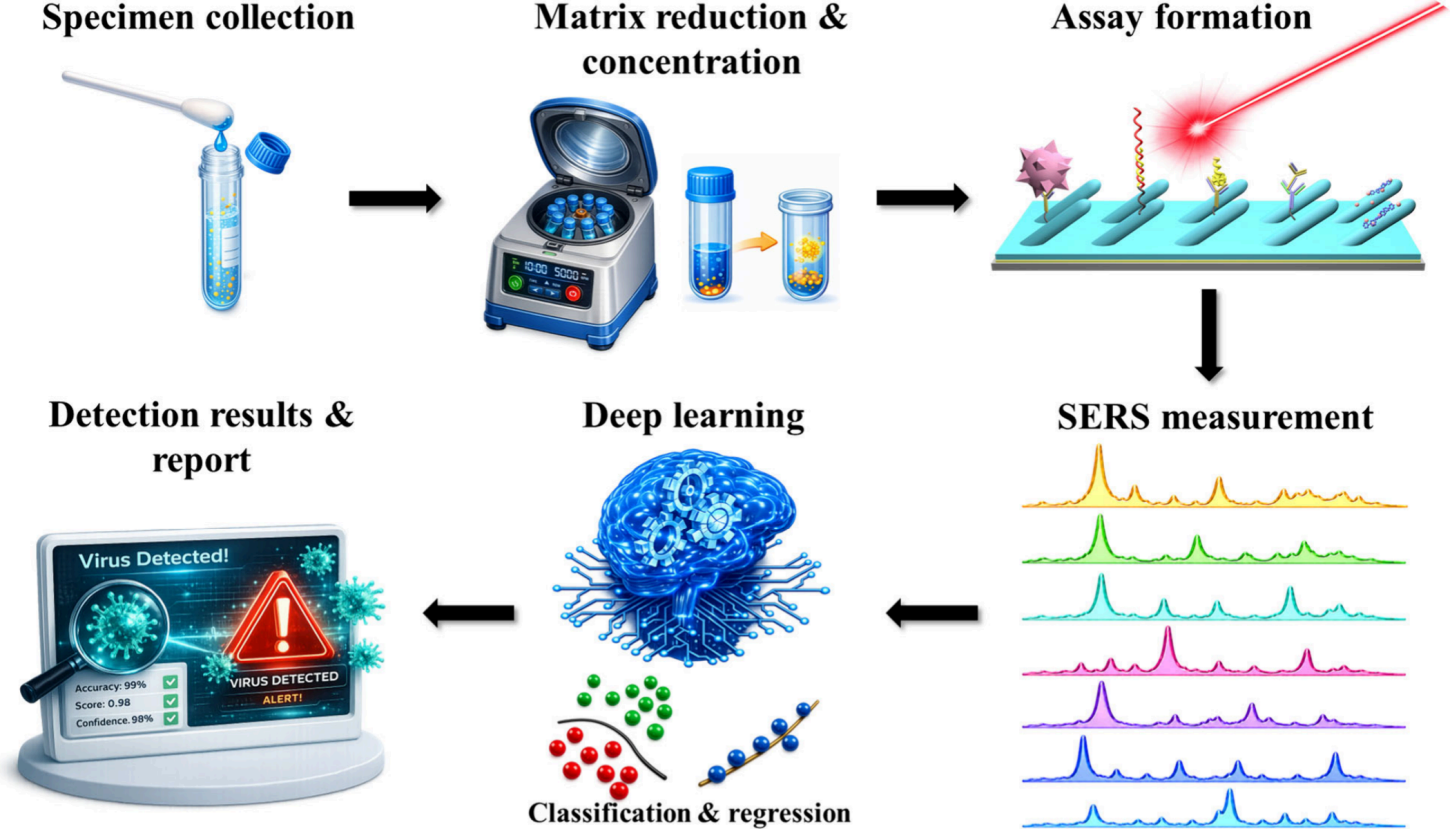
Standardized workflow for SERS-based viral detection from clinical specimens. Samples (e.g., swabs, saliva, blood, CSF) undergo matrix-aware preprocessing, including clarification, viscosity reduction, and target enrichment, to minimize background interference. Selective capture using antibodies, aptamers, or probes enables affinity-based or labeled SERS detection, which can be integrated into microfluidic or lateral flow platforms for POC use. Direct intrinsic SERS detection does not necessarily require a selective-capture step. Acquired Raman/SERS spectra are preprocessed and analyzed by chemometric or machine learning methods to determine viral presence and load, with performance benchmarked against gold-standard assays such as RT-PCR or ELISA.

#### 7.2.1 Viral Inactivation

Viral inactivation is essential for biosafety but can substantially alter Raman and SERS signatures. Common methods include heat treatment, chemical fixation (e.g., formaldehyde, β-propiolactone, ethanol, detergents), and UV irradiation. While effective at eliminating infectivity, these treatments can collapse viral envelopes, disrupt capsids, denature proteins, or damage nucleic acids, leading to band shifts, peak attenuation, and increased fluorescence backgrounds.^409^ Mild heat or low-dose UV often preserves diagnostically useful features, whereas harsh chemical or thermal treatments frequently cause irreversible spectral changes.^410^

The impact of inactivation depends strongly on the detection strategy. Direct SERS is most sensitive to structural disruption; even modest alterations can weaken adsorption or shift key vibrational bands. Pezzotti et al. showed pronounced protein and DNA damage in inactivated HSV-1 virions,^411^ while Andreeva et al. demonstrated that SERS fingerprints of inactivated influenza A virions differ systematically from intact particles and should not be used as diagnostic references.^412^ For affinity-based assays, inactivation primarily affects molecular recognition: denaturation of surface antigens decreases antibody or aptamer binding, and nucleic acid damage reduces hybridization efficiency. Selecting epitopes and probe sequences that remain structurally stable postinactivation can partially mitigate these effects. Labeled SERS assays are generally more tolerant because their readout derives from external Raman reporters; however, similar to affinity detection, severe inactivation can still destroy binding epitopes required for capture or sandwich formation. Using denaturation-resistant antibody pairs and optimizing tag-to-target ratios can help preserve assay performance.

#### 7.2.2 Specimen Storage and Freeze–Thaw

Specimen storage is often unavoidable but can degrade molecular integrity through ice-crystal formation, protein denaturation or aggregation, lipid oxidation, and metabolite degradation.^413–415^ These changes manifest in SERS spectra as intensity fluctuations, band shape distortion, or elevated background fluorescence, compromising sensitivity and classifier accuracy, especially in direct and affinity-based detection. Labeled SERS is comparatively resilient but remains susceptible to nanoparticle aggregation or tag instability during a prolonged storage. To maintain spectral integrity and diagnostic reliability, SERS workflows should employ standardized storage and quality control protocols. Recommended best practices include^416,417^ (1) storing specimens under validated temperature and duration conditions appropriate for each biological matrix, (2) aliquoting specimens into single-use volumes to prevent repeated thawing, and (3) incorporating stability controls such as same-batch reference aliquots to identify storage-related batch effects. Whenever possible, freshly processed specimens should be prioritized for the spectral acquisition and model calibration. When stored or inactivated specimens must be analyzed, their spectral and diagnostic equivalence to fresh materials should first be confirmed to ensure consistent classifier performance and preserve the overall analytical robustness.

#### 7.2.3 Clarification (Low-Speed Centrifugation and Filtration)

Clarification removes cells, mucins, and particulates that block plasmonic hotspots or generate Raman background.^78,418^ Low-speed centrifugation and membrane filtration can improve SNR and spatial reproducibility but must be optimized to avoid unintended loss of virus-containing complexes. Spike–recovery assays and pellet/supernatant analysis are essential for validating analyte retention.^415,419,420^ Optimized clarification significantly improves diagnostic performance. For example, 30-kDa ultrafiltration enhanced dengue virus detection in serum by unmasking low-molecular-weight biomarkers,^421^ while 50-kDa filtration improved hepatitis B viral load prediction.^422^ Similar benefits were observed for HCV RNA detection, achieving *R* ^2^ ≈ 0.91 and RMSEP ≈ 0.32 log IU/mL after clarification (Figure 18).^423^

**Figure 18:**
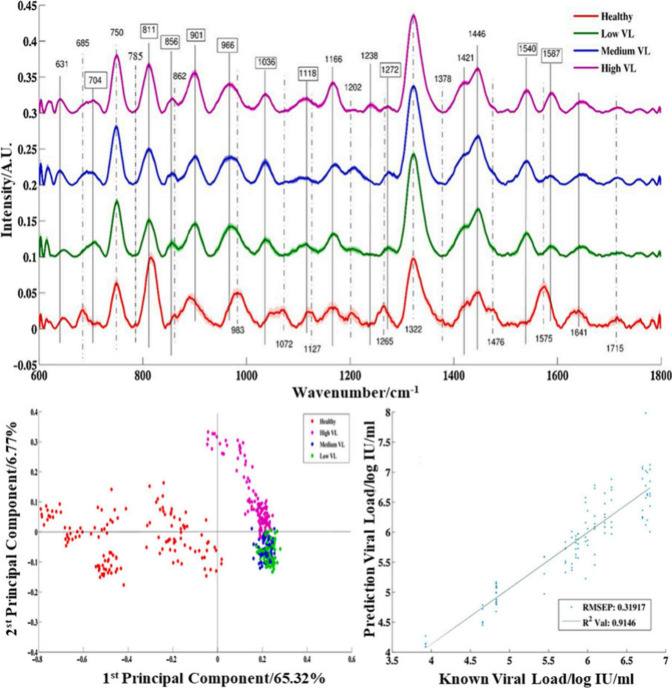
Raman spectra, PCA, and PLSR performance for HCV RNA detection; Reproduced with permission from ref 423. Copyright 2021, Elsevier.

#### 7.2.4 Extraction, Purification, Amplification, and Concentration of Viral Analytes

Following clarification, additional processing is often required to isolate and enrich viral targets for sensitive and reproducible SERS detection. Whole virions are commonly purified using density-gradient ultracentrifugation (e.g., sucrose or iodixanol), which preserves structural integrity while removing host contaminants.^424^ In affinity-based SERS, immunomagnetic capture enables selective enrichment either in solution or directly on plasmonic substrates, reducing background interference.^425,426^ To maintain spectral fidelity, gradient media and buffers must be Raman-silent, and residual proteins or salts should be rigorously removed. Recovery efficiency should be verified using pellet–supernatant analysis or spike–recovery controls to ensure analyte retention throughout processing.^415,419,420^ Viral protein extraction typically employs mild lysis followed by clarification and chromatographic purification (e.g., affinity or ion-exchange).^427,428^ Subsequent buffer exchange or ultrafiltration is essential to eliminate detergents and salts that elevate fluorescence or obscure Raman bands. Preserving conformational epitopes is critical for affinity-based detection; excessive lysis or improper buffer control can cause denaturation and increased background. Functional integrity is commonly validated through immunoreactivity assays or SERS signal stability. Nucleic acid extraction generally relies on chaotropic lysis (e.g., guanidinium isothiocyanate) followed by silica-column or magnetic-bead purification.^429,430^ Residual chaotropic agents or ethanol can generate strong Raman backgrounds, requiring thorough post-elution cleanup. For low-copy viral genomes, nucleic-acid amplification may precede SERS detection; however, unremoved primers, enzymes, or surfactants can distort spectra, making postamplification purification and buffer exchange into Raman-compatible media essential.

The influence of these preparatory steps depends on the SERS detection configuration. In direct SERS, purification and concentration reveal native vibrational fingerprints, but excessive or harsh processing can disrupt structural features and shift characteristic bands. In affinity-based SERS, contaminant removal improves recognition efficiency, yet overlysis or denaturation reduces binding affinity and reproducibility. In labeled SERS, rigorous purification is essential to eliminate unbound tags, surfactants, and buffer residues that generate spurious Raman signals, ensuring quantitative accuracy and reliable multiplexed detection.

#### 7.2.5 Buffer Exchange and Ionic-Strength Control

Buffer exchange into Raman-quiet media is a critical final step to remove background contributions from transport media, lysis buffers, or amplification reagents.^58,99^ Buffer composition also governs NP stability,^431^ hotspot formation,^432^ and probe-target interactions. In colloidal SERS, controlled ionic strength, often in the low millimolar salt range, can promote reproducible nanoparticle aggregation and hotspot formation, whereas excessive salt may cause precipitation, uncontrolled clustering, and spectral variability. However, aggregation should not be viewed as a buffer-driven process alone. Proteins, antibodies, viral antigens, and whole virions can also participate directly in nanoparticle aggregation by adsorbing onto metal surfaces, screening surface charge, forming a protein corona, or bridging multiple particles. For example, protein-induced nanoparticle aggregation has been used as the basis for SERS detection of proteins and viral biomarkers, while antibody- or antigen-mediated aggregation has been exploited in immuno-SERS assays. Therefore, the final aggregation state in biological SERS samples reflects the combined effects of ionic strength, pH, nanoparticle surface chemistry, and analyte concentration. In affinity-based and labeled SERS assays, pH and ionic strength further influence binding kinetics, epitope presentation, tag stability, and nonspecific adsorption.^251,433^ Thus, a well-controlled buffer exchange protocol not only improves SNR but also standardizes the premeasurement equilibrium among analytes, NPs, and detection surfaces. This ensures that the observed spectra accurately reflect the molecular signature of the viral target rather than background interference or inconsistent aggregation behavior.

### 7.3 Integration with Microfluidics

Although off-chip SERS workflows enable sensitive viral detection, their multistep nature remains time-intensive, operator-dependent, and poorly suited for POC or high-throughput use. Microfluidic integration addresses these limitations by miniaturizing and automating sample preparation and detection within closed, programmable platforms. Microfluidic–SERS systems support continuous sample handling with improved reproducibility, reduced contamination risk, and robust performance across diverse clinical matrices.^435,436^ Fully integrated devices typically incorporate sequential modules for sample introduction, on-chip enrichment, SERS interaction, washing and buffer exchange, and optical access for Raman readout (Figure 19).

**Figure 19:**
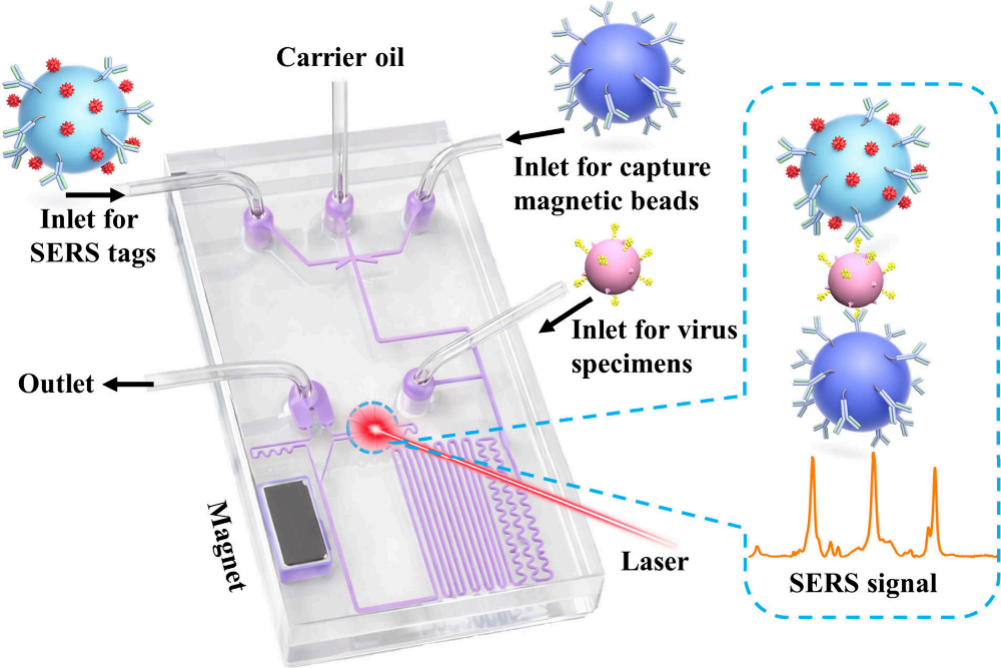
Overview of an integrated microfluidic–SERS platform for automated viral detection. Clinical samples enter through microfluidic inlets and undergo on-chip mixing and enrichment (e.g., magnetic beads, filters, or DEP/DLD structures) to isolate viral targets. Detection occurs in a SERS interaction zone using immobilized plasmonic substrates or confined SERS tags. Automated washing and buffer exchange reduce background interference, and Raman excitation through an optical window enables on-chip spectral readout, supporting reproducible and point-of-care operation. The concept is adapted from ref 434.

Clinical specimens such as saliva, serum, or swab eluates enter through inlet ports and are processed under laminar flow. Passive mixers (e.g., herringbone grooves or serpentine channels) enhance the reagent dispersion and uniform treatment. On-chip enrichment modules including antibody-coated magnetic beads, DEP, nanoporous membranes, and deterministic lateral displacement (DLD) arrays can selectively isolate viral particles from debris and host components, particularly benefiting viscous or heterogeneous samples, where conventional clarification yields variable recovery. Detection regions employ either immobilized plasmonic substrates (substrate-on-chip, SOC) or suspended SERS tags in droplets or flow channels. SOC formats enable direct viral capture on functionalized nanostructures, whereas droplet-based systems confine immunoreactions within controlled microvolumes, improving the binding kinetics and reducing reagent consumption. Assay performance is optimized through the precise control of residence time, flow rate, and temperature. Following capture or labeling, automated washing and buffer exchange remove unbound reagents and background interferents. Raman-compatible buffers are introduced via microvalves or digital microfluidic actuation, while waste is routed to sealed reservoirs to maintain the biosafety. Optical windows fabricated from low autofluorescence materials allow Raman excitation and signal collection using microscope objectives or fiber probes. Some platforms integrate internal reference zones (e.g., immobilized 4-MBA) to enable ratiometric calibration and improve the quantitative accuracy.

Recent demonstrations highlight the clinical potential of microfluidic–SERS systems. Yeh et al. reported a handheld device using Au-decorated, nitrogen-doped carbon nanotube herringbone arrays for label-free virus detection directly from patient swabs, achieving ∼ 70× enrichment, minute-scale analysis, and ∼ 90% specificity (Figure 20A).^120^ Zhang et al. developed a droplet-based microfluidic–SERS platform combining immunomagnetic capture with SERS nanotags, reaching a detection limit of 0.22 PFU/mL with <2% coefficient of variation and <10 min assay time for SARS-CoV-2 (Figure 20B).^437^ Collectively, these studies demonstrate that microfluidic–SERS platforms can surpass conventional ELISA and LFA in speed, sensitivity, and standardization.

**Figure 20:**
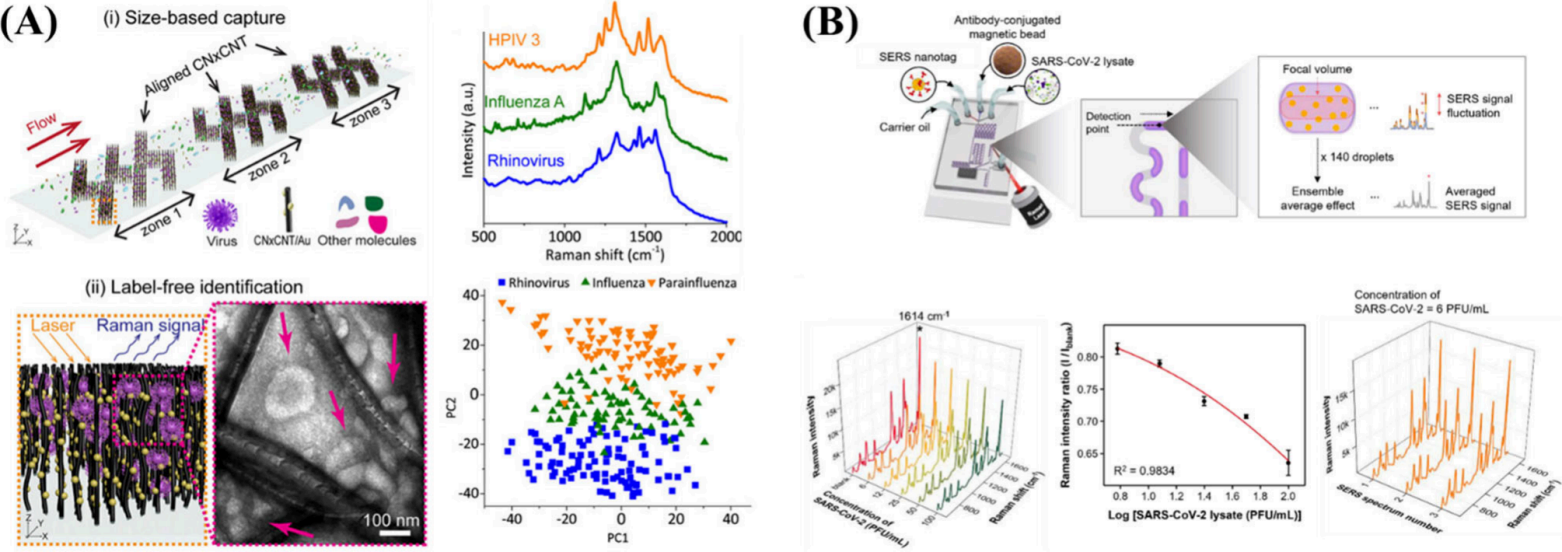
(A) Design and working principle of a handheld microfluidic device for effective virus capture and identification; Reproduced with permission from ref 120. Copyright 2020, National Academy of Sciences under CC-BY 4.0 https://creativecommons.org/licenses/by/4.0/. (B) Detection of SARS-CoV-2 lysate using a SERS-based microdroplet sensor; Reproduced with permission from ref 437. Copyright 2022, Elsevier.

### 7.4 Integration with Lateral Flow Assays

Although microfluidic–SERS platforms provide high precision and automation, their fabrication complexity and infrastructure requirements can limit use in resource-constrained settings. To enable portable, low-cost viral diagnostics, SERS has been effectively integrated into LFAs, a format valued for simplicity, disposability, and POC compatibility. SERS-LFA platforms replace conventional colorimetric reporters with SERS-active nanotags and spectral readout, enabling sensitive, quantitative, and multiplexed detection of viral antigens, antibodies, and nucleic acid reporters in complex matrices such as saliva, serum, and NPS.^438^ This integration directly overcomes the key limitations of traditional LFAs (limited sensitivity, qualitative output, and poor multiplexing) by leveraging the orders-of-magnitude signal enhancement provided by SERS nanotags (Section 6) and objective Raman readout using portable spectrometers.

A typical SERS-LFA retains the standard strip architecture (sample pad, conjugate pad, nitrocellulose membrane with test and control lines, and absorbent pad) but substitutes colorimetric labels with SERS nanotags (Figure 21). Upon sample introduction, target–nanotag complexes migrate and accumulate at the test line, where Raman spectra provide chemically specific and quantitative readout. This modular design supports diverse assay formats, including direct antigen detection, IgM/IgG serology, competitive assays for small viral antigens, and nucleic-acid detection schemes incorporating isothermal amplification or CRISPR-based reporters within the strip.^382^ Multiplexing is readily achieved through spatially separated test lines or spectrally distinct Raman reporters.^54^

**Figure 21:**
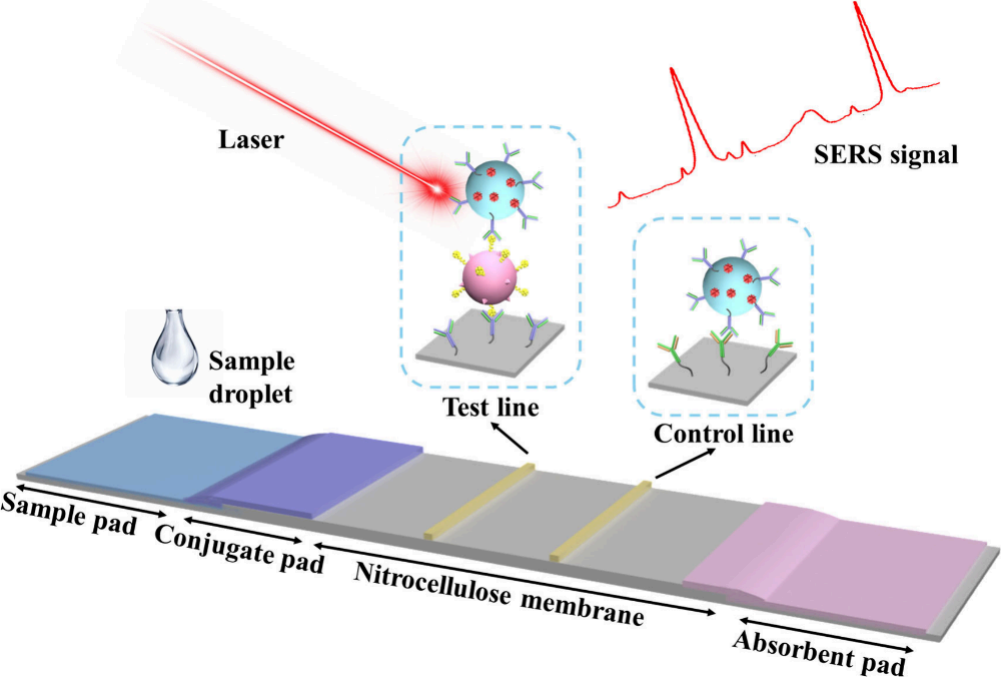
Schematics of SERS-LFAs integrating SERS nanotags into conventional strip architectures. Target–nanotag complexes migrate to the test line, where Raman excitation enables sensitive, quantitative, and multiplexed detection of viral antigens, antibodies, or nucleic acid reporters under point-of-care conditions.

Beyond conventional horizontal strips, SERS-LFA platforms adopt alternative fluidic configurations to address specific analytical needs. Vertical flow assays (VFAs) improve flow rate and reduce background but are less compatible with Raman scanning.^439^ Three-dimensional folded strips and microfluidic paper-based analytical devices (μPADs) enable stepwise operations such as amplification, washing, and buffer exchange.^440^ Magnetically guided LFAs further enhance sensitivity by concentrating targets at defined detection zones.^441^ Each format represents a trade-off among flow control, optical accessibility, and matrix tolerance. Substituting colorimetric labels with SERS probes yields substantial sensitivity gains. Reported limits of detection for viral antigens and antibodies range from low pg/mL to fg/mL levels, corresponding to 100–1000× improvements over conventional LFAs.^57,366,369^ Recent SERS-LFA systems have achieved sub-pg/mL detection,^226,326,382^ with diagnostic sensitivity and specificity comparable to ELISA, while offering faster turnaround, improved reproducibility, and quantitative performance under POC conditions.

## 8 Challenges and Future Directions

Although substantial progress has been made in SERS-based viral detection, from nanostructure design and surface functionalization (Sections 3–6) to specimen preparation and integration with microfluidic and LFA platforms (Section 7), significant challenges still impede widespread clinical translation. Even with optimized preprocessing, many limitations arise from more fundamental factors: the intrinsic principles of different SERS detection strategies, the complexity and variability of biological matrices, and the high dimensionality and overlap/complexity of SERS spectral data. Virus–substrate interactions depend sensitively on nanoscale geometry, molecular orientation, and distance-dependent electromagnetic enhancement, while real specimens introduce competing adsorption, strong background signals, and sample-to-sample variability. Meanwhile, SERS spectra represent overlapping contributions from multiple molecular components, complicating robust interpretation, quantification, and cross-study comparison. Together, these constraints limit sensitivity, reproducibility, and standardization and cannot be fully addressed by sample preparation alone. The following sections therefore examine method-specific challenges and emerging solutions within this broader physical, chemical, and data-analytic framework.

### 8.1 Challenges in Direct Intrinsic Virus Detection

Direct, label-free SERS detection of intact viruses is attractive because of its simplicity, speed, and molecular fingerprinting capability. However, despite substantial experimental progress, its clinical translation remains limited by fundamental and practical challenges related to the signal strength, specificity, reproducibility, and standardization.

A central limitation is the intrinsically weak Raman scattering cross-section of viral biomolecules. Proteins, lipids, and nucleic acids generate much weaker signals than synthetic Raman reporters, resulting in low signal-to-noise ratios even under optimized enhancement conditions.^442,443^ This challenge is exacerbated by geometric mismatch: viruses (∼20–150 nm) are often too large to access sub-10 nm plasmonic gaps, reducing efficient coupling to electromagnetic hotspots.^78^ Although open-hotspot architectures and hierarchical or porous nanostructures can partially mitigate this issue, size-dependent coupling remains a fundamental constraint.

The multilayered architecture of viruses (Figure 7A) further complicates intrinsic detection. Because EM enhancement decays rapidly with distance, only surface components such as envelope proteins and lipids are consistently enhanced, while interior capsid proteins and genomes often lie outside the SERS-active zone.^90^ As a result, measured spectra represent incomplete molecular fingerprints dominated by generic protein and nucleic acid features, limiting diagnostic specificity and complicating peak assignments.

Spectral interpretation is further hindered by the biological and chemical complexity of real specimens. Viral spectra exhibit substantial overlap with signals from background biomolecules including host proteins, metabolites, and salts. The detectability of virus-specific features is highly dependent on viral load, which may be insufficient for reliable detection during early infection stages without amplification. Viral heterogeneity, including strain variation and mutation, introduces additional spectral variability, while buffer composition, pH, ionic strength, and conformational changes can further perturb spectral profiles.

Real-world specimens intensify these challenges. Even with rigorous preprocessing, residual background signals often persist (Section 7). Multivariate and AI-based spectral decomposition approaches, including PCA,^444^ multivariate curve resolution (MCR),^445^ and AI-based unmixing (e.g., autoencoders, regression networks),^202^ can help isolate viral contributions, especially when combined with specimen metadata and substrate information. However, these methods rely on reproducible, high-quality data that are difficult to obtain consistently.

Reproducibility remains a major barrier. Variations in viral adsorption behavior, surface chemistry, orientation, and nanostructure heterogeneity lead to substantial interstudy spectral discrepancies.^446,447^ This variability is clearly illustrated by published data. Figure 22A shows SERS spectra of Influenza A obtained from three different groups. Spectra reported by Yang et al. (ref 40) and Lim et al. (ref 169) are broadly consistent in the 700–1500 cm^–1^ range, whereas the spectrum reported in ref 172 differs considerably. Similarly, Figure 22B presents SARS-CoV-2 spectra from three independent studies. Although some similarities can be observed between ref 40 and ref 137, the profile in ref 172 is entirely different. These discrepancies may arise from differences in substrate design, excitation wavelength, laser power, polarization, detector sensitivity, and signal processing, underscoring the need for standardized fabrication, measurement, and analysis protocols.

**Figure 22:**
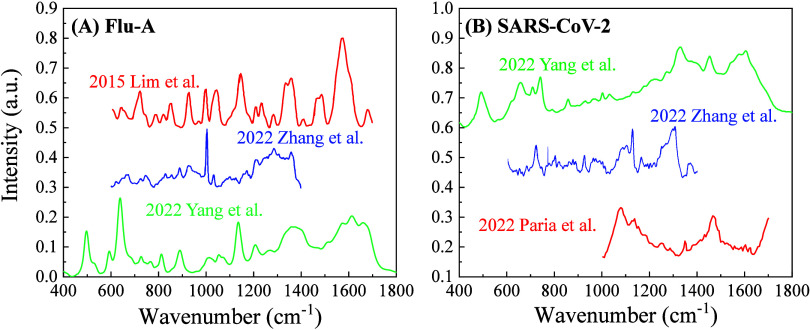
Examples of SERS spectra from different publications for (A) Influenza A: green (adapted with permission from ref 40. Copyright 2022, Elsevier), blue (adapted with permission from ref 172. Copyright 2022, Elsevier), and red (adapted with permission from ref 169. Copyright 2015, American Chemical Society); and (B) SARS-CoV-2: green (adapted with permission from ref 40. Copyright 2022, Elsevier), blue (adapted with permission from ref 172. Copyright 2022, Elsevier), and red (adapted with permission from ref 137. Copyright 2022, American Chemical Society).

In the absence of affinity ligands (e.g., antibodies, aptamers), direct SERS must rely on subtle spectral distinctions to identify targets. This becomes problematic when differentiating closely related viruses or variants at low concentrations.^448^ Chemometric methods such as PCA, HCA, and PLS^449^ and, more recently, CNNs and SVMs^265^ show promise; however, their performance is constrained by limited, noisy, and poorly annotated training sets. Addressing this will require spectral libraries, simulated data augmentation, and better biochemical mapping of peaks to viral structures. Without public, standardized databases of well-annotated viral SERS spectra, including metadata on substrates, viral strains, specimen types, and acquisition conditions, reproducibility remains elusive.^212^ Community-driven databases (e.g., SpectraGuru.org) that incorporate spectral preprocessing, deconvolution tools, and metadata standards will be critical for benchmarking and cross-platform validation.

### 8.2 Challenges in Affinity-Based Detection

Affinity-based SERS sensors improve specificity and robustness by using molecular recognition elements (MREs) to selectively capture viral targets near the plasmonic hotspots. While this strategy effectively suppresses background interference and enhances reproducibility relative to direct detection, it introduces a distinct set of challenges rooted in plasmonic physics, surface chemistry, and biomolecular recognition.

A central limitation lies in the decay of the electromagnetic enhancement with distance from the metal surface. Large capture agents (e.g., antibodies, ACE2 receptors) may position the viral target several nanometers away from the hotspot, diminishing the Raman signal strength and limiting quantitative sensitivity. This is especially problematic for analytes with weak intrinsic Raman cross sections or subtle conformational differences. Compact receptors, such as aptamers, nanobodies, or short peptides, mitigate this issue by reducing steric hindrance and improving hotspot accessibility. Engineering MREs such as monobody scaffolds and synthetic receptor analogues with distinct SERS signatures can also enable ratiometric or differential detection, enhancing target discrimination.^450^

Similar to direct detection, target-specific signals may be masked by background contributions from MREs or the matrix. Binding-induced changes are often minor and are difficult to distinguish from spectral noise. Computational spectral extraction using PCA, neural networks, or regression models can isolate the true analyte spectrum from overlapping components, increasing quantification accuracy and enabling identification in complex biological environments.^451^

Receptor immobilization and orientation represent another major source of variability. Common surface chemistries (e.g., thiol–gold bonding, EDC/NHS coupling) often yield heterogeneous, randomly oriented receptor layers, where active sites may be inaccessible; receptors may also be weakly attached and lost during washing. To overcome these limitations, optimized surface functionalization strategies are essential. Techniques employing PEG spacers,^452^ multivalent linkers,^453,454^ and bioorthogonal click chemistries^455^ can improve receptor orientation, flexibility, and stability, while surface passivation agents such as PEG or BSA help suppress nonspecific adsorption and maintain biorecognition activity under physiological conditions.^456^ Emerging DNA origami-based nanostructures offer a powerful route to achieve precise spatial control over receptor positioning.^457,458^

Affinity sensors are also vulnerable to off-target binding and viral evolution. Antibodies may cross-react with conserved epitopes across viral families, while aptamers can exhibit unintended affinity for nontarget proteins. Viral mutations further threaten diagnostic reliability by altering surface epitopes. Strategies to address these issues include variant-tolerant aptamer libraries,^459^ broadly reactive antibodies,^460,461^ and engineered receptor mimics (e.g., ACE2 derivatives).^462,463^ ML-based spectral pattern recognition can further distinguish true binding events from nonspecific fluctuations.

Reproducibility remains a persistent challenge. Variability in nanostructure fabrication, receptor conjugation efficiency, and hotspot distribution leads to inconsistent receptor density, binding kinetics, and signal output. Environmental factors, such as pH, ionic strength, and competing biomolecules, exacerbate these effects. Addressing these issues requires stringent quality control, standardized substrate production, and optimized receptor-conjugation protocols. Microfluidic or magnetic preenrichment platforms can regulate reaction conditions, reduce batch effects, and improve interlaboratory reproducibility.

Finally, affinity-based SERS performance is strongly influenced by upstream specimen handling. Cleanup and enrichment methods, including centrifugal filtration, magnetic capture, and electrokinetic preconcentration, can significantly improve target availability near hotspots while preserving MRE activity.^264,464,465^ Integrating these into automated SERS–microfluidic systems^466^ offers a promising path toward reproducible, low-LOD, and field-deployable diagnostics.

### 8.3 Challenges in Labeled Detection

Labeled SERS detection offers exceptional sensitivity, quantitative capability, and multiplexing by translating molecular recognition into external Raman readouts. However, these advantages come at the cost of increased system complexity, introducing fundamental challenges in tag reproducibility, nanoscale geometry control, signal stability, and clinical integration.

A primary challenge lies in SERS tag fabrication and conjugation. Tag assembly involves nanoparticle synthesis, reporter loading, probe attachment, and protective encapsulation, each contributing to batch-to-batch variability and inconsistent signal outputs.^467^ Incomplete control over the loading density or surface chemistry can compromise stability and reproducibility. Progress depends on standardized conjugation strategies, such as click chemistry, and robust encapsulation (e.g., silica or polymer shells) that prevent reporter leakage and enable scalable manufacturing.^455^

Assay performance is also constrained by binding efficiency and nanoscale geometry. Incomplete formation of substrate–analyte–tag complexes or steric hindrance among bulky components reduces hotspot formation and weakens enhancement. Because Raman intensity depends critically on interparticle spacing and orientation, effective labeled detection requires precise spatial design to ensure target recognition and signal generation occur within plasmonically active distances. Hybrid nanostructures and kinetic modeling provide promising strategies for optimizing these interactions and improving quantitative reliability.

Signal reliability represents another major limitation. SERS tags face issues such as reporter photobleaching, leaching, or incomplete release in signal-off assays, leading to false negatives.^468^ Hotspot heterogeneity further complicates the quantification. Ratiometric calibration using internal standards and digital SERS approaches, where binary counting replaces intensity measurements, have shown promise in reducing variability and improving quantitation. Wider adoption and standardization of these methods will be critical for clinical applications.^447^

A further limitation is multiplexing capacity. Many commonly used Raman reporters exhibit overlapping spectral features, constraining the number of analytes that can be resolved simultaneously.^298,339^ Expanding multiplexing will require reporters with narrow, nonoverlapping signatures such as isotopically encoded tags, metal–ligand complexes, or spectrally encoded microparticles (MPs), combined with advanced spectral unmixing and digital SERS strategies.

Finally, matrix effects in real biological specimens challenge tag stability and signal fidelity. Variations in pH, ionic strength, and nonspecific adsorption can alter tag performance or obscure true signals. While upstream enrichment and microfluidic integration can mitigate these effects, encapsulated or core–shell tag designs are particularly important for protecting reporters and maintaining long-term stability in complex environments.

### 8.4 Clinical-Scale Variability and Adaptability Challenges

Translating SERS-based virus detection from controlled laboratory studies to real-world clinical diagnostics requires platforms that are robust to both the specimen-level heterogeneity and viral evolution. Unlike purified laboratory samples, patient-derived specimens such as saliva, swabs, blood, stool, and CSF present highly variable biochemical environments that directly impact sensitivity, reproducibility, and diagnostic specificity. At the same time, the inherent rapid mutation of viruses introduces an additional layer of clinical variability as circulating viral strains may differ across patients, populations, and outbreak phases.

Viruses are distributed across diverse biological fluids, with each presenting distinct challenges. Blood and plasma contain abundant proteins, metabolites, and anticoagulants that complicate Raman readout;^264,469^ saliva shows strong inter- and intraindividual variability driven by diet, hydration, and circadian factors; stool contains heterogeneous food, bile, and microbial backgrounds; and CSF, while relatively clean, often contains extremely low viral titers.^401^ Specimen composition can vary substantially across individuals and populations due to age, sex, geographic region, and health status, further complicating standardization.^470^ These specimen-dependent variations are further compounded by viral evolution, as mutations may subtly alter viral protein composition, glycosylation, or structural organization without eliminating the overall biochemical signatures.

Endogenous biomolecules frequently generate strong Raman features that overlap with viral signatures, reducing the diagnostic specificity. Transport and storage media introduce additional complications. Viral transport media, preservatives, and inactivation reagents are essential for biosafety and PCR workflows^471^ but produce strong Raman signals that can mask viral features.^205^ Moreover, many clinical specimens are collected in lysis buffers that disrupt intact virions, shifting detectable targets toward fragmented nucleic acids or proteins and complicating direct SERS detection strategies. Importantly, mutation-induced changes in viral structure or epitope presentation can further degrade the performance of narrowly targeted assays, if not explicitly accounted for.

Low and variable viral loads pose another major challenge, particularly during early infection. Viral shedding differs across specimen types (e.g., saliva vs nasal swabs vs blood) and across patient populations, making it difficult to define universal diagnostic thresholds.^463^ In addition, as discussed in Section 7, storage and transport conditions further contribute to variability: freeze–thaw cycles, prolonged storage, and stabilizing additives can alter viral integrity or potentially damage SERS substrates, degrading enhancement efficiency.

Addressing these challenges requires specimen-aware and evolution-aware strategies, rather than static workflows. Matrix-specific preprocessing such as microfluidic preconcentration for saliva, magnetic capture for blood, or enzymatic pretreatment for stool can improve analyte recovery while suppressing background interference. Beyond sample handling, a central advantage of SERS-based diagnostics lies in ML-driven spectral interpretation. Models trained on diverse, longitudinal clinical spectra can learn invariant biochemical features shared across viral variants, rather than relying on single epitopes or sequence motifs. This enables generalization to previously unseen variants and incremental model updating as new data become available, reducing the need for frequent redesign of probes, primers, or assay hardware.

This adaptability is tightly coupled to the development of shared cloud-based SERS spectral infrastructures. Aggregating validated spectra from confirmed clinical samples across laboratories, specimen types, patient demographics, and viral lineages enables the rapid dissemination of emerging variant signatures, standardized metadata annotation, and cross-laboratory benchmarking. Continuous model retraining on these evolving data sets shifts adaptation from sensing hardware to data sharing and algorithmic updating, effectively transforming viral mutation from a fundamental barrier into a continuously learnable, data-driven problem.

Viewed from a systems-level perspective, mutation resilience in SERS diagnostics emerges from the coordinated integration of broad-spectrum SERS sensing, specimen-aware preprocessing, ML-based interpretation, and shared spectral databases. This ecosystem clarifies where SERS platforms are intrinsically robust, where vulnerabilities remain, and how coordinated data and infrastructure strategies can mitigate these challenges in real-world clinical deployment.

### 8.5 Standardization and Cross-Laboratory Validation

A major barrier to the clinical translation of SERS-based virus detection is the limited reproducibility of results across laboratories. Spectral variability arises not only from biological and matrix effects (Sections 8.1–8.4), but also from the absence of standardized experimental protocols, unified metadata requirements, and benchmarking frameworks. Current studies employ widely different approaches to specimen preparation, substrate fabrication, surface functionalization, spectral acquisition, and data processing. Even modest variations in buffer composition, pH, drying conditions, excitation wavelength, acquisition time, or instrumentation can substantially alter spectral appearance, complicating cross-study comparison and reducing confidence in reported diagnostic performance.

The lack of standardization directly hinders the development of robust spectral libraries and limits the generalizability of ML models trained on narrowly defined data sets. To date, very few interlaboratory or round-robin studies have systematically evaluated how differences in substrates, instrumentation, or sample handling affect key performance metrics such as sensitivity, specificity, reproducibility, and dynamic range.^447^ As a result, most SERS virus detection studies remain at the proof-of-concept stage with limited external validation.

Encouraging precedents from related analytical fields demonstrate that these challenges can be surmounted. In proteomics, community-driven repositories such as ProteomeXchange and PRIDE have enabled cross-laboratory comparability through standardized data formats and metadata requirements.^472^ Metabolomics has adopted interlaboratory ring trials and shared platforms such as MetaboLights to enforce harmonized workflows.^473^ Similarly, Raman and infrared biospectroscopy communities have conducted round-robin validation studies to establish reproducibility standards across instruments and laboratories.^474,475^ To advance SERS-based virus detection from fragmented research efforts to reliable diagnostic platforms, a coordinated community action is required. Key priorities include (1) developing standardized guidelines for specimen preparation, substrate design, excitation conditions, spectral acquisition, and preprocessing; (2) organizing interlaboratory blind studies to identify dominant sources of variability; (3) adopting shared performance benchmarks (e.g., LOD/limit of quantification (LOQ), dynamic range, reproducibility, matrix effects, and stability); (4) establishing open-access spectral libraries with complete experimental metadata; and (5) defining community-accepted standards for reporting ML models, including training data, preprocessing pipelines, validation strategies, and interpretability analyses. Such efforts will be essential to ensure reproducibility, enable fair benchmarking, and ultimately support the regulatory approval and clinical adoption of SERS-based viral diagnostics.

### 8.6 Regulatory, Payment/Coverage, and Clinical Adoption Barriers

Beyond technical performance, successful clinical translation of SERS-based viral diagnostics depends on acceptance within the healthcare ecosystem. New diagnostic modalities face substantial barriers related to regulatory approval, clinical adoption, and reimbursement. Regulatory agencies such as the United States Food and Drug Administration (U.S. FDA) require rigorous demonstration of analytical validity, clinical performance, reproducibility, and robustness across diverse patient populations and specimen types. For SERS platforms, additional challenges arise from the use of complex nanomaterials, spectral databases, and ML-based classification, which complicate standardization, validation, and postapproval modification.

Clinical adoption further depends on integration with existing laboratory workflows and demonstrated advantages over entrenched technologies, such as PCR and ELISA, in terms of turnaround time, cost, or actionable information. Even after regulatory clearance, real-world deployment may remain limited if the test is not covered, funded, or paid for by healthcare systems, public payers, or private insurers. This issue is especially visible in the United States, where diagnostic reimbursement typically depends on coding, coverage, and payment decisions, but analogous health-technology-assessment, funding, and payment barriers exist in other healthcare systems. For point-of-care diagnostics, these barriers can be particularly important because the clinical site performing the test, the payer, and the reimbursement or coverage mechanism may not align easily. Addressing these challenges will require early engagement with clinicians, regulators, and payers; development of standardized reporting and validation frameworks; and clear demonstration of clinical utility and cost-effectiveness. Recognizing and addressing these nontechnical barriers is essential for translating SERS from promising laboratory demonstrations into widely adopted diagnostic tools capable of impacting patient care and public health.

## 9 Conclusion and Outlook

SERS has evolved into a powerful optical platform for viral diagnostics, offering ultrahigh sensitivity, molecular-level fingerprinting, and compatibility with a broad range of assay formats. Over the past two decades, numerous proof-of-concept studies have demonstrated detection of intact viruses, viral proteins, and nucleic acids at extremely low concentrations, sometimes down to single-particle levels. Both direct and indirect strategies, including affinity-based and labeled approaches, have shown substantial potential for rapid detection, multiplexing, and POC deployment. Taken together, these advances position SERS as a compelling complement, in select contexts, a potential alternative to established diagnostic methods such as PCR and ELISA, particularly when rapid, information-rich analysis is needed.

However, comparisons between SERS and established diagnostic technologies must be made cautiously, because these platforms differ in target analyte, amplification mechanism, specimen type, workflow complexity, and intended use. PCR remains the benchmark for nucleic acid detection because enzymatic amplification enables very high analytical sensitivity and sequence specificity. ELISA and lateral-flow assays are clinically established and scalable for antigen or antibody detection but are limited by antibody quality, antigen abundance, cross-reactivity, or delayed serological response. SERS offers complementary strengths, including molecular fingerprinting, narrow spectral features, multiplexing potential, and compatibility with portable Raman systems. Its clinical competitiveness, however, depends on whether comparable sensitivity and specificity can be achieved in real specimens using reproducible substrates, standardized workflows, and validated decision thresholds. A quantitative comparison of representative diagnostic platforms is summarized in Table S10.

Despite these strengths, widespread clinical adoption remains hindered by several fundamental challenges. Direct detection, while attractive for its simplicity, suffers from weak intrinsic viral signatures, a mismatch between viral dimensions and plasmonic hotspots, and significant spectral variability. Affinity-based approaches improve specificity but introduce signal attenuation, cross-reactivity, and variability in bioreceptor immobilization. Labeled detection provides the strongest sensitivity and multiplexing capacity yet depends on robust SERS tag synthesis, high signal stability, and scalable fabrication of spectrally distinct nanotags. Across all detection configurations, additional barriers persist, including matrix complexity in clinical specimens, inconsistent preprocessing pipelines, the absence of standardized spectral libraries, and limited cross-laboratory benchmarking.

Nevertheless, the field is rapidly advancing toward clinically meaningful solutions. Improved nanofabrication has yielded substrates with engineered nanocavities, nanopores, and hierarchical plasmonic structures that more effectively match viral dimensions and enhance reproducibility. Integration with microfluidics, magnetic enrichment, and DEP manipulation now enables efficient preconcentration of viral particles and removal of biofluid contaminants—critical capabilities for handling real clinical samples. At the analytical level, ML/DL methods are transforming spectral interpretation through denoising, peak identification, background removal, classification, and interpretation of overlapping Raman features in multiplexed assays.

Equally important, the field must distinguish between SERS biosensors and SERS diagnostics. Most reported platforms have established biosensing feasibility by detecting viral analytes, distinguishing viral spectra, or achieving high analytical sensitivity under defined conditions. However, a diagnostic is not only a sensitive sensor; it is a decision-making tool embedded in a specific clinical or public health workflow. Therefore, future SERS studies should define the intended use of the test, including the disease context, specimen matrix, end-user, turnaround time requirement, reporting format, acceptable error profile, and downstream action. These requirements will differ for point-of-care triage, laboratory confirmation, viral-load monitoring, multiplexed respiratory panels, home testing, and outbreak or environmental surveillance. Thus, SERS diagnostic development should proceed from the intended clinical or public health decision backward to the assay format, substrate design, sample preparation workflow, spectral analysis pipeline, and validation strategy. This intended use framework provides the conceptual basis for the translational roadmap outlined below. Looking forward, the successful translation of SERS into practical viral diagnostics requires coordinated progress in system integration, standardization, scalability, and regulatory alignment. A realistic pathway from laboratory demonstrations to deployable technologies can be framed through a six-phase translational roadmap (Figure 23):

**Figure 23:**
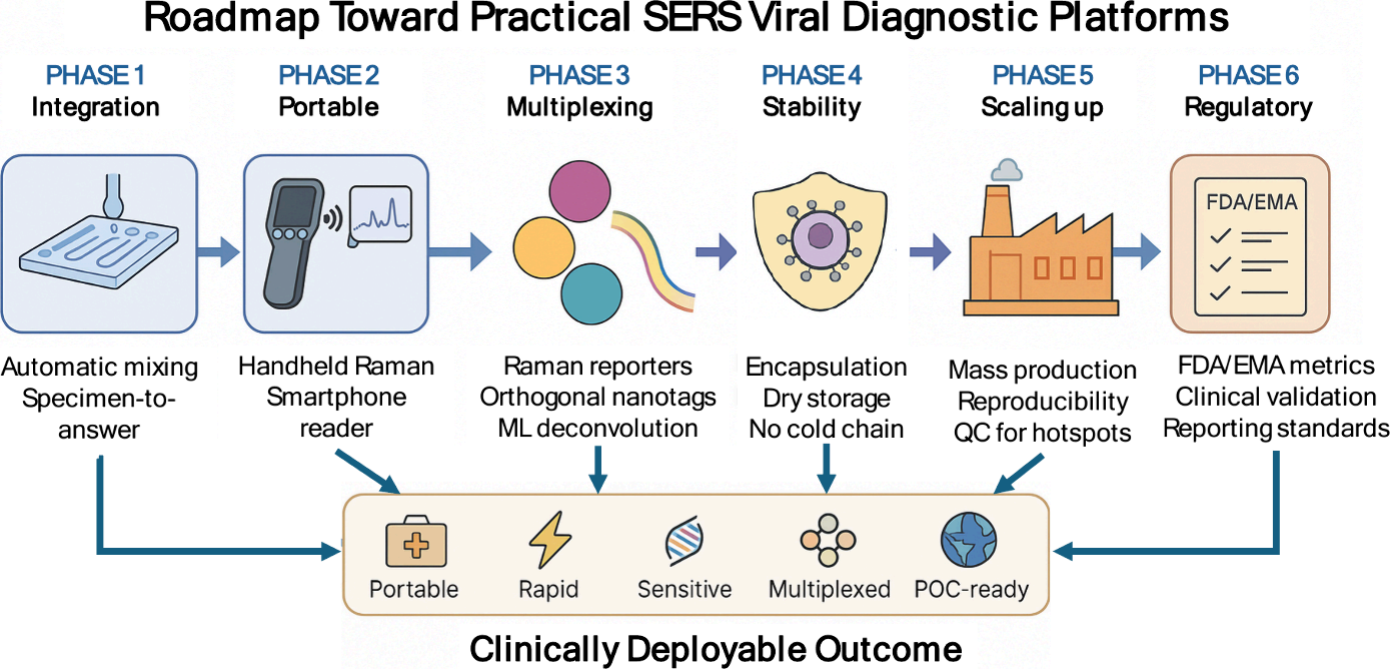
Roadmap toward practical SERS viral diagnostic platforms.

- **Phase 1: Workflow Integration and Automation.** Existing SERS assays often rely on complex, manual procedures such as sample pretreatment, substrate loading, washing, incubation, and spectral collection, all of which introduce operator variability and limit throughput. Transitioning to automated specimen-to-answer workflows is essential. Microfluidic–SERS platforms offer a promising path by integrating sample handling, mixing, enrichment, and detection directly on chip.^476^ These systems reduce human error, improve reproducibility, and make SERS diagnostics more suitable for nonexpert users and decentralized settings.
- **Phase 2: Hardware Miniaturization and POC Deployment**. Despite improvements in portable Raman spectrometers, most viral SERS assays still depend on benchtop instruments that are expensive and immobile. Emerging handheld Raman devices can approach laboratory-grade performance, but they require systematic validation using clinical samples before widespread adoption. Embedding SERS assays into compact, mobile hardware will enable rapid diagnostics in settings such as clinics, emergency rooms, airports, and eventually, home-based monitoring.
- **Phase 3: Multiplexing and High-Information Assays.** Many infectious diseases involve coinfections or require discrimination between closely related viral strains.^206^ Although Raman scattering is intrinsically well suited for multiplexing due to its sharp spectral lines, practical implementation remains hindered by overlapping signatures and the limited number of spectrally distinct Raman reporters. Expanding the reporter library, engineering tags with nonoverlapping signatures, and applying ML-based spectral deconvolution are key steps toward reliable multiplexed viral panels.
- **Phase 4: Stability Engineering and Field Compatibility**. SERS diagnostic components, including SERS tags, antibodies, aptamers, and plasmonic substrates, are often sensitive to temperature, humidity, and photodegradation, reducing shelf life and complicating distribution to low-resource settings. Stability can be improved through strategies such as silica or polymer encapsulation to prevent reporter leakage and photobleaching as well as the development of dry-stored or rehydration-activated devices that eliminate cold-chain requirements.
- **Phase 5: Scalable Manufacturing for Clinical Use**. Many high-performance substrates rely on fabrication techniques (e.g., e-beam lithography, glancing-angle deposition) that are difficult or expensive to scale. Clinical translation demands manufacturing methods compatible with mass production, such as nanoimprint lithography, roll-to-roll processes, or high-yield NP synthesis, while maintaining consistent hotspot density and signal reproducibility across batches.
- **Phase 6: Regulatory Pathway and Clinical Validation**. Achieving regulatory approval requires meeting stringent analytical and clinical performance standards. Platforms must demonstrate stable analytical accuracy (LOD, specificity, reproducibility, linearity, and dynamic range) and undergo extensive validation using real clinical samples across diverse populations and matrices. Establishing standardized reporting frameworks will smooth the regulatory pathway and build trust among clinicians and diagnostic developers. Platforms such as SpectraGuru.org exemplify early community-level efforts in data standardization and shared preprocessing pipelines.

In summary, SERS-based viral detection is at a transformative moment. The scientific foundation is robust with wide-ranging demonstrations of sensitivity, specificity, and versatility across viral targets and assay formats. The essential next steps lie in translation: standardizing protocols, scaling up reproducible substrate fabrication, building annotated and interoperable spectral libraries, and integrating SERS into automated, portable, and regulatory-aligned diagnostic systems. With coordinated advancements across nanotechnology, microfluidics, AI-driven data science, and clinical validation, SERS has the potential to mature from a powerful research tool to a practical, reliable, and globally accessible diagnostic technology. Such platforms would not only support rapid clinical decision-making but also strengthen outbreak surveillance, pandemic preparedness, and global public health infrastructure.

## Supplementary Material



## Notes

A listing of abbreviations and viruses detected, summary tables of direct virus and viral component detection, receptors for affinity-based detection, summary of affinity sensors, labeled detection of intact viruses, viral proteins, antibodies, and nucleic acids, as well as comparison of SERS-based viral detection with conventional viral diagnostic methods (PDF)
