# Supplementary material for: Surface-Enhanced Raman Spectroscopy for Viral Diagnostics: Principles, Strategies, Clinical Challenges, and Future Directions
Source: Chem Rev. 2026 Jul 30;126(15):8694–742. doi: 10.1021/acs.chemrev.6c00201 (PMC13474570; doi:10.1021/acs.chemrev.6c00201)
Supplement: Supplementary file 1 [file cr6c00201_si_001.pdf]

Supporting Information

Surface-Enhanced Raman Spectroscopy for Viral Diagnostics: Principles, Strategies, Clinical Challenges, and Future Directions

Yanjun Yang and Yiping Zhao*

Department of Physics and Astronomy, The University of Georgia, Athens, GA 30602, USA

* Corresponding Author: E-mail: zhaoy@uga.edu

List of Abbreviations

Common analysis and machine learning methods:

(1) Spectral assignment, dimensionality reduction, and unsupervised analysis

- DFT: Density Functional Theory
- PCA: Principal Component Analysis
- HCA: Hierarchical Cluster Analysis
- MCR: Multivariate Curve Resolution

(2) Classical classification methods

- LDA: Linear Discriminant Analysis
- GNB: Gaussian Naive Bayes
- KNN: K-Nearest Neighbors
- LR: Logistic Regression
- SVM: Support Vector Machine
- SVC: Support Vector Classifier
- RF: Random Forest
- DT: Decision Tree
- PLS-DA / PLSDA: Partial Least Squares Discriminant Analysis

(3) Regression and quantification methods

- PLS: Partial Least Squares
- SVR: Support Vector Regression
- MCR: Multivariate Curve Resolution

(4) Deep learning methods

- BPNN / BP neural network: Backpropagation Neural Network
- MLP: Multilayer Perceptron
- CNN: Convolutional Neural Network
- RNN: Recurrent Neural Network

(5) Model interpretation and performance evaluation

- Grad-CAM: Gradient-weighted Class Activation Mapping
- ROC: Receiver Operating Characteristic
- AUC: Area Under the Curve
- RMSE: Root Mean Square Error
- MAE: Mean Absolute Error
- LOOCV: Leave-One-Out Cross-Validation
- R^2: Coefficient of Determination
- ACC: Accuracy
- SEN / Sensitivity: Sensitivity
- SPE / Specificity: Specificity

(6) Analytical performance metrics

- LOD: Limit of Detection
- LOQ: Limit of Quantification
- RSD: Relative Standard Deviation
- CV: Coefficient of Variation
- S/N or SNR: Signal-to-Noise Ratio

SERS substrates and related technologies:

(1) Plasmonic nanostructures

- AgNPs / Ag NPs: Silver Nanoparticles
- AuNPs / Au NPs: Gold Nanoparticles
- AgNRs / Ag NRs: Silver Nanorods
- AuNRs / Au NRs: Gold Nanorods
- AgNWs: Silver Nanowires
- AuNSs: Gold Nanostars
- AgNCs: Silver Nanocubes
- Au@Ag NPs: Gold-core/silver-shell nanoparticles
- Ag@Au NPs: Silver-core/gold-shell nanoparticles

(2) Oxide, semiconductor, and support materials

- SiO_2: Silicon Dioxide
- TiO_2: Titanium Dioxide
- $pZrO_2$: Porous Zirconium Dioxide
- Si: Silicon
- c-Si: Crystalline Silicon
- SnS_2: Tin Disulfide

(3) 2D and carbon-based materials

- CNTs: Carbon Nanotubes
- GO: Graphene Oxide
- rGO: Reduced Graphene Oxide

- SLGO: Single-Layer Graphene Oxide
- Nb_2C MXene: Niobium Carbide MXene
- Ta_2C MXene: Tantalum Carbide MXene

(4) Fabrication and patterning methods

- FIB: Focused Ion Beam
- GLAD: Glancing Angle Deposition
- EBL: Electron-Beam Lithography
- PVD: Physical Vapor Deposition
- CVD: Chemical Vapor Deposition
- ALD: Atomic Layer Deposition
- AAO: Anodic Aluminum Oxide

Raman reporters

(1) Common thiolated/aromatic Raman reporters

- 4-MBA / MBA: 4-Mercaptobenzoic Acid
- 4-MPY: 4-Mercaptopyridine
- 4-ATP: 4-Aminothiophenol
- 4-NBT: 4-nitrobenzenethiol
- DTNB: 5,5′-Dithiobis(2-nitrobenzoic acid); Ellman's reagent
- BPE: 1,2-Bis(4-pyridyl)ethylene
- BDMT: 1,4-Benzenedimethanethiol

(2) Dye-based Raman reporters

- MGITC: Malachite Green Isothiocyanate
- NBA: Nile Blue A
- Cy3: Cyanine 3 dye
- Cy5: Cyanine 5 dye
- ROX: Carboxy-X-rhodamine
- TAMRA: Carboxytetramethylrhodamine
- R6G: Rhodamine 6G
- CV: Crystal Violet
- MB: Methylene Blue
- HITC: 1,1′,3,3,3′,3′-Hexamethylindotricarbocyanine iodide

(3) Enzyme/colorimetric reaction products used as SERS signals

- TMB: 3,3′,5,5′-Tetramethylbenzidine; oxidized TMB can be used as a Raman reporter
- NBT: Nitroblue Tetrazolium; often used in enzyme-mediated colorimetric/precipitation reactions

Detection methods and amplification methods

(1) Immunoassay and rapid diagnostic formats

- LFA: Lateral Flow Assay

- LFIA: Lateral Flow Immunoassay
- ELISA: Enzyme-Linked Immunosorbent Assay
- CLIA: Chemiluminescence Immunoassay
- FIA: Fluorescence Immunoassay
- ECLIA: Electrochemiluminescence Immunoassay
- ICA: Immunochromatographic Assay

(2) PCR-based nucleic acid amplification
- PCR: Polymerase Chain Reaction
- qPCR: Quantitative Polymerase Chain Reaction
- RT-PCR: Reverse Transcription Polymerase Chain Reaction
- RT-qPCR: Reverse Transcription Quantitative Polymerase Chain Reaction
- dPCR: Digital Polymerase Chain Reaction
- ddPCR: Droplet Digital Polymerase Chain Reaction

(3) Isothermal nucleic acid amplification
- LAMP: Loop-Mediated Isothermal Amplification
- RT-LAMP: Reverse Transcription Loop-Mediated Isothermal Amplification
- RPA: Recombinase Polymerase Amplification
- RT-RPA: Reverse Transcription Recombinase Polymerase Amplification
- NASBA: Nucleic Acid Sequence-Based Amplification
- HDA: Helicase-Dependent Amplification
- SDA: Strand Displacement Amplification
- RCA: Rolling Circle Amplification
- ERA: Enzymatic Recombinase Amplification

(4) Signal amplification based on DNA nanotechnology
- HCR: Hybridization Chain Reaction
- CHA: Catalytic Hairpin Assembly
- LCHA: Localized Catalytic Hairpin Assembly
- EXPAR: Exponential Amplification Reaction
- TMSD: Toehold-Mediated Strand Displacement

(5) CRISPR-based detection
- CRISPR-Cas: Clustered Regularly Interspaced Short Palindromic Repeats–CRISPR-associated system
- Cas12a: CRISPR-associated protein 12a, commonly used for DNA-target detection
- Cas13a: CRISPR-associated protein 13a, commonly used for RNA-target detection

Buffers, media, biological reagents, and assay materials

(1) Cell culture media, buffers, and sample media
- DMEM: Dulbecco’s Modified Eagle Medium
- RPMI / RPMI 1640: Roswell Park Memorial Institute Medium
- DPBS: Dulbecco’s Phosphate-Buffered Saline

- PBS: Phosphate-Buffered Saline
- VTM: Viral Transport Medium
- FBS: Fetal Bovine Serum

(2) Immunological receptors and viral targets

- IgG: Immunoglobulin G
- IgM: Immunoglobulin M
- mAb: Monoclonal antibody
- Ab: Antibody
- ACE2: Angiotensin-Converting Enzyme 2
- SARS-CoV-2 RBD: Severe Acute Respiratory Syndrome Coronavirus 2 Receptor-Binding Domain
- S protein / Spike: SARS-CoV-2 spike protein
- N protein / NP: Nucleocapsid protein

(3) Surface functionalization and coupling reagents

- EDC: 1-Ethyl-3-(3-dimethylaminopropyl)carbodiimide
- NHS: N-Hydroxysuccinimide
- MCH: 6-Mercapto-1-hexanol
- BSA: Bovine Serum Albumin
- SA: Streptavidin
- Biotin: Biotin, commonly used in streptavidin–biotin binding systems

(4) Virus quantification units

- $TCID_{50}$: 50% Tissue Culture Infectious Dose
- PFU: Plaque-Forming Unit
- CFU: Colony-Forming Unit
- FFU/mL: Focus-forming units per milliliter
- HAU/mL: Hemagglutination units per milliliter
- copies/mL: Genome copies per milliliter
- IU: International Units

(5) Assay materials and separation methods

- SDS-PAGE: Sodium Dodecyl Sulfate-Polyacrylamide Gel Electrophoresis
- NC membrane: Nitrocellulose membrane
- PVDF membrane: Polyvinylidene Fluoride membrane
- T-line: Test Line
- C-line: Control Line

Table S1 A list of viruses, their abbreviations, strains/variants, structure, gene, and size, used for SERS detection in literature.

Full Name	Abbreviation	Strains	Structure	Gene	Size (nm)	Host
Adenovirus	AD	Mad-1<br>3, 5, 7	Nonenveloped	DNA	90-100	Animal/Human
Avian influenza virus	AIV	H5N1, H5N2<br>H5N3	Enveloped	RNA(-)	80-120	Animal
Canine distemper virus	CDV		Enveloped	RNA(-)	150-300	Animal
Canine Parvovirus	CPV		Nonenveloped	DNA	18-26	Animal
Coronavirus	CoV	MHV, NL63,<br>229E, OC43	Enveloped	RNA(+)	100	Human/Animal
Coxsackievirus	CAV	B3<br>3Cv3	Nonenveloped	RNA(+)	22-30	Human
Dengue virus	DENV		Enveloped	RNA(+)	40-60	Human
Encephalomyocarditis virus	EMCV		Nonenveloped	RNA(+)	30	Human/Animal/Plant
Enterovirus	EV		Nonenveloped	RNA(+)	15-30	Human/Animal
Hepatitis A virus	HAV		Nonenveloped	RNA	27-29	Human/Animal
Human immunodeficiency virus	HIV	X4<br>R5	Enveloped	RNA(+)	100	Human/Animal
Human papillomavirus	HPV	HPV-16, HPV-18	Nonenveloped	DNA	50-60	Human
Infectious Bronchitis Virus			Enveloped	RNA(+)	120	Animal
Influenza A virus	IAV	H3N2, H1N1	Enveloped	RNA(-)	80-120	Human/Animal
Influenza B virus	IBV		Enveloped	RNA(-)	80-120	Human
Marburg virus	MVD		Enveloped	RNA(-)	80 × 14,000	Human/Animal/Plant
Metapneumovirus	hHMPV	A, B	Enveloped	RNA(-)	150-600	Human/Animal
Myxoma virus	MYXV		Enveloped	DNA	250×300×200	Animal
Norovirus	NoV		Nonenveloped	RNA(+)	23-40	Human/Animal/Plant
Poliovirus	PV	1M, 1S	Nonenveloped	RNA(+)	25-30	Human
Porcine circovirus	PCV	2	Nonenveloped	RNA	17	Animal/Human
Pseudorabies virus	PRV		Enveloped	DNA	200-250	Animal
Reovirus	REV		Nonenveloped	RNA	60-80	Human/Animal/Plant
Respiratory syncytial virus	RSV	A2, B1	Enveloped	RNA(-)	150-300	Human/Animal
Rhinovirus	RV	4	Nonenveloped	RNA(+)	15-30	Human/Animal
Rift Valley fever virus	RVFV		Enveloped	RNA(+)	90-110	Human/Animal/Plant
Rotavirus	ROV	Simian rotavirus SA11	Nonenveloped	RNA(+)	70	Human/Animal
SARS-CoV-2	CoV2	Ancestral isolate WA1/2020; Alpha variant B.1.1.7; Beta variant B.1.351; Gamma variant P.1; Delta variant B.1.617.2; Omicron variant B.1.1.529; Omicron sublineages BA.1, BA.2, BA.5, XBB, JN.1, KP.3.1.1, LP.8.1, NB.1.8.1, and XFG.	Enveloped	RNA(+)	60-140	Human/Animal
Sendai virus	MPV		Enveloped	RNA(-)	150-200	Animal

(Murine parainfluenza virus)						
Tabacco Mosaic virus	TMV		Nonenveloped	RNA(+)	18×300	Plant
Turnip Mosaic virus	TMV		Nonenveloped	RNA(+)	720	Plant
Turnip yellow mosaic Virus	TYMV		Nonenveloped	RNA(+)	30	Plant
Vesicular stomatitis virus	VSV		Enveloped	RNA(-)	70×200	Animal
West Nile virus	WNV		Enveloped	RNA(+)	50	Animal/Human
Zika virus	ZIKV		Enveloped	RNA(+)	50	Animal & human

Table S2. Summary of direct virus detection.

	Target virus	SERS substrate	Spectral analysis method	Characterization	LOD	Medium	Ref.
**Thin films**	Adenovirus<br>Rhinovirus<br>HIV<br>RSV<br>Flu A HKx31 (H3N2)<br>Flu A WSN/33 (H1N1)<br>Flu A PR/8 (H1N1)	AgNR	Calibration curve	Spectral peak identification; Quantification	10^2 PFU/mL for RSV	Allantoic fluid<br>Cell lysate<br>Water	1
	4 RSV strains	AgNR	PCA, HCA	Classification		Water	2
	RSV<br>HIV<br>Rotavirus<br>Mycoplasma	AgNR	PCA, HCA	Classification		DMEM<br>RPMI<br>Water	3
	Avian influenza H5N2	AgNR	PCA, HCA	Classification		Allantoic fluid	4
	RSV<br>Coxsackievirus B3	Ag coated poly(chloro-*p*-xylylene) film		Spectral peak identification		NaCl, tris-HCl, $MgSO_4$ and sucrose	5
	8 Rotavirus strains: F45, RV3, RV4, RV5, S2, ST-3, Wa, YO	AgNR	PLS-DA, PLS	Classification; Quantification	1.4×10^4 FFU/mL	MA104 cell lysate	6
	Norovirus MNV4;<br>Adenovirus Mad-1;<br>Parvovirus MVMp;<br>Simian rotavirus SA11;<br>Coronavirus MHV;<br>Sendai virus Sendai;<br>Herpersvirus strain Smith MSGV	Commercial Klarite™ gold SERS-active substrate	Soft independent modeling ofclass analogy (SIMCA); PCA	Spectral peak identification; Classification	10^2 PFU/mL	PBS	7

	Encephalomyocarditis virus (EMCV); Adenovirus; Flu A H1N1	Inverted triangular Au nano-cavities array		Spectral peak identification; Quantification	10^6 PFU/mL for EMCV & Adenovirus 10^4 PFU/mL for Flu A	PBS	8
	EMCV Adenovirus 5 Flu A H1N1 Flu A H3N2 Flu A H2N2	FIB-fabricated hexagon-like Au nanorod array		Spectral peak identification		PBS	9
	Avian influenza virus (AVI) H5N1 AVI H5N2 AVI H5N3	AgNR	PLS-DA PCA	Spectral peak identification; Classification		Allantoic fluid	10
	Porcine circovirus 2 (PCV2); Porcine pseudorabies virus (PRV); AVI H5N1	Sputter coated Ag nanoflowers	PCA	Spectral peak identification; Classification; Quantification	10^3 PFU/mL for PCV2	Water	11
	Rhinovirus type 4; Poliovirus type 1 Mahoney (PV1M); Poliovirus type 1 Sabin (PV1S)	Electrochemical deposition Ag nanoplates		Spectral peak identification			12
	PCV2; Porcine parvovirus (PPV); porcine pseudorabies virus (PRV)	AgNP decorated porous carbon films	PCA	Spectral peak identification; Classification	10^7 copy/mL	Ultrapure water	13
	Myxoma virus (MYXV); Canine Distemper virus (CDV); Tobacco mosaic virus (TMV); Potato virus X (PVX)	Porous/rough Ag nanofilm on mica fabricated by EBPVD	PCA-LDA	Spectral peak identification; Classification		tris-HCl solution (pH 8.0)	14
	Wild-type poliovirus Mahoney	Ag-film-covered Au nanoparticle cluster-in-bowl array		Spectral peak identification			15
	Adenovirus 5; Coxsackievirus type 3Cv3	Gold hollow nanocones in microbowls array		Spectral peak identification		PBS	16
	SARS-CoV-2 Flu H1N1 A Marburg Zika virus	Nanoimprinted Ag/SiO_2 multilayered pillars	PCA, RF	Spectral peak identification Classification		Saliva Treated by radiation & heat Cell lysate	17
	SARS-CoV-2 S pseudovirus; Vesicular stomatitis virus G (VSV-G) pseudo-type lentivirus	Gold nanocavities Au NPs/$pZrO_2$ nanobowls		Spectral peak identification; Inactivation effect		Pseudovirus suspension	18
	13 respiratory viruses	SiO_2 coated AgNR	SVM, SVR	Spectral peak identification; Classification;	~ 190 PFU/mL	DMEM; Saliva	19

				Quantitation			
	SARS-CoV-2	Au-TiO_2 nanocomposite SERS chip integrated on a face mask	Ablation-assisted autoencoder; logistic regression for quantitative classification	Spectral peak identification; Classification; Quantitation	10 PFU/mL	Artificial respiratory aerosols	20
	SARS-CoV-2	Ag island film	PLS-DA PCA-LDA SVMC	Spectral peak identification; Classification		Saliva; nasopharyngeal swabs	21
	HIV-1 X4/R5/dual-tropic strains	GLAD-fabricated Ag nanorods; two-arm Ag nanorods; Au-sputtered Ag nanorods	Calibration curve PCA	Spectral peak identification; Classification; Quantitation	~ 100-200 copies/mL	Human plasma; HIV-1 strains spiked in DEPC-treated water/plasma for tropism study	22
	Avian influenza A viruses, H5N2/H7N2; reovirus; clinical respiratory viruses including rhinovirus, influenza A, and HPIV-3	VIRRION microfluidic chip with aligned nitrogen-doped carbon nanotube arrays decorated with Au nanoparticles	PCA	Classification	~10^2 EID_{50}/mL for avian influenza	Virus culture supernatant; nasopharyngeal swab transport medium / clinical respiratory samples	23
	14 RNA viruses, including human respiratory viruses, avian viruses, and enteroviruses	AuNPs on nitrogen-doped carbon nanotube array	1D-CNN; XGBoost comparison; full-gradient feature-importance analysis	Spectral peak identification; Classification	No universal LOD; influenza dilution test detected to ~10^5 dilution, corresponding to ~1–2 infectious viruses and ~142–227 RNA copies per 10 µL	Propagated virus samples/virus cultures	24
	Influenza A	Dendritic Ag@c-Si nanostructure	PCA SVM	Spectral peak identification; Classification		STE buffer (NaCl, Tris, and EDTA with pH = 8.0)	25
	SARS-CoV-2	100 nm Ag coated Si nanostructures	PCA GNB RF SVC LR	Spectral peak identification; Classification		Saliva and nasopharyngeal swabs	26
	Dengue virus	Ag nanorod SERS chip fabricated by GLAD sputtering on Si substrate	PCA, LDA, LR, PCA-LDA, MLP, SVM, DT, RF; LOOCV; ROC/AUC analysis	Spectral peak identification; Classification		Human plasma from pediatric patients	27

	SARS-CoV-2 variants: WT, Kappa, Delta, Omicron; Omicron subvariants BA.1, BA.2, BA.5, XBB	Ag nanorod arrays fabricated by GLAD	PCA; SVM and directional long short-term memory network (BiLSTM) classification; SHAP interpretation	Spectral peak identification; Classification	~100 PFU/mL	Virus isolates and clinical nasopharyngeal swab samples	28
	Influenza A, RSV, human rhinovirus, SARS-CoV-2 Omicron, SARS-CoV-2 Delta	Ag nano-sculptured thin film on Si, fabricated by GLAD	RF, SVM, LR, KNN, MLP	Classification		Clinical nasal/nasopharyngeal samples in viral transport medium (VTM)	29
	Influenza A, Influenza B, RSV, SARS-CoV-2, and SARS-CoV-2 variants/sublineages	3D Au plasmonic nanopillar substrate	1D-CNN classification; Grad-CAM interpretation; PCA comparison	Spectral peak identification; Classification	~3 PFU/mL	DMEM; universal transport media	30
	SARS-CoV-2 BA.2 variant	Au NPs/pZrO_2 and Au NPs/fZrO_2	Characteristic peak/ROI extraction; ensemble subspace kNN	Spectral peak identification; Classification	10^5 copies/mL	Clinical nasopharyngeal and throat swabs in UTM	31
	SARS-CoV-2, RSV, influenza A, and influenza B	3D plasmonic Au-PS SERS chip; 78 nm AuNPs assembled on ordered PS microsphere array, combined with acoustic droplet enrichment	PCA–LDA; ensemble ML model combining autoencoder, DNN, and CNN	Spectral peak identification; Classification; Quantification	5×10^2 PFU/mL	SDS-PAGE buffer; nasal swab samples	32
**Colloid NPs**	Turnip yellow mosaic Virus (TYMV)	Au NP (29 nm)	PCA-LDA	Spectral peak identification; Classification		Sodium phosphate buffer	33
	Pseudotyped viruses HIV-1 Two Flu A H1N1 strains	Au NP (80 nm)		Spectral peak identification			34
	SARS-CoV-2 Human adenovirus type 7 Flu A H1N1	Clustered silver NPs (37 nm)	LDA	Spectral peak identification; Classification; Quantitation	10 PFU/test	Saliva, Serum	35
	SARS-CoV-2 Human adenovirus 3 Flu A H1N1	Silver NPs clusters with acetonitrile	PCA	Spectral peak identification; Classification; Quantitation	100 copies/test (PFU/test)	PBS; artificial saliva; fetal bovine serum	36
	Monkeypox virus (MPXV) Human papillomavirus (HPV)	Silver NPs (29 & 40 nm)	PCA	Spectral peak identification; Classification; Quantitation	100 copies/mL	Fetal bovine serum for MPXV; artificial vaginal discharge for HPV	37

	H1N1, influenza B virus (IBV), respiratory syncytial virus (RSV), human adenovirus type 5 (HAdV); Monkeypox virus (MPXV)	Self-assembled plasmonic magnifier based on Ag NPs/C12 DNA/Ca^{2+} virus-triggered hotspot formation	PCA, LDA, PLS-DA, t-SNE, decision tree, and SVM classification	Spectral peak identification; Classification; Quantitation	RSV: 2×10^{-5} copies/mL; H1N1 and HAdV: 5×10^{-5} copies/mL; IBV: 5×10^{-4} copies/mL	Virus standards; artificial saliva; fetal bovine serum/serum	38
	Influenza A and Influenza B	AgNP colloids	PCA-LDA SVM	Classification		Clinical nasal swab/nasal fluid in saline; heat-inactivated samples	39
	SARS-CoV-2 SARS-CoV MERS-CoV	AuNPs (50 nm)	PCA SVM	Classification		Saliva	40
**Others**	SARS-CoV-2	SnS_2 microspheres	PCA SVM	Classification			41
	SARS-CoV-2 RSV Flu-B CoV OC43	Si nanostructure made by ultrashort pulsed laser	PCA; decision tree; t-SNE; linear regression	Spectral peak identification; Classification; Quantitation	50 viral particle/mL	Human saliva	42

Table S3. The summary of direct detection of viral components.

Target Analyte	Virus	SERS Substrate	Spectra analysis method	Sensor performance	LOD	Ref.
Virus coat protein	Turnip mosaic virus, TuMV	AgNP	Peak identification		~ 3 µg	43
Spike and N protein	SARS-CoV-2	Au–Cu nanostars on few-layer MoS_2	Peak identification Gaussian/Hartree–Fock simulation		8.89 × 10^{-9} M for S protein	44
Spike protein	SARS-CoV-2	Self-assembly AuNP (40 nm) monolayer on Si	DFT<br>RNN	Accuracy: 87.7%<br>Sensitivity: 83.3%<br>Specificity: 92.5%	< 1 ppm	45
Spike protein	SARS-CoV-2	Nb_2C and Ta_2C MXenes<br>Au NPs	Peak identification<br>DFT		5×10^{-9} M	46
Spike protein	SARS-CoV-2	SnS_2 microspheres	PCA		10^{-14} mol/L	41
NS1 protein	Dengue virus	Plasma-engineered Ag nanooctapods	Peak identification	Serotype differentiation of DENV1 and DENV4 NS1 proteins	0.5 µg/mL for DENV1; 5 ng/mL for DENV4	47
Spike protein; IgG antibody	SARS-CoV-2	Metallic H-shaped nano-aperture array on Au film	Peak identification; PCA		0.85 ng/mL for IgG	48
Spike protein	SARS-CoV-2 Wild-type; Omicron; Delta	Au nanostars aggregated with $CuSO_4 \cdot 5H_2O$	Peak identification; PCA; Quantitation	Linear range: 1.0 × 10^{-7} - 1.0 × 10^{-3} g/mL for Wild-type, Omicron, and Delta	~10^{8} g/mL	49
RNA	SARS-CoV-2	SnS_2 microspheres	PCA		10^{4} copies/mL	41
RNA	SARS-CoV-2 Omicron variant	Ag dendrites	Peak identification			50

Table S4. Summary of receptors for different viruses and viral biomarkers.

Virus	Receptor	Ref.	Antibody	Ref.	Aptamer (5'-3')	Targets (Affinity K_d)	Ref.
Human coronavirus OC43 (CoV OC43)	N-acetyl-9-*O*-acetylneuraminic acid	51	IgG, antibody that reacts to S protein	52			
Human coronavirus HKU1 (CoV HKU1)	9-*O*-acetylneuraminic acid	53	IgG	52			
Human coronavirus 229E (CoV 229E)	Aminopeptidase N (hAPN [CD13])	54	IgG, antibody that reacts to S protein	52			
Human coronavirus NL63 (CoV NL63)	ACE2	51	IgG, antibody that reacts to S protein	52			
Middle East respiratory syndrome-related coronavirus (MERS-CoV)	Dipeptidyl peptidase-4	51	IgG	55			
Severe acute respiratory syndrome coronavirus (SARS-CoV)	ACE2	56			GCA ATG GTA CGG TAC TTC CGG ATG CGG AAA CTG GCT AAT TGG TGA GGC TGG GGC GGT CGT GCA GCA AAA GTG CAC GCT ACT TTG CTA A	Nucleocapsid protein (4.93 ± 0.30 nM)	57
					GGG AGA GCG GAA GCG UGC UGG GCC UGU CGG UUC GCU GUC UUG CUA CGU UAC GUU ACA CGG UUG GCA UAA CCC AGA GGU CGA UGG AUC CCC CC	Nucleocapsid protein (1.65 ± 0.41)	58
Severe acute respiratory syndrome coronavirus 2 (SARS-CoV-2)	ACE2	59	IgM, IgG	60	TGG GAG CCT GGG ACA TAG TGG GGA AAG AGG GGA AGA GTG GGT CT	Envelop protein (2 nM)	61
					CAG CAC CGA CCT TGT GCT TTG GGA GTG CTG GTC CAA GGG CGT TAA TGG ACA	Receptor binding domain (RBD) (5.8 ± 0.8 nM)	62
					CAG CAC CGA CCT TGT GCT TTG GGA GTG CTG GTC CAA GGG CGT TAA TGG ACA	S1 subunit (2.7 ± 0.2 nM)	63
					TCG CTC TTT CCG CTT CTT CGC GGT CAT TGT GCA TCC TGA CTG ACC CTA AGG TGC GAA CAT CGC CCG CGT AAG TCC GTG TGT	N-terminal domain (80 nM)	64

					GCG AA		
					GCT GGA TGT CGC TTA CGA CAA TAT TCC TTA GGG GCA CCG CTA CAT TGA CAC ATC CAG C	Nucleocapsid (0.49 ± 0.05 nM)	23
					GCT GGA TGT CAC CGG ATT GTC GGA CAT CGG ATT GTC TGA GTC ATA TGA CAC ATC CAG C	Nucleocapsid (0.70 ± 0.06 nM)	
Hepatitis B virus	Sodium taurocholate cotransporting polypeptide (NTCP)	65	Anti-HBs	66	GGG AAT TCG AGC TCG GTA CCG GCA CAA GCA TAT GGA CTC CTC TGA ACC TAC GAT GTA GTA CCT GCA GGC ATG CAA GCT TGG	HBsAg	67
					TTT TTT GGT CAG ATG AGG CTT GGT GAT CGT GCC CAG GCC ATA TGA GCA AGG AAC CCC TAT GCG TGC T	HBeAg (1.2 nM)	68
					AGC AGC ACA GAG GTC AGA TGA GGC CTG GTG ATC GTG CCC AGG CCA TAT GAG CAA GGA ACC CCT ATG CGT GCT ACC GTG AA	HBcAg (0.2 nM)	69
Hepatitis C Virus	CD81 tetraspanin, scavenger receptor class B type I (SR-BI), mannose-binding lectins DC-SIGN and L-SIGN, low-density lipoprotein receptor, heparan sulphate proteoglycans and the asialoglycoprotein receptor.	70			GCG GAA TTC TAA TAC GAC TCA CTA TAG GGA ACA GTC CGA GCC GAA TGA GGA ATA ATC TAG CTC CTT CGC TGA GGG TCA ATG CGT CAT A	Envelope Glycoprotein E2 (1.05 nM)	71
Influenza virus A (H1N1)	Sialic acid-containing molecules	72	Anti-hemagglutinin	73	TAC TGC ACA CGA CAC CGA CTG TCA CCA TCA CCT CGG CGC AGT GCT GGA ACT CCC CGA CTG C		74
H5N1					GTG TGC ATG GAT AGC ACG TAA CGG TGT AGT AGA TAC GTG CGG GTA GGA AGA AAG GGA AAT AGT TGT CCT GTT GTT GCC ATG TGT ATG TGG G	HA (4.65 nM)	75
Influenza B	Sialic acid-containing molecules				ATT ATG GCG TAT TGC AGC GTT CTG GTT GGT GGT GGG CAG GTG GTG GTA CTG CGC TGC AGC TTG TTG GTG AGG TAA CGG CT	Nucleoprotein (0.97 ± 0.6 nM)	76
Avian influenza					CGT ACG GTC GAC GCT AGC TAA CGG TGT	Intact virus	77

(H5N1)					GGC CCG GGG GTA CAG CGC ACT CAC GTG GAG CTC GGA TCC	particle (7×10^4 EID_{50}/mL)	
Avian influenza (H5N2)					CGT ACG GAA TTC GCT AGC TGA TGG TGT GGC GGG GGG CGG CCT GGG GCG GGC CGC CGA TGG GAT CCG AGC TCC ACG TG	Whole virus particle (6.913 $\times10^5$ EID_{50}/mL)	78
Human papillomavirus (HPV)	Heparan sulfate proteogylcans (HSPGs)	79	Anti-HPV		GGG AAC GGG AAC AAA AGC UGC ACA GGU UAC CCC CGC UUG GGU CUC CCU AUA GUG AGU CGU AUU ATT TTT	L1 capsid (0.05 pM)	80
					GGG AGG ACG AUG CGG AAG CAT CAA GGG TGA TCG TTT GAC CCT CCC CAG ACG ACU CGC CCG A	E7 protein	81
HIV	CD4 receptor and a coreceptor (either CCR5 or CXCR4)	82	Anti-HIV		ACG AAG CUU GAU CCC GUU UGC CGG UCG AUC GCU UCG A	Tat protein (120 ± 13 pM)	83

Table S5. Summary of affinity sensors for SERS-based virus detection.

Target Viruses	SERS substrate	Receptor	Receptor immobilization	Peak identification	Quantification	Spectral analysis methods	Media	LOD	Other sensor performance	Ref.
**Intact virus**										
RSV	AgNR	IgG	Direct	Yes	No		Water			84
ROV	Plasmonic-magnetic hybrid 3D graphene oxide architecture	Antibody	Amine groups	Yes	No		Water			85
DENV<br>WNV	Au nanoparticle	Antiflavivirus 4G2 antibody	EDC and NHS	Yes	Yes	Baseline removal<br>Spectral smoothing	PBS	10 PFU/mL	High selectivity against CHIKV	86
HAV	Au inverted pyramidal nanohole array	Monoclonal anti-HAV antibody IgG3HepA	physical adsorption & BSA blocking	Yes	No		PBS	1000 PFU/mL or 13 pg/mL		87
SARS-CoV-2	Au coated triangular nanocavities	SARS-CoV-2 spike antibody	electrostatic interactions	Yes	Yes		PBS<br>Cell culture	88 PFU/mL		88
EV71	Au nanostar	Recombinant scavenger receptor class B, member 2 (SCARB2) proteins	Electrostatic and hydrophobic interactions	Yes	Yes	PCA	DMEM		High selectivity against DENV	89
SARS-CoV-2 pseudovirus	Fe_3O_4-Au nanocomposite and Au nanoneedles array	His-tagged ACE2	Electrostatic interaction	Yes		SVM (100%)	Nasal and throat swabs	100 copies/mL	15 minutes	90
SARS-CoV-2 two variants & CoV-NL63	AgNR@SiO_2 array	ACE2	Electrostatic interaction & BSA blocking	Yes	Yes	CNN	PBS	10 - 20 PFU/mL	20 minutes	91
SARS-CoV-2	Au-coated Silmeco	ACE2 mimetic peptide	Thiol - Au bonding	Yes	Yes	Multivariate curve resolution (MCR)	PBS	300 nM for spike protein	High selectivity against SARS-CoV-1 and MERS	92
SARS-CoV-2 Delta and Omicron variants	Ag nanoforest	DNA aptamer	Thiol – Ag bonding	Yes	Yes	ROC curve	Nasopharyngeal swab	240 aM of spike protein		93
**Viral protein**										
Spike protein of SARS-CoV-2	Au nanoislands on graphene	SARS-CoV-2 S1 antibody	EDC/NHS chemistry	Yes	Yes		PBS	100 ng/mL		94
Spike & nucleocapsid proteins of SARS-CoV-2	Au nanoneedle array	ACE2	noncovalent interactions between amide groups (negative charge) and His-tagged ACE2	Yes		PCA	PBS<br>Urine	17.7 pM in PBS<br>80 copies/mL in urine	10^6-fold virus enrichment<br>5 minutes	95
Spike protein of two SARS-CoV-2 variants and Spike protein of SARS-CoV-1	AgNR@SiO_2 array	ACE2	Electrostatic interaction & BSA blocking	Yes	Yes	CNN	PBS	~ 10−11 g/mL	20 minutes	91

SARS-CoV-2 S protein and variants; clinical SARS-CoV-2 samples	Au nanoarray SERS chip combined with Fe_3O_4 magnetic enrichment beads	ACE2	Fe_3O_4 beads aminated with APTMS; ACE2 covalently linked to amino- Fe_3O_4 through amide bonding	Yes	Yes	SVM	DPBS; cultured virus samples; clinical throat-swab samples	1 pg/mL for SARS-CoV-2 S protein; Ct ≈ 39 for SARS-CoV-2 BF.7	Magnetic enrichment factor: ~10.6–11.9×; S protein detection accuracy: 96.67%; sensitivity: 97.78%; specificity: 100%; clinical blind-test accuracy: 94%; variant identification accuracy: 96.59% for 8 SARS-CoV-2 variants	96
SARS-CoV-2 S protein; SARS-CoV-2 S protein aerosol; SARS-CoV-2 Spike pseudovirus	3D Ag/ZnO nanorod arrays grown on inner wall of quartz capillary	ACE2	ACE2 adsorption & BSA blocking	Yes	Yes	Peak identification; linear calibration	PBS; pharyngeal swab solution; aerosolized PBS/PSS samples; TBST for pseudovirus	1 fg/mL for S protein in PBS/PSS; 10 fg/mL for S protein aerosol; 100 fg/mL for Spike pseudovirus	specificity against MERS-CoV S1 and H1N1 Hemagglutinin/HA0 Protein; micro-volume capillary assay	97
Nucleoproteins of 3 Flu strains	AgNR array	Polyvalent anti-influenza aptamer	5’-C6 disulfide bond	Yes		PCA	Cell lysate			98, 99
Spike protein of SARS-CoV-2	Ag nanoparticles	DNA aptamer	Thiol – Ag bonding	Yes	Yes	PCA	PBS	1 fM		100
Spike protein of SARS-CoV-2 (Delta & Omicron variants)	Ag nanoforest	DNA aptamer	Thiol group at the 5′ end of the aptamers	Yes	Yes	ROC curve	Nasopharynge al swab	240 aM of spike protein		93
hemagglutinin protein of Influenza A virus	Ag nanoisland	DNA aptamer	Thiol – silver bonding	Yes	Yes		PBS Biological fluids	190 viral particles/mL		101
Spike protein of SARS-CoV-2	Au-coated Silmeco	ACE2 mimetic peptide	Thiol (cysteine)-gold bonding	Yes	Yes	Multivariate curve resolution (MCR)	PBS	300 nM	High selectivity against SARS-CoV-1 and MERS	92
**Viral DNA/RNA**										
SARS-CoV-2 RNA	AgNR array	Thiolated ssDNA	Thiol – Ag bonding	Yes	Yes	SVM RF BP CNN RNN	Human nasopharyngea l specimens	1000 copies/mL		102
RNA sequences associated with the PB1-F2 protein of Flu	AgNR array	Thiolated ssDNA	Thiol – Ag bonding	Yes	Yes	PLS HCA	Buffer	10 nM		103
RNA sequences associated with the neuraminidase (NA) stalk motifs	CTAB coated Au NP	5'-thiolated (ssDNA)	Thiol – Au bonding	Yes		PLS-DA SVM	Buffer			104

of N1 subtype influenza A										
SARS-CoV-2 RNA, nucleocapsid phosphoprotein (N) gene	Au NP	Antisense oligonucleotides	Thiol – Au bonding	Yes		PCA<br>SVM	Nasal swab	63 copies/mL		105
Respiratory syncytial virus (RSV; synthetic RSV RNA-mimic tDNA)	Fe_3O_4@SiO_2@Ag magnetic core–shell-satellite microspheres integrated with Au nanotriangle microfluidic chip	RSV-specific pDNA probe	Fe_3O_4@SiO_2@Ag surface silanization/carboxylation, EDC activation, streptavidin coupling, then pDNA conjugation	Yes	Yes		PBS	1 pM		106
SARS-CoV-2 genomic sequence targeting RBD region of Spike protein; synthetic RBD ssDNA used as viral RNA analogue	AuNPs@glass	Thiolated peptide nucleic acid probe complementary to SARS-CoV-2 RBD ssDNA	Au–S chemisorption	Yes	Yes	Principal component analysis (PCA) and regression (PCR)	Hybridization buffer; simulated saliva buffer	110 ± 20 pM in buffer; ~220–260 pM in simulated saliva		107
RSV gene / tRSV-DNA	TiO_2 nanorod@AgNP chip + magnetic multifunctional SERS microspheres （Fe_3O_4@SiO_2@AgNPs）	RSV probe DNA (biotin-modified pDNA)	Streptavidin (SA) modified on carboxylated Fe_3O_4@SiO_2@AgNP microspheres via EDC activation; biotin–SA coupling of RSV-pDNA; magnetic enrichment onto TiO_2NR@AgNP chip	Yes	Yes	Peak-intensity calibration	Buffer	<1 pM		108
**Other target molecules**										
Breath volatile organic compounds (BVOCs)	Ag nanocubes	4-MBA, 4-MPY, 4-ATP	Thiol – Ag bonding	Yes		PLSDA	Breath flows			109

Table S6. Summary of labeled detection of intact virus.

Target Viruses	Detection strategy and combined technology	SERS Tag (NPs; capture molecules; Raman reporters)	Capture substrate (SERS substrate; capture molecule)	Spectral analysis methods	Media	Detection range	LOD	Detection time	Other sensor performance	Ref.
Respiratory Syncytial Virus (RSV)	Sandwich ELISA + HRP–TMB reaction with SERS readout via AgNP aggregation	Citrate-AgNPs; anti-RSV Ab (HRP-labeled); oxidized TMB as Raman reporter	ELISA plate coated with anti-RSV capture Ab	Linear calibration curve with SERS intensity vs. RSV concentration	PBS buffer and cell lysates	0.5–20 pg/mL	0.05 pg/mL	~85 min	~20× lower LOD vs colorimetric ELISA; works in cell lysate	110
Avian Influenza A/H3 subtype	SERS-based immunoassay using extrinsic Raman labels	AuNPs with 4-NBT and antibody monolayer; ~60 nm size	NHS-acrylic acid modification of antibodies to stabilize binding to AuNP	Calibration curve with SERS intensity vs. virus concentration	PBS and allantoic fluid	0.5–5000 ng/mL	0.38 ng/mL	~60 min	High specificity for H3 subtype; inter-assay variability in intensity due to uncontrolled aggregation	111
Influenza A H3N2 virus	Magnetic sandwich immunoassay, magnetic enrichment and separation	Au NPs; Influenza A IgG; 4-MBA Raman reporter	Fe_3O_4/Au magnetic NPs; Influenza A IgG	SERS intensity against virus concentration; linear calibration	PBS (virus standards); inactivated virus	10^2–5×10^3 $TCID_{50}$/mL	10^2 $TCID_{50}$/mL	~25 min	Portable Raman; magnetic preconcentration; high specificity vs H1N1; POC-ready	112
Oseltamivir-resistant influenza A pH1N1 (H275Y mutant)	SERS-based detection using OHT-functionalized urchin Au nanoparticles	Urchin Au NPs; oseltamivir hexylthiol (OHT) receptor; MGITC Raman reporter	OHT-functionalized glass slide; OHT capture receptor	Calibration curve of SERS intensity vs. virus concentration	DI water; human nasal fluid & saliva	1–10^3 PFU	1 PFU	~5 h	Selective mutant vs WT discrimination; works in mixed WT/mutant samples; clinically relevant fluids	113
Antiviral multidrug-resistant influenza A (I223R/H275Y pH1N1)	Sandwich immunoassay with lateral flow assay	Au NPs; HA antibody (immunoprobe); MGITC Raman reporter	Single-crystalline Au nanoplates; A4 mutation-specific antibody (via protein G)	SERS intensity plotted against virus concentration to construct calibration curves	PBS; human nasopharyngeal swab / nasal fluid	1–10^3 PFU	1.5 PFU for I223R/H275Y mutant virus	~30–60 min (SERS/LFA formats)	Discriminates multidrug-resistant vs WT/single-mutant viruses; compatible with POC LFA; naked-eye + SERS dual-mode	114
Norovirus (NoV; GII subtypes)	Dual SERS nanotag/substrate sandwich immunoassay with immunomagnetic separation + graphene-mediated SERS	Mag-MoO_3 nanocubes (plasmonic/magnetic); anti-NoV Ab; reporter-free (SLGO) or 4-MBA co-reporter	Single-layer graphene oxide (SLGO) on glass; anti-NoV Ab (±4-MBA on SLGO)	Raman intensity of SLGO D/G bands (1348/1597 cm^{-1}) or 4-MBA peaks (1096/1596 cm^{-1})	PBS; human serum; human fecal samples	10 fg/mL–100 ng/mL (LPs); 10^2–10^6 RNA copies/mL (feces)	5.2 fg/mL (LPs); ~60 RNA copies/mL (feces)	~30 min incubation (+ magnetic separation)	~10^3× lower LOD vs ELISA; high specificity; RSD < 9%; POC-compatible	115

Hepatitis E virus (HEV); Norovirus (NoV)	Nanogel-regulated sandwich immuno-SERS with magnetic enrichment and pH-triggered hotspot formation	MoO_3 QDs encapsulated in pH-responsive nanogels; virus-specific Abs; 4-MBA reporter	2D hexagonal boron nitride (h-BN) substrate; Ab-functionalized maleimide magnetic NPs	Raman intensity at 1098 cm^{-1} (4-MBA); log–log calibration	PBS; human serum; clinical fecal samples	HEV-LPs: 10 fg/mL–1 ng/mL; clinical HEV: 10^2–10^8 copies/mL; NoV: 10^1–10^5 copies mL^{-1}	6.5 fg/mL (HEV-LPs); 87 copies/mL (HEV); ~10 copies/mL (NoV)	~30–40 min (immuno-magnetic capture + pH release)	~10^3× sensitivity gain vs glass; RSD ≈ 6%; stable ≥30 days; strong matrix tolerance	116
Oseltamivir-resistant influenza A pH1N1 (H275Y; also I223R/H275Y)	Mutation-specific sandwich immuno-SERS using 6E3 monoclonal antibody	Au NPs; 6E3 antibody; MGITC Raman reporter	Single-crystalline Au nanoplates (protein-G mediated); 6E3 antibody	Integrated MGITC band at 1615 cm^{-1}; quantitative calibration	PBS; human nasopharyngeal aspirate	10^2–10^5 PFU/mL	10^2 PFU/mL	~2 h	High selectivity vs WT; detects 0.1% mutant in WT/mutant mixtures; works without sample pretreatment	117
SARS-CoV-2	Magnetically assisted sandwich immunoassay	Hollow Au NPs; reporter Ab; MGITC	Magnetic beads; detection Ab	MGITC peak intensity at 1170 cm^{-1}; linear calibration	PBS; viral lysate; nasopharyngeal aspirates	Antigen: 10–100 pg/mL; Virus: 1–1000 PFU/mL	2.56 fg/mL (antigen); 3.4 PFU/mL (virus)	≤30 min	High specificity vs influenza; clinical validation; on-site POC compatible	118
SARS-CoV-2 (WT + 13 variants incl. Alpha–Omicron/XBB)	Sandwich immunoassay	Au NPs; R2 reporter Ab; MGITC	Flexible plasmonic Au nanostructure (PI nanopillars/Au); D5 capture Ab	MGITC peak at 1174 cm^{-1}; linear calibration	Viral lysate; nasopharyngeal swabs; aerosols (face mask)	~10^2–10^5 PFU/mL	2.30 PFU/mL	~2 h	98.9% sensitivity, 100% specificity (270 clinical samples); broad-variant coverage; flexible & wearable	119
SARS-CoV-2 (WT + variants: D614G, K417N/E484K/N501Y, Omicron)	One-step aptamer sandwich SERS (OSARRA) with magnetic enrichment	AuNPs (35 nm); spike-RBD aptamer cocktail (CoV-2-1C/4C/6C); Nile Blue A (NBA)	Streptavidin magnetic beads; biotinylated spike-RBD aptamer cocktail	NBA peak at 592 cm^{-1}; linear calibration	PBS; pseudovirus; oropharyngeal swabs	250–10,000 TU/µL	124 TU/µL	≤5 min	95% sensitivity, 100% specificity (blind swabs); no pretreatment; cost ≈ \$0.31/test; POC-ready	120
SARS-CoV-2	SERS-ELISA	Au core–satellite NPs @SiO_2; anti-N protein Ab; BDMT (dithiol linker/reporter)	384-well glass plate; anti-N protein capture Ab	Raman mapping; BDMT peak at 1597 cm^{-1}; sigmoidal calibration	Viral lysate; clinical nasopharyngeal samples	0–1000 PFU/mL	8.81 PFU/mL	<30 min	~10× more sensitive than ELISA, ~100× vs LFA; 100% sensitivity for Ct < 30; high reproducibility (RSD ≈ 5%)	121

Influenza A H1N1; Human adenovirus (HAdV)	Magnetic SERS-based lateral flow immunoassay (LFIA)	Fe_3O_4@Ag MNPs; virus-specific Abs; DTNB (dual-layer)	NC membrane LFA strip; test lines coated with anti-H1N1 and anti-HAdV Abs	Raman intensity of DTNB at 1332 cm^{-1}; log calibration	PBS; human blood, serum, sputum	H1N1: 50–10^7 PFU/mL; HAdV: 10–10^7 PFU/mL	H1N1: 50 PFU/mL; HAdV: 10 PFU/mL	~30 min	~2000× more sensitive than Au-LFA; multiplexed detection; no sample pretreatment; RSD <5%; stable for 2 months	122
SARS-CoV-2 (spike, nucleocapsid, intact virus)	Digital SERS-enabled lateral flow test dipstick for rapid virus quantification	Au@Ag core–shell NPs; anti-S or anti-N Abs; 4-MBA	NC membrane LFT strip; ACE2 (spike) or anti-N Ab (nucleocapsid/virus)	Large-area SERS mapping + digital thresholding (sum of positive pixels, 1076 cm^{-1})	PBS; viral lysate; indoor dust suspensions	Proteins: fg–ng; Virus: 5–2500 PFU	180 fg (spike); 120 fg (N); 7 PFU (virus)	<30 min	~10× more sensitive than commercial antigen LFTs; robust to dust; quantitative POC potential	123
Avian influenza A (H7N9)	SERS-based lateral flow immunoassay strip (LFIA)	Au@AgNPs with 4-ATP in core-shell structure; anti-H7N9 mAb	NC membrane LFA strip; test line: anti-H7N9 mAb; control: goat anti-mouse IgG	Raman intensity at 1580 cm^{-1}; calibration vs HAU	PBS; avian cloacal & throat swabs	0.0025–0.5 HAU	0.0018 HAU	~20 min	~10^3× more sensitive than HA assay; high specificity vs H5/H9/NDV; results consistent with RT-PCR	124
Influenza A (H1N1) *(co-detected with S. pneumoniae)*	Dual-mode SERS-LFA	Si@Au/Au nanogapped NPs (0.5 nm PEI gap); anti-H1N1 Ab; DTNB	NC membrane LFA strip; mixed capture Abs on single T-line	SERS mapping; DTNB peak 1331 cm^{-1}; averaged pixel intensity	PBS; saliva, throat swabs, environmental surfaces	10-10^6 PFU/mL (H1N1), 10-10^6 cells/mL (S. pneumoniae)	29 PFU/mL (H1N1), 16 cells/mL (S. pneumoniae)	<20 min	~172× more sensitive than Au-LFA; multiplexed single-line readout; 100% clinical agreement vs ELISA	125
Zika virus (ZIKV); Dengue virus (DENV-3) (NS1 biomarkers)	Multiplexed sandwich SERS-LFA (dipstick immunoassay)	Gold nanostars; anti-ZIKV NS1 Ab / anti-DENV NS1 Ab; BPE (ZIKV), MBA (DENV)	Nitrocellulose LFA strip; immobilized anti-ZIKV or anti-DENV NS1 Ab	Peak intensity at 1609 cm^{-1} (BPE) and 1584 cm^{-1} (MBA); CLS for multiplex decoding	Human serum	0–500 ng/mL	0.72 ng/mL (Zika), 7.67 ng/mL (Dengue)	~15 min	15× (ZIKV) and 7× (DENV) sensitivity improvement vs colorimetric LFA; clear multiplex discrimination; portable Raman compatible	126
Influenza Virus A (H1N1)	SERS-based lateral flow immunoassay (SERS-LFA)	AuNPs (~33 nm); ferret anti-influenza A pAb; MGITC	Nitrocellulose LFA strip; mouse anti-influenza A mAb (test line)	MGITC peak at 1614 cm^{-1}; averaged multi-point intensity; 4-parameter logistic fit	Distilled water; virus standards	10^2–10^6 PFU/mL	1.9×10^4 PFU/mL	~15 min	~10× sensitivity improvement over colorimetric LFA; quantitative readout; portable Raman compatible	127
Group A rotavirus (RVA)	Dual-signal SERS–LFIA sandwich immunoassay	Au nanostars; anti-RVA mAb1; DTNB reporter	LFIA nitrocellulose strip; T line with anti-RVA mAb2; C line with goat anti-mouse IgG	Calibration of Raman intensity vs log viral load	Animal fecal samples from swine, cattle, and poultry	5.12×10^2–6.56×10^4 copies/μL	Visual: 1.02×10^3 copies/μL; SERS: 3.73×10^2 copies/μL	20 min	No cross-reactivity with common diarrheal pathogens; intra-batch CV 3.3%, inter-batch CV 4.0%; retained 95% Raman signal after 28 days and	128

									90% after 60 days at 4 °C; 95% agreement with RT-PCR for 80 clinical fecal samples	
H1N1 and SARS-CoV-2	Multiplex colorimetric/SERS dual-mode LFIA; sandwich assay	PDA core decorated with Au@Ag NPs; virus-specific detection antibodies; DTNB reporter	LFIA strip with two test lines; immobilized capture antibodies for H1N1 and SARS-CoV-2	Sigmoid calibration	Inactivated viruses; clinical nasopharyngeal swab specimens	H1N1: 1.65 × 10^4–1.65 × 10^7 copies/mL; SARS-CoV-2: 2.46 × 10^3–1.23 × 10^7 copies/mL	H1N1: 2.18 × 10^3 copies/mL; SARS-CoV-2: 624 copies/mL	<20 min; optimized assay reaction ~15–16 min	100% clinical accuracy; 756-fold/394-fold lower LOD than commercial Au-LFIA	129
Porcine epidemic diarrhea virus (PEDV)	SERS-based lateral flow immunoassay	Gold nanoantennas/AuNP dimers; anti-PEDV antibody; 4-MBA Raman reporter	LFIA strip with NC membrane; anti-PEDV monoclonal antibody on T line; goat anti-mouse IgG on C line	Linear calibration vs log[PEDV]	PEDV standards; spiked porcine feces	1.0–1.0 × 10^6 $TCID_{50}$/mL	1.0 $TCID_{50}$/mL by SERS; 1.0 × 10^2 $TCID_{50}$/mL by naked-eye observation	20 min	Specificity against other porcine/bacterial pathogens; intra-batch RSD 3.34%, inter-batch RSD 6.39%; stable for 6 months; feces recovery 94.91–102.1%	130
Chikungunya virus (CHIKV)	Magnetic enrichment + SERS-LFIA for intact-virus detection	Fe_3O_4@Au-DTNB magnetic SERS tags; anti-CHIKV E1 antibody conjugated through EDC/NHS; DTNB Raman reporter	LFIA strip; anti-CHIKV E1 antibody immobilized on T-line; mouse anti-human IgG on C-line	Calibration curve vs log viral concentration	Inactivated CHIKV; mosquito homogenate samples; field-collected mosquito pools	Inactivated CHIKV: ~10^3–1.74×10^8 copies/mL; E1 protein: 0.01–100 ng/mL	Inactivated CHIKV: 887 copies/mL; recombinant E1 protein: 15 pg/mL	~30 min total: 15 min magnetic capture/enrichment + 15 min LFIA	Specificity against DENV, JEV, ZIKV, CCHFV, HRP Ⅱ; RSD <6%; detected 3 positive and 6 negative mosquito samples consistent with RT-qPCR	131
Influenza A virus; *Streptococcus pneumoniae*	Dual-channel magnetic enrichment SERS-LFA	Fe_3O_4@Au-WGA magnetic SERS tags; dual-layer DTNB reporter; wheat germ agglutinin (WGA) as universal capture molecule	LFA strip; anti-FluA antibody on T1 line; anti-*S. pneumoniae* antibody on T2 line	Calibration curve vs log concentration; ROC analysis for clinical samples	Throat swabs and sputum; FluA and *S. pneumoniae* samples	FluA: 10–10^6 copies/mL; *S. pneumoniae*: 10–10^6 cells/mL	FluA: 14 copies/mL; *S. pneumoniae*: 10 cells/mL	~40 min total: 30 min magnetic capture + 10 min LFA	Capture efficiency: 98–100% for FluA, 95–100% for *S. pneumoniae*; RSD <5%; 100% accuracy in 110 clinical samples; 19% higher than commercial Au-LFA	132
SARS-CoV-2	Sandwich assay with nanomixing	Au–Ag alloy nanoboxes; polyclonal anti-RBD Ab; MMC	Gold microelectrode array; nanoyeast-scFv (S309) capture probes	SERS mapping; mean intensity at 1603 cm^{-1}; log-linear calibration	PBS; diluted human saliva (simulated patients)	~20–20 000 virus/µL	17 virus/µL	~3 h (28 electrodes, parallel)	Comparable to RT-qPCR without amplification; high specificity; scFvs are low-cost, rapidly produced Ab alternatives	133

SARS-CoV-2	Sandwich assay with nanomixing	Au–Ag nanoboxes; mPEG surface for oriented αN BsAb conjugation; αN BsAb composed of anti-nucleocapsid nanobody + anti-mPEG scFv; 4-mercaptobenzoic acid (MBA) reporter	Asymmetric ring-circular gold electrode microchip; anti-nucleocapsid antibody capture; AC-field nanomixing	Digital SERS active-pixel counting; thresholded 1580 cm^{-1} Raman signal; linear regression; ROC analysis	PBS buffer; clinical nasopharyngeal swab samples in viral transport medium or PBS	Inactivated virus: 10^3–10^7 copies/mL; nucleocapsid protein: 0.5–5000 ng/mL	2.7 copies/mL for inactivated SARS-CoV-2; 2.01 ng/mL for nucleocapsid protein		Clinical test: sensitivity 90%, specificity 73.33%, accuracy 81.67%, AUC 0.8783; high specificity from mPEG antifouling and nanomixing-reduced nonspecific binding	134
SARS-CoV-2 (spike antigen; pseudotyped virus)	Signal-off		Gold nanostars (GNS); spike-specific DNA aptamer; Rhodamine-6G (quenched reporter)	NSET fluorescence change; corroborated by Rh-6G SERS bands (376, 615, 778 cm^{-1})	Buffer; artificial nasal mucus	Antigen: fg/mL range; Virus: 10–500 particles/mL	130 fg/mL (antigen); ~8 particles/mL (virus)	≤10 min	Label-free operation; high selectivity vs flu/rotavirus; virus inhibition (ACE2 blocking, membrane damage)	135
Influenza A/H1N1	Signal-off		Au nano-popcorn NPs; HA-specific DNA aptamer; Cy3	Calibration curves; Raman mapping (625 pixels); Cy3 peak ~1590 cm^{-1}; ensemble averaging	PBS; artificial serum/mucus	10–75,000 PFU/mL	97 PFU/mL	~20 min	~1000× more sensitive than ELISA; high reproducibility (RSD ≈ 6%); discriminates H1N1 vs H3N2	136
Influenza A virus: H1N1, human/avian H3N2, H7N1	Signal-off		Spike-like PET track-etched membrane coated with 20 nm Ag; RHA0385 aptamer captures influenza A HA; Cy3	SEL/SERS ratio; positive/negative discrimination	Diluted allantoic fluid, PBS, clinical nasal swabs	120–1.1 × 10^8 VP/mL, strain-dependent	120–2000 VP/mL	~10–20 min	Broad influenza A subtype detection; 100% specificity against NDV and influenza B; stable Ag coating in biological media; qualitative screening, not quantitative	137
Model antigen system (anti-goat IgG) *(methodological platform; virus-agnostic)*	Aggregation-induced enhancement	Au@Ag core–shell NPs; DNA-conjugated M13 phage (antigen-binding p3 proteins); Cy3 embedded reporter	Silica microparticles (streptavidin-coated); biotinylated capture Ab	Raman intensity at 1580 cm^{-1} (Cy3); single-bead SERS mapping; layer-by-layer amplification	PBS buffer	10 pM–10 nM antigen	≈5 fmol (10 pM)	~1–2 h	~75× signal amplification vs antibody-based SERS; enables single-particle readout; multiplex-ready via DNA coding	138

SARS-CoV-2 (WT + Alpha, Beta, Gamma, Delta, Omicron; pseudotype)	Aggregation-induced enhancement	Au-decorated Fe_3O_4 nanocubes (plasmonic–magnetic heterostructure); ACE2-derived peptide; 4-ATP		4-ATP peaks at ~1590 and 1078 cm^{-1}; intensity vs concentration	Buffer; artificial nasal mucus			~30 min	~100% magnetic separation; strong variant-dependent binding (Omicron highest); complete inhibition of pseudovirus entry	139
SARS-CoV-2 (pseudotyped virus)	Aggregation-induced enhancement (self-assembly of single-virus hotspots on solid surfaces)	Au@Ag NPs; spike-protein aptamer (MSA1-T3); Cy3-labeled aptamer	Solid surfaces (Si wafer, packaging materials); no pre-fabricated SERS substrate	Raman mapping; Cy3 peak at 1590 cm^{-1}; average mapping intensity	Reaction buffer; packaging surfaces (plastic, foil, etc.)	10^1–10^4 $TCID_{50}$/mL	5.26 $TCID_{50}$/mL	~20 min	True in-situ detection (no sampling); 100% sensitivity & accuracy (20 blind samples); surface contamination down to ~1 $TCID_{50}$ cm^{-2}	140

Table S7. Summary of labeled detection of viral proteins.

Target Viruses	Detection strategy and combined technology	SERS Tag (NPs; capture molecules; Raman reporters)	Capture substrate (SERS substrate; capture molecule)	Spectral analysis methods	Media	Detection range	LOD	Detection time	Other sensor performance	Ref.
SARS-CoV-2 and H1N1 co-infection (target proteins)	In situ sampling + immuno-flocked swab capture + magnetic enrichment + multichannel SERS immunoassay (sandwich: swab–virus–SERS tag)	Magnetic multihotspot SERS nanotags: Fe3O4@SiO2–Au/Ag "satellite/island" NPs; virus-specific detection antibodies on tags; DTNB reporter (SARS-CoV-2 channel) and 4-MBA reporter (H1N1 channel)	Immuno-flocked swab (nylon fibers) functionalized (carboxylated) and conjugated with capture antibodies for SARS-CoV-2 and H1N1	Calibration curves	Nasopharyngeal swabs, clinical media	50–10^6 pg/mL	7.76 pg/mL for SARS-CoV-2 and 8.13 pg/mL for H1N1	15 min	98 clinical samples, High sensitivity (88% for SARS-CoV-2, 95.83% for H1N1) and specificity (100% for both viruses)	141
Hepatitis B virus (HBV) surface antigen	Sandwich immunoassay with silver staining enhancement	Au NPs (~13 nm); anti-HBsAg monoclonal antibody; MBA reporter	Si or quartz chip; immobilized anti-HBsAg polyclonal antibody	Calibration curves	PBS buffer	1–40 μg/mL	0.5 μg/mL	~4 hours (total protocol time)	High specificity; Ag staining gives 10–100× enhancement; minimal nonspecific binding.	142
Bovine Leukemia Virus (BLV) gp51 antigen	Magnetic sandwich immunoassay	Au nanorods; anti-gp51 antibody; DTNB/TNB Raman reporter	Magnetic Au–Fe_3O_4 NPs (MNP-Au); fragmented anti-gp51	Calibration curves	PBS; 9% milk (spiked)	0–0.06 mg/mL (PBS); 0.025–0.2 mg/mL (milk)	0.95 μg/mL	~65 minutes	High linearity; milk recovery 85.5–100%; RSD ≤ 8.9%; magnetic separation improves specificity	143
West Nile virus (WNV) (E protein) & Rift Valley fever virus (RVFV) (N protein)	Magnetic capture–based multiplex immunoassay (Au NPs + paramagnetic NPs; sandwich format)	Au NPs (60 nm); anti-E (WNV) or anti-N (RVFV) IgG; IR-792 (WNV) and Nile Blue (RVFV) reporters	Fe_3O_4 paramagnetic NPs (~200 nm); polyclonal anti-E or anti-N IgG	Calibration curves	PBS; PBS + 5% FBS	0.005–50 pg/mL (PBS); 0.1–100 ng/mL (5% FBS)	5 fg/mL (PBS); 0.1 ng/mL (5% FBS)	~1 h	log-linear over >3 orders; strong serum interference noted	144
HBV (HBsAg antigen)	Microfluidic sandwich immunoassay	Au nanoflowers; anti-HBsAg Ab; basic fuchsin (FC)	Au–Ag–coated GaN nanopillar substrate; anti-HBsAg Ab	Calibration curves with peak intensity	Human plasma & serum	0.0125–60 IU/mL (plasma/serum)	0.01 IU/mL	~40–60 min	RSD <10%; high specificity; GaN/Au–Ag substrate gives ~60× LOD improvement vs non-SERS surface	145
Influenza A virus (H1N1) nucleoprotein antigen	sandwich assay	25 nm AuNPs; anti-influenza A NP Ab; TBBT	2D Au@Ag NP array on ITO; immobilized NP antigen (direct assay)	Calibration curves with peak intensity	Infected allantoic fluid	5–56 $TCID_{50}$/mL	6 $TCID_{50}$/mL	~1.5–2 h (30 min antigen + 1 h probe + washing)	~4× sensitivity vs flat Au; RSD ~4.6%; high selectivity	146

Zika virus (ZIKV) (NS1 antigen)	Sandwich immunoassay	Au-SHINs (~100 nm Au core + SiO_2 shell); anti-Zika NS1 monoclonal antibodies; Nile Blue (NB)	Glass slide; immobilized anti-ZIKV NS1 monoclonal antibodies	Calibration curves with peak intensity; interactive document map for visualization	PBS buffer	10 ng/mL to 50 µg/mL	12.5 ng/mL	~1–1.5 h (incubation ; mapping ~20–25 min)	High specificity (no DENV cross-reactivity); ~4 orders sensitivity improvement vs ELISA; EF ~10^3	147
SARS-CoV-2 spike protein	SERS-based immunoassay using sandwich structure with gold and silver nanoparticles	Ag NPs (~47 nm); anti-spike antibody; 4-MBA	Au NP bilayer on Si wafer (self-assembly); anti-spike antibody	log-linear calibration	PBS, untreated saliva, serum, blood	1 fg/mL to 10 ng/mL (saliva); 10 fg/mL to 10 ng/mL (serum); 100 fg/mL to 100 ng/mL (blood)	0.77 fg/mL (PBS); 6.07 fg/mL (saliva); 7.60 fg/mL (serum); 0.10 pg/mL (blood)	~2 h	High specificity vs HIV-1 p24, EBV GP350; good reproducibility (RSD ≈ 10%); no sample pretreatment	148
SARS-CoV-2 spike protein	Magnetic scFv-based sandwich SERS immunoassay	Silica-encapsulated Au SERS nanotags (~60 nm core); scFv antibodies; Nile Blue reporter	Magnetic nanoparticles (MNPs) conjugated with matching scFv	log-fit calibration	Viral transport medium (VTM); saliva (validated)	257 fg/mL for spike protein; 4.1 × 10^4 genomes/mL for whole virus	257 fg/mL (spike protein); 4.1 × 10^4 genomes/mL (inactivated virus)	~30 min	Detects Alpha, Beta, Delta variants; no HKU1 cross-reactivity; 10–100× more sensitive than LFA; portable, low-power	149
SARS-CoV-2 spike glycoprotein (S1)	Sandwich immunoassay	AuNPs (~14 nm) coated with MB@SiO_2 (~6 nm shell); anti–SARS-CoV-2 Ab; methylene blue (MB)	Glass cover slide functionalized with anti–SARS-CoV-2 Ab	Calibration curves	PBS	0.01 ng/mL–100 µg/mL	0.046 ng/mL (0.60 pM)	~2 h	Specific detection with minimal influence from pharmaceutical interferents; early diagnosis capability	150
SARS-CoV-2 (spike protein; variants)	Single-nanoparticle digital SERS sandwich immunoassay	Bumpy Au core–shell NPs (~120 nm) with internal nanogap; anti-S protein antibody; 4-nitrobenzenethiol (4-NBT)	Si substrate; immobilized anti-S protein antibody	Digital SERS	Serum, PBS buffer	3.7 × 10^{-15} to 3.7 × 10^{-8} M (PBS)	7.1 × 10^{-16} M	~5 h	Detects Alpha, Beta, Gamma, Delta, Omicron mutations; far outperforms ELISA in LOD & dynamic range	151
SARS-CoV-2 (S1 protein)	Dual-modal colorimetric and SERS-based lateral flow immunoassay (LFIA)	Gold nanocrown with core–gap–shell morphology; anti-S1 antibody; HITC reporter	LFIA nitrocellulose membrane; immobilized anti-S1 capture Ab	Calibration curves	PBS; human saliva (direct, no pretreatment)	100 fg/mL to 1 µg/mL (PBS & saliva)	57.21 fg/mL (PBS); 39.65 fg/mL (saliva)	~20 min	>10^3× sensitivity improvement vs colorimetric LFIA; high reproducibility (RSD ≈ 5%); portable Raman compatible	152
SARS-CoV-2 (spike glycoprotein)	sandwich immunoassay	annular Au nanotrenches (~120 nm); anti-spike Ab; 4-MBA	Au-coated Si wafer; immobilized anti-spike Ab	Calibration curves	PBS; clinical nasal fluid	Spike protein: fg/mL–ng/mL (PBS); Virus particles: pg–fg/mL range	~1 fg/mL (spike protein); Virus: 500 fg/mL (B.1), 5 pg/mL (Delta), 50 pg/mL (Omicron)	~1–2 h	Clinical samples (n=50): 96% sensitivity, 100% specificity; ~10^6× more sensitive than ELISA; detects multiple variants	153

SARS-CoV-2 spike protein	sandwich immunoassay	AuNPs; anti-SARS-CoV-2 spike antibody; 4-MBA reporter	Ag nanotripod array; 3-MPA-linked anti-SARS-CoV-2 spike antibody	linear calibration vs log concentration; multimodal fusion neural network for mixed proteins	PBS; saliva	PBS: 10 fg/mL–1 ng/mL; saliva: 1 pg/mL–10 ng/mL	PBS: 0.10 pg/mL; saliva: 0.77 pg/mL	~60 min assay incubation; saliva pretreatment by centrifugation 30 min	RSD < 8%; peak RSD ~5–6%; good specificity against IL-6 and BSA; saliva recovery 81.4–106.7%; 100% validation accuracy for classifying IL-1, TNF-α, SARS-CoV-2 spike, and mixed samples	154
SARS-CoV-2 S2 spike protein	Sandwich SERS immunoassay using dual-enhancement hotspot engineering	Au nanoraspberry@4-ATP@Ag core–shell nanotags; SARS-CoV-2 S2 polyclonal Ab; 4-ATP reporter	FTO–SiO_2–AuNP chip; SARS-CoV-2 S2 monoclonal Ab	Linear fitting of SERS intensity vs log concentration	PBS; human serum albumin (HSA)	1–500 ng/mL	1.07 pg/mL in PBS; 1.30 pg/mL in HSA	30 min	Selective vs H1N1, H3N2, RSV, SARS-CoV S1; RSD 7.5%; recovery ~96%	155
SARS-CoV-2 S and N proteins; influenza A H1N1 protein	sandwich immunoassay on wax-printed paper	AuNPs; target-specific antibodies; MBA for SARS-CoV-2 S/N proteins and DTNB for H1N1 protein	Wax-printed Whatman filter paper; antibody-conjugated AuNP capture probes	log-linear calibration; spectral deconvolution for multiplexing	PBS	SARS-CoV-2 S/N: 0.05–100 ng/mL; H1N1/SARS-CoV-2 multiplex test: 0.5–100 ng/mL and antigen ratios of 10:0 to 0:10	SARS-CoV-2 S: 0.8 pM; SARS-CoV-2 N: 2.0 pM; multiplex test: SARS-CoV-2 S ~252–300 pg/mL, H1N1 ~160 pg/mL	≥10 min drying; Raman acquisition 1000 ms × 10 scans	Portable and low-cost platform; 15-day signal stability; SERS intensity correlated with concentration and PCR CT values; proof-of-concept dual-analyte detection	156
Hepatitis B virus (HBV) surface antigen	Magnetic sandwich SERS immunoassay	AuNPs (~55 nm); anti-HBsAg Ab; Nile Blue A (NBA)	Carboxylated magnetic beads; anti-HBsAg capture Ab	linear calibration curves	PBS buffer and human serum	3.5–1771 pg/mL	2.88 pg/mL	12 min	High specificity with 100% sensitivity and specificity; clinical sample testing validated results	157
Rotavirus antigen	SERS-enhanced immunochromatographic assay (SERS-ICA) (LFIA)	Au-core/Ag-shell NPs with dual-layer reporter; anti-rotavirus Ab; DTNB	Nitrocellulose LF strip; immobilized anti-rotavirus Ab (test line)	Calibration curves	Feces (clinical)	8–40,000 pg/mL	8 pg/mL	~20 min	No cross-reactivity; RSD < 10%; 100% agreement with RT-qPCR & Au-ICA (95 samples)	158
SARS-CoV-2, Influenza A (IAV), Influenza B (IBV), RSV (viral antigens)	Sandwich immunoassay; electrohydrodynamic nanobox-on-mirror (E-NoM) multiplex SERS immunoassay; electro-mixing-enhanced antigen capture	Au–Ag alloy nanoboxes (core–shell); virus-specific antibodies; distinct Raman reporters for each virus	Gold microelectrode mirror array; immobilized capture antibodies	Calibration curves; multiplex SERS fingerprint peak intensity; parallel readout of 4 channels	Nasal swabs, serum	0.352–0.612 pg/mL (LOD varies by virus)	SCV2: 0.352 pg/mL, IAV: 0.445 pg/mL, IBV: 0.612 pg/mL, RSV: 0.612 pg/mL	~30 min	Clinical performance: 100.0% sensitivity, 96.48% specificity, 96.91% accuracy; true multiplexing; strong signal uniformity via EHD flow	159, 160

SARS-CoV-2 spike protein	Aptamer-based SERS biosensor with plasmonic coupling + catalytic hairpin assembly (CHA) signal amplification	Au@Ag core–shell NPs; DNA hairpin (H2); 4-mercaptobenzonitrile (4-MBN)	Au NPs on COFs (Au/COFs); thiolated DNA hairpin (H1)	Log-linear calibration	Buffer; human saliva (spiked)	1×10^{-16} to 1×10^{-11} g/mL (buffer/saliva)	3.0×10^{-16} g/mL	~2 h	High specificity vs other viral proteins; recovery 98.9–102.2%; RSD ~7%; enzyme-free amplification	161
SARS-CoV-2 3CL protease, 3CLpro	Dual-mode ECL/SERS ratiometric biosensor; 3CLpro cleaves a DNA–peptide–DNA precursor, generating an intermediate DNA that triggers entropy-driven strand-displacement assembly and recruits Fc-labeled reporter to the electrode; signal readout: SERS increase and ECL decrease	Fc-labeled DNA reporter strand, DNA4–Fc; Raman reporter: ferrocene, Fc	$CsPbBr_3$@PDA@Au nanocomposite on glassy carbon electrode; Au provides plasmonic hotspots and DNA anchoring; DNA1/DNA2/DNA3 capture scaffold	Calibration with log concentration	Buffer; patient-derived pharyngeal swab eluates	1 aM–10^8 aM	2.44 aM	15 min 3CLpro cleavage + 30 min strand displacement; total sensing reaction ~45 min	High selectivity against non-target proteases and matrix interferents; inter-electrode RSD ≈ 2.9%; retained ~96–99% signal over 12 h and ≥90% over 7 days at 4 °C; successfully applied to clinical swab samples	162
Wild-type pseudorabies virus (PRV); discrimination vs gE-deleted vaccine	Sandwich-based lateral flow assay	Au–Ag core–shell NPs embedded with 4-ATP; anti-PRV gE mAb	NC membrane LFA strip; test line: anti-PRV gB mAb; control: goat anti-mouse IgG	Raman intensity at 1585.7 cm^{-1}; semi-quantitative calibration	Viral standards; pig tissue extracts	41–650 ng/mL	5 ng/mL	15 min	Selective for WT PRV (no signal from gE-deleted vaccine, CSFV, PEDV); portable Raman; PCR-consistent clinical results	163
SARS-CoV-2 nucleocapsid (N) protein	Digital SERS-based LFIA (Lateral Flow Immunoassay)	Au@Ag core–shell NPs; anti-N protein mAbs; RBITC	Nitrocellulose LF strip; anti-N protein mAb (test line), Protein G (control)	SERS mapping + digital counting (binary thresholding, 4PL fit)	Nasal swabs (spiked with N protein or inactivated virus)	0.001 to 10 pg/mL (N protein in nasal swab), 0.03 $TCID_{50}$/mL (viral load)	0.9 fg/mL (1.9 aM, N protein), 0.03 TCID50/mL (viral load)	~30–40 min total (≈8 min SERS mapping)	Orders-of-magnitude sensitivity improvement vs colorimetric LFIA; quantitative over 4–5 orders; high robustness via digital analysis	164
SARS-CoV-2 (S & NP proteins)	Dual-channel magnetic SERS-LFA (sandwich immunoassay)	Fe_3O_4@Au nanogap satellite NPs; anti-S / anti-NP mAbs; DTNB (dual-encoded)	Nitrocellulose LF strip; anti-S mAb (T1), anti-NP mAb (T2)	Sigmoid/log calibration	PBST buffer; throat swab	S: 0.05–100 ng/mL (buffer); NP: 0.01–50 ng /mL; virus: throat swab	S: 23 pg/mL; NP: 2 pg/mL; virus: 2.5×10^4 copies/mL	~30 min total (≈10 min incubation + 15 min flow)	Simultaneous S/NP detection; magnetic enrichment; variant discrimination (Omicron vs others); high reproducibility (RSD <6%)	165
Influenza A (FluA) and B (FluB) antigens	SERS-LFIA multiplex immunochromatography on single T-line	Raman nanospheres (Au core–Ag shell); tracer mAbs (anti-FluA / anti-FluB);	Nitrocellulose LF strip; mixed capture mAbs (anti-FluA + anti-FluB) on one T-line	Calibration curves	Buffer / lysate	0.25–50 ng/mL	0.25 ng/mL	15 min	No cross-reactivity (RSV, Cpn, MP, SARS-CoV-2); CV < 5%; 8× sensitivity vs gold LFIA	166

SARS-CoV-2 protein	Portable SERS-LFA immunoassay (on-strip Raman readout)	AuNPs (40–50 nm); anti-NP mAb; MGITC reporter	Nitrocellulose LFA strip; anti-NP mAb (test), goat anti-mouse (control)	Calibration curve	Viral transport medium (VTM); clinical nasal swabs	1–1000 PFU/mL (spiked VTM)	3.53 PFU/mL	~15 min	Sensitivity 96% (vs 57% LFA); AUC 0.980; strong specificity vs flu A/B, RSV; on-site portable reader	167
Influenza A H1N1 antigen; inactivated H1N1 virus	Dual-mode colorimetric/SERS LFIA sandwich immunoassay	Fe-doped ZnO@Au nanotag; anti-H1N1 antibody; DTNB reporter	LFIA strip/NC membrane; anti-H1N1 antibody on T line; goat anti-mouse IgG on C line	Calibration curve	Buffer-spiked H1N1 antigen; inactivated H1N1 virus; clinical nasopharyngeal/throat swab samples	Antigen: ~0.01–10^4 pg/mL; inactivated virus: ~10^2–10^7 copies/mL	Antigen: 0.047 pg/mL by SERS, 10 pg/mL visual; inactivated virus: 196.3 copies/mL by SERS, 10^5 copies/mL visual	~15 min	Specific against SARS, MERS, Flu B, PIV-I/II/III, and ADV; RSD <10%; 100% accuracy in 20 clinical samples; ROC AUC = 1.00; good storage stability	168
Influenza A virus (IAV); broad subtypes H1N1, H3N2, H5N1, H7N9, H9N2; target protein: nucleoprotein	Sandwich SERS-LFA using multivalent nanobodies and biotin–streptavidin signal amplification	2D MoS_2@Au nanosheets; streptavidin–biotinylated multivalent nanobody; DTNB reporter	LFA nitrocellulose test line; multivalent anti-IAV nucleoprotein nanobody	calibration curve; ROC analysis for clinical samples	IAV nucleoprotein in buffer; inactivated IAVs; clinical throat swab samples	Nucleoprotein: 0.001–10 ng/mL; inactivated IAVs: 0.1–10^4 PFU/mL	Nucleoprotein: 1.7 pg/mL; inactivated viruses: 42 PFU/mL (H1N1), 0.54 PFU/mL (H3N2), 0.45 PFU/mL (H5N1), 0.15 PFU/mL (H7N9), 0.62 PFU/mL (H9N2)	15 min	No cross-reactivity with tested respiratory viruses; RSD 2.18% at 5 ng/mL and 1.76% at 0.05 ng/mL; 52 clinical samples showed 100% agreement with RT-PCR, AUC = 1	169
Influenza B virus antigen; extended validation with influenza A antigen	SERS–LFIA sandwich immunoassay integrated with large-area Raman scanning and ResNet-18 deep learning image recognition; automated portable Raman readout	Au nanostars; anti-FluB-1 or anti-FluA detection antibody; DTNB reporter	LFIA nitrocellulose strip T line; anti-FluB-2 or anti-FluA capture antibody; C line with goat anti-mouse IgG	Calibration curve; ResNet-18 classification; SVM and peak-threshold comparison	Lysis buffer for FluB standards; simulated human nasopharyngeal swab lysate for FluA	FluB: 10^{-7}–10^{-16} g/mL; FluA validation: 10^{-8}–10^{-15} g/mL	FluB: visual 1 pg/mL, Raman 10 fg/mL, theoretical 2.5 fg/mL; FluA: ~10 fg/mL	10 min chromatography; Raman scanning afterward	ResNet-18: 100% training accuracy, 95% validation accuracy; overall 98% on 200 FluB samples; FluA simulated samples: 95% sensitivity, 95% specificity, 95% accuracy; better than peak intensity method and SVM	170
SARS-CoV-2 nucleocapsid protein (NP)	Automated SERS–LFIA sandwich immunoassay integrated with large-area SERS scanning imaging and ResNet-18 deep learning classification	SiO_2-coated Au nanostars; SARS-CoV-2 antibody COVID-19-PS-MAb1; DTNB Raman reporter	LFIA nitrocellulose strip; T line with SARS-CoV-2 antibody COVID-19-PS-MAb2; C line with goat anti-mouse IgG	SERS image recognition; ResNet-18 classification	Lysis buffer; simulated nasopharyngeal swab samples	0.1–10,000 pg/mL	Visual: 100 pg/mL; theoretical Raman: 1.8 pg/mL; image-based reliable detection: 2.5 pg/mL	10–15 min	ResNet-18: 100% training accuracy, 94.52% testing accuracy; testing sensitivity 91.43%, specificity 97.37%; AUC 0.98; reduced false positives from random probe aggregation; automated POCT readout	171

Human immunodeficiency virus p24 antigen (HIV p24) and hepatitis B virus surface antigen (HBsAg)	Electrophoresis-assisted SERS lateral flow assay	AuNP SERS nanotags; anti-HIV p24 or anti-HBsAg detection antibodies; MGITC for HIV tag and RBITC for HBV tag	Nitrocellulose membrane electrophoresis strip; no pre-immobilized capture antibody	Logistic calibration curve	Mixed viral lysates; spiked human serum	HBV: 0.0064–100 IU/mL; HIV: 0.0128–200 ng/mL	HBV: 0.0731 IU/mL; HIV: 0.0217 ng/mL	40 min	>15-fold lower LOD than conventional SERS-LFA; S/N: 52.2 for HBV and 64.3 for HIV; good selectivity in serum with no obvious cross-interference; RSD 2.33% for HIV signal and 5.15% for HBV signal in mimic clinical samples	172
Norovirus (NoV) and adenovirus (AdV)	Dual-mode immunochromatographic assay combining nanozyme-catalyzed colorimetric enhancement with SERS quantitative detection; sandwich immunoassay on ICA strip	DSAIA SERS-nanozyme tag: dendritic mesoporous SiO_2 core loaded with 5 nm AuIr nanozyme particles and coated with 35 nm AuNPs; modified with anti-NoV or anti-AdV antibodies; DTNB Raman reporter	ICA nitrocellulose strip with two test lines: anti-AdV antibody on T1 and anti-NoV antibody on T2; IgG on control line	Calibration curve of peak intensity vs virus concentration; ROC analysis for clinical samples	PBS; orange juice; human stool samples; clinical stool samples	NoV: 10–0.0001 ng/mL; AdV: 10^6–10 PFU/mL; 6-order dynamic range in SERS mode	Nanozyme visual mode: 0.001 ng/mL for NoV and 10^2 PFU/mL for AdV; SERS mode: 0.14 pg/mL for NoV and 11 PFU/mL for AdV	~20 min	RSD <10.80%; recovery 89.63–113.67%; clinical validation with 92 stool samples: NoV sensitivity 98.04%, specificity 100%, AUC 0.993; AdV sensitivity 95.00%, specificity 100%, AUC 0.998; 100-day strip stability	173
SARS-CoV-2 variants (Alpha, Beta, Gamma, Delta, Omicron) spike protein RBD	Magnetic separation + SERS identification + infection inhibition via ACE2–spike interaction	Au-decorated Fe_3O_4 nanocubes; ACE2 peptide; 4-ATP		Calibration curves	Artificial nasal mucus	10^2–3×10^3 pseudovirus/mL (nasal mucus)		~30 min incubation	High specificity, 100% separation efficiency, “hot spot” enhancement (6 orders of magnitude Raman signal)	139
Influenza virus (H1, H3, H5)	Sandwich aptasensor (aptamer–virus–aptamer)	Secondary DNA aptamers (RHA0385) labeled with Cy3 or BDP FL Raman dyes	Nanoisland Ag SERS substrate on Si/SiO_2; thiolated primary aptamer (RHA0385-SH)	Calibration curves	PBS, Allantoic fluid	6.4×10^{-5}–6.4 HAU/mL (allantoic fluid)	7×10^{-5}–1.3×10^{-4} HAU per sample (≈10^4–2×10^4 virus particles/sample)	~12 min	Broad strain specificity (H1–H12 except H9/H12 weak); RSD ~16%; antibody-free, low cost	174
SARS-CoV-2 (spike protein, viral lysate)	SERS aptasensor; “on–off” signal via aptamer release upon spike binding	DNA aptamer labeled with Cy3; 4-MBA as internal standard	Au nanopopcorn substrate; capture DNA hybridized with spike aptamer	Calibration curves with ratiometric normalization	PBS; transport medium with nasopharyngeal swabs (spiked)	0.53–1000 PFU/mL (PBS & clinical matrix)	0.53 PFU/mL (PBS); 3.67 PFU/mL (clinical matrix)	~15 min	High selectivity vs influenza A (H1N1, H3N2); RSD reduced to 2.3% using internal standard	175
SARS-CoV-2 receptor-binding domain (RBD)	SERS-based detection using a fishing mode device with aptamer functionalization	PVP-AuNPs (55 nm); RBD aptamer + NAG–MPBA small molecule; MPBA beacon	Ag acupuncture needle decorated with AuNPs; aptamer capture	Calibration curves	PBS; BSA-PBS; cell lysate; urine & blood	0.625–20 ng/mL (PBS / protein mix); 1.25–20 ng/mL (urine, blood)	0.625 ng/mL in PBS; 1.25 ng/mL in blood/urine	~15 min	High specificity (intact RBD only); stable ~8 nm hotspots; portable, ELISA-comparable accuracy	176

Table S8. Summary of labeled detection of antibodies.

Target Viruses	Detection strategy and combined technology	SERS Tag (NPs; capture molecules; Raman reporters)	Capture substrate (SERS substrate; capture molecule)	Spectral analysis methods	Media	Detection range	LOD	Detection time	Other sensor performance	Ref.
West Nile virus (WNV) *(anti-WNV envelope IgG)*	Solution-phase sandwich immunoassay	Au NPs (60 nm) coated with WNV E antigen; malachite-green–protein A/G (MG-pA/G) reporter		Calibration curves	Rabbit serum	Linear over ~4 orders (≈20 µg/mL → 2 ng/mL serum protein)	2 ng/mL serum (≈50 pg/mL IgG equiv.)	~4 h (2 h Ab binding + 2 h reporter; readout seconds)	~400× more sensitive than ELISA; broad Ig binding via pA/G; solution assay (no aggregation substrate)	177
SARS-CoV-2 (anti-S IgM & IgG)	Sandwich assay, lateral flow immunoassay (LFIA)	SiO_2@Ag core–shell NPs (~240 nm); S-protein; DTNB (dual-layer)	Nitrocellulose LFIA strip; anti-human IgM & IgG (two test lines)	DTNB peak at 1328 cm^{-1}; test-line intensity vs dilution	Human serum; whole blood	1 pg/mL to 10 ng/mL	1 pg/mL	~25 min	100 % accuracy & specificity (68 clinical samples); portable Raman; stable ≥60 days	178
SARS-CoV-2 (neutralizing antibodies against RBD–ACE2 interaction)	Sandwich assay, Competitive SERS-LFIA for neutralizing Ab quantification	SiO_2@Ag NPs; human ACE2 protein; DTNB	Nitrocellulose LFIA strip; immobilized SARS-CoV-2 RBD	DTNB peak at 1331 cm^{-1}; signal inversely proportional to Ab concentration	Human serum	0.03–1000 ng/mL	IgM: 1 ng/mL, IgG: 0.1 ng/mL	~25 min	Differentiates neutralizing vs non-neutralizing Abs; good correlation with ELISA; suitable for vaccine evaluation	179
SARS-CoV-2 (anti-S IgM / IgG)	Sandwich assay, lateral flow immunoassay (SERS-LFA) with portable line-scan Raman reader	Au nanostars; anti-human IgM or IgG secondary Ab; NIR-797-ITC	Nitrocellulose LFA strip; SARS-CoV-2 RBD (test line)	Spectrally integrated Raman intensity over TL (full spectrum integration)	Human serum; buffer (IgM standards)	IgM detectable to 1:5000 serum dilution	100 fg/mL IgM	~20–30 min	≥10× sensitivity vs naked-eye LFA; 7-order-of-magnitude improvement in purified system; compatible with POCT	180

Table S9. Summary of labeled detection of viral RNA/DNA.

Target Viruses	Detection strategy and combined technology	Amplification methods	SERS Tag (NPs; capture molecules; Raman reporters)	Capture substrate (SERS substrate; capture molecule)	Spectral analysis methods	Media	Detection range	LOD	Detection time	Other sensor performance	Ref.
HIV (gene-related DNA)	Sandwich DNA hybridization + magnetic separation	None	Ag/SiO_2 NPs + DNA probe + RBITC reporter	SiO_2-coated Fe_3O_4 magnetic NPs + capture DNA	Peak intensity comparison	PBS	~10 fM–1 nM	~10 fM	~1 h	High specificity; mismatch discrimination	181
RSV (gene/RNA)	Molecular beacon (hairpin probe) hybridization	None	Ag- or Au-metallized nanoPPX polymer substrate; Cy5 or Hex-labeled DNA molecular beacon		Peak intensity comparison	Buffer	10^{-10} M–10^{-6} M	10^{-10} M	10 min–3 h hybridization	High reproducibility (<10% variation); dual-mode self-confirmation	182
West Nile virus (WNV, DNA)	Solution-phase DNA sandwich hybridization + magnetic capture–concentration	None	Au NPs conjugated with reporter DNA; Raman reporter DSNB	Silica-coated magnetic NPs functionalized with capture DNA	Calibration curves	PBS	10 pM–100 nM	10 pM	~1 h	Portable-compatible; high specificity vs control DNA	183
Influenza A (H1N1 DNA)	DNA hybridization + AuNP aggregation-dependent fluorescence & SERS discrimination of single-base mismatch	None	Unmodified AuNPs; dye-labeled DNA (Texas Red)		Peak intensity comparison	PBS	25–50 nM (buffer)	Not reported	Minutes–<1 h (hybridization + readout)	Single-base mismatch discrimination; PL better under non-aggregation, SERS requires aggregation	184
West Nile virus (WNV); Rift Valley fever virus (RVFV)	Sandwich hybridization	None	Au@PMPs; thiolated capture DNA on Au shell; reporter DNA labeled with erythrosin B (WNV) or malachite green (RVFV)	Au-coated paramagnetic nanoparticles; capture DNA	Calibration curves	Tricine buffer (pH 7.0)	20-100 nM (RVFV); 100 - 300 nM (WNV)	20 nM (RVFV); 100 nM (WNV)	~1 h hybridization + 10 s spectral acquisition	Multiplex detection; high specificity; magnetically focused signal; portable-Raman compatible	185
Hepatitis B virus (HBV DNA)	Sandwich hybridization	None	Ag nanorice@MGITC@ SiO_2; detection DNA probe; Raman reporter MGITC	Au triangle nanoarray chip (periodic SERS substrate) functionalized with capture DNA	Calibration curves	Phosphate buffer (high-salt hybridization buffer)	50 aM–2 nM	50 aM	~40 min hybridization; 10 s acquisition	High specificity (single-base mismatch discrimination), robust and reproducible performance, portable	186
Viral infection (host-response RNA marker: RSAD2; induced by respiratory viruses)	Molecular Sentinel (MS) SERS nanoprobes: hairpin DNA probe distance-modulated SERS on Ag NPs	None	Ag nanoparticles; thiolated hairpin DNA probe; Raman reporter Cy3 (also Cy5.5 for control)		Calibration curves	Tris–HCl buffer + $MgCl_2$; total RNA from human lymph node	1–100 nM (buffer); detectable in µg-level total RNA	~1 nM	~20 min hybridization; 10 s spectral acquisition	High specificity; multiplex capable; washing-free; portable Raman compatible	187

Dengue virus serotype 4 (DENV-4 DNA)	Homogeneous DNA bioassay-on-chip based on plasmonic modulation + SERS “signal-on” detection	None	Cy5-labeled hairpin reporter DNA + placeholder DNA	Bimetallic Ag/Au nanowave chip (MFON-type) functionalized with thiolated reporter/placeholder DNA	Calibration curves	High-salt phosphate buffer (0.5 M NaCl, pH ~7)	0.1–1 µM DNA (buffer); attomole-level in probed area	~6 attomoles (within laser spot)	Single incubation ~2 h; no washing; seconds-level spectral acquisition	Washing-free; signal-on reduces false positives; multiplex potential	188
Highly pathogenic avian influenza virus (HPAI, PB1-F2 RNA marker)	Molecular Sentinel (hairpin DNA) SERS biochip; signal-off RNA hybridization on MFON substrate	None	Cy3-labeled hairpin DNA probe immobilized via thiol	MFON (metal film over nanospheres) Au substrate; capture via thiolated hairpin DNA	Calibration curves	PBS + 0.5 M NaCl (buffer)	0–60 attomoles	2.67 attomoles	Single-step hybridization (~8 h); 1 s spectral acquisition	High specificity; CV < 10%; washing-free; EF ~ 1.7 × 10^5; point-of-care potential	189
SARS-CoV-2 (RdRp & N RNA)	RNA hybridization-based indirect SERS assay (probe-labeled dye + AgNP enhancement)	None	Ag nanoparticles; FAM-labeled DNA probes targeting RdRp & N genes	PEI-coated glass wells (electrostatic RNA immobilization) + drop-cast AgNPs	Calibration curves	Extracted RNA (buffer; clinical samples)	60.23–60.23 × 10^7 copies/mL	58.39 copies/mL (Ct ≈ 38 RdRp; 37.5 N)	~1.5 h prep + 10 min hybridization; 3 s acquisition	High specificity vs RSV & Flu A; 100% concordance with PCR (24 samples); RSD ≤ 7.7%	190
Dengue virus 1 (DENV-1 DNA); *Plasmodium falciparum* DNA	Magnetic bead–assisted sandwich DNA hybridization	None	Au nanorattles (core–gap–shell) loaded with HITC Raman reporter; thiolated reporter DNA	Streptavidin magnetic beads with biotinylated capture DNA	Peak intensity comparison	Hybridization buffer (0.3 M NaCl, PBS-based)	~100 fM–100 pM (buffer)	~100 attomoles	~2–3 h incubation; 1 s acquisition	Single-nucleotide mismatch discrimination; ~10^3× brighter than Au NPs; POC-compatible	191
SARS-CoV-2 (RdRp, E, N gene DNA targets)	Two-step magnetic microparticle (MMP) hybridization	None	AgNP clusters (or Ag nanostars/triangular plates); DNA signal probes; ATTO 488/565/647N	COOH magnetic beads; amine-DNA capture probes	Calibration curves	PBS buffer	10^{-9}–10^{-15} M (RdRp, E); 10^{-9}–10^{-12} M (N)	1 fM (RdRp, E); 1 pM (N); down to 10 aM with Ag nanostars	~3 h hybridization (seconds for SERS readout)	Multiplex (3 genes), low false positives, high reproducibility	192
SARS-CoV-2 (N-gene cDNA)	Magnetic-responsive sandwich SERS with tunable hot spots (vertical gap modulation)	None	DTNB-AuNPs; DNA probes (DNA1–DNA4)	AuNP/rGO-modified Si wafer; DNA capture probes; SPION linkers	Calibration curves	TE buffer	1 fM–1 nM (buffer)	1 fM (no magnet); 100 aM (+magnet)	~4–6 h total	10× LOD improvement with magnetic modulation; reversible, high selectivity (single-base mismatch)	193
Dengue virus (DENV-4 DNA); host viral-infection biomarkers (e.g., RSAD2, IFI27/IFI44L)	Plasmonic molecular sentinel-on-chip (MSC / iMS-on-chip) SERS nanochip; distance-modulated signal-on/off DNA hybridization	None	Raman-labeled DNA stem-loop probes (e.g., Cy5)	Nanowave plasmonic chip (bimetallic MFON); thiolated DNA probes	Peak intensity comparison	Buffer solutions		~6 attomoles (DENV-4 DNA, probed area)	~1–2 h incubation	Single-step, wash-free; multiplexable; high reproducibility; large-area chip	194
Hepatitis B virus (HBV DNA)	Temperature-responsive sandwich hybridization SERS sensor	None	Free AuNPs + DNA-reporter strand; ICG Raman reporter	Hybrid Si–poly(MEO_2-MA)–AuNP chip; DNA capture probes	Calibration curves	PBS buffer; spiked serum	0.1 fM–6 µM (buffer)	0.44 fM at 25°C, 0.14 fM at 37°C	~4 h	High selectivity (single-base mismatch); reusable & stable; validated in serum	195

HIV-1 DNA	SERS-based lateral flow assay with sandwich DNA hybridization	None	AuNPs functionalized with detection DNA; MGITC Raman reporter	Nitrocellulose LF strip; streptavidin–biotin capture DNA	Calibration curves	SSC buffer; spiked samples	8 pg/mL–64 ng/mL	0.24 pg/mL	~15 min	Quantitative POC readout; ~1000× sensitivity vs colorimetric LF; high specificity	196
KSHV DNA; BA DNA (Kaposi's sarcoma–associated herpesvirus; Bacillary angiomatosis)	Multiplex SERS-based lateral flow assay (two test lines + control)	None	AuNPs functionalized with detection DNA; MGITC Raman reporter	Nitrocellulose LF strip; streptavidin–biotin capture DNAs	Calibration curves	SSC buffer	0.1–100 pM (buffer)	0.043 pM (KSHV), 0.074 pM (BA)	~20 min	Simultaneous dual-DNA detection; ~10^4× sensitivity vs colorimetric LF; high specificity	197
11 RTI pathogens (Influenza A/B, Parainfluenza 1–3, RSV, Adenovirus, *C. pneumoniae*, *C. burnetii*, *M. pneumoniae*, *L. pneumophila*)	SERS-encoded lateral flow microarray (2×3 dots) with multiplex sandwich DNA hybridization	None	Ag@Au core–shell SERS nanotags; DNA probes; NBA/MB Raman dyes	Nitrocellulose LFM; biotin–streptavidin capture DNAs	Calibration curves	TE buffer; human throat swab	1 pM–50 nM (buffer)	~0.03–0.041 pM (depending on pathogen)	~20 min (7 min flow + 13 min read)	11-plex on one strip; CV ≈ 9%; recoveries 93–108% in swabs	198
SARS-CoV-2 (RdRp/ORF1ab, E gene RNA)	Triple-mode biosensor: AuNP aggregation–based colorimetric + fluorescence + SERS readout	None	AuNPs (~17 nm); ssDNA probes; Cy3 Raman/fluorescence label		Calibration curves for all three modes	TE buffer; simulated throat swab	160 fM–1 nM (buffer)	395 fM	~40 min	Single-base mismatch discrimination; multi-site probes boost sensitivity; extraction-free	199
SARS-CoV-2 and Influenza A/H1N1	Dual-mode SERS DNA aptasensor for virus discrimination	None	DNA aptamers labeled with Cy3 (SARS-CoV-2) and RRX (H1N1); internal standard 4-MBA	Au nanopopcorn substrate; spike-protein & hemagglutinin aptamers	Calibration curves with peak ratios	Transport medium; spiked nasopharyngeal samples	0–5000 PFU/mL (SARS-CoV-2), 0–2016 HAU/mL (H1N1)	0.78 PFU/mL (SARS-CoV-2), 0.68 HAU/mL (H1N1)	~15 min incubation (+ readout)	Low cross-reactivity; quantitative duplex detection; RSD ≈ 5% with internal standard	200
SARS-CoV-2 (RNA, ORF1ab fragment)	SERS/EC dual-mode aptasensor based on single Au nanowire platform	None	Au@Ag core–shell NPs; RNA aptamer; 4-ATP (SERS) / methylene blue (EC)	Single Au nanowire electrode; SH-DNA / aptamer	Calibration curves	PBS; 1% human serum	10 pM–500 nM (buffer)	1.02 pM	~40 min incubation (+ readout)	Dual-mode cross-validation; high selectivity (single-base mismatch); RSD < 5%; serum recovery ~98%	201
Tobacco mosaic virus (TMV RNA)	Reagentless in situ SERS sensing hydrogel (direct leaf contact)	None	Ag-coated Au nanostar IMS probes; DNA hairpin probe; Cy5 reporter	Agar hydrogel embedded with SERS probes; no capture ligand	Peak intensity comparison	Leaf interstitial fluid (intact plants)	Qualitative / semi-quantitative (in planta)	1000 amol	<60 min	Field-deployable; no extraction; ROC AUC = 0.85 (susceptible plants); multiplexable potential	202

HIV-1 (gag gene DNA)	PCR + SERS gene probe (SERGene) using SERS-labeled primers and post-hybridization metal coating	PCR (30 cycles)	Cresyl fast violet (CFV)-labeled DNA primers	Polystyrene wells + thermally evaporated Ag film; immobilized DNA capture probe	Peak intensity comparison	Buffer (SSC hybridization media)	Not explicitly quantified	PCR-level sensitivity (qualitative)	PCR (~2–3 h) + hybridization/SERS	Extremely narrow Raman bandwidth (<1 nm); multiplexing potential (≤100 spots); non-radioactive probes	203
HIV-1 (gag gene DNA)	Plasmonic molecular sentinel (hairpin DNA) SERS nanoprobe; signal-off via hybridization-induced distance increase	PCR for initial amplification	AgNPs; hairpin DNA probe; R6G or TMR Raman label		Linear SERS intensity change due to quenching during hybridization	Phosphate buffer; PCR amplicons	0–2 µM (buffer, quenching response)	Not explicitly defined	2–3 h hybridization (~1 min for PCR-product readout)	Wash-free, homogeneous; high specificity; multiplexing potential via narrow Raman bands	204
MRSA biomarker genes (MecA, FemA DNA)	Real-time PCR–SERS on-chip with dialysis-mediated reporter transfer	PCR with TaqMan assay (real-time)	Aggregated AgNP colloid; TaqMan-like DNA probes; R6G (MecA), Cy3 (FemA)	Thermoplastic microfluidic chip with dialysis membrane separating PCR & SERS wells	Real-time monitoring of amplification progress based on SERS signal intensity; ratiometric peak analysis	PCR buffer	Qualitative real-time amplification curves	PCR-level sensitivity (10^5 copies demonstrated)	~2–3 h (≤60 cycles)	True real-time, single-well multiplexing; no post-PCR mixing; POC-compatible	205
Bacillus anthracis (pagA DNA marker)	SERS–PCR gene assay using bridge-probe hydrolysis and magnetic sandwich detection	PCR (5–20 cycles)	Hollow AuNP SERS nanotags; detection DNA; MGITC reporter	Streptavidin magnetic beads; biotinylated capture DNA	Linear regression of peak intensity vs DNA concentration	PCR buffer	0.1–1000 pM (after 20 cycles)	0.11 pM (20 cycles); 960 pM (5 cycles)	PCR ≤20 cycles (~30–60 min) + SERS readout	Lower LOD than RT-qPCR at low cycle numbers; wide dynamic range; CV < 10%	206
SARS-CoV-2 (E, RdRp genes)	SERS-PCR-based molecular diagnostic assay	RT-PCR (reduced cycles)	80 nm AuNP SERS nanotags; bridge DNA; MGITC reporter	Au nanodimple substrate (AuNDS); thiolated capture DNA	Calibration curve: ΔRaman intensity vs thermocycle number	PCR buffer	10^2–10^5 copies/µL	Detection after 8 cycles at 10^5 copies/µL (≈2.6×10^7 copies/µL)	≤1 h PCR + SERS	3×–5× fewer PCR cycles than RT-PCR; RSD ≈ 4.6%; higher SNR, reduced false negatives	207
Avian influenza A H7N9 (H7 & N9 gene DNA)	SERS detection using Exo III-assisted amplification	Exonuclease III-assisted cycling amplification	DNA probes; Cy5 (H7), ROX (N9) reporters	Ag nanorod array (AgNRs); thiolated capture DNA; MCH blocking	Linear calibration curve for SERS intensity vs log concentration	TM buffer	1 fM–100 pM	31 aM (H7), 44 aM (N9)	~7 h	Dual-gene detection; high specificity (single-base mismatch); RSD < 6.2%; recovery 93.8–106.2%	208
Influenza A virus (IAV, M gene DNA/RNA)	SERS-based lateral flow strip combined with ERA (Sandwich structure)	Enzymatic recombinase amplification (ERA)	SiO_2@Au nanoraspberries; streptavidin; DTNB	NC LF strip; anti-FAM test line; anti-SA control line	Sigmoidal curve of SERS intensity vs. DNA concentration logarithm	Buffer; pseudovirus	2.63×10^3 to 10^9 copies/mL	2.63×10^3 copies/mL	~25 min amplification + ~5 min lateral flow	Visual + SERS dual readout; high specificity vs IBV & SARS-CoV-2; RSD ~5–7%	209

SARS-CoV-2 RdRp gene	Dual-mode ECL/SERS ratiometric biosensor	Nt.BbvCI-assisted enzymatic cleavage	Ferrocene (Fc)-labeled DNA probe as Raman reporter/ECL quencher; Au NPs on COF/$CsPbBr_3$/Au provide SERS hotspots	COF/$CsPbBr_3$/Au-modified GCE; thiolated DNA tetrahedral molecular machine immobilized through Au–S bonds	SERS peak intensity of Fc bands; ECL/SERS ratiometric calibration vs log target concentration	Buffer; food packaging; pork; diluted serum	0.01 fM–1 pM	3.2 aM	~60 min after sensor assembly: 30 min target incubation + 30 min Nt.BbvCI reaction	RSD 2.72%; good mismatch selectivity; serum recovery 97.2–104.0%	210
HBV; HPV-16; HPV-18 (viral DNA)	CRISPR-Cas12a–assisted amplification-free SERS (trans-cleavage signal-off)	CRISPR-Cas12a–assisted amplification	RAuNPs (TAMRA-labeled AuNPs) linked by ssDNA	GO/triangle Au nanoflower (GO-TANF) nanoarray	Linear correlation between ΔRaman intensity and DNA concentration	Buffer	1 aM – 100 pM	1 aM (HBV DNA)	~20 min	Multiplex (3 targets); wide LDR; no washing/labeling after reaction; high selectivity	211
HPV-16, HPV-18 (viral dsDNA)	CRISPR/Cas12a–SERS aggregation assay (trans-cleavage–induced signal-off)	Cas12a collateral cleavage provides signal amplification	AuNPs@DNA@MBN probes; linker ssDNA; 4-mercaptobenzonitrile (MBN)	Colloidal AuNP probe pairs bridged by linker ssDNA	Linear calibration curve of intensity vs DNA concentration	Buffer; serum; pseudovirus	6.72×10^{-12} to 6.72×10^{-7} M	~6.72 pM	~40 min	Amplification-free; high specificity (single-base discrimination); universal via crRNA exchange; serum-compatible	212
Hepatitis A virus (HAV cDNA; foodborne virus model)	CRISPR/Cas12a-powered signal-off SERS biosensor	CRISPR-Cas12a with ssDNA cleavage	ssDNA–ROX reporter (thiolated)	Gold nanocones grown on nickel foam (Au–NF); ssDNA immobilization	Linear regression of ΔRaman intensity vs DNA concentration	Bottled water (food model); buffer	2.39×10^{-9} – 4.78×10^{-13} mol/L	8.23 fM	30 min	High specificity vs HBV/NoV; avoids Mg^{2+}-induced NP aggregation; recovery 82–108% in water	213
Chikungunya virus, CHIKV RNA	CRISPR/Cas13a-mediated SERS lateral flow immunoassay; lyophilized CRISPR/Cas13a platform; signal-off detection based on Cas13a cleavage of FAM–biotin RNA probes	RT-RAA isothermal amplification; T7 transcription; Cas13a collateral cleavage	Au/WOX nanowires; anti-FAM antibody; DTNB reporter	NC LF strip; streptavidin test line; goat anti-mouse IgG control line	Calibration curve of peak intensity vs target concentration; linear regression; ROC analysis for clinical samples	Buffer; inactivated CHIKV; clinical serum	0.5–1000 copies/µL (plasmid); 1–1000 copies/µL (inactivated CHIKV)	0.56 copies/µL (plasmid); 1.03 copies/µL (inactivated CHIKV)	~55 min	Visual + SERS dual readout; no cross-reactivity with DENV1–4, ZIKV, or YFV; RSD 5.72% and 2.1% at 80 and 20 copies/µL; 34 clinical serum samples showed 100% agreement with qPCR, AUC = 1, 100% sensitivity, and 100% specificity, though with limited sample size	214
African swine fever virus (ASFV, B646L dsDNA gene)	Background-free SCOPE-RCA SERS assay; CRISPR/Cas12a "select-before-amplification" releases RNA from	CRISPR/Cas12a trans-cleavage; rolling circle amplification (RCA); catalytic	AuNPs modified with Cy5-labeled hairpin DNAs H1/H2 via Au–S bonds; Cy5 as Raman reporter	Magnetic bead@DNA/RNA chimeric hairpin preprimer; Cas12a-crRNA recognition of ASFV dsDNA; CHA-induced AuNP	Calibration of peak intensity vs log concentration	Buffer; heat-treated swine serum/mock-infected	0.1 aM–100 fM	0.098 aM, equivalent to 59 copies/mL	~75 min minimum; 20 min Cas12a cleavage + ~55 min RCA/SER	High specificity against single-base mismatched ASFV DNA, PVR2 DNA, and PRV DNA; anti-interference in mixed dsDNA samples;	215

	DNA/RNA chimeric hairpin, followed by RCA-triggered CHA assembly of SERS probes	hairpin assembly (CHA)		network/hotspots used as SERS substrate		samples; ASFV pseudoviral nucleic acid reference			S amplification	intra-assay RSD 7.36%; detected some samples not identified by qPCR	
Dengue virus type 1 (DENV-1) nucleic acid	CRISPR/Cas12a-assisted signal-off SERS assay; target-triggered TWJ formation generates Trigger-strands, activates Cas12a, cleaves ssDNA Linker, prevents Probe1/Probe2 aggregation, and decreases SERS signal	Strand displacement amplification (SDA) + CRISPR/Cas12a collateral cleavage	AuNPs; SH-DNA1 or SH-DNA2; 4-mercaptobenzoic acid (4-MBA)		Linear regression vs log target concentration	Buffer; 10% FBS; spiked saliva/swab samples	0.1 pM–10 nM	4.68 fM		R^2 = 0.9942; uniformity RSD = 4.81%; reproducibility RSD = 4.83%; distinguished DENV-1 from DENV-2/3/4, ZIKV, CHIKV, and HCV cDNA; recovery 99.2–107.2% in 10% FBS	216
Norovirus GII-4 target RNA	CRISPR platform combining DNA hydrogel fishing strategy with fluorescence, colorimetric, and SERS readouts; target RNA activates CRISPR-Cas12a after amplification, degrading DNA hydrogel and releasing GCSNA probes	RT-RPA + CRISPR-Cas12a trans-cleavage; RCA used to prepare DNA hydrogel	Gap-containing spherical nucleic acid nanoparticles, GCSNAs; Au@Au nanogap particles modified with ssDNA-FAM; PATP Raman reporter embedded in internal nanogap	DNA hydrogel/GCSNA complex; RCA-generated DNA hydrogel captures GCSNAs	Calibration curve	Buffer; spiked strawberry and oyster samples	SERS: 0.001–1000 pM; fluorescence: 0.01–1000 pM; colorimetric: 0.001–100 pM	SERS: 0.16 fM; fluorescence: 0.5 fM; colorimetric: 83 aM		High specificity against random RNA and other diarrheal pathogens; recoveries in strawberry/oyster: fluorescence 91.81–117.17%, colorimetric 96.60–102.78%, SERS 93.72–107.08%; CV generally <6%; multimodal validation reduces false results	217
Influenza A virus H1N1 RNA	CRISPR/Cas13a-mediated SERS lateral flow assay	Multienzyme isothermal rapid amplification; T7 transcription before Cas13a reaction	SiO_2@Au core–shell SERS tags; anti-FAM antibody; DTNB reporter	LFA strip; T line: goat anti-mouse IgG; C line: streptavidin	Nonlinear calibration curve; ROC analysis for clinical samples	Buffer; clinical throat swabs	10–10^4 copies/µL	4.4 copies/µL	45 min	Specific vs RSV-A/B, HRV, HPIVs, SARS-CoV-2, IBV, H3N2; RSD 2.1–3.3%; 100% agreement with RT-qPCR for 51 clinical samples; AUC = 1	218
Dengue virus (DENV-2 gene DNA)	Cascade signal amplification-based SERS assay	LCHA (Localized Catalytic Hairpin Assembly) and HCR (Hybridization Chain Reaction)	ROX-labeled DNA hairpins (no NP tags)	Ag nanorod (AgNR) array with MCH blocking; thiolated DNA	Linear relationship: SERS intensity vs log DNA concentration	TM buffer	1 fM–10 nM	0.49 fM	~2.5 h	High specificity (single-base mismatch); good uniformity (RSD ≈ 8.8%); recoveries 93–114%	219

SARS-CoV-2 viral particles (S protein; pseudovirus)	One-pot SERS aptasensor with locking amplifier (virus-triggered activation)	SDA-based cyclic amplification (KF polymerase + Nt.BbvCI)	AuNP SERS probes; capture DNA + ROX-DNA reporter	Magnetic beads–AuNP complexes; aptamer recognition of S protein	Calibration curve: SERS intensity vs viral particle concentration	Tap water, river water, cold-chain fish samples	625–10000 TU/μL (environmental samples)	260 TU/μL	~40 min	High selectivity vs lentivirus; RSD ≈ 3–7%; recoveries 93–106%; minimal background	220
SARS-CoV-2 (ORF1ab RNA)	Non-enzymatic signal amplification-powered SERS	Catalytic Hairpin Assembly (CHA), enzyme-free	AuNPs functionalized with probe ssDNA + DTNB	Ag nanorod (AgNR) array chip; thiolated capture ssDNA + MCH blocking	Linear regression of I_T/I_{blank} vs log concentration	Buffer; extracted clinical RNA	10^2–10^6 copies/mL	51.38 copies/mL	~50 min	High selectivity vs Flu A/B, RSV, AdV; RSD ≤ 7.7%; multi-well portable chip	221
Hepatitis C virus (HCV) RNA	Enzyme-free catalytic hairpin assembly (CHA)-triggered plasmonic core–satellite assembly	Catalytic Hairpin Assembly (CHA)	AuNP satellites (10 or 20 nm) functionalized with DNA hairpins (H2/H3); Raman reporter: MGITC	60 nm AuNP core functionalized with DNA hairpin H1 (core–satellite SERS substrate via 3-way junction)	Logarithmic calibration curve of SERS intensity vs HCV RNA concentration	Buffer (CHA reaction buffer; aqueous)	100 fM–10 nM	1.706 fM (60–20 nm configuration)	~2–6 h (CHA + assembly; isothermal, 37 °C)	~10× sensitivity gain for 20 nm vs 10 nm satellites; pan-genotypic target (5′-UTR, 23-nt); enzyme-free	222
SARS-CoV-2 RNA	One-pot SERS assay using Cas13a-CHA-based strategy (Sandwich structure)	CRISPR/Cas13a collateral cleavage + catalytic hairpin assembly (CHA)	AuNPs (~30 nm); probe DNA (P); DTNB	Ag nanorod (AgNR) array; thiolated hairpin H1 (MCH blocked)	Linear calibration curve, SERS intensity vs log RNA concentration	Buffer; RNA from swab samples	10^3–10^8 copies/mL (buffer)	5.18×10^2 copies/mL	~60 min	Single-base mutation discrimination; no cross-reactivity with SARS-CoV, MERS-CoV, influenza, RSV; RSD < 6%	223
African swine fever virus (ASFV DNA; p72 gene)	Magnetic-bead SERS sandwich hybridization assay	Hybridization Chain Reaction (HCR)	AuNPs (30 nm); HCR DNA probes; DTNB	Streptavidin magnetic beads; biotin-DNA capture probes	Linear relationship of intensity vs concentration	Buffer; serum samples	10^2 to 10^{10} copies/mL	10^2 copies/mL	~15 min	High specificity vs SFV/PRRSV/JEV; RSD ≈ 5.6%; comparable to qRT-PCR without enzymatic amplification	224
SARS-CoV-2 variants (DNA targets; Omicron vs Delta)	Lyophilized reagentless SERS sensors, mix-and-detect (Signal-on)	DNA catalysis with trehalose stabilization	Ag@Au nanostars; stem-loop DNA probe; Cy5 reporter		Calibration curve of normalized SERS intensity vs DNA concentration	Buffer; 50% human saliva	0.5–10 nM	0.5 nM	~2 h incubation + readout	Shelf-life >35 days at RT after lyophilization; single-base discrimination; true one-pot reagentless assay	225

Table S10. Comparison of SERS-based viral detection with conventional viral diagnostic methods.

Method	Main target	Typical LOD/sensitivity	Specificity	Assay time	Multiplexing	Advantages	Limitations	Typical use
**RT-PCR / qPCR**	Viral RNA/DNA	Very high; often copies-level after amplification	Very high	~1–4 h	Moderate	Gold standard; high sensitivity and specificity	Requires extraction, reagents, thermal cycling, trained personnel	Laboratory confirmation, viral-load monitoring
**Isothermal amplification**	Viral RNA/DNA	High	High	~15–60 min	Moderate	Faster than PCR; simpler equipment	Primer design, contamination risk, matrix inhibition	POC or near-POC nucleic-acid testing
**ELISA**	Viral antigen or antibody	Moderate to high	High when antibodies/reagents are specific	~1–3 h	Low–moderate	Established, scalable, quantitative	Requires washing/incubation; delayed antibody response for serology	Antigen or antibody testing
**Lateral flow assay**	Viral antigen or antibody	Moderate	Moderate–high	~10–30 min	Limited	Low-cost, rapid, user-friendly	Lower sensitivity; often qualitative	Rapid screening
**SERS direct detection**	Intact virion or viral components	Reported from ~10 to 10^4 PFU/mL depending on substrate/matrix	Depends on spectral library and ML model	Minutes to <1 h after sample preparation	High potential	Label-free, molecular fingerprint, multiplexing potential	Matrix interference, reproducibility, hotspot accessibility	Rapid screening, virus classification
**SERS affinity-based**	Viral antigen, intact virion, RNA/DNA	Often improved over direct SERS; assay-dependent	High with specific receptors	~15 min–2 h	Moderate–high	Improved selectivity, target enrichment	Non-specific binding, receptor stability, surface chemistry	Targeted viral detection
**SERS labeled/tag-based**	Viral antigen, antibody, nucleic acid	Often very low LOD; assay-dependent	High with capture/detection probes	~15 min–2 h	High	Strong signal, multiplexing, quantitative potential	Tag synthesis, reporter stability, assay complexity	Sensitive and multiplexed detection

References

(1) Shanmukh, S.; Jones, L.; Driskell, J.; Zhao, Y.; Dluhy, R.; Tripp, R. A., Rapid and Sensitive Detection of Respiratory Virus Molecular Signatures Using a Silver Nanorod Array SERS Substrate. *Nano Letters* **2006,** *6* (11), 2630-2636.

(2) Shanmukh, S.; Jones, L.; Zhao, Y. P.; Driskell, J. D.; Tripp, R. A.; Dluhy, R. A., Identification and classification of respiratory syncytial virus (RSV) strains by surface-enhanced Raman spectroscopy and multivariate statistical techniques. *Analytical and Bioanalytical Chemistry* **2008,** *390* (6), 1551-1555.

(3) Driskell, J. D.; Shanmukh, S.; Liu, Y.-J.; Hennigan, S.; Jones, L.; Zhao, Y.-P.; Dluhy, R. A.; Krause, D. C.; Tripp, R. A., Infectious agent detection with SERS-active silver nanorod arrays prepared by oblique angle deposition. *IEEE Sensors Journal* **2008,** *8* (5-6), 863-870.

(4) Abell, J. L.; Driskell, J. D.; Dluhy, R. A.; Tripp, R. A.; Zhao, Y. P., Fabrication and characterization of a multiwell array SERS chip with biological applications. *Biosensors and Bioelectronics* **2009,** *24* (12), 3663-3670.

(5) Demirel, M. C.; Kao, P.; Malvadkar, N.; Wang, H.; Gong, X.; Poss, M.; Allara, D. L., Bio-organism sensing via surface enhanced Raman spectroscopy on controlled metal/polymer nanostructured substrates. *Biointerphases* **2009,** *4* (2), 35-41.

(6) Driskell, J. D.; Zhu, Y.; Kirkwood, C. D.; Zhao, Y.; Dluhy, R. A.; Tripp, R. A., Rapid and Sensitive Detection of Rotavirus Molecular Signatures Using Surface Enhanced Raman Spectroscopy. *PLOS ONE* **2010,** *5* (4), e10222.

(7) Fan, C.; Hu, Z.; Riley, L. K.; Purdy, G. A.; Mustapha, A.; Lin, M., Detecting Food- and Waterborne Viruses by Surface-Enhanced Raman Spectroscopy. *Journal of Food Science* **2010,** *75* (5), M302-M307.

(8) Chang, C.-W.; Liao, J.-D.; Shiau, A.-L.; Yao, C.-K., Non-labeled virus detection using inverted triangular Au nano-cavities arrayed as SERS-active substrate. *Sensors and Actuators B: Chemical* **2011,** *156* (1), 471-478.

(9) Lin, Y.-Y.; Liao, J.-D.; Yang, M.-L.; Wu, C.-L., Target-size embracing dimension for sensitive detection of viruses with various sizes and influenza virus strains. *Biosensors and Bioelectronics* **2012,** *35* (1), 447-451.

(10) Chunyuan, S.; Jeremy, D. D.; Ralph, A. T.; Yiping, C.; Yiping, Z., The use of a handheld Raman system for virus detection. *Chemical, Biological, Radiological, Nuclear, and Explosives (CBRNE) Sensing XIII, Proc. SPIE* **2012,** *8358*, 83580I, https://doi.org/10.1117/12.918758.

(11) Shao, F.; Lu, Z.; Liu, C.; Han, H.; Chen, K.; Li, W.; He, Q.; Peng, H.; Chen, J., Hierarchical Nanogaps within Bioscaffold Arrays as a High-Performance SERS Substrate for Animal Virus Biosensing. *ACS Applied Materials & Interfaces* **2014,** *6* (9), 6281-6289.

(12) Yang, S.; Slotcavage, D.; Mai, J. D.; Guo, F.; Li, S.; Zhao, Y.; Lei, Y.; Cameron, C. E.; Huang, T. J., Electrochemically created highly surface roughened Ag nanoplate arrays for SERS biosensing applications. *Journal of Materials Chemistry C* **2014,** *2* (39), 8350-8356.

(13) Luo, Z.; Chen, L.; Liang, C.; Wei, Q.; Chen, Y.; Wang, J., Porous carbon films decorated with silver nanoparticles as a sensitive SERS substrate, and their application to virus identification. *Microchimica Acta* **2017,** *184* (9), 3505-3511.

(14) Durmanov, N. N.; Guliev, R. R.; Eremenko, A. V.; Boginskaya, I. A.; Ryzhikov, I. A.; Trifonova, E. A.; Putlyaev, E. V.; Mukhin, A. N.; Kalnov, S. L.; Balandina, M. V.; Tkachuk, A. P.; Gushchin, V. A.; Sarychev, A. K.; Lagarkov, A. N.; Rodionov, I. A.; Gabidullin, A. R.; Kurochkin, I. N., Non-labeled selective virus detection with novel SERS-active porous silver nanofilms fabricated by Electron Beam Physical Vapor Deposition. *Sensors and Actuators B: Chemical* **2018,** *257*, 37-47.

(15) Wang, Y.; Yu, Y.; Liu, Y.; Yang, S., Template-Confined Site-Specific Electrodeposition of Nanoparticle Cluster-in-Bowl Arrays as Surface Enhanced Raman Spectroscopy Substrates. *ACS Sensors* **2018,** *3* (11), 2343-2350.

(16) Zhang, X.; Zhang, X.; Luo, C.; Liu, Z.; Chen, Y.; Dong, S.; Jiang, C.; Yang, S.; Wang, F.; Xiao, X., Volume-Enhanced Raman Scattering Detection of Viruses. *Small* **2019,** *15* (11), 1805516.
(17) Paria, D.; Kwok, K. S.; Raj, P.; Zheng, P.; Gracias, D. H.; Barman, I., Label-Free Spectroscopic SARS-CoV-2 Detection on Versatile Nanoimprinted Substrates. *Nano Letters* **2022,** *22* (9), 3620-3627.
(18) Sitjar, J.; Xu, H.-Z.; Liu, C.-Y.; Wang, J.-R.; Liao, J.-D.; Tsai, H.-P.; Lee, H.; Liu, B. H.; Chang, C.-W., Synergistic surface-enhanced Raman scattering effect to distinguish live SARS-CoV-2 S pseudovirus. *Analytica Chimica Acta* **2022,** *1193*, 339406.
(19) Yang, Y.; Xu, B.; Murray, J.; Haverstick, J.; Chen, X.; Tripp, R. A.; Zhao, Y., Rapid and quantitative detection of respiratory viruses using surface-enhanced Raman spectroscopy and machine learning. *Biosensors and Bioelectronics* **2022,** *217*, 114721.
(20) Hwang, C. S. H.; Lee, S.; Lee, S.; Kim, H.; Kang, T.; Lee, D.; Jeong, K.-H., Highly Adsorptive Au-TiO2 Nanocomposites for the SERS Face Mask Allow the Machine-Learning-Based Quantitative Assay of SARS-CoV-2 in Artificial Breath Aerosols. *ACS Applied Materials & Interfaces* **2022,** *14* (49), 54550-54557.
(21) Berus, S. M.; Nowicka, A. B.; Wieruszewska, J.; Niciński, K.; Kowalska, A. A.; Szymborski, T. R.; Dróżdż, I.; Borowiec, M.; Waluk, J.; Kamińska, A., SERS Signature of SARS-CoV-2 in Saliva and Nasopharyngeal Swabs: Towards Perspective COVID-19 Point-of-Care Diagnostics. *International Journal of Molecular Sciences* **2023,** *24* (11), 9706.
(22) Yadav, S.; Senapati, S.; Kulkarni, S. S.; Singh, J. P., A SERS based clinical study on HIV-1 viral load quantification and determination of disease prognosis. *Journal of Photochemistry and Photobiology B: Biology* **2023,** *239*, 112629.
(23) Yeh, Y.-T.; Gulino, K.; Zhang, Y.; Sabestien, A.; Chou, T.-W.; Zhou, B.; Lin, Z.; Albert, I.; Lu, H.; Swaminathan, V.; Ghedin, E.; Terrones, M., A rapid and label-free platform for virus capture and identification from clinical samples. *Proceedings of the National Academy of Sciences* **2020,** *117* (2), 895-901.
(24) Ye, J.; Yeh, Y.-T.; Xue, Y.; Wang, Z.; Zhang, N.; Liu, H.; Zhang, K.; Ricker, R.; Yu, Z.; Roder, A.; Perea Lopez, N.; Organtini, L.; Greene, W.; Hafenstein, S.; Lu, H.; Ghedin, E.; Terrones, M.; Huang, S.; Huang, S. X., Accurate virus identification with interpretable Raman signatures by machine learning. *Proceedings of the National Academy of Sciences* **2022,** *119* (23), e2118836119.
(25) Prigoda, K.; Ermina, A.; Bolshakov, V.; Tabarov, A.; Levitskii, V.; Andreeva, O.; Gazizulin, A.; Pavlov, S.; Danilenko, D.; Vitkin, V.; Zharova, Y., Dendritic Ag@c-Si structure for influenza A virus detection by SERS and machine learning. *Optical Materials* **2024,** *149*, 114977.
(26) Szymborski, T. R.; Berus, S. M.; Nowicka, A. B.; Słowiński, G.; Kamińska, A., Machine Learning for COVID-19 Determination Using Surface-Enhanced Raman Spectroscopy. *Biomedicines* **2024,** *12* (1), 167.
(27) Waiwijit, U.; Eiamchai, P.; Limwichean, S.; Horprathum, M.; Prommool, T.; Puttikhunt, C.; Songjaeng, A.; Kaewjiw, N.; Prayongkul, D.; Malasit, P.; Avirutnan, P.; Noisakran, S.; Nuntawong, N., Discrimination of Dengue Diseases in Children Using Surface-Enhanced Raman Spectroscopy Coupled with Machine Learning Approaches. *Analytical Chemistry* **2025,** *97* (28), 15122-15131.
(28) Senapati, S.; Kaushik, A.; Rajan; Singh, A.; Gupta, I.; Virkar, R.; Kulkarni, S. S.; Arankalle, V.; Singh, J. P., Rapid, On-Site SARS-CoV-2 Variant Detection and Differentiation Using GLAD-Pristine Silver Nanorod Arrays and Machine Learning-Enhanced SERS. *ACS Omega* **2025,** *10* (39), 44978-44988.
(29) Yadav, A.; Naik, R.; Gupta, E.; Roy, P. P.; Srivastava, S. K., Single-shot, receptor-free, rapid detection and classification of five respiratory viruses by machine learning integrated SERS sensing platform. *Biosensors and Bioelectronics* **2025,** *279*, 117394.
(30) Kang, H.; Lee, J.; Lee, S. H.; Jeon, J.; Mun, C.; Yang, J.-Y.; Seo, D.; Kwon, H.-J.; Lee, I.-C.; Kim, S.; Lim, E.-K.; Jung, J.; Jung, Y.; Park, S.-G.; Ryu, S.; Kang, T., Interpretability-driven

deep learning for SERS-based classification of respiratory viruses. *Biosensors and Bioelectronics* **2025,** *289*, 117891.
(31) Sitjar, J.; Tsai, H.-P.; Lee, H.; Chang, C.-W.; Wu, X.-N.; Liao, J.-D., Extracting the SERS characteristic features of SARS-CoV-2 and using machine learning to quickly screen positive specimens among hospitalized patients. *Talanta* **2026,** *297*, 128621.
(32) Ma, Y.; Wong, M.-C.; Song, M.; Wang, P.; Liu, Y.; Zhao, Y.; Chen, H.; Liu, J.; Hao, J., Machine Learning-Assisted 3D SERS Chip with Acoustic Enrichment for High-Accuracy Diagnosis of Respiratory Viruses and Emerging Pathogens. *ACS Sensors* **2025,** *10* (10), 7886-7898.
(33) Kim, S.; Lee, S.; Chi, H.-Y.; Kim, M.-K.; Kim, J.-S.; Lee, S.-H.; Chung, H., Feasibility Study for Detection of Turnip yellow mosaic virus (TYMV) Infection of Chinese Cabbage Plants Using Raman Spectroscopy. *The Plant Pathology Journal* **2013,** *29* (1), 105-109.
(34) Lim, J.-y.; Nam, J.-s.; Yang, S.-e.; Shin, H.; Jang, Y.-h.; Bae, G.-U.; Kang, T.; Lim, K.-i.; Choi, Y., Identification of Newly Emerging Influenza Viruses by Surface-Enhanced Raman Spectroscopy. *Analytical Chemistry* **2015,** *87* (23), 11652-11659.
(35) Zhang, Z.; Jiang, S.; Wang, X.; Dong, T.; Wang, Y.; Li, D.; Gao, X.; Qu, Z.; Li, Y., A novel enhanced substrate for label-free detection of SARS-CoV-2 based on surface-enhanced Raman scattering. *Sensors and Actuators B: Chemical* **2022,** *359*, 131568.
(36) Zhang, Z.; Li, D.; Wang, X.; Wang, Y.; Lin, J.; Jiang, S.; Wu, Z.; He, Y.; Gao, X.; Zhu, Z.; Xiao, Y.; Qu, Z.; Li, Y., Rapid detection of viruses: Based on silver nanoparticles modified with bromine ions and acetonitrile. *Chemical Engineering Journal* **2022,** *438*, 135589.
(37) Lv, X.; Zhang, Z.; Zhao, Y.; Sun, X.; Jiang, H.; Zhang, S.; Sun, X.; Qiu, X.; Li, Y., Label-free detection of virus based on surface-enhanced Raman scattering. *Spectrochimica Acta Part A: Molecular and Biomolecular Spectroscopy* **2023,** *302*, 123087.
(38) Guo, L.; Wang, Q.; Xing, Y.; Zhao, X.; Ma, R.; Liu, D.; Gao, P.; Li, Y., Self-Assembled Plasmonic Magnifier: A New Platform for Ultra-Sensitive Detection of Respiratory Viruses Using Surface-Enhanced Raman Spectroscopy. *Analytical Chemistry* **2025,** *97* (40), 21891-21904.
(39) He, S.; Cao, S.; Yuan, J.; Yu, Z.; Liu, Y.; Wu, Y.; Weng, S.; Zong, M.; Lin, D., Fast identification of influenza using label-free SERS combined with machine learning algorithms via clinical nasal swab samples. *Analytical Methods* **2025,** *17* (29), 6117-6123.
(40) Karunakaran, V.; Joseph, M. M.; Yadev, I.; Sharma, H.; Shamna, K.; Saurav, S.; Sreejith, R. P.; Anand, V.; Beegum, R.; Regi David, S.; Iype, T.; Sarada Devi, K. L.; Nizarudheen, A.; Sharmad, M. S.; Sharma, R.; Mukhiya, R.; Thouti, E.; Yoosaf, K.; Joseph, J.; Sujatha Devi, P.; Savithri, S.; Agarwal, A.; Singh, S.; Maiti, K. K., A non-invasive ultrasensitive diagnostic approach for COVID-19 infection using salivary label-free SERS fingerprinting and artificial intelligence. *Journal of Photochemistry and Photobiology B: Biology* **2022,** *234*, 112545.
(41) Peng, Y.; Lin, C.; Li, Y.; Gao, Y.; Wang, J.; He, J.; Huang, Z.; Liu, J.; Luo, X.; Yang, Y., Identifying infectiousness of SARS-CoV-2 by ultra-sensitive SnS2 SERS biosensors with capillary effect. *Matter* **2022,** *5* (2), 694-709.
(42) Ganesh, S.; Dhinakaran, A. K.; Premnath, P.; Venkatakrishnan, K.; Tan, B., Label-Free Saliva Test for Rapid Detection of Coronavirus Using Nanosensor-Enabled SERS. *Bioengineering* **2023,** *10* (3), 391.
(43) Deng, H.; He, Q.; Xu, Z.; Wang, X.; Sheng, R., The study of Turnip Mosaic virus coat protein by surface enhanced Raman spectroscopy. *Spectrochimica Acta Part A: Molecular Spectroscopy* **1993,** *49* (12), 1709-1714.
(44) Sanchez, J. E.; Jaramillo, S. A.; Settles, E.; Velazquez Salazar, J. J.; Lehr, A.; Gonzalez, J.; Rodríguez Aranda, C.; Navarro-Contreras, H. R.; Raniere, M. O.; Harvey, M.; Wagner, D. M.; Koppisch, A.; Kellar, R.; Keim, P.; Jose Yacaman, M., Detection of SARS-CoV-2 and its S and N proteins using surface enhanced Raman spectroscopy. *RSC Advances* **2021,** *11* (41), 25788-25794.
(45) Huang, J.; Wen, J.; Zhou, M.; Ni, S.; Le, W.; Chen, G.; Wei, L.; Zeng, Y.; Qi, D.; Pan, M.; Xu, J.; Wu, Y.; Li, Z.; Feng, Y.; Zhao, Z.; He, Z.; Li, B.; Zhao, S.; Zhang, B.; Xue, P.; He, S.; Fang, K.; Zhao, Y.; Du, K., On-Site Detection of SARS-CoV-2 Antigen by Deep Learning-Based

Surface-Enhanced Raman Spectroscopy and Its Biochemical Foundations. *Analytical Chemistry* **2021,** *93* (26), 9174-9182.
(46) Peng, Y.; Lin, C.; Long, L.; Masaki, T.; Tang, M.; Yang, L.; Liu, J.; Huang, Z.; Li, Z.; Luo, X.; Lombardi, J. R.; Yang, Y., Charge-Transfer Resonance and Electromagnetic Enhancement Synergistically Enabling MXenes with Excellent SERS Sensitivity for SARS-CoV-2 S Protein Detection. *Nano-Micro Letters* **2021,** *13* (1), 52.
(47) Chien, T.-H.; Chiu, Y. L.; Lee, C.-C.; Hsiao, W. W.-W.; Chiang, W.-H., Plasma-Engineered Anisotropic Nanostructures with Multipolar Plasmon Coupling for Label-Free Biosensing. *ACS Applied Nano Materials* **2025,** *8* (43), 20801-20811.
(48) Gao, Y.; Jiang, Z.; Ioannou, N. K.; Kotsifaki, D. G., SERS Detection of SARS-CoV-2 Spike Proteins and IgG Antibodies Using Metallic H-type Nano-Apertures. *Advanced Physics Research* **2025,** *4* (12), e00143.
(49) Chen, L.; Zeng, X.; Yang, F.; Yang, T.; Chen, Y.; Zhao, Y.; Luo, X.; Li, Y., Rapid and Sensitive Detection of Mutations in SARS-CoV-2 by Surface-Enhanced Raman Spectroscopy. *Langmuir* **2025,** *41* (5), 3583-3591.
(50) Leonardi, A. A.; Sciuto, E. L.; Lo Faro, M. J.; Morganti, D.; Midiri, A.; Spinella, C.; Conoci, S.; Irrera, A.; Fazio, B., Molecular Fingerprinting of the Omicron Variant Genome of SARS-CoV-2 by SERS Spectroscopy. *Nanomaterials* **2022,** *12* (13), 2134.
(51) Li, F., Structure, Function, and Evolution of Coronavirus Spike Proteins. *Annual Review of Virology* **2016,** *3* (1), 237-261.
(52) Anderson, E. M.; Goodwin, E. C.; Verma, A.; Arevalo, C. P.; Bolton, M. J.; Weirick, M. E.; Gouma, S.; McAllister, C. M.; Christensen, S. R.; Weaver, J.; Hicks, P.; Manzoni, T. B.; Oniyide, O.; Ramage, H.; Mathew, D.; Baxter, A. E.; Oldridge, D. A.; Greenplate, A. R.; Wu, J. E.; Alanio, C.; D'Andrea, K.; Kuthuru, O.; Dougherty, J.; Pattekar, A.; Kim, J.; Han, N.; Apostolidis, S. A.; Huang, A. C.; Vella, L. A.; Kuri-Cervantes, L.; Pampena, M. B.; Betts, M. R.; Wherry, E. J.; Meyer, N. J.; Cherry, S.; Bates, P.; Rader, D. J.; Hensley, S. E., Seasonal human coronavirus antibodies are boosted upon SARS-CoV-2 infection but not associated with protection. *Cell* **2021,** *184* (7), 1858-1864.e10.
(53) Hulswit, R. J. G.; Lang, Y.; Bakkers, M. J. G.; Li, W.; Li, Z.; Schouten, A.; Ophorst, B.; van Kuppeveld, F. J. M.; Boons, G.-J.; Bosch, B.-J.; Huizinga, E. G.; de Groot, R. J., Human coronaviruses OC43 and HKU1 bind to 9-O-acetylated sialic acids via a conserved receptor-binding site in spike protein domain A. *Proceedings of the National Academy of Sciences* **2019,** *116* (7), 2681.
(54) Fehr, A. R.; Perlman, S., Coronaviruses: An Overview of Their Replication and Pathogenesis. In *Coronaviruses: Methods and Protocols*, Maier, H. J.; Bickerton, E.; Britton, P., Eds. Springer New York: New York, NY, 2015; pp 1-23.
(55) Liljander, A.; Meyer, B.; Jores, J.; Müller, M. A.; Lattwein, E.; Njeru, I.; Bett, B.; Drosten, C.; Corman, V. M. J. E. i. d., MERS-CoV antibodies in humans, Africa, 2013–2014. *Emerging Infectious Diseases* **2016,** *22* (6), 1086-1089.
(56) Ge, X.-Y.; Li, J.-L.; Yang, X.-L.; Chmura, A. A.; Zhu, G.; Epstein, J. H.; Mazet, J. K.; Hu, B.; Zhang, W.; Peng, C.; Zhang, Y.-J.; Luo, C.-M.; Tan, B.; Wang, N.; Zhu, Y.; Crameri, G.; Zhang, S.-Y.; Wang, L.-F.; Daszak, P.; Shi, Z.-L., Isolation and characterization of a bat SARS-like coronavirus that uses the ACE2 receptor. *Nature* **2013,** *503* (7477), 535-538.
(57) Cho, S.-J.; Woo, H.-M.; Kim, K.-S.; Oh, J.-W.; Jeong, Y.-J., Novel system for detecting SARS coronavirus nucleocapsid protein using an ssDNA aptamer. *Journal of Bioscience and Bioengineering* **2011,** *112* (6), 535-540.
(58) Ahn, D.-G.; Jeon, I.-J.; Kim, J. D.; Song, M.-S.; Han, S.-R.; Lee, S.-W.; Jung, H.; Oh, J.-W., RNA aptamer-based sensitive detection of SARS coronavirus nucleocapsid protein. *Analyst* **2009,** *134* (9), 1896-1901.

(59) Chen, H.; Das, A.; Bi, L.; Choi, N.; Moon, J.-I.; Wu, Y.; Park, S.; Choo, J., Recent advances in surface-enhanced Raman scattering-based microdevices for point-of-care diagnosis of viruses and bacteria. *Nanoscale* **2020,** *12* (42), 21560-21570.
(60) Siracusano, G.; Pastori, C.; Lopalco, L., Humoral Immune Responses in COVID-19 Patients: A Window on the State of the Art. *Frontiers in Immunology* **2020,** *11*, 1049.
(61) Gupta, A.; Anand, A.; Jain, N.; Goswami, S.; Anantharaj, A.; Patil, S.; Singh, R.; Kumar, A.; Shrivastava, T.; Bhatnagar, S.; Medigeshi, G. R.; Sharma, T. K., A novel G-quadruplex aptamer-based spike trimeric antigen test for the detection of SARS-CoV-2. *Molecular Therapy - Nucleic Acids* **2021,** *26*, 321-332.
(62) Song, Y.; Song, J.; Wei, X.; Huang, M.; Sun, M.; Zhu, L.; Lin, B.; Shen, H.; Zhu, Z.; Yang, C., Discovery of Aptamers Targeting the Receptor-Binding Domain of the SARS-CoV-2 Spike Glycoprotein. *Analytical Chemistry* **2020,** *92* (14), 9895-9900.
(63) Li, J.; Zhang, Z.; Gu, J.; Stacey, H. D.; Ang, J. C.; Capretta, A.; Filipe, C. D. M.; Mossman, K. L.; Balion, C.; Salena, Bruno J.; Yamamura, D.; Soleymani, L.; Miller, M. S.; Brennan, John D.; Li, Y., Diverse high-affinity DNA aptamers for wild-type and B.1.1.7 SARS-CoV-2 spike proteins from a pre-structured DNA library. *Nucleic Acids Research* **2021,** *49* (13), 7267-7279.
(64) Kacherovsky, N.; Yang, L. F.; Dang, H. V.; Cheng, E. L.; Cardle, I. I.; Walls, A. C.; McCallum, M.; Sellers, D. L.; DiMaio, F.; Salipante, S. J., Discovery and characterization of spike N-terminal domain-binding Aptamers for rapid SARS-CoV-2 detection. *Angewandte Chemie* **2021,** *133* (39), 21381-21385.
(65) Li, W., The Hepatitis B Virus Receptor. *Annual Review of Cell and Developmental Biology* **2015,** *31* (1), 125-147.
(66) Nishioka, K.; Levin, A. G.; Simons, M. J., Hepatitis B antigen, antigen subtypes, and hepatitis B antibody in normal subjects and patients with liver disease. *Bull World Health Organ* **1975,** *52* (3), 293-300.
(67) Xi, Z.; Huang, R.; Li, Z.; He, N.; Wang, T.; Su, E.; Deng, Y., Selection of HBsAg-Specific DNA Aptamers Based on Carboxylated Magnetic Nanoparticles and Their Application in the Rapid and Simple Detection of Hepatitis B Virus Infection. *ACS Applied Materials & Interfaces* **2015,** *7* (21), 11215-11223.
(68) Liu, Y.; Le, C.; Tyrrell, D. L.; Le, X. C.; Li, X.-F., Aptamer Binding Assay for the E Antigen of Hepatitis B Using Modified Aptamers with G-Quadruplex Structures. *Analytical Chemistry* **2020,** *92* (9), 6495-6501.
(69) Liu, Y.; Wang, C.; Li, F.; Shen, S.; Tyrrell, D. L. J.; Le, X. C.; Li, X.-F., DNase-Mediated Single-Cycle Selection of Aptamers for Proteins Blotted on a Membrane. *Analytical Chemistry* **2012,** *84* (18), 7603-7606.
(70) Cocquerel, L.; Voisset, C.; Dubuisson, J., Hepatitis C virus entry: potential receptors and their biological functions. *Journal of General Virology* **2006,** *87* (5), 1075-1084.
(71) Chen, F.; Hu, Y.; Li, D.; Chen, H.; Zhang, X.-L., CS-SELEX Generates High-Affinity ssDNA Aptamers as Molecular Probes for Hepatitis C Virus Envelope Glycoprotein E2. *PLOS ONE* **2009,** *4* (12), e8142.
(72) García-Sastre, A., Influenza Virus Receptor Specificity: Disease and Transmission. *The American Journal of Pathology* **2010,** *176* (4), 1584-1585.
(73) Kanekiyo, M.; Wei, C.-J.; Yassine, H. M.; McTamney, P. M.; Boyington, J. C.; Whittle, J. R. R.; Rao, S. S.; Kong, W.-P.; Wang, L.; Nabel, G. J., Self-assembling influenza nanoparticle vaccines elicit broadly neutralizing H1N1 antibodies. *Nature* **2013,** *499* (7456), 102-106.
(74) Bhardwaj, J.; Chaudhary, N.; Kim, H.; Jang, J., Subtyping of influenza A H1N1 virus using a label-free electrochemical biosensor based on the DNA aptamer targeting the stem region of HA protein. *Analytica Chimica Acta* **2019,** *1064*, 94-103.
(75) Wang, R.; Zhao, J.; Jiang, T.; Kwon, Y. M.; Lu, H.; Jiao, P.; Liao, M.; Li, Y., Selection and characterization of DNA aptamers for use in detection of avian influenza virus H5N1. *Journal of Virological Methods* **2013,** *189* (2), 362-369.

(76) Kang, J.; Yeom, G.; Jang, H.; Park, C.-J.; Kim, M.-G., Highly sensitive and universal detection strategy based on a colorimetric assay using target-specific heterogeneous sandwich DNA aptamer. *Analytica Chimica Acta* **2020,** *1123*, 73-80.
(77) Nguyen, V.-T.; Seo, H. B.; Kim, B. C.; Kim, S. K.; Song, C.-S.; Gu, M. B., Highly sensitive sandwich-type SPR based detection of whole H5Nx viruses using a pair of aptamers. *Biosensors and Bioelectronics* **2016,** *86*, 293-300.
(78) Kim, S. H.; Lee, J.; Lee, B. H.; Song, C.-S.; Gu, M. B., Specific detection of avian influenza H5N2 whole virus particles on lateral flow strips using a pair of sandwich-type aptamers. *Biosensors and Bioelectronics* **2019,** *134*, 123-129.
(79) Schiller, J. T.; Day, P. M.; Kines, R. C., Current understanding of the mechanism of HPV infection. *Gynecologic Oncology* **2010,** *118* (1, Supplement 1), S12-S17.
(80) Leija-Montoya, A. G.; Benítez-Hess, M. L.; Toscano-Garibay, J. D.; Alvarez-Salas, L. M., Characterization of an RNA Aptamer Against HPV-16 L1 Virus-Like Particles. *Nucleic Acid Therapeutics* **2014,** *24* (5), 344-355.
(81) Gourronc, F. A.; Rockey, W. M.; Thiel, W. H.; Giangrande, P. H.; Klingelhutz, A. J., Identification of RNA aptamers that internalize into HPV-16 E6/E7 transformed tonsillar epithelial cells. *Virology* **2013,** *446* (1), 325-333.
(82) Carrington, M.; Dean, M.; Martin, M. P.; O'Brien, S. J., Genetics of HIV-1 Infection: Chemokine Receptor Ccr5 Polymorphism and Its Consequences. *Human Molecular Genetics* **1999,** *8* (10), 1939-1945.
(83) Yamamoto, R.; Katahira, M.; Nishikawa, S.; Baba, T.; Taira, K.; Kumar, P. K. R., A novel RNA motif that binds efficiently and specifically to the Tat protein of HIV and inhibits the trans-activation by Tat of transcription in vitro and in vivo. *Genes to Cells* **2000,** *5* (5), 371-388.
(84) Tripp, R. A.; Dluhy, R. A.; Zhao, Y., Novel nanostructures for SERS biosensing. *Nano Today* **2008,** *3* (3-4), 31-37.
(85) Fan, Z.; Yust, B.; Nellore, B. P. V.; Sinha, S. S.; Kanchanapally, R.; Crouch, R. A.; Pramanik, A.; Chavva, S. R.; Sardar, D.; Ray, P. C., Accurate Identification and Selective Removal of Rotavirus Using a Plasmonic–Magnetic 3D Graphene Oxide Architecture. *The Journal of Physical Chemistry Letters* **2014,** *5* (18), 3216-3221.
(86) Paul, A. M.; Fan, Z.; Sinha, S. S.; Shi, Y.; Le, L.; Bai, F.; Ray, P. C., Bioconjugated Gold Nanoparticle Based SERS Probe for Ultrasensitive Identification of Mosquito-Borne Viruses Using Raman Fingerprinting. *Journal of Physical Chemistry C* **2015,** *119* (41), 23669-23675.
(87) Palermo, G.; Rippa, M.; Conti, Y.; Vestri, A.; Castagna, R.; Fusco, G.; Suffredini, E.; Zhou, J.; Zyss, J.; De Luca, A.; Petti, L., Plasmonic Metasurfaces Based on Pyramidal Nanoholes for High-Efficiency SERS Biosensing. *ACS Applied Materials & Interfaces* **2021,** *13* (36), 43715-43725.
(88) Rippa, M.; Marchesano, V.; Vestri, A.; Sagnelli, D.; Fusco, G.; Zyss, J.; Lin, D.; Zhou, J.; Petti, L., Fractal Plasmonic Molecule for Multi-Sensing: SERS Platform for SARS-CoV-2 Detection. *ACS Applied Nano Materials* **2024,** *7* (7), 6958-6968.
(89) Reyes, M.; Piotrowski, M.; Ang, S. K.; Chan, J.; He, S.; Chu, J. J. H.; Kah, J. C. Y., Exploiting the Anti-Aggregation of Gold Nanostars for Rapid Detection of Hand, Foot, and Mouth Disease Causing Enterovirus 71 Using Surface-Enhanced Raman Spectroscopy. *Analytical Chemistry* **2017,** *89* (10), 5373-5381.
(90) Li, Y.; Lin, C.; Peng, Y.; He, J.; Yang, Y., High-sensitivity and point-of-care detection of SARS-CoV-2 from nasal and throat swabs by magnetic SERS biosensor. *Sensors and Actuators B: Chemical* **2022,** *365*, 131974.
(91) Yang, Y.; Cui, J.; Luo, D.; Murray, J.; Chen, X.; Hülck, S.; Tripp, R. A.; Zhao, Y., Rapid Detection of SARS-CoV-2 Variants Using an Angiotensin-Converting Enzyme 2-Based Surface-Enhanced Raman Spectroscopy Sensor Enhanced by CoVari Deep Learning Algorithms. *ACS Sensors* **2024,** *9* (6), 3158-3169.

(92) Payne, T. D.; Klawa, S. J.; Jian, T.; Kim, S. H.; Papanikolas, M. J.; Freeman, R.; Schultz, Z. D., Catching COVID: Engineering Peptide-Modified Surface-Enhanced Raman Spectroscopy Sensors for SARS-CoV-2. *ACS Sensors* **2021,** *6* (9), 3436-3444.
(93) Park, K. S.; Choi, A.; Kim, H. J.; Park, I.; Eom, M.-S.; Yeo, S.-G.; Son, R. G.; Park, T.-I.; Lee, G.; Soh, H. T.; Hong, Y.; Pack, S. P., Ultra-sensitive label-free SERS biosensor with high-throughput screened DNA aptamer for universal detection of SARS-CoV-2 variants from clinical samples. *Biosensors and Bioelectronics* **2023,** *228*, 115202.
(94) Wang, K.-S.; Kuan, T.-Y.; Chen, Y.-C.; Chu, Y.-J.; Chen, J.-S.; Chen, C.-C.; Liu, T.-Y., Simultaneous detection of SARS-CoV-2 S1 protein by using flexible electrochemical and Raman enhancing biochip. *Biosensors and Bioelectronics* **2024,** *249*, 116021.
(95) Yang, Y.; Peng, Y.; Lin, C.; Long, L.; Hu, J.; He, J.; Zeng, H.; Huang, Z.; Li, Z.-Y.; Tanemura, M.; Shi, J.; Lombardi, J. R.; Luo, X., Human ACE2-Functionalized Gold "Virus-Trap" Nanostructures for Accurate Capture of SARS-CoV-2 and Single-Virus SERS Detection. *Nano-Micro Letters* **2021,** *13* (1), 109.
(96) Peng, Y.; Li, D.; Li, Y.; Zhao, S.; Xu, M.; Zhang, W.; Teng, Z.; Liu, M.; Tanemura, M.; Yang, Y., A label-free magnetic enrichment SERS platform for ultra-sensitive detection and AI intelligent identification of novel coronavirus variants. *Chemical Engineering Journal* **2025,** *526*, 170723.
(97) Qin, Z.; Fang, X.; Liu, Y.; Wang, G.; Li, Y.; Zhang, X., Multi-phase SERS detection of SARS-CoV-2 from liquids to aerosols and pseudovirus. *Sensors and Actuators B: Chemical* **2026,** *458*, 139821.
(98) Negri, P.; Kage, A.; Nitsche, A.; Naumann, D.; Dluhy, R. A., Detection of viral nucleoprotein binding to anti-influenza aptamers via SERS. *Chemical Communications* **2011,** *47* (30), 8635-8637.
(99) Negri, P.; Chen, G.; Kage, A.; Nitsche, A.; Naumann, D.; Xu, B.; Dluhy, R. A., Direct Optical Detection of Viral Nucleoprotein Binding to an Anti-Influenza Aptamer. *Analytical Chemistry* **2012,** *84* (13), 5501-5508.
(100) Stanborough, T.; Given, F. M.; Koch, B.; Sheen, C. R.; Stowers-Hull, A. B.; Waterland, M. R.; Crittenden, D. L., Optical Detection of CoV-SARS-2 Viral Proteins to Sub-Picomolar Concentrations. *ACS Omega* **2021,** *6* (9), 6404-6413.
(101) Zhdanov, G.; Gambaryan, A.; Akhmetova, A.; Yaminsky, I.; Kukushkin, V.; Zavyalova, E., Nanoisland SERS-Substrates for Specific Detection and Quantification of Influenza A Virus. *Biosensors* **2024,** *14* (1), 20.
(102) Yang, Y.; Li, H.; Jones, L.; Murray, J.; Haverstick, J.; Naikare, H. K.; Mosley, Y.-Y. C.; Tripp, R. A.; Ai, B.; Zhao, Y., Rapid Detection of SARS-CoV-2 RNA in Human Nasopharyngeal Specimens Using Surface-Enhanced Raman Spectroscopy and Deep Learning Algorithms. *ACS Sensors* **2023,** *8* (1), 297-307.
(103) Negri, P.; Dluhy, R. A., Detection of genetic markers related to high pathogenicity in influenza by SERS. *Analyst* **2013,** *138* (17), 4877-4884.
(104) Choi, J.; Martin, S. J. H.; Tripp, R. A.; Tompkins, S. M.; Dluhy, R. A., Detection of neuraminidase stalk motifs associated with enhanced N1 subtype influenza A virulence via Raman spectroscopy. *Analyst* **2015,** *140* (22), 7748-7760.
(105) Moitra, P.; Chaichi, A.; Abid Hasan, S. M.; Dighe, K.; Alafeef, M.; Prasad, A.; Gartia, M. R.; Pan, D., Probing the mutation independent interaction of DNA probes with SARS-CoV-2 variants through a combination of surface-enhanced Raman scattering and machine learning. *Biosensors and Bioelectronics* **2022,** *208*, 114200.
(106) Yang, X.; Ding, C.; Cui, J.; Zhang, H.; Zhang, S., Magnetic-plasmonic microfluidic SERS chip for ultrasensitive RSV detection via tDNA recognition. *Applied Surface Science* **2026,** *725*, 165790.
(107) Esposito, A.; Martino, S.; Yilmaz, D.; Mangini, M.; De Stefano, L.; Corteggio, A.; Italiani, P.; Rea, I.; De Luca, A. C., PNA-SERS biosensor for label-free detection of SARS-CoV-2 genomic sequences in saliva. *Sensors and Actuators Reports* **2025,** *10*, 100369.

(108) Zhang, H.; Du, S.; Li, J.; Cheng, X.; Wang, L.; Zhang, Z., Virus gene sensing detection method based on TiO2NR @ AgNPs chip and the multifunctional SERS microspheres. *Applied Surface Science Advances* **2026,** *32*, 100920.
(109) Leong, S. X.; Leong, Y. X.; Tan, E. X.; Sim, H. Y. F.; Koh, C. S. L.; Lee, Y. H.; Chong, C.; Ng, L. S.; Chen, J. R. T.; Pang, D. W. C.; Nguyen, L. B. T.; Boong, S. K.; Han, X.; Kao, Y.-C.; Chua, Y. H.; Phan-Quang, G. C.; Phang, I. Y.; Lee, H. K.; Abdad, M. Y.; Tan, N. S.; Ling, X. Y., Noninvasive and Point-of-Care Surface-Enhanced Raman Scattering (SERS)-Based Breathalyzer for Mass Screening of Coronavirus Disease 2019 (COVID-19) under 5 min. *ACS Nano* **2022,** *16* (2), 2629-2639.
(110) Zhan, L.; Zhen, S. J.; Wan, X. Y.; Gao, P. F.; Huang, C. Z., A sensitive surface-enhanced Raman scattering enzyme-catalyzed immunoassay of respiratory syncytial virus. *Talanta* **2016,** *148*, 308-312.
(111) Filbrun, S. L.; Filbrun, A. B.; Lovato, F. L.; Oh, S. H.; Driskell, E. A.; Driskell, J. D., Chemical modification of antibodies enables the formation of stable antibody-gold nanoparticle conjugates for biosensing. *Analyst* **2017,** *142* (23), 4456-4467.
(112) Sun, Y.; Xu, L.; Zhang, F.; Song, Z.; Hu, Y.; Ji, Y.; Shen, J.; Li, B.; Lu, H.; Yang, H., A promising magnetic SERS immunosensor for sensitive detection of avian influenza virus. *Biosensors and Bioelectronics* **2017,** *89*, 906-912.
(113) Eom, G.; Hwang, A.; Kim, H.; Yang, S.; Lee, D. K.; Song, S.; Ha, K.; Jeong, J.; Jung, J.; Lim, E.-K.; Kang, T., Diagnosis of Tamiflu-Resistant Influenza Virus in Human Nasal Fluid and Saliva Using Surface-Enhanced Raman Scattering. *ACS Sensors* **2019,** *4* (9), 2282-2287.
(114) Guk, K.; Kim, H.; Lee, M.; Choi, Y.-A.; Hwang, S. G.; Han, G.; Kim, H.-N.; Kim, H.; Park, H.; Yong, D.; Kang, T.; Lim, E.-K.; Jung, J., Development of A4 antibody for detection of neuraminidase I223R/H275Y-associated antiviral multidrug-resistant influenza virus. *Nature Communications* **2020,** *11* (1), 3418.
(115) Achadu, O. J.; Abe, F.; Suzuki, T.; Park, E. Y., Molybdenum Trioxide Nanocubes Aligned on a Graphene Oxide Substrate for the Detection of Norovirus by Surface-Enhanced Raman Scattering. *ACS Applied Materials & Interfaces* **2020,** *12* (39), 43522-43534.
(116) Achadu, O. J.; Abe, F.; Li, T.-C.; Khoris, I. M.; Lee, D.; Lee, J.; Suzuki, T.; Park, E. Y., Molybdenum Trioxide Quantum Dot-Encapsulated Nanogels for Virus Detection by Surface-Enhanced Raman Scattering on a 2D Substrate. *ACS Applied Materials & Interfaces* **2021,** *13* (24), 27836-27844.
(117) Kim, H.; Kang, H.; Kim, H.-N.; Kim, H.; Moon, J.; Guk, K.; Park, H.; Yong, D.; Bae, P. K.; Park, H. G.; Lim, E.-K.; Kang, T.; Jung, J., Development of 6E3 antibody-mediated SERS immunoassay for drug-resistant influenza virus. *Biosensors and Bioelectronics* **2021,** *187*, 113324.
(118) Cha, H.; Kim, H.; Joung, Y.; Kang, H.; Moon, J.; Jang, H.; Park, S.; Kwon, H.-J.; Lee, I.-C.; Kim, S.; Yong, D.; Yoon, S.-W.; Park, S.-G.; Guk, K.; Lim, E.-K.; Park, H. G.; Choo, J.; Jung, J.; Kang, T., Surface-enhanced Raman scattering-based immunoassay for severe acute respiratory syndrome coronavirus 2. *Biosensors and Bioelectronics* **2022,** *202*, 114008.
(119) Ryu, J. S.; Lee, S. H.; Kim, H.; Kang, H.; Li, P.; Lee, J. H.; Jang, H.; Kim, S.; Kwon, H.-J.; Jung, H. S.; Jung, Y.; Lim, E.-K.; Jung, J.; Park, S.-G.; Kang, T., Advancing SARS-CoV-2 Variant Detection with High Affinity Monoclonal Antibodies and Plasmonic Nanostructure. *Advanced Functional Materials* **2024,** *34* (45), 2405340.
(120) Guan, P.-C.; Zhang, H.; Li, Z.-Y.; Xu, S.-S.; Sun, M.; Tian, X.-M.; Ma, Z.; Lin, J.-S.; Gu, M.-M.; Wen, H.; Zhang, F.-L.; Zhang, Y.-J.; Yu, G.-J.; Yang, C.; Wang, Z.-X.; Song, Y.; Li, J.-F., Rapid Point-of-Care Assay by SERS Detection of SARS-CoV-2 Virus and Its Variants. *Analytical Chemistry* **2022,** *94* (51), 17795-17802.
(121) Yu, Q.; Trinh, H. D.; Lee, Y.; Kang, T.; Chen, L.; Yoon, S.; Choo, J., SERS-ELISA using silica-encapsulated Au core-satellite nanotags for sensitive detection of SARS-CoV-2. *Sensors and Actuators B: Chemical* **2023,** *382*, 133521.

(122) Wang, C.; Wang, C.; Wang, X.; Wang, K.; Zhu, Y.; Rong, Z.; Wang, W.; Xiao, R.; Wang, S., Magnetic SERS Strip for Sensitive and Simultaneous Detection of Respiratory Viruses. *ACS Applied Materials & Interfaces* **2019,** *11* (21), 19495-19505.
(123) Wang, W.; Srivastava, S.; Garg, A.; Xiao, C.; Hawks, S.; Pan, J.; Duggal, N.; Isaacman-VanWertz, G.; Zhou, W.; Marr, L. C.; Vikesland, P. J., Digital Surface-Enhanced Raman Spectroscopy–Lateral Flow Test Dipstick: Ultrasensitive, Rapid Virus Quantification in Environmental Dust. *Environmental Science & Technology* **2024,** *58* (11), 4926-4936.
(124) Xiao, M.; Xie, K.; Dong, X.; Wang, L.; Huang, C.; Xu, F.; Xiao, W.; Jin, M.; Huang, B.; Tang, Y., Ultrasensitive detection of avian influenza A (H7N9) virus using surface-enhanced Raman scattering-based lateral flow immunoassay strips. *Analytica Chimica Acta* **2019,** *1053*, 139-147.
(125) Shen, W.; Liu, J.; Wang, C.; Wang, C.; Nie, Y.; Gu, B.; Xiao, R.; Hao, R., Development of a dual-Raman signal lateral flow immunoassay with self-assembling nanogapped SERS tags for the simultaneous monitoring of respiratory bacteria and virus. *Sensors and Actuators B: Chemical* **2024,** *417*, 136078.
(126) Sánchez-Purrà, M.; Carré-Camps, M.; de Puig, H.; Bosch, I.; Gehrke, L.; Hamad-Schifferli, K., Surface-Enhanced Raman Spectroscopy-Based Sandwich Immunoassays for Multiplexed Detection of Zika and Dengue Viral Biomarkers. *ACS Infectious Diseases* **2017,** *3* (10), 767-776.
(127) Park, H. J.; Yang, S. C.; Choo, J., Early Diagnosis of Influenza Virus A Using Surface-enhanced Raman Scattering-based Lateral Flow Assay. *Bulletin of the Korean Chemical Society* **2016,** *37* (12), 2019-2024.
(128) Meng, S.; Huang, J.; Duan, X.; Luo, Y.; He, J.; Peng, H.; Li, J.; Chen, H., Gold nanostars-based SERS-LFIA for the rapid and sensitive detection of group A rotavirus. *Spectrochimica Acta Part A: Molecular and Biomolecular Spectroscopy* **2026,** *350*, 127441.
(129) Liu, X.; Dong, Z.; Wang, H.; Wang, G.; Xiao, R., PDA-integrated Au@Ag with colorimetric/SERS dual-signal enhancement capability for flexible lateral flow immunoassay detection of respiratory viruses. *Microchimica Acta* **2025,** *192* (11), 781.
(130) Shi, X.; Yan, H.; Li, J.; Tang, Y.; Yang, S.; Yao, Y.; Wang, Y.; Chen, H.; Guo, L., Strong plasmon coupled gold nanoantennas with closed hot-spots enabled ultrasensitive lateral flow immunoassay of porcine epidemic diarrhea virus. *Biosensors and Bioelectronics* **2025,** *287*, 117699.
(131) Zhu, S.; Zhou, X.; Liu, Z.; Han, H.; Liu, X.; He, S.; Li, C.; Xiao, R., Magnetic SERS-based lateral flow immunoassay for ultrasensitive on-site detection of Chikungunya virus in mosquito vectors. *Microchimica Acta* **2026,** *193* (4), 224.
(132) Jia, X.; Liu, Z.; Zhou, J.; Cao, C.; Hao, Y.; Chen, J.; Han, H.; Liang, J.; Zhao, Z.; Wang, Y.; Niu, Z.; Xiao, R., Universal and highly sensitive detection of influenza A virus and streptococcus pneumoniae using WGA-modified magnetic SERS nanotags-based lateral flow assay. *Nanomedicine: Nanotechnology, Biology and Medicine* **2025,** *69*, 102853.
(133) Yi, X.; Song, Y.; Xu, X.; Peng, D.; Wang, J.; Qie, X.; Lin, K.; Yu, M.; Ge, M.; Wang, Y.; Zhang, D.; Yang, Q.; Wang, M.; Huang, W. E., Development of a Fast Raman-Assisted Antibiotic Susceptibility Test (FRAST) for the Antibiotic Resistance Analysis of Clinical Urine and Blood Samples. *Analytical Chemistry* **2021,** *93* (12), 5098-5106.
(134) Wang, J.; Zhou, Q.; Lowry, K.; Howard, C. B.; Trau, M., A digital SERS platform powered by bispecific antibody fragments for SARS-CoV-2 diagnostics. *Biosensors and Bioelectronics* **2026,** *294*, 118252.
(135) Pramanik, A.; Gao, Y.; Patibandla, S.; Mitra, D.; McCandless, M. G.; Fassero, L. A.; Gates, K.; Tandon, R.; Ray, P. C., Aptamer Conjugated Gold Nanostar-Based Distance-Dependent Nanoparticle Surface Energy Transfer Spectroscopy for Ultrasensitive Detection and Inactivation of Corona Virus. *The Journal of Physical Chemistry Letters* **2021,** *12* (8), 2166-2171.
(136) Chen, H.; Park, S.-G.; Choi, N.; Moon, J.-I.; Dang, H.; Das, A.; Lee, S.; Kim, D.-G.; Chen, L.; Choo, J., SERS imaging-based aptasensor for ultrasensitive and reproducible detection of influenza virus A. *Biosensors and Bioelectronics* **2020,** *167*, 112496.

(137) Tikhonova, D.; Andreev, E.; Akhmetova, A.; Meshcheryakova, N.; Zaborova, O.; Mukhametova, L.; Aliyeva, R.; Kravchenko, E.; Boravleva, E.; Dubkov, S.; Eremin, S.; Yaminsky, I.; Nechaev, A.; Zavyalova, E., Specific determination of influenza virus A by SERS-active spike-like track-etched membranes. *Analytica Chimica Acta* **2026,** *1388*, 345100.
(138) Lee, J. H.; Xu, P. F.; Domaille, D. W.; Choi, C.; Jin, S.; Cha, J. N., M13 Bacteriophage as Materials for Amplified Surface Enhanced Raman Scattering Protein Sensing. *Advanced Functional Materials* **2014,** *24* (14), 2079-2084.
(139) Pramanik, A.; Mayer, J.; Sinha, S. S.; Sharma, P. C.; Patibandla, S.; Gao, Y.; Corby, L. R.; Bates, J. T.; Bierdeman, M. A.; Tandon, R.; Seshadri, R.; Ray, P. C., Human ACE2 Peptide-Attached Plasmonic-Magnetic Heterostructure for Magnetic Separation, Surface Enhanced Raman Spectroscopy Identification, and Inhibition of Different Variants of SARS-CoV-2 Infections. *ACS Applied Bio Materials* **2022,** *5* (9), 4454-4464.
(140) Song, K.; Xue, W.; Li, X.; Chang, Y.; Liu, M., Self-Assembly of Single-Virus SERS Hotspots for Highly Sensitive In Situ Detection of SARS-CoV-2 on Solid Surfaces. *Analytical Chemistry* **2024,** *96* (21), 8830-8836.
(141) Jiao, C.; Liang, X.; Wu, X.; Shang, Y.; Wu, Y.; Zhang, F.; Liu, X.; Zeng, J.; Yang, C., SERS Platform for Integrated Enrichment, Isolation, and Identification of Multiple Respiratory Viruses in a Single Assay Using 3D Stereoscopic SERS Tags and Flocked Swabs. *Analytical Chemistry* **2024,** *96* (32), 13042-13049.
(142) Xu, S. P.; Ji, X. H.; Xu, W. Q.; Li, X. L.; Wang, L. Y.; Bai, Y. B.; Zhao, B.; Ozaki, Y., Immunoassay using probe-labelling immunogold nanoparticles with silver staining enhancement via surface-enhanced Raman scattering. *Analyst* **2004,** *129* (1), 63-68.
(143) Baniukevic, J.; Hakki Boyaci, I.; Goktug Bozkurt, A.; Tamer, U.; Ramanavicius, A.; Ramanaviciene, A., Magnetic gold nanoparticles in SERS-based sandwich immunoassay for antigen detection by well oriented antibodies. *Biosensors and Bioelectronics* **2013,** *43*, 281-288.
(144) Neng, J.; Harpster, M. H.; Wilson, W. C.; Johnson, P. A., Surface-enhanced Raman scattering (SERS) detection of multiple viral antigens using magnetic capture of SERS-active nanoparticles. *Biosensors and Bioelectronics* **2013,** *41*, 316-321.
(145) Kamińska, A.; Witkowska, E.; Winkler, K.; Dzięcielewski, I.; Weyher, J. L.; Waluk, J., Detection of Hepatitis B virus antigen from human blood: SERS immunoassay in a microfluidic system. *Biosensors and Bioelectronics* **2015,** *66*, 461-467.
(146) Karn-orachai, K.; Sakamoto, K.; Laocharoensuk, R.; Bamrungsap, S.; Songsivilai, S.; Dharakul, T.; Miki, K., Extrinsic surface-enhanced Raman scattering detection of influenza A virus enhanced by two-dimensional gold@silver core–shell nanoparticle arrays. *RSC Advances* **2016,** *6* (100), 97791-97799.
(147) Camacho, S. A.; Sobral-Filho, R. G.; Aoki, P. H. B.; Constantino, C. J. L.; Brolo, A. G., Zika Immunoassay Based on Surface-Enhanced Raman Scattering Nanoprobes. *ACS Sensors* **2018,** *3* (3), 587-594.
(148) Zhang, M.; Li, X.; Pan, J.; Zhang, Y.; Zhang, L.; Wang, C.; Yan, X.; Liu, X.; Lu, G., Ultrasensitive detection of SARS-CoV-2 spike protein in untreated saliva using SERS-based biosensor. *Biosensors and Bioelectronics* **2021,** *190*, 113421.
(149) Antoine, D.; Mohammadi, M.; Vitt, M.; Dickie, J. M.; Jyoti, S. S.; Tilbury, M. A.; Johnson, P. A.; Wawrousek, K. E.; Wall, J. G., Rapid, Point-of-Care scFv-SERS Assay for Femtogram Level Detection of SARS-CoV-2. *ACS Sensors* **2022,** *7* (3), 866-873.
(150) Bistaffa, M. J.; Camacho, S. A.; Pazin, W. M.; Constantino, C. J. L.; Oliveira, O. N.; Aoki, P. H. B., Immunoassay platform with surface-enhanced resonance Raman scattering for detecting trace levels of SARS-CoV-2 spike protein. *Talanta* **2022,** *244*, 123381.
(151) Shim, J.-E.; Kim, Y. J.; Choe, J.-H.; Lee, T. G.; You, E.-A., Single-Nanoparticle-Based Digital SERS Sensing Platform for the Accurate Quantitative Detection of SARS-CoV-2. *ACS Applied Materials & Interfaces* **2022,** *14* (34), 38459-38470.

(152) Atta, S.; Zhao, Y. H.; Li, J. Q.; Vo-Dinh, T., Dual-Modal Colorimetric and Surface-Enhanced Raman Scattering (SERS)-Based Lateral Flow Immunoassay for Ultrasensitive Detection of SARS-CoV-2 Using a Plasmonic Gold Nanocrown. *Analytical Chemistry* **2024,** *96* (12), 4783-4790.
(153) Lee, S.; Lee, S.; Park, W.; Lee, S.; Kwon, S.; Oh, M. J.; Haddadnezhad, M.; Jung, I.; Kim, B.; Park, J.; Shin, K. S.; Lee, H.; Yoo, J.; Kim, W.-K.; Park, S., Plasmonic Annular Nanotrenches with 1 nm Nanogaps for Detection of SARS-CoV-2 Using SERS-Based Immunoassay. *Nano Letters* **2024,** *24* (14), 4233-4240.
(154) Sun, K.; Zhang, H.; Duan, L.; Chen, H.; Li, R.; Wang, Y.; Yan, C.; Liu, Y.; Yao, J.; Huang, X.; Qu, L.; Han, C., Silver Nanotripod Array-Based Sandwich Immunosensor for Sensitive SERS Detection of SARS-CoV-2 Spike Protein and Multiplexed Protein Samples. *Analysis & Sensing* **2026,** *6* (3), e202500215.
(155) Nwahara, N.; Niaz, S.; Managa, M.; Nkanga, C. I.; Adegoke, O.; Achadu, O. J., Nanoplasmonic core–shell nanoraspberry chip for ultrasensitive surface-enhanced Raman scattering detection of SARS-CoV-2: a modular nanobiosensor for respiratory virus diagnostics. *Sensors & Diagnostics* **2026,** *5* (4), 567-578.
(156) Gordon, J.; Mousavi, S. D.; Li, J.; Filippone, N.; Walter, L.; Cheng, H.-W.; Skeete, Z.; Feldman, H.; Hakimi, S.; Cappuccio, K.; Wang, S.; Hader, M.; Shang, G.; Turner, J.; Cameron, A.; Bane, S.; Poliks, M.; Lu, S.; Yuan, T. T.; Zhong, C.-J., Plasmonic Nanoprobe-Enabled SERS Detection of SARS-CoV-2 Proteins and Virus Samples on Wax-Printed Paper Substrates. *Analytical Chemistry* **2025,** *97* (39), 21303-21313.
(157) Xu, S. S.; Wu, X. H.; Wu, L.; Zhai, J. M.; Li, S. J.; Kou, Y. C.; Peng, W.; Zheng, Q. N.; Tian, J. H.; Zhang, Y. J.; Li, J. F., Systematic Optimization of Universal Real-Time Hypersensitive Fast Detection Method for HBsAg in Serum Based on SERS. *Analytical Chemistry* **2024,** *96* (17), 6784-6793.
(158) Zhang, Y.; Wu, G.; Wei, J.; Ding, Y.; Wei, Y.; Liu, Q.; Chen, H., Rapid and sensitive detection of rotavirus by surface-enhanced Raman scattering immunochromatography. *Microchimica Acta* **2021,** *188* (1), 3.
(159) Pang, Y.; Ding, Q.; Xu, L., A novel surface-enhanced Raman based molecular identification platform for multiplexed and highly accurate clinical diagnosis of viral diseases. *Journal of Materials Chemistry B* **2024,** *12* (31), 7461-7462.
(160) Li, J.; Guan, R.; Wuethrich, A.; Yan, M.; Cheng, J.; Liu, G.; Zhan, J.; Trau, M.; Sun, Y., High Accuracy of Clinical Verification of Electrohydrodynamic-Driven Nanobox-on-Mirror Platform for Molecular Identification of Respiratory Viruses. *Analytical Chemistry* **2024,** *96* (11), 4495-4504.
(161) Huang, Q.; Zhou, N.; Peng, J.; Zeng, X.; Du, L.; Zhao, Y.; Luo, X., Sensitivity-improved SERS detection of SARS-CoV-2 spike protein by Au NPs/COFs integrated with catalytic-hairpin-assembly amplification technology. *Analytica Chimica Acta* **2024,** *1318*, 342924.
(162) Zhang, C.; Ren, H.; Luo, J.; Liang, L.; Wei, Y.; Luo, Q.; Zhang, K.; Liao, X., Dual-mode ECL/SERS ratiometric sensing of 3CLpro via enzyme-gated, entropy-driven DNA assembly on $CsPbBr_3$@PDA@Au. *Colloids and Surfaces B: Biointerfaces* **2026,** *266*, 115782.
(163) Shen, H.; Xie, K.; Huang, L.; Wang, L.; Ye, J.; Xiao, M.; Ma, L.; Jia, A.; Tang, Y., A novel SERS-based lateral flow assay for differential diagnosis of wild-type pseudorabies virus and gE-deleted vaccine. *Sensors and Actuators B: Chemical* **2019,** *282*, 152-157.
(164) González-Cabaleiro, L.; Fernández-Lodeiro, C.; Vázquez-Iglesias, L.; Soriano-Maldonado, P.; van Raaij, M. J.; Bodelón, G.; Pérez-Juste, J.; Pastoriza-Santos, I., Pushing the Limits of Lateral Flow Immunoassay by Digital SERS for the Ultralow Detection of SARS-CoV-2 Virus. *Small Science* **2024,** *4* (11), 2400259.
(165) Liu, X.; Yang, X.; Wang, C.; Liu, Q.; Ding, Y.; Xu, S.; Wang, G.; Xiao, R., A nanogap-enhanced SERS nanotag–based lateral flow assay for ultrasensitive and simultaneous monitoring of SARS-CoV-2 S and NP antigens. *Microchimica Acta* **2024,** *191* (2), 104.
(166) Li, Z.; Zhu, A.; Zhao, B.; Zhang, Y.; Zhang, Q.; Zhou, H.; Liu, T.; Li, J.; Zhou, X.; Shi, Q.; Li, Y.; Liang, M.; Zhang, X.; Lu, D.; Li, X., Establishment of a Raman nanosphere based

immunochromatographic system for the combined detection of influenza A and B viruses' antigens on a single T-line. *Nanotechnology* **2024,** *35* (50), 505501.
(167) Joung, Y.; Kim, K.; Lee, S.; Chun, B.-S.; Lee, S.; Hwang, J.; Choi, S.; Kang, T.; Lee, M.-K.; Chen, L.; Choo, J., Rapid and Accurate On-Site Immunodiagnostics of Highly Contagious Severe Acute Respiratory Syndrome Coronavirus 2 Using Portable Surface-Enhanced Raman Scattering-Lateral Flow Assay Reader. *ACS Sensors* **2022,** *7* (11), 3470-3480.
(168) Xiong, R.; Su, J.; Wang, S.; Tong, Z.; Zhu, R.; Liu, Y.; Xiao, R., Electronic structure engineered Fe-ZnO@au-DTNB nanotags for ultrasensitive SERS-based lateral flow immunoassay. *Chemical Engineering Journal* **2026,** *534*, 175105.
(169) Liu, Z.; Li, Y.; Wang, F.; Fang, M.; Sun, Y.; Kang, X.; Jiang, T.; Xiao, R., A signal-amplified SERS-LFA leveraging multivalent nanobodies on 2D substrates for clinical detection of influenza A virus. *Sensors and Actuators B: Chemical* **2026,** *453*, 139466.
(170) Zhao, S.; Li, Y.-Y.; Lin, C.-L.; Xu, M.-M.; Zhang, W.-D.; Li, D.; Peng, Y.-S.; Tanemura, M.; Zhao, T.-S.; Yang, Y., Deep Learning–Driven Surface-Enhanced Raman Scattering–Lateral Flow Immunoassay With Au Nanostars for High-Accuracy Respiratory Virus Detection. *Rare Metals* **2026,** *45* (2), e70072.
(171) Zhao, S.; Xu, M.; Lin, C.; Zhang, W.; Li, D.; Peng, Y.; Tanemura, M.; Yang, Y., AI-Driven Comprehensive SERS-LFIA System: Improving Virus Automated Diagnostics Through SERS Image Recognition and Deep Learning. *Biosensors* **2025,** *15* (7), 458.
(172) Lu, M.; Qi, J.; Joung, Y.; Wang, R.; Choo, J., Electrophoresis-assisted SERS-LFA strip for simultaneous HIV and HBV detection. *Biosensors and Bioelectronics* **2026,** *306*, 118701.
(173) Li, J.; Yu, Q.; Li, Z.; Xu, Z.; Wang, B.; Wu, T.; Li, G.; Wang, C.; Gu, B., Dendritic Mesoporous SiO2-Based SERS-Nanozyme-Mediated Immunochromatographic Detection of Foodborne Viruses with Enhanced Sensitivity across Multiple Scenarios. *Analytical Chemistry* **2025,** *97* (37), 20255-20265.
(174) Kukushkin, V. I.; Ivanov, N. M.; Novoseltseva, A. A.; Gambaryan, A. S.; Yaminsky, I. V.; Kopylov, A. M.; Zavyalova, E. G., Highly sensitive detection of influenza virus with SERS aptasensor. *PLOS One* **2019,** *14* (4), e0216247.
(175) Chen, H.; Park, S.-G.; Choi, N.; Kwon, H.-J.; Kang, T.; Lee, M.-K.; Choo, J., Sensitive Detection of SARS-CoV-2 Using a SERS-Based Aptasensor. *ACS Sensors* **2021,** *6* (6), 2378-2385.
(176) Huang, G.; Zhao, H.; Li, P.; Liu, J.; Chen, S.; Ge, M.; Qin, M.; Zhou, G.; Wang, Y.; Li, S.; Cheng, Y.; Huang, Q.; Wang, J.; Wang, H.; Yang, L., Construction of Optimal SERS Hotspots Based on Capturing the Spike Receptor-Binding Domain (RBD) of SARS-CoV-2 for Highly Sensitive and Specific Detection by a Fish Model. *Analytical Chemistry* **2021,** *93* (48), 16086-16095.
(177) Neng, J.; Harpster, M. H.; Zhang, H.; Mecham, J. O.; Wilson, W. C.; Johnson, P. A., A versatile SERS-based immunoassay for immunoglobulin detection using antigen-coated gold nanoparticles and malachite green-conjugated protein A/G. *Biosensors and Bioelectronics* **2010,** *26* (3), 1009-1015.
(178) Liu, H.; Dai, E.; Xiao, R.; Zhou, Z.; Zhang, M.; Bai, Z.; Shao, Y.; Qi, K.; Tu, J.; Wang, C.; Wang, S., Development of a SERS-based lateral flow immunoassay for rapid and ultra-sensitive detection of anti-SARS-CoV-2 IgM/IgG in clinical samples. *Sensors and Actuators B: Chemical* **2021,** *329*, 129196.
(179) Chen, S.; Meng, L.; Wang, L.; Huang, X.; Ali, S.; Chen, X.; Yu, M.; Yi, M.; Li, L.; Chen, X.; Yuan, L.; Shi, W.; Huang, G., SERS-based lateral flow immunoassay for sensitive and simultaneous detection of anti-SARS-CoV-2 IgM and IgG antibodies by using gap-enhanced Raman nanotags. *Sensors and Actuators B: Chemical* **2021,** *348*, 130706.
(180) Srivastav, S.; Dankov, A.; Adanalic, M.; Grzeschik, R.; Tran, V.; Pagel-Wieder, S.; Gessler, F.; Spreitzer, I.; Scholz, T.; Schnierle, B.; Anastasiou, O. E.; Dittmer, U.; Schlücker, S., Rapid and Sensitive SERS-Based Lateral Flow Test for SARS-CoV2-Specific IgM/IgG Antibodies. *Analytical Chemistry* **2021,** *93* (36), 12391-12399.

(181) Liang, Y.; Gong, J.-L.; Huang, Y.; Zheng, Y.; Jiang, J.-H.; Shen, G.-L.; Yu, R.-Q., Biocompatible core-shell nanoparticle-based surface-enhanced Raman scattering probes for detection of DNA related to HIV gene using silica-coated magnetic nanoparticles as separation tools. *Talanta* **2007,** *72* (2), 443-449.

(182) Malvadkar, N. A.; Demirel, G.; Poss, M.; Javed, A.; Dressick, W. J.; Demirel, M. C., Fabrication and Use of Electroless Plated Polymer Surface-Enhanced Raman Spectroscopy Substrates for Viral Gene Detection. *Journal of Physical Chemistry C* **2010,** *114* (24), 10730-10738.

(183) Zhang, H.; Harpster, M. H.; Park, H. J.; Johnson, P. A.; Wilson, W. C., Surface-Enhanced Raman Scattering Detection of DNA Derived from the West Nile Virus Genome Using Magnetic Capture of Raman-Active Gold Nanoparticles. *Analytical Chemistry* **2011,** *83* (1), 254-260.

(184) Ganbold, E.-O.; Kang, T.; Lee, K.; Lee, S. Y.; Joo, S.-W., Aggregation effects of gold nanoparticles for single-base mismatch detection in influenza A (H1N1) DNA sequences using fluorescence and Raman measurements. *Colloids and Surfaces B-Biointerfaces* **2012,** *93*, 148-153.

(185) Zhang, H.; Harpster, M. H.; Wilson, W. C.; Johnson, P. A., Surface-Enhanced Raman Scattering Detection of DNAs Derived from Virus Genomes Using Au-Coated Paramagnetic Nanoparticles. *Langmuir* **2012,** *28* (8), 4030-4037.

(186) Li, M.; Cushing, S. K.; Liang, H.; Suri, S.; Ma, D.; Wu, N., Plasmonic Nanorice Antenna on Triangle Nanoarray for Surface-Enhanced Raman Scattering Detection of Hepatitis B Virus DNA. *Analytical Chemistry* **2013,** *85* (4), 2072-2078.

(187) Wang, H.-N.; Fales, A. M.; Zaas, A. K.; Woods, C. W.; Burke, T.; Ginsburg, G. S.; Vo-Dinh, T., Surface-enhanced Raman scattering molecular sentinel nanoprobes for viral infection diagnostics. *Analytica Chimica Acta* **2013,** *786*, 153-158.

(188) Ngo, H. T.; Wang, H.-N.; Fales, A. M.; Nicholson, B. P.; Woods, C. W.; Vo-Dinh, T., DNA bioassay-on-chip using SERS detection for dengue diagnosis. *Analyst* **2014,** *139* (22), 5655-5659.

(189) Pang, Y.; Wang, J.; Xiao, R.; Wang, S., SERS molecular sentinel for the RNA genetic marker of PB1-F2 protein in highly pathogenic avian influenza (HPAI) virus. *Biosensors and Bioelectronics* **2014,** *61*, 460-465.

(190) Awad, H.; El-Brolossy, T. A.; Abdallah, T.; Osman, A.; Negm, S.; Mansour, O. I.; Girgis, S. A.; Hafez, H. M.; Zaki, A. M.; Talaat, H., Accurate and reliable surface-enhanced Raman spectroscopy assay for early detection of SARS-CoV-2 RNA with exceptional sensitivity. *Spectrochimica Acta Part A: Molecular and Biomolecular Spectroscopy* **2024,** *315*, 124184.

(191) Hoan, T. N.; Naveen, G.; Andrew, M. F.; Steve, M. T.; Tuan, V.-D. In *DNA detection and single nucleotide mutation identification using SERS for molecular diagnostics and global health*, Proc.SPIE, 2017; p 100540C.

(192) Jang, A. S.; Praveen Kumar, P. P.; Lim, D.-K., Attomolar Sensitive Magnetic Microparticles and a Surface-Enhanced Raman Scattering-Based Assay for Detecting SARS-CoV-2 Nucleic Acid Targets. *ACS Applied Materials & Interfaces* **2022,** *14* (1), 138-149.

(193) Yin, B.; Ho, W. K. H.; Zhang, Q.; Li, C.; Huang, Y.; Yan, J.; Yang, H.; Hao, J.; Wong, S. H. D.; Yang, M., Magnetic-Responsive Surface-Enhanced Raman Scattering Platform with Tunable Hot Spot for Ultrasensitive Virus Nucleic Acid Detection. *ACS Applied Materials & Interfaces* **2022,** *14* (3), 4714-4724.

(194) Hoan, T. N.; Pietro, S.; Hsin-Neng, W.; Bridget, M. C.; Andrew, M. F.; Tuan, V.-D. In *Plasmonic nanochip for SERS chemical and biomedical sensing*, Proc.SPIE, 2017; p 102150G.

(195) Zengin, A.; Tamer, U.; Caykara, T., SERS detection of hepatitis B virus DNA in a temperature-responsive sandwich-hybridization assay. *Journal of Raman Spectroscopy* **2017,** *48* (5), 668-672.

(196) Fu, X.; Cheng, Z.; Yu, J.; Choo, P.; Chen, L.; Choo, J., A SERS-based lateral flow assay biosensor for highly sensitive detection of HIV-1 DNA. *Biosensors and Bioelectronics* **2016,** *78*, 530-537.

(197) Wang, X.; Choi, N.; Cheng, Z.; Ko, J.; Chen, L.; Choo, J., Simultaneous Detection of Dual Nucleic Acids Using a SERS-Based Lateral Flow Assay Biosensor. *Analytical Chemistry* **2017,** *89* (2), 1163-1169.

(198) Zhang, D.; Huang, L.; Liu, B.; Ge, Q.; Dong, J.; Zhao, X., Rapid and Ultrasensitive Quantification of Multiplex Respiratory Tract Infection Pathogen via Lateral Flow Microarray based on SERS Nanotags. *Theranostics* **2019,** *9* (17), 4849-4859.
(199) Gao, Y.; Han, Y.; Wang, C.; Qiang, L.; Gao, J.; Wang, Y.; Liu, H.; Han, L.; Zhang, Y., Rapid and sensitive triple-mode detection of causative SARS-CoV-2 virus specific genes through interaction between genes and nanoparticles. *Analytica Chimica Acta* **2021,** *1154*, 338330.
(200) Chen, H.; Park, S.-K.; Joung, Y.; Kang, T.; Lee, M.-K.; Choo, J., SERS-based dual-mode DNA aptasensors for rapid classification of SARS-CoV-2 and influenza A/H1N1 infection. *Sensors and Actuators B: Chemical* **2022,** *355*, 131324.
(201) Dai, Q.; Huang, J.; Qiu, X.; Chen, X.; Li, Y., SERS/EC Dual-Mode Sensing System for the Assay of SARS-CoV-2 RNA by Using Single Gold Nanowires as a Platform. *ACS Applied Nano Materials* **2024,** *7* (6), 6369-6379.
(202) Kissell, L. N.; Liu, H.; Sheokand, M.; Vang, D.; Kachroo, P.; Strobbia, P., Direct Detection of Tobacco Mosaic Virus in Infected Plants with SERS-Sensing Hydrogels. *ACS Sensors* **2024,** *9* (1), 514-523.
(203) Isola, N. R.; Stokes, D. L.; Vo-Dinh, T., Surface-Enhanced Raman Gene Probe for HIV Detection. *Analytical Chemistry* **1998,** *70* (7), 1352-1356.
(204) Wabuyele, M. B.; Vo-Dinh, T., Detection of human immunodeficiency virus type 1 DNA sequence using plasmonics nanoprobes. *Analytical Chemistry* **2005,** *77* (23), 7810-7815.
(205) Restaino, S. M.; White, I. M., Real-time multiplexed PCR using surface enhanced Raman spectroscopy in a thermoplastic chip. *Lab on a Chip* **2018,** *18* (5), 832-839.
(206) Wu, Y.; Choi, N.; Chen, H.; Dang, H.; Chen, L.; Choo, J., Performance Evaluation of Surface-Enhanced Raman Scattering–Polymerase Chain Reaction Sensors for Future Use in Sensitive Genetic Assays. *Analytical Chemistry* **2020,** *92* (3), 2628-2634.
(207) Wu, Y.; Dang, H.; Park, S.-G.; Chen, L.; Choo, J., SERS-PCR assays of SARS-CoV-2 target genes using Au nanoparticles-internalized Au nanodimple substrates. *Biosensors and Bioelectronics* **2022,** *197*, 113736.
(208) Song, C.; Liu, Y.; Jiang, X.; Zhang, J.; Dong, C.; Li, J.; Wang, L., Ultrasensitive SERS determination of avian influenza A H7N9 virus via exonuclease III-assisted cycling amplification. *Talanta* **2019,** *205*, 120137.
(209) Zhu, C. S.; Xiong, S. Y.; Wang, C. X.; Dong, P. T.; Wu, X. Z., SERS-Based Lateral Flow Immunoassay Combined with Enzymatic Recombinase Amplification for Quantitative, High-Sensitive Detection of Influenza A Virus. *ACS Applied Nano Materials* **2024,** *7* (3), 3373-3384.
(210) Gong, Y.; Yang, T.; Zhou, J.; Wei, J.; Ren, H.; Tang, Q.; Zhang, K.; Liao, X., Self-validated SARS-CoV-2 detection via an automated DNA molecular machine-regulated ECL/SERS signal switching platform. *Biosensors and Bioelectronics* **2026,** *308*, 118777.
(211) Choi, J.-H.; Shin, M.; Yang, L.; Conley, B.; Yoon, J.; Lee, S.-N.; Lee, K.-B.; Choi, J.-W., Clustered Regularly Interspaced Short Palindromic Repeats-Mediated Amplification-Free Detection of Viral DNAs Using Surface-Enhanced Raman Spectroscopy-Active Nanoarray. *ACS Nano* **2021,** *15* (8), 13475-13485.
(212) Su, A.; Liu, Y.; Cao, X.; Xu, W.; Liang, C.; Xu, S., A universal CRISPR/Cas12a-mediated AuNPs aggregation-based surface-enhanced Raman scattering (CRISPR/Cas-SERS) platform for virus gene detection. *Sensors and Actuators B: Chemical* **2022,** *369*, 132295.
(213) Liu, Y.; Gou, S.; Qiu, L.; Xu, Z.; Yang, H.; Yang, S.; Zhao, Y., A CRISPR/Cas12a-powered gold/nickel foam surface-enhanced Raman spectroscopy biosensor for nucleic acid specific detection in foods. *Analyst* **2024,** *149* (17), 4343-4350.
(214) Liu, Z.; Qi, J.; Sun, L.; Su, J.; Li, W.; Wu, L.; Liang, Y.; Wei, C.; He, F.; Han, Y.; Sun, Y.; Yan, H.; Li, H.; Xiao, R., Oxygen-Vacancy-Engineered WOX Nanowire-Based Surface-Enhanced Raman Scattering Biosensor with Lyophilized CRISPR/Cas13a Platform for CHIKV Detection. *ACS Sensors* **2026,** *11* (5), 4132-4144.

(215) Guo, X.-M.; Fu, S.-Y.; Wang, Y.-Q.; Chen, Y.; Li, H.; Zhang, Y.-J.; Lin, J.-S.; Li, J.-F., Background-Free Rolling Circle Amplification for SERS Bioassay Using a Chimeric Hairpin-Integrated CRISPR/Cas12a System. *Analytical Chemistry* **2025,** *97* (37), 20557-20566.

(216) Zhang, J.; Zhang, W.; Yan, J.; Zhang, Y.; Zheng, J.; Liu, Y.; Gu, N.; Han, K., A proximity induced strand displacement amplification and CRISPR/Cas12a-based SERS assay for ultrasensitive detection of dengue virus. *Biosensors and Bioelectronics* **2026,** *304*, 118651.

(217) Wang, W.; Sun, J.; Gao, Y.; Jia, X. x.; Ye, Y.; Ren, S.; Peng, Y.; Han, D.; Zhou, H.; Gao, Z.; Sun, X., Ultra-sensitive detection of norovirus using a three-in-one CRISPR platform based on a DNA hydrogel and composite functional nanomaterials. *Journal of Hazardous Materials* **2025,** *482*, 136523.

(218) Wang, B.; Zhao, R.; Liu, Z.; Liu, X.; Han, H.; Tu, J.; Xiao, R., Point-of-Care diagnostic platform for H1N1: Merging CRISPR/Cas13a specificity with MIRA rapid amplification and SERS quantitative analysis based on immunochromatographic strips. *Sensors and Actuators B: Chemical* **2025,** *445*, 138520.

(219) Song, C.; Zhang, J.; Liu, Y.; Guo, X.; Guo, Y.; Jiang, X.; Wang, L., Highly sensitive SERS assay of DENV gene via a cascade signal amplification strategy of localized catalytic hairpin assembly and hybridization chain reaction. *Sensors and Actuators B: Chemical* **2020,** *325*, 128970.

(220) Tian, C.; Zhao, L.; Qi, G.; Zhu, J.; Zhang, S., One-pot and rapid detection of SARS-CoV-2 viral particles in environment using SERS aptasensor based on a locking amplifier. *Sensors and Actuators B: Chemical* **2022,** *371*, 132445.

(221) Zhang, J.; Miao, X.; Song, C.; Chen, N.; Xiong, J.; Gan, H.; Ni, J.; Zhu, Y.; Cheng, K.; Wang, L., Non-enzymatic signal amplification-powered point-of-care SERS sensor for rapid and ultra-sensitive assay of SARS-CoV-2 RNA. *Biosensors and Bioelectronics* **2022,** *212*, 114379.

(222) Jaitpal, S.; Ng, K. W.; San Juan, A. M.; Martinez, C.; Phillips, C.; Tripathy, S.; Mabbott, S., DNA-directed formation of plasmonic core–satellite nanostructures for quantification of hepatitis C viral RNA. *Chemical Science* **2024,** *15* (21), 8112-8126.

(223) Zhang, J.; Chen, Z.; Lv, H.; Liang, J.; Yan, C.; Song, C.; Wang, L., Rapid and accurate SERS assay of disease-related nucleic acids based on isothermal cascade signal amplifications of CRISPR/Cas13a system and catalytic hairpin assembly. *Biosensors and Bioelectronics* **2024,** *253*, 116196.

(224) Wang, Y.; Yin, H.; Qi, X.; Wang, C.; Li, B.; Qian, B.; Zou, M.; Xue, F., Rapid detection of African swine fever virus via SERS probe-modified sandwich hybridization assay. *Microchimica Acta* **2024,** *191* (10), 589.

(225) Naher, L.; Quarin, S. M.; Vang, D.; Strobbia, P., Lyophilizing SERS biosensors to enable translation into an easy-to-use assay. *Analytical Methods* **2024,** *16* (44), 7613-7623.
